# Structurally Divergent Lithium Catalyzed Friedel–Crafts Reactions on Oxetan‐3‐ols: Synthesis of 3,3‐Diaryloxetanes and 2,3‐Dihydrobenzofurans

**DOI:** 10.1002/chem.201604031

**Published:** 2016-10-10

**Authors:** Rosemary A. Croft, James J. Mousseau, Chulho Choi, James A. Bull

**Affiliations:** ^1^ Department of ChemistryImperial College London, South KensingtonLondonSW7 2AZUK; ^2^ Pfizer Global Research and Development445 Eastern Point Rd.GrotonCT 06340USA

**Keywords:** carbocations, homogeneous catalysis, lithium, oxetanes, oxygen heterocycles

## Abstract

The first examples of 3,3‐diaryloxetanes are prepared in a lithium‐catalyzed and substrate dependent divergent Friedel–Crafts reaction. *para*‐Selective Friedel–Crafts reactions of phenols using oxetan‐3‐ols afford 3,3‐diaryloxetanes by displacement of the hydroxy group. These constitute new isosteres for benzophenones and diarylmethanes. Conversely, *ortho*‐selective Friedel–Crafts reactions of phenols afford 3‐aryl‐3‐hydroxymethyl‐dihydrobenzofurans by tandem alkylation–ring‐opening reactions; the outcome of the reaction diverging to structurally distinct products dependent on the substrate regioselectivity. Further reactivity of the oxetane products is demonstrated, suitable for incorporation into drug discovery efforts.

## Introduction

**Figure 1:**
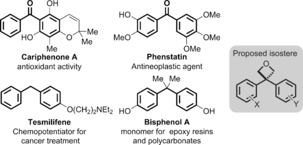
Some important benzophenones and diarylmethanes and proposed oxetane replacement.

The use of bioisosteres to replace problematic functionality whilst maintaining advantageous characteristics is an important strategy in drug discovery.^1^ The implementation of new isosteres provides access to novel chemical as well as intellectual property (IP) space, and presents interesting property modulation of biologically significant groups.^2^ Diarylketones display important biological activity and are prevalent in natural products (Figure 1).^3^, ^4^ However, their use in medicinal chemistry efforts suffers from issues of metabolic and chemical stability due to the electrophilic carbonyl, and lack of appropriate isosteres. Indeed, benzophenones are commonly exploited as photoactivatable cross‐linkers in chemical biology.^5^ Similarly, diarylmethane derivatives provide varied biological activity,^6^ for example tesmilifene has shown activity as a chemopotentiator in clinical studies including for metastatic breast cancer.^7^ However, the methylene group can often present a metabolic liability.^8^ The incorporation of blocking substituents, such as *gem*‐dimethyl groups, can improve metabolic stability, but deliver a negative impact on a compounds physicochemical properties, such as lipophilicity (LogD) and solubility. A similar motif appears in bisphenol A (BPA), used in bulk as a monomer in polycarbonates to ensure optically clarity, and in other diverse consumer products.^9^ This compound is controversial due to potential interference with the endocrine system and is banned in baby bottles, for example, in many countries. Due to these varied functions diaryl motifs have been the subject of wide ranging synthetic efforts.^10^

Recently, Carreira has presented oxetanes as replacement motifs for *gem*‐dimethyl and carbonyl derivatives due to similar spatial properties and lone pair arrangements.^11^, ^12^ In drug discovery settings, the incorporation of the polar, low molecular weight oxetane moiety has often afforded compounds with enhanced properties such as improved metabolic stability, solubility and lipophilicity.^13^ As part of our interest in the synthesis of novel oxetane derivatives,^14^ we were intrigued by possible approaches to 3,3‐diaryloxetanes, designed to provide isosteres for diarylketone and diarylmethane structures with improved properties for drug discovery. At the outset of this work, there were no reported examples of disubstituted 3,3‐diaryloxetanes in the literature.^15^, ^16^ Indeed, internal efforts within Pfizer corroborated this observation, with this motif being recognized as difficult to access.

Herein we report the synthesis of 3,3‐diaryloxetanes, prepared by a lithium‐catalyzed Friedel–Crafts reaction of phenols with readily accessible oxetan‐3‐ols. We also disclose an efficient synthesis of 3‐aryl‐3‐hydroxymethyl‐dihydrobenzofuran derivatives using the same conditions, the reaction outcome being dependent on the regioselectivity of the Friedel–Crafts reaction. This presents a rare example of a divergent reaction whereby a regiochemical difference leads to structurally distinct products, so avoiding closely related isomers.^17^

## Results and Discussion

**Scheme 1:**
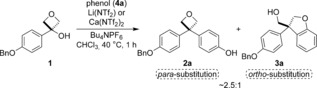
Friedel–Crafts alkylation of oxetane **1** with phenol.

We envisaged that the synthesis of 3,3‐diaryloxetanes might be achieved through a catalytic Friedel–Crafts reaction, directly displacing a hydroxy group from 3‐aryloxetan‐3‐ols. These substrates would be readily generated by the addition of aryl‐metal reagents to commercially available oxetan‐3‐one.11a However, this strategy posed interesting questions on the nature and feasibility of the required carbocationic intermediate due to the increased *p*‐character of the strained ring bonds, and the electron‐withdrawing nature of the oxygen atom. To date there were only two transformations to displace the hydroxy group of *tert*‐oxetanol derivatives, both on aryl derivatives; i) direct OH displacement with diethylaminosulfur trifluoride (DAST) to generate tertiary fluorides and ii) conversion to the tosylate and reduction with LiAlH_4_ to form the 3‐aryl oxetane.11a Furthermore, achieving the desired transformation under catalytic conditions (see Scheme 1), would require the activation of the hydroxyl group selectively, over coordination to the Lewis basic oxetane lone pairs^18^ and other oxygen containing species, and must prevent ring opening polymerization of the oxetanes under acidic conditions.^19^

We selected aryl‐oxetanol **1** for investigation being likely to stabilize a carbocationic intermediate, and with the potential to reveal phenol functionality for further derivatization on benzyl deprotection (Scheme 1). Inspired by recent developments of Friedel–Crafts reactions displacing hydroxy groups at tertiary centers,^20^, ^21^ we examined a variety of Lewis acids as potential catalysts with electron rich aromatic nucleophiles.^22^ We were delighted to observe that both calcium triflimide with Bu_4_NPF_6_ additive, as reported by Niggemann,^21^ and lithium triflimide/Bu_4_NPF_6_ successfully formed the 3,3‐diaryloxetane **2 a** using excess phenol on heating at 40 °C in chloroform. This oxetane product was formed exclusively as the *para*‐substituted phenol derivative, in a ratio of around 2.5:1 with a minor product. Intriguingly, the minor product was identified as 2,3‐dihydrobenzofuran **3 a** resulting from reaction at the *ortho*‐position and oxetane ring opening. The two heterocyclic products **2 a** and **3 a** were readily separated by flash column chromatography to give structurally distinct compounds. This suggested the potential for an unusual substrate controlled structurally divergent reaction based on the phenol regioselectivity.

To investigate and optimize the parameters of the reaction, a design‐of‐experiments (DOE) approach was adopted, and was conducted for both Ca and Li catalysts.^22^ Temperature, concentration, catalyst and additive loading, equivalents of nucleophile and reaction time were investigated (see Table 1 for representative results). Comparable results were obtained with Ca(NTf_2_)_2_ at 5 mol % and Li(NTf_2_) at 10 mol % loadings (entries 1 and 2). On optimization the best results were achieved using the Li(NTf_2_) catalyst (11 mol %) and five equivalents phenol affording a combined yield of 83 % for the two products after 1 h (entry 3). The Bu_4_NPF_6_ additive was needed, presumably to solubilize the catalyst, and a 2:1 ratio of Li(NTf_2_) to Bu_4_NPF_6_ gave the best yield (entries 3–5). Increasing the amount of phenol to 10 equivalents did not result in an improvement. A decrease in the yield of oxetane product was observed on further increasing the catalyst loading to 20 mol % (entry 7). Dichloroethane, chlorobenzene and hexane were also suitable solvents but delivered slightly reduced yields of oxetane (52–58 %). Both Li(NTf_2_) and Bu_4_NPF_6_ are inexpensive and easily handled powders. Increasing the scale of the reaction to 7.5 mmol gave comparable results, providing >1 g of oxetane **2 a** (57 %) along with dihydrobenzofuran **3 a** (21 %, entry 8). Oxetane **2 a** was stored at room temperature in air for >6 months without any noticeable degradation.

**Table 1:** Selected optimization for the reaction of **1** with phenol.

Entry^[a]^	Catalyst	Cat./Bu_4_NPF_6_ [mol %]	Equiv. phenol	Yield **2 a** [%]^[b]^	Yield **3 a** [%]^[b]^
1	Ca(NTf_2_)_2_	5/5	3	44	19
2	Li(NTf_2_)	10/5	3	47	19
**3**	**Li(NTf_2_)**	**11/5.5**	**5**	**63 (61)^[c]^**	**20 (19)^[c]^**
4	Li(NTf_2_)	11/‐	5	0	0
5	Li(NTf_2_)	11/11	5	22	6^[d]^
6	Li(NTf_2_)	11/5.5	10	65	21
7	Li(NTf_2_)	20/10	5	51	20
8^[e]^	Li(NTf_2_)	11/5.5	5	(57)^[c]^	(21)^[c]^

[a] Conditions: **1** (0.25 mmol), CHCl_3_, 0.5 m, 40 °C, 1 h. [b] Yield determined by ^1^H NMR using 1,3,5‐trimethoxybenzene as internal standard. [c] Yield of isolated product in parenthesis. [d] **1** recovered in 46 %. [e] 7.5 mmol **1** used.

**Scheme 2:**
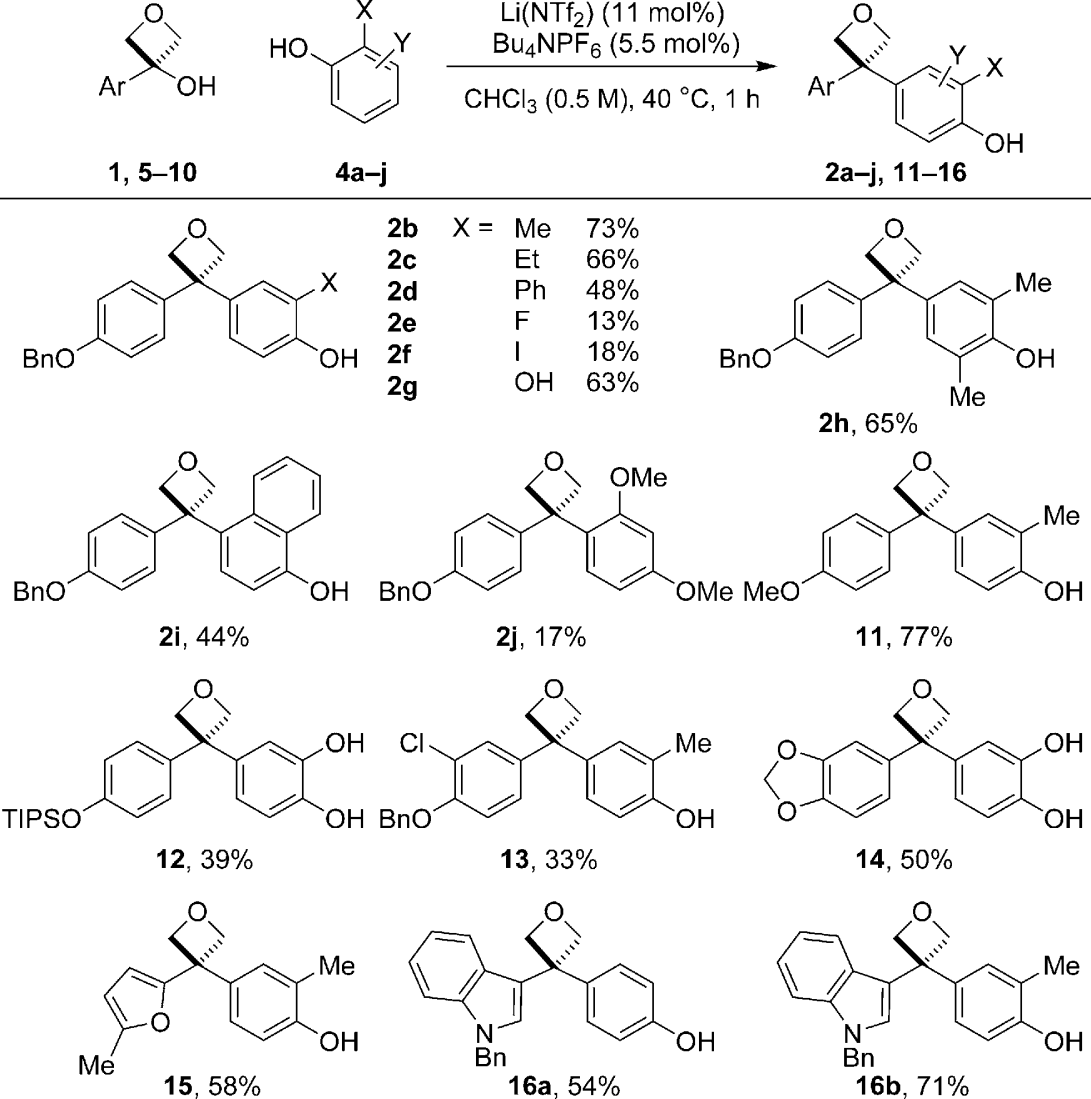
Scope of 3,3‐diaryloxetanes using *ortho*‐substituted phenols.

With optimized conditions in hand, the scope of the reaction to selectively form oxetane products was investigated. Pleasingly, the use of *ortho*‐substituted phenols gave excellent selectivity for the diaryloxetane products, which were formed exclusively (Scheme 2, <3 % dihydrobenzofuran for all examples by ^1^H NMR of the crude reaction). Electron‐rich phenols gave high yields of diaryloxetanes **2 a**–**c**. Electron‐withdrawing and electron‐neutral *ortho*‐substituents were tolerated on phenol (**2 d**–**f, h**), though reduced yields were obtained for the halide examples. This is likely to be due to decomposition of the unstable carbocation with lower rates of addition for these nucleophiles. In all cases the starting material was consumed within the 1 h reaction time. Catechol and 1‐naphthol also proved to be suitable nucleophiles with the diaryloxetanes **2 g** and **2 i** formed good yield (63 and 44 %, respectively). Furthermore, dimethoxybenzene provided an example without phenol functionality (**2 j**), albeit in low yield.

Alternative 3‐aryl‐oxetan‐3‐ol substrates were prepared (**5**–**10**), each with a *para*‐donating aromatic groups (see the Supporting Information). Using either catechol or 2‐methylphenol gave the desired oxetane products in each case (**11**–**14**) in 33–77 % yields. Importantly, heterocycles were also suitable as the preinstalled aromatic group. 2‐Methylfuran was capable of stabilizing the carbocationic intermediate affording oxetane **15** in 58 % yield, providing another functionalizable handle. Furthermore, indole‐containing oxetanes **16 a** and **16 b** were formed in high yields under the same conditions. As such, by using a combination of different nucleophiles and preinstalled aryl groups a variety of 3,3‐diaryl‐oxetanes can be rapidly prepared, with attractive functionality for further elaboration.

The dihydrobenzofuran product **3 a** would exhibit the diaryl motif in an alternative defined orientation and represented an interesting compound in its own right. This scaffold is present in a multitude of natural products and synthetic compounds including marketed drugs with wide ranging biological activity.^23^, ^24^ The intramolecular ring opening of oxetane products to generate other heterocycles has received interest in recent years,^25^ including the formation of certain dihydrobenzofuran derivatives.^26^ Herein little studied 3‐aryl‐3‐hydroxymethyl‐dihydrobenzofurans are prepared in a short sequence,^27^ whereby oxetan‐3‐one functions as a ‘triple electrophile’ over 2 steps.

**Scheme 3:**
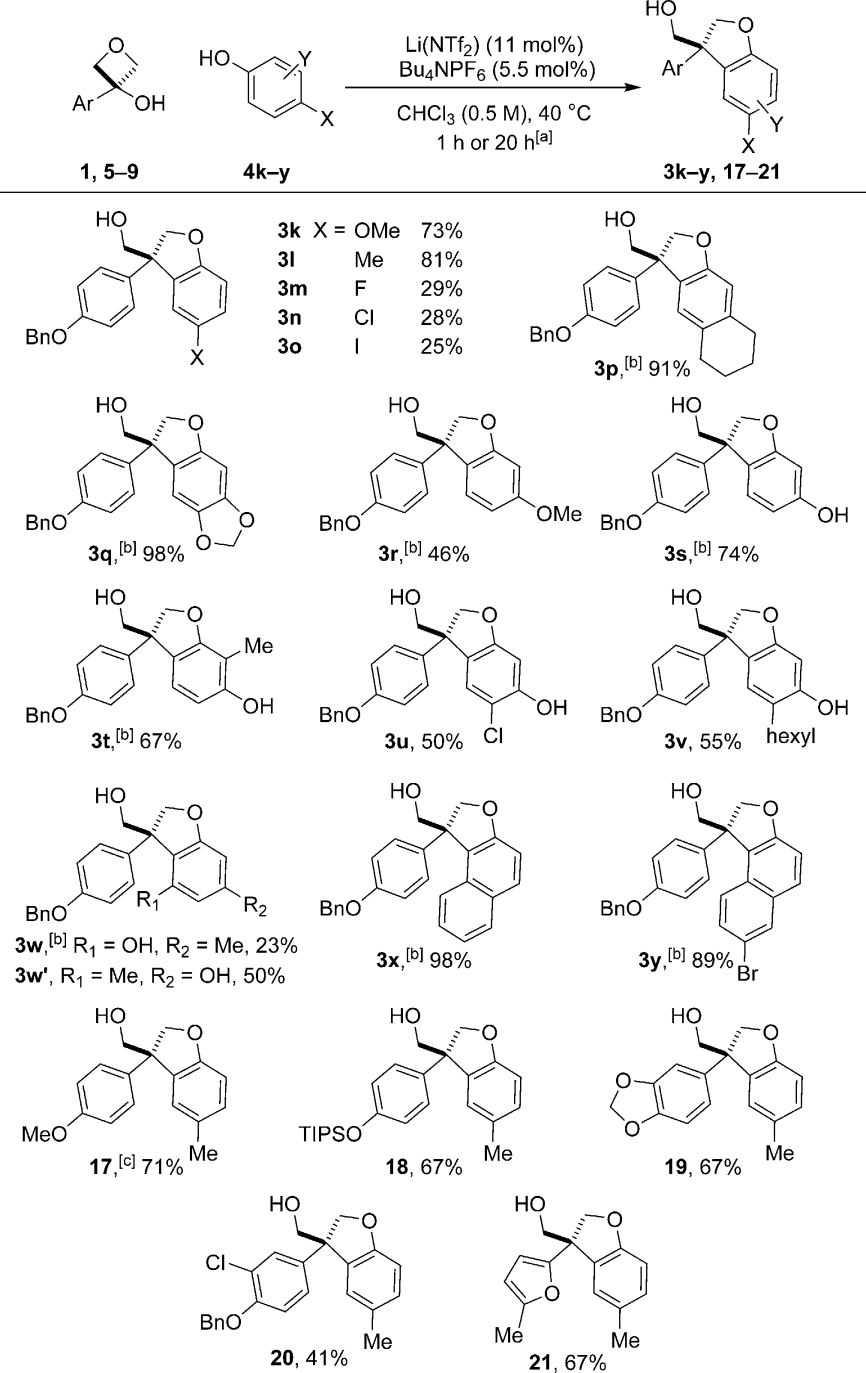
Scope of dihydrobenzofurans using *meta* and *para*‐substituted phenols. [a] Reaction time of 20 h unless stated. [b] Reaction time of 1 h. [c] Reaction time of 6 h.

We anticipated that blocking the phenol *para*‐position would afford these products exclusively, which was proven correct (Scheme 3). Dihydrobenzofurans were prepared using a wide selection of *para*‐ and *meta*‐substituted phenols and related compounds. *para*‐Substituted phenols (**4 k**–**o**) were successful using an extended reaction time of 20 h. As with the oxetane products, more electron donating substituents gave good yields, and lower yields for halide substituted examples. A 98 % yield of sesamol derivative **3 q** was obtained, formed as a single regioisomer. 3‐Methoxyphenol also gave exclusively the dihydrobenzofuran product **3 r**, the steric difference between hydroxy and methoxy proving sufficient to completely prevent addition at the 4‐position of the phenol. Resorcinols proved to be good substrates forming the dihydrobenzofuran products exclusively in yields of 67–74 % (**3 s**–**v**). With 5‐methylresorcinol, a mixture of dihydrobenzofuran regioisomers were obtained in 73 % combined yield (**3 w** and **3 w’**). 2‐Napthol derivatives gave excellent yields of single regioisomers of derivatives **3 x** and **3 y**. Substrates in which the preinstalled aromatic group was varied were successful upon reaction with *p*‐methylphenol to give dihydrobenzofuran derivatives **17**–**21**.

**Scheme 4:**
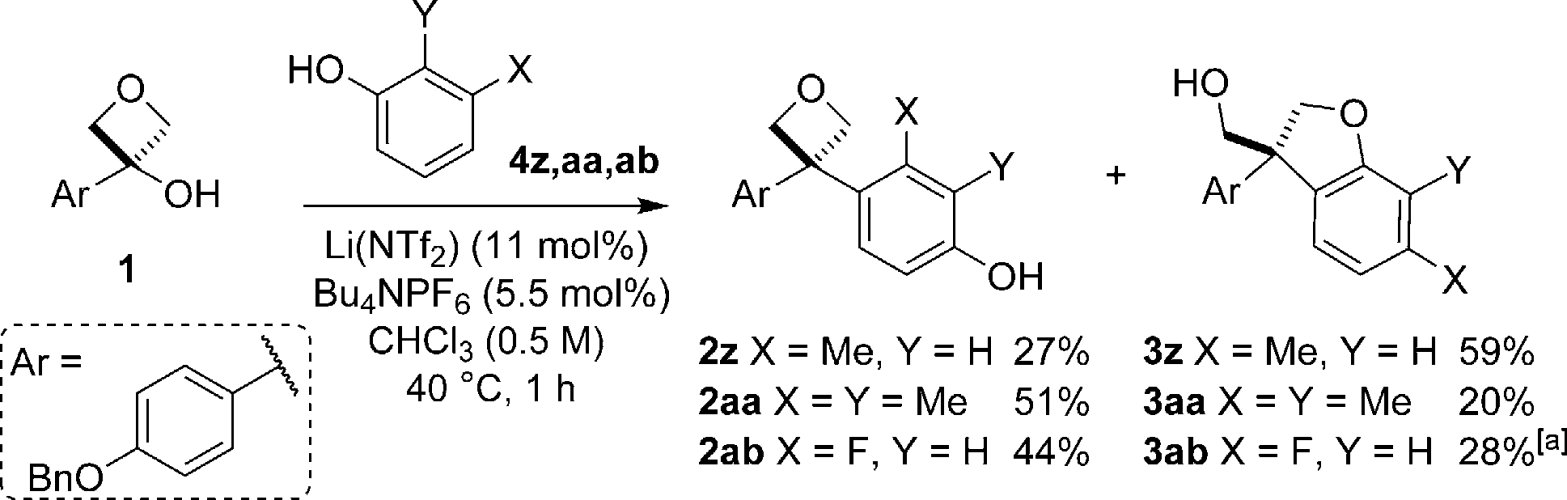
Some *meta*‐substituted phenols gave separable mixtures of oxetane and dihydrobenzofuran products. [a] Formed as a mixture of 6‐F and 4‐F dihydrobenzofurans (85:15).

Only a small selection of *meta*‐substituted phenols gave rise to mixtures of product types, with the product ratio affected by the bulk of the *meta‐*substituent and the presence of an additional *ortho*‐substituent (Scheme 4). Importantly the structural isomers were readily separable in each case. With 3‐methylphenol as the nucleophile, dihydrobenzofuran **3 z** was formed as the major product yield (**2 z**:**3 z**;1:2) in 86 % combined yield. 2,3‐Dimethylphenol led to 3,3‐diaryloxetane **2 aa** as the major product. The reaction with 3‐fluorophenol showed a slight preference for oxetane **2 ab**, and dihydrobenzofuran **3 ab** was formed as a mixture of 6‐F and 4‐F isomers.

**Scheme 5:**
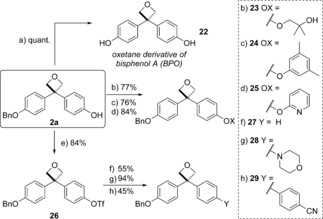
Derivatization of 3,3‐diaryloxetane **2 a**. a) Pd/C (10 % w/w), H_2_ (1 atm), EtOH, 0.1 m, 25 °C, 14 h. b) 1,1‐Dimethyloxirane, K_2_CO_3_, DMF, 0.5 m, 100 °C, 16 h. c) 5‐Iodo‐*m*‐xylene, CuI (5 mol %), *N*,*N*‐dimethylglycine.HCl (18.5 mol %), Cs_2_CO_3_, dioxane, 0.5 m, 90 °C, 20 h. d) 2‐iodopyridine, CuI (5 mol %), picolinic acid (10 mol %), K_3_PO_4_, DMSO, 0.5 m, 90 °C, 23 h. e) Tf_2_O, pyridine, CH_2_Cl_2_, 0.5 m, 25 °C, 2 h. f) Pd(OAc)_2_ (2 mol %), 1,1′‐ferrocenediyl‐bis(diphenylphosphine) (=dppf) (2 mol %), Et_3_N, HCO_2_H, DMF, 0.5 m, 60 °C, 1 h. g) Morpholine, Pd(OAc)_2_ (1 mol %), JohnPhos (2 mol %), K_3_PO_4_, THF, 0.5 m, 65 °C, 24 h. h) 4‐Cyanoboronic acid, Pd(OAc)_2_ (5 mol %), SPhos (10 mol %), K_3_CO_3_, THF, 0.5 m, 65 °C, 18 h.

With a selection of 3,3‐diaryloxetanes available, we demonstrated the removal of the benzyl group to reveal the phenol functionality. Simple hydrogenolysis of **2 a** using Pd/C under H_2_ gave oxetane **22** in quantitative yield (Scheme 5). Oxetane **22** is a novel derivative of BPA, in which the *gem*‐dimethyl is linked with an oxygen atom, which may present interesting properties as a BPA alternative.

To exploit the phenol functionality, the derivatization of oxetane **2 a** was explored through a range of reactions, intended to demonstrate the stability of the oxetane unit. Alkylation by ring opening of 1,1‐dimethyloxirane under basic conditions gave oxetane **23** in 77 % yield. Copper‐catalyzed Ullmann couplings proceeded in good yields with both 1‐iodo‐3,5‐dimethylbenzene and 2‐iodopyridine (**24** and **25**),^28^ indicating good stability of the oxetane over extended reaction times at elevated temperatures. Conversion of oxetane **2 a** to triflate **26** was successful under standard conditions (Tf_2_O, pyridine). From triflate **26**, palladium‐catalyzed deoxygenation afforded phenyl substituted oxetane **27** (X=H).^29^ Buchwald–Hartwig amination gave morpholine substituted oxetane **28** in an excellent 94 % yield.^30^ Finally, Suzuki–Miyaura cross‐coupling using Pd(OAc)_2_ and SPhos gave biaryl **29**.

**Scheme 6:**
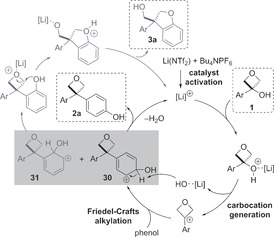
Proposed catalytic cycle.

Mechanistically, we propose a catalytic cycle involving initial activation of the Li(NTf_2_) pre‐catalyst through disruption of the tetrahedrally coordinated solid‐state lattice,^31^ aided by the ammonium salt additive (Scheme 6). Coordination and activation of the *tertiary*‐hydroxy group is likely to generate the key carbocationic intermediate. Addition of phenol through the *para‐* or *ortho‐*position would generate intermediates **30** or **31**, respectively (boxed). From intermediate **30**, rearomatization would yield the 3,3‐diaryloxetane product **2 a**, with a loss of water regenerating the catalyst. Conversely, if *ortho*‐addition into the carbocation occurs, rearomatization and coordination of the Lewis acid catalyst to the oxetane oxygen initiates intramolecular cyclization to dihydrobenzofuran **3 a**. This is rapid and the *ortho*‐substituted diaryloxetane was not observed in any dihydrobenzofuran forming example. On this path, the Li catalyst displays selective sequential activation of the hydroxy group for Friedel–Crafts alkylation and then activation of the oxetane to ring opening.

## Conclusion

We have developed a rapid approach to 3,3‐diaryloxetane and 2,3‐dihydrobenzofuran scaffolds through a Friedel–Crafts reaction on oxetanols with phenols, using an inexpensive and widely available lithium catalyst. The reaction is divergent to give the two heterocyclic classes which can be accessed with complete selectivity dependent on the *ortho*‐ or *para*‐regioselectivity of the phenol substrates. We present successful conditions for the efficient synthesis of 3,3‐diaryloxetanes, the first examples of this motif, designed as an isostere for diarylmethanes and diaryl ketones. High yields were achieved using *ortho*‐substituted phenols and related derivatives, with complete selectivity for the 3,3‐diaryloxetane product observed. Remarkably, the lithium catalyst for the Friedel–Crafts reaction selectively activates the *tertiary*‐hydroxy group of oxetan‐3‐ols without being impeded by coordination to other oxygen containing species present, and without causing ring opening or polymerization of the oxetanes. Furthermore, good reactivity and stability of the products to a number of synthetically useful transformations such as alkylation, deoxygenation and metal‐catalyzed cross‐coupling reactions, was demonstrated, suitable for their use in medicinal chemistry programs. In all cases the oxetane derivatives showed very high stability to the reaction conditions. Under the same conditions, *ortho*‐selective alkylation afforded the synthesis of dihydrobenzofurans, also a biologically important motif, by one‐pot Friedel–Crafts alkylation and oxetane ring opening. The use of *meta*‐ and *para*‐substituted phenols, resourcinols and 2‐napthol derivatives enabled formation of these novel scaffolds in high yields. Both product classes present novel substitution patterns on the O‐heterocycles and a wide scope of derivatives is prepared. The reaction likely proceeds via an unusual carbocationic intermediate on the oxetane ring formed through selective activation of the hydroxyl group of oxetan‐3‐ols. Future studies on the properties of the 3,3‐diaryloxetanes, and application to other ring systems will be reported in due course.

## Supporting information

As a service to our authors and readers, this journal provides supporting information supplied by the authors. Such materials are peer reviewed and may be re‐organized for online delivery, but are not copy‐edited or typeset. Technical support issues arising from supporting information (other than missing files) should be addressed to the authors.

Supplementary
