# Supplementary material for: Structurally Divergent Lithium Catalyzed Friedel–Crafts Reactions on Oxetan‐3‐ols: Synthesis of 3,3‐Diaryloxetanes and 2,3‐Dihydrobenzofurans
Source: Chemistry. 2016 Oct 10;22(45):16271–6. doi: 10.1002/chem.201604031 (PMC5095816; doi:10.1002/chem.201604031)
Supplement: Supplementary file 1 — Supplementary [file CHEM-22-16271-s001.pdf]

CHEMISTRY
A European Journal

Supporting Information

Structurally Divergent Lithium Catalyzed Friedel–Crafts Reactions on Oxetan-3-ols: Synthesis of 3,3-Diaryloxetanes and 2,3-Dihydrobenzofurans

Rosemary A. Croft,[a] James J. Mousseau,[b] Chulho Choi,[b] and James A. Bull*[a]

chem_201604031_sm_miscellaneous_information.pdf

Table of Contents

Table of Contents S1
Structures of Phenols **4a**–**4ab** and Oxetan-3-ols **1**, and **5–10** S2
General Experimental Considerations S3
Reaction Optimization S4
- DOE Optimization with $Ca(NTf_2)_2$ Catalyst S5
- DOE Optimization with $Li(NTf_2)$ Catalyst S8
- Further Optimization with $Li(NTf_2)$ Catalyst S11

Synthesis of **1**, **2a** and **3a** S12
Synthesis of Friedel-Crafts Cyclization Precursors **5**–**10** S14
Synthesis of Diaryloxetanes **2a**–**j, 11**–**16** S17
Synthesis of Dihydrobenzofurans **3k**–**y**, **17**–**21** S23
Synthesis of Diaryloxetanes **2z**–**2ab** and Dihydrobenzofurans **3z**–**3ab** S32
Functionalization of Oxetane **2a** S35
References S38
^{1}H and ^{13}C NMR Spectra for Selected Compounds S39

The raw NMR data and also Mestrenova files for novel compounds can be accessed at the following link: https://imperialcollegelondon.box.com/v/diaryloxetanes

Structures of Phenols 4a–4ab and Oxetan-3-ols 1, and 5–10

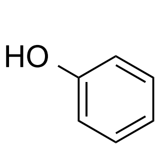
4a
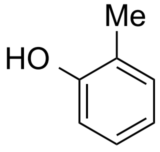
4b
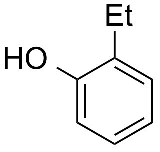
4c
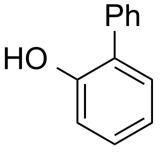
4d
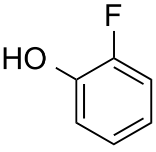
4e
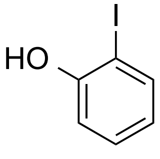
4f
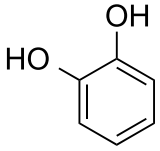
4g
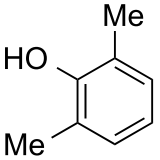
4h
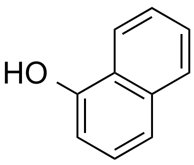
4i
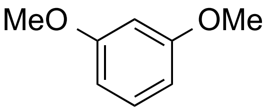
4j
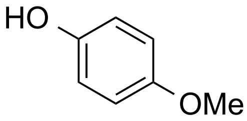
4k
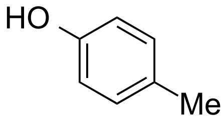
4l
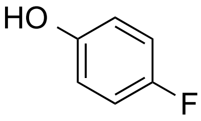
4m
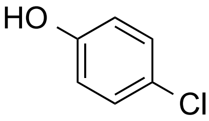
4n
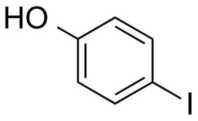
4o
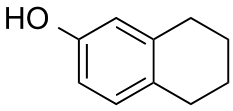
4p
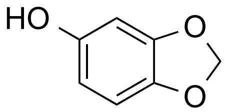
4q
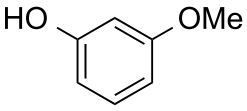
4r
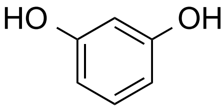
4s
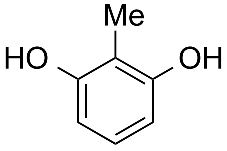
4t
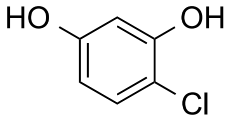
4u
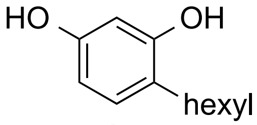
4v
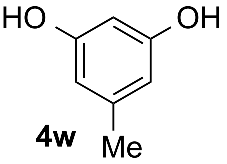
4w
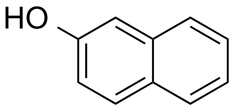
4x
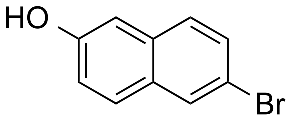
4y
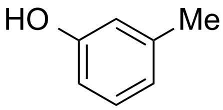
4z
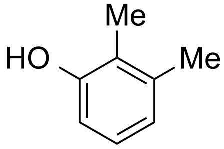
4aa
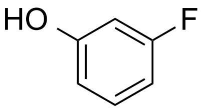
4ab

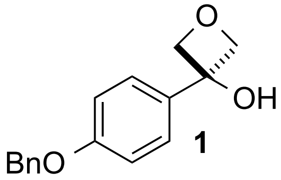
1
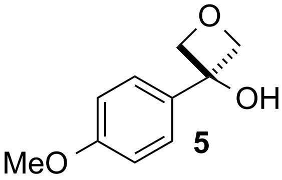
5
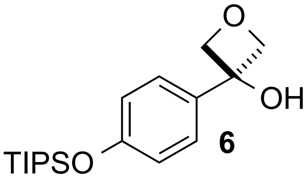
6
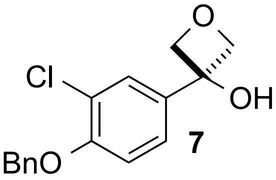
7
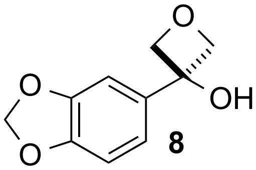
8
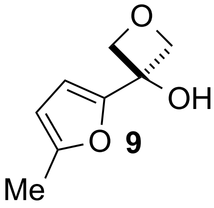
9
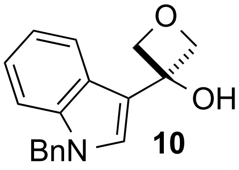
10

General Experimental Considerations

All nonaqueous reactions were run under an inert atmosphere (argon) with flame-dried glassware using standard techniques. Anhydrous solvents were obtained by filtration through drying columns (CH_3CN, THF, CH_2Cl_2) or used directly from commercial sources (1,4-dioxane, DMF, EtOH, $CHCl_3$). Flash column chromatography was performed using 230-400 mesh silica with the indicated solvent system according to standard techniques. Analytical thin-layer chromatography (TLC) was performed on precoated, glass-backed silica gel plates. Visualization of the developed chromatogram was performed by UV absorbance (254 nm), aqueous potassium permanganate stain or PMA (phosphomolybdic acid) or vanillin. Infrared spectra (ν_{max}, FTIR ATR) were recorded in reciprocal centimeters (cm^{-1}). Nuclear magnetic resonance spectra were recorded on 400 MHz spectrometers. Chemical shifts for ^1H NMR spectra are recorded in parts per million from tetramethylsilane with the solvent resonance as the internal standard ($CDCl_3$, δ = 7.27 ppm, $(CD_3)_2SO$: 2.50 ppm, CD_3OD: 3.31). Data is reported as follows: chemical shift [multiplicity (s = singlet, d = doublet, t = triplet, quin = quintet, sep = septet, m = multiplet and br = broad), coupling constant in Hz, integration, assignment]. ^{13}C NMR spectra were recorded with complete proton decoupling. Chemical shifts are reported in parts per million from tetramethylsilane with the solvent resonance as the internal standard ($^{13}CDCl_3$: 77.0 ppm, $(^{13}CD_3)_2SO$: 39.5 ppm, $^{13}CD_3OD$: 49.0). ^{19}F NMR spectra were recorded with complete proton decoupling. Chemical shifts are reported in parts per million referenced to the standard monofluorobenzene: −113.5 ppm. Chemical shifts are reported in parts per million. *J* values are reported in Hz. Assignments of $^1H/^{13}C$ spectra were made by the analysis of δ/J values, and COSY, HSQC, and HMBC experiments as appropriate. Melting points were obtained using Optimelt MPA100 melting point apparatus and are uncorrected.

Reagents: Commercial reagents were used as supplied or purified by standard techniques where necessary (pyridine, triethylamine, morpholine).

Notes:

- *For several of the dihydrobenzofurans the O–H peak in the ^1H NMR was obscured by the water peak but confirmed by COSY NMR techniques.*
- *When assigning the dihydrobenzofurans, aromatic peaks in the ^1H and ^{13}C NMR spectra related to the dihydrobenzofuran structure are denoted with DHBF.*

Reaction Optimization

Table S1 Initial catalyst screen: Selected examples

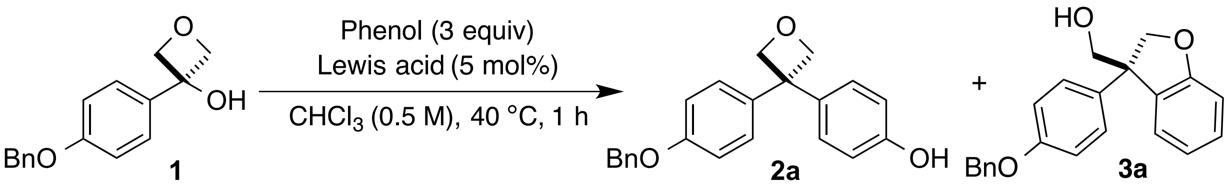

Entry	Loading of Lewis acid catalyst (mol%)	Oxetane product 2a (%)[a]	Dihydrobenzofuran product 3a (%)[a]	SM 1 (%)[a]
1	$Fe(OTf)_3$	23	10	0
2	$Yb(OTf)_3$	0	0	100
3	$FeCl_3$	0	0	98
4	$Ca(NTf_2)_2$ + Bu_4NPF_6	42	20	0
5	$Ca(BF_4)_2$ + Bu_4NPF_6	14	7	45
6	$Ca(OTf)_2$ + Bu_4NPF_6	0	0	85
7	$Li(NTf_2)$ (10 mol%)+ Bu_4NPF_6	47	19	0

[a]Yield calculated by ^{1}H NMR using 1,3,5-trimethoxybenzene as an internal standard.

A number of catalysts were screened for the reaction between oxetanol **1** and electron rich aromatics (selected examples in Table S1). $Ca(NTf_2)_2$ or $Li(NTf_2)$ in combination with Bu_4NPF_6 and gave good combined yields of oxetane **2a** and dihydrobenzofuran **3a** (62% and 66% respectively). Several additional catalysts were also examined with 1,3-dimethoxybenzene with other oxetanol derivatives eg *p*-methoxyphenyl derivative (**5**), shown to have similar reactivity to the OBn derivative. $Ga(OTf)_3$, $Bi(OTf)_3$, $Sc(OTf)_3$, $Hf(OTf)_3$, $Fe(OTf)_2$ all resulted in no product being formed. $Ca(NTf_2)_2$ gave an 18% yield of diaryloxetane product by ^{1}H NMR at 40 ºC. Lower temperatures (25 ºC, 30 ºC and 35 ºC) gave no product and full recovery of starting material. Higher temperatures (up to 55 ºC) gave no improvement in yield. Early studies on reaction concentration did not give improved yields of oxetane (18–20%), but lower concentrations showed an increase in recovered starting material (34% at 0.5 M to 63% at 0.1 M).

Design of Experiments (DOE) Optimization with $Ca(NTf_2)_2$ catalyst

A DOE approach using Modde pro 11 software (MKS Umetrics) was used to analyse the reaction space in an efficient manner. The advantage of using a DOE approach was that by changing multiple factors simultaneously, relationships between these factors and the effect on yield could be investigated fully. Five continuous factors considered significant for the reaction were investigated: temperature, concentration, catalyst loading, equivalents of nucleophile and time (Table S2). The upper and lower limits were chosen to keep the reaction conditions mild whilst allowing a reasonable variation in reaction space.

Table S2: Parameters for DOE optimisation with $Ca(NTf_2)_2/Bu_4NPF_6$

Parameter	Units	Lower limit	Upper limit
Temperature	°C	40	55
Concentration	M	0.05	0.5
Catalyst loading	mol%	1	9
Equivs of nucleophile	Equivs	1	5
Time	mins	30	240

A central composite faced (CCF) design was used. This took maximum, minimum and mid-point values for each factor and generated a list of 29 combinations of these values to effectively cover reaction space (Table S3, entries 1–29). Three repeats using the midpoint values for all factors were conducted to confirm reproducibility (Entries 27–29).

Table S3.

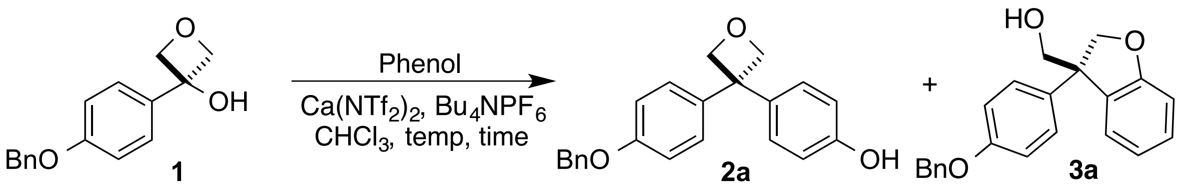

Entry	Temp/ °C	Conc/ M	Loading of $Ca(NTf_2)_2$ /mol%	Equivs of nucl	Time	Yield oxetane 2a (%)[a]	Yield of dihydrobenzofuran 3a (%)[a]	Recovery of 1 (%)[a]
1	40	0.05	1	1	240	0	0	85
2	55	0.05	1	1	30	0	0	83
3	40	0.5	1	1	30	0	0	78
4	55	0.5	1	1	240	0	0	62
5	40	0.05	9	1	30	0	0	29
6	55	0.05	9	1	240	29	24	53
7	40	0.5	9	1	240	21	12	33
8	55	0.5	9	1	30	22	23	45
9	40	0.05	1	5	30	12	7	52
10	55	0.05	1	5	240	0	0	50
11	40	0.5	1	5	240	42	12	54
12	55	0.5	1	5	30	0	0	93
13	40	0.05	9	5	240	36	24	60
14	55	0.05	9	5	30	54	27	81
15	40	0.5	9	5	30	49	22	71
16	55	0.5	9	5	240	0	3	3
17	40	0.275	5	3	135	47	19	66
18	55	0.275	5	3	135	31	26	57
19	47.5	0.05	5	3	135	48	25	73
20	47.5	0.5	5	3	135	31	14	45
21	47.5	0.275	1	3	135	0	0	14
22	47.5	0.275	9	3	135	9	11	20
23	47.5	0.275	5	1	135	26	12	38
24	47.5	0.275	5	5	135	40	22	62
25	47.5	0.275	5	3	30	31	13	44
26	47.5	0.275	5	3	240	29	21	50
27	47.5	0.275	5	3	135	33	21	54
28	47.5	0.275	5	3	135	33	20	53
29	47.5	0.275	5	3	135	33	20	53
30	40	0.5	5.5	5	60	59	22	0

[a]Yield calculated by ^{1}H NMR using 1,3,5-trimethoxybenzene as an internal standard.

Summary of Fit:

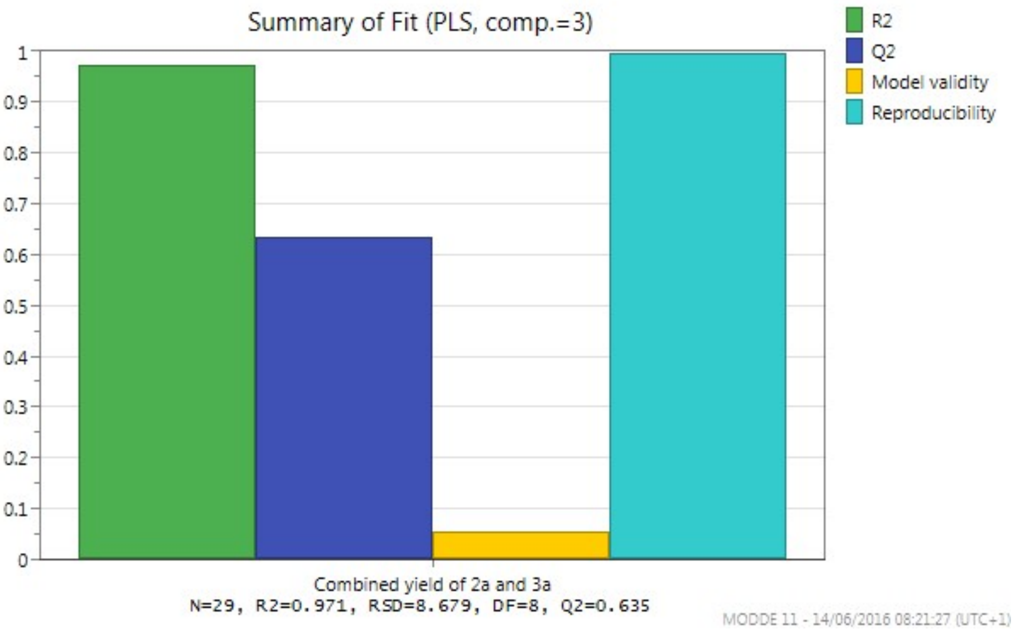

R2 shows the model fit (>0.5 for a significant model); Q2 gives an estimate for future prediction precision (>0.5 for a good model); model validity can be very low even for good and complete models when the replicates are almost identical; Reproducibility (>0.5 for good models).[1]

Observed vs predicted outcomes based on PLS model:

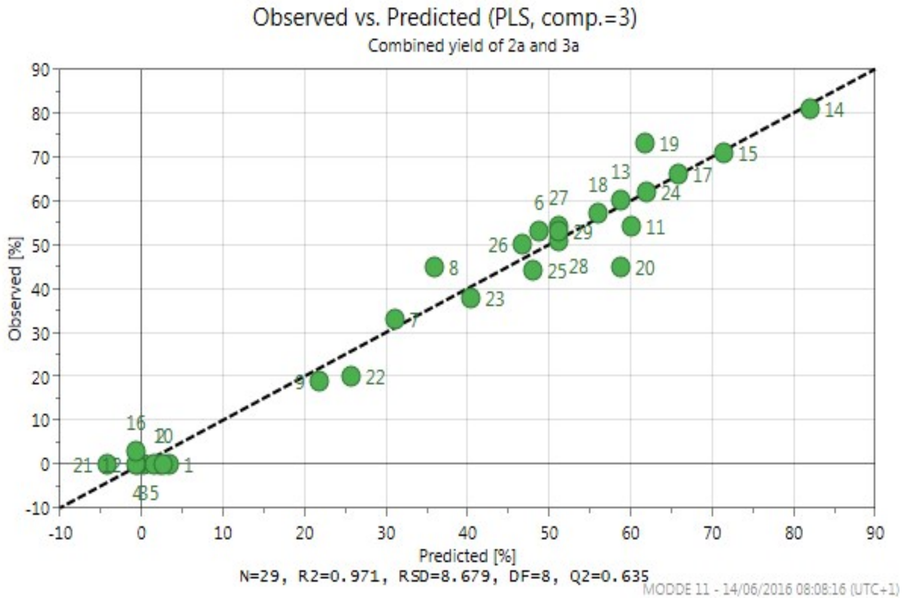

Analysis of the data using using a partial least squares (PLS) modelling method allowed the generation of a reaction profile. Use of this model determined equivalents of nucleophile and catalyst loading to be the most important considerations in terms of yield whereas time (within the confines examined) was considered essentially irrelevant. Additionally, an interesting relationship between concentration and temperature was observed; at high temperatures, lower concentrations were favourable whereas at lower temperatures, higher concentrations maximized yields.

Use of the optimization function, with the aim of maximizing the yield of product, gave the conditions shown in entry 30. Indeed, these conditions gave the highest yield reported (59% of oxetane **2a** and 22% dihydrobenzofuran **3a** by ^{1}H NMR).

DOE Optimization with $Li(NTf_2)$ catalyst

Similarly to above, five continuous factors were investigated (Table S4). Due to the finding that an extended reaction time did not improve yields, the reaction time was set at 60 mins. However, for the $Li(NTf_2)$ catalyst different combinations of catalyst and additive loading were investigated.

Table S4. Parameters for DOE optimisation with $Li(NTf_2)/Bu_4NPF_6$

Parameter	Units	Lower limit	Upper limit
Catalyst loading (mol %)	mol%	5	15
Additive loading (mol %)	mol%	2.5	7.5
Equivalents of nucleophile	Equivs	2	5
Concentration	M	0.05	1
Temperature (°C)	°C	40	60

As before, a CCF factorial design was used taking maximum, minimum and mid-point values for each factor in order to generate a list of 29 reactions covering the reaction space (Table S5).

Table S5.

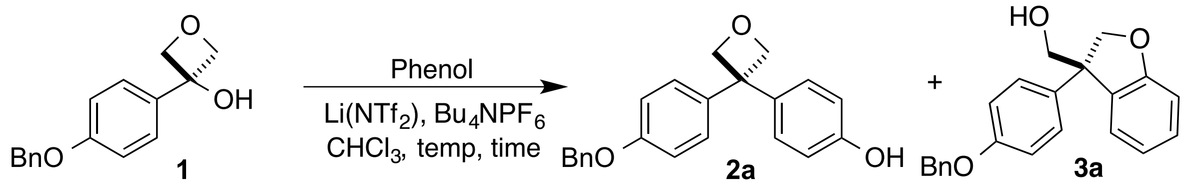

Entry	Temp/ °C	Loading of $Li(NTf_2)$ /mol%	Loading of Bu_4NPF_6 /mol%	Conc/ M	Equivs of nucl	Yield of oxetane 2a (%)[a]	Yield of dihydrobenzofuran 3a (%)[a]	Recovery of material 1 (%)[a]
1	40	5	2.5	0.05	5	20	7	66
2	60	5	2.5	0.05	2	41	21	62
3	40	15	2.5	0.05	2	45	23	68
4	60	15	2.5	0.05	5	49	32	81
5	40	5	7.5	0.05	2	0	0	100
6	60	5	7.5	0.05	5	0	0	82
7	40	15	7.5	0.05	5	53	29	82
8	60	15	7.5	0.05	2	35	19	54
9	40	5	2.5	1	2	0	0	90
10	60	5	2.5	1	5	56	23	79
11	40	15	2.5	1	5	0	0	99
12	60	15	2.5	1	2	0	0	0
13	40	5	7.5	1	5	0	6	33
14	60	5	7.5	1	2	0	8	20
15	40	15	7.5	1	2	35	15	50
16	60	15	7.5	1	5	3	12	15
17	40	10	5	0.525	3.5	57	18	75
18	60	10	5	0.525	3.5	0	0	0
19	50	5	5	0.525	3.5	6	2	17
20	50	15	5	0.525	3.5	37	23	60
21	50	10	2.5	0.525	3.5	53	23	76
22	50	10	7.5	0.525	3.5	53	22	75
23	50	10	5	0.05	3.5	44	28	72
24	50	10	5	1	3.5	41	22	63
25	50	10	5	0.525	2	39	21	60
26	50	10	5	0.525	5	51	23	74
27	50	10	5	0.525	3.5	45	18	63
28	50	10	5	0.525	3.5	42	24	66
29	50	10	5	0.525	3.5	41	21	62

[a]Yield calculated by ^1H NMR using 1,3,5-trimethoxybenzene as an internal standard.

Summary of Fit:

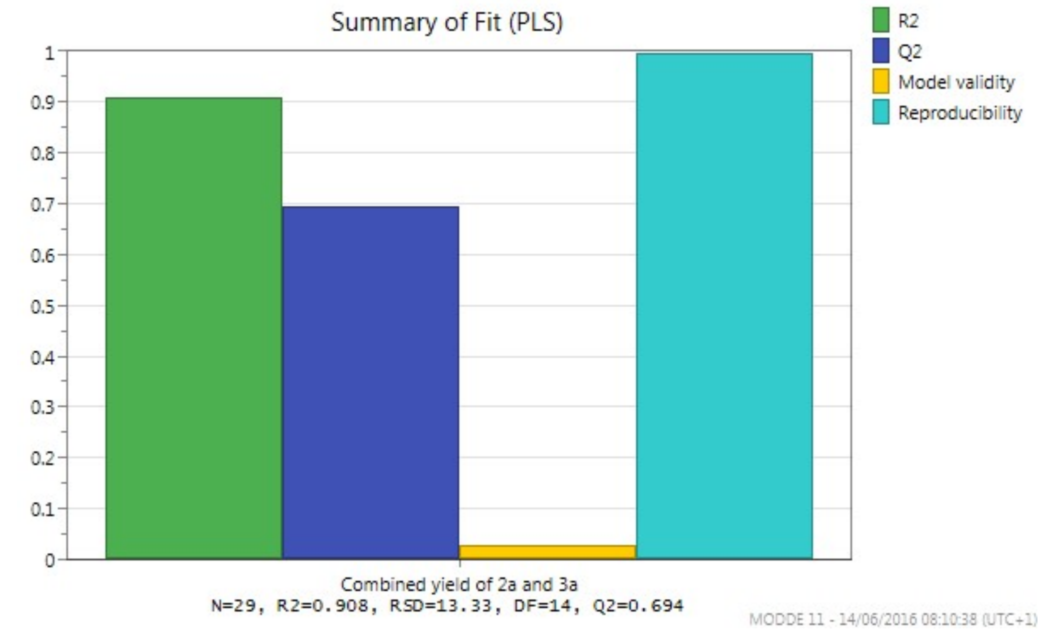

R2 shows the model fit (>0.5 for a significant model); Q2 gives an estimate for future prediction precision (>0.5 for a good model); model validity can be very low even for good and complete models when the replicates are almost identical; Reproducibility (>0.5 for good models).[1]

Observed vs predicted outcomes based on PLS model:

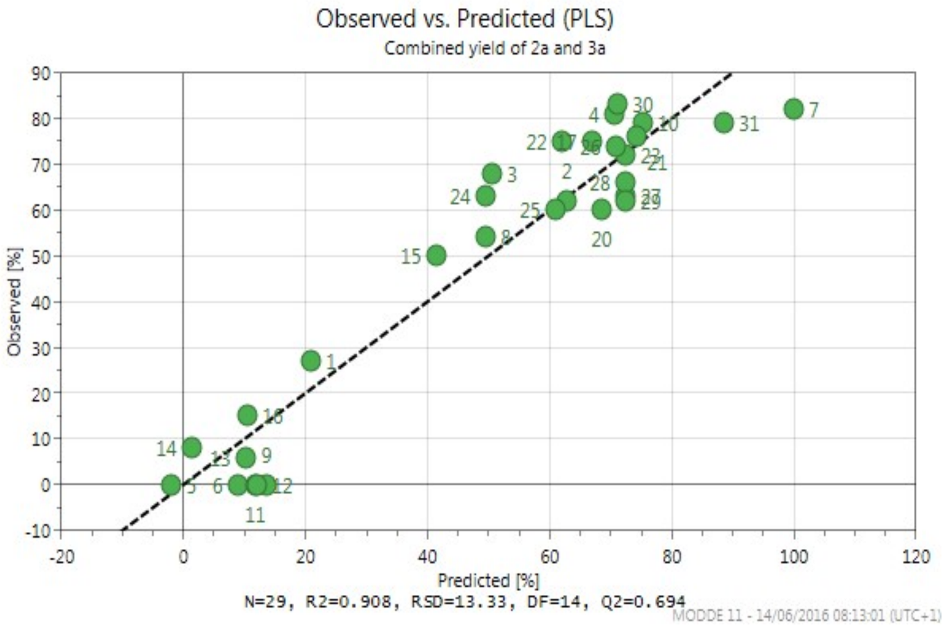

A PLS modelling method was used and the resultant model revealed a catalyst to additive ratio <1:1 was detrimental to the success of the reaction. However, a ratio of 2:1 or greater was sufficient to give maximum yields. Following statistical analysis of this data, the optimiser tool was used, but this model gave no increase in yield compared to those previously observed.

Applying the learnings from the two DOE studies, gave a set of conditions that gave the highest yield to date (83% combined yield) using the Li catalyst [Li(NTf_2) (11 mol%), Bu_4NPF_6 (5.5), 5 equiv phenol, $CHCl_3$, 0.5 M, 40 ºC]. Under these conditions, using the Li catalyst, higher yields for oxetane **2a** were reproducibily observed (63% oxetane **2a** and 20% dihydrobenzofuran **3a** by ^{1}H NMR), along with marginally better selectivity (**2a**:**3a**).

Further Optimization with Li(NTf_2) catalyst

Final variation of the reaction conditions confirmed the optimal conditions (Table S6, entry 1)

Table S6

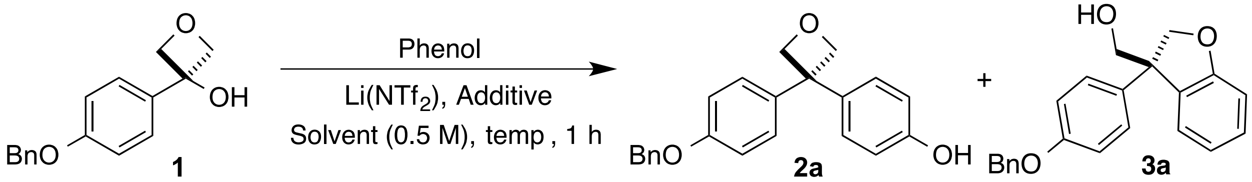

Entry	Additive (mol%)	Ratio of catalyst to additive	Equiv of nucl	Solvent	Temp (°C)	Yield of 2a (%)[a]	Yield of 3a (%)[a]	SM 1 (%)[a]
1	Bu_4NPF_6 (5.5)	2:1	5	$CHCl_3$	40	63	20	0
2	Bu_4NPF_6 (5)	1:1	5	$CHCl_3$	40	11	4	32
3	Bu_4NPF_6 (11)	1:1	5	$CHCl_3$	40	22	6	46
4	Bu_4NPF_6 (7.5)	2:1	5	$CHCl_3$	40	59	20	0
5	Bu_4NPF_6 (10)	2:1	5	$CHCl_3$	40	51	20	0
6	Bu_4NPF_6 (5)	2:1	5	$CHCl_3$	25	0	0	86
7	$AgPF_6$ (5.5)	2:1	5	$CHCl_3$	40	63	20	0
8	Bu_4NPF_6 (5.5)	2:1	10	$CHCl_3$	40	65	21	0
9	Bu_4NPF_6 (5.5)	2:1	5	dichloroethane	40	56	19	0
10	Bu_4NPF_6 (5.5)	2:1	5	hexane	40	58	16	0
11	Bu_4NPF_6 (5.5)	2:1	5	chlorobenzene	40	52	13	0
12	Bu_4NPF_6 (5.5)	2:1	5	toluene	40	0	0	87

[a]Yield calculated by ^1H NMR using 1,3,5-trimethoxybenzene as an internal standard.

A 1:1 ratio of catalyst:additive at both a 5 mol% and 10 mol% loading was confirmed to be detrimental to the yield of the reaction (Table S6, entries 2 and 3) compared to a 2:1 ratio (entry 1). Higher catalyst loadings of 15 or 20% led to a reduction in yield (entries 4 and 5). A temperature of 25 ºC compared to the standard 40 ºC afforded to no product (entry 6). A different hexafluorophosphate additive ($AgPF_6$) gave no difference in yield. Increasing the equivalents of nucleophile from 5 to 10 led to comparable yields of products, which was considered to not offer an advantage. Dichloroethane, hexane and chlorobenzene were found to be suitable solvents, however, decreases in yield of 5–11% led to chloroform being maintained as the solvent of choice (entries 9–11). Use of toluene as the solvent did not results in a productive reaction.

Considering the prices of the Li/Ca salts (US$36/ 5 g for Li($NTf_2$) and US$122/ 1 g for $Ca(NTf_2)_2$, prices quoted from www.sigmaaldrich.com, accessed June 2016), as well as the improved performance of the Li catalyst, the Li reagent was used to explore the reaction scope as described in the manuscript. However, use of the Ca reagent at [$Ca(NTf_2)_2$ (5.5 mol%) and Bu_4NPF_6 (5.5 mol%)] also provides viable conditions.

The conditions in entry 1 were adopted as optimal.

Synthesis of 1, 2a and 3a

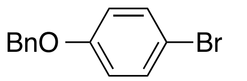

1-(Benzyloxy)-4-bromobenzene

Potassium carbonate (3.87 g, 28.0 mmol) was added to a solution of 4-bromophenol (3.51 g, 20.0 mmol) in acetonitrile (50 mL) at rt. Benzyl bromide (3.42 g, 20.0 mmol) was added dropwise. The reaction mixture was stirred at rt for 6 h then filtered through celite. The filtrate was concentrated *in vacuo* to afford 1-(benzyloxy)-4-bromobenzene (5.09 g, 97%) as a white solid which was used without further purification. R_f = 0.15 (30% EtOAc/hexane); mp = 60–62 °C [Lit = 61 °C][2]; IR (film)/cm^{-1} 3089, 3062, 3033, 2889, 2953, 1884, 1587, 1576, 1486, 1451, 1378, 1289, 1243, 1170, 1103, 1070, 1041, 1026, 999, 971, 932, 905, 849, 823, 813, 731, 693, 656; ^{1}H NMR (400 MHz, $CDCl_3$) δ 7.48–7.31 (m, 7 H, 7 × Ar-CH), 6.90–6.84 (m, 2 H, 2 × Ar-CH), 5.05 (s, 2 H, CH_2); ^{13}C NMR (101 MHz, $CDCl_3$) δ 157.8 (Ar-C_q-O), 136.5 (Ph-C_q), 132.5 (2 × Ar-CH), 128.6 (2 × Ar-CH), 128.1 (Ar-CH), 127.4 (2 × Ar-CH), 116.7 (2 × Ar-CH), 113.1 (Ar-C_q-Br), 70.2 (CH_2). The observed spectroscopic data for this compound was consistent with that previously reported.[3]

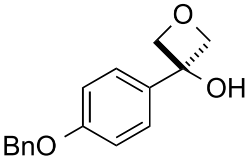

3-[4-(Benzyloxy)phenyl]oxetan-3-ol (1)

n-BuLi (2.24 M in THF, 2.90 mL, 6.50 mmol) was added dropwise over 5 min to a solution of 1-(benzyloxy)-4-bromobenzene (1.71 g, 6.50 mmol) in THF (20 mL) at –78 °C. The reaction mixture was stirred at –78 °C for a further 30 min. Oxetan-3-one (0.32 mL, 5.00 mmol) was added dropwise to the reaction mixture. Following a further 45 min at –78 °C the reaction mixture was warmed to rt then quenched with water (20 mL). The layers were separated and the aqueous portion extracted with diethylether (3 × 30 mL). The organic extracts were combined, washed with brine, dried over Na_2SO_4, filtered and concentrated *in vacuo*. Purification by flash column chromatography (40% EtOAc/hexane) afforded oxetanol **1** (902 mg, 70%) as a white solid. R_f = 0.24 (40% EtOAc/hexane); mp = 114–115 °C; IR (film)/cm^{-1} 3394 (br. OH), 2974, 2936, 2877, 1609, 1580, 1517, 1458, 1399, 1380, 1318, 1239, 1226, 1179, 1126, 1043, 1008, 958, 892, 843, 822, 756, 733, 701, 656; ^{1}H NMR (400 MHz, $CDCl_3$) δ 7.59–7.29 (m, 7 H, 7 × Ar-CH), 7.06–6.99 (m, 2 H, 2 × Ar-CH), 5.12 (s, 2 H, Ph-CH_2), 4.94 (d, *J* = 6.9 Hz, 2 H, C*H*HOC*H*H), 4.92 (d, *J* = 6.9 Hz, 2 H, CH*H*OCH*H*), 2.44 (s, 1 H, OH); ^{13}C NMR (101 MHz, $CDCl_3$) δ 158.4 (Ar-C_q-O), 136.8 (Ph-C_q), 134.7 (Ar-C_q-C_q), 128.6 (2 × Ar-CH), 128.0 (Ar-CH), 127.4 (2 × Ar-CH), 125.9 (2 × Ar-CH), 115.0 (2 × Ar-CH), 85.5 (CH_2OCH_2), 75.7 (C_q), 70.1 ($PhCH_2$); HRMS (ESI-TOF) *m/z* calcd for $C_{16}H_{15}O_2^+$ $[M-OH]^+$: 239.1072, Found: 239.1068. The observed spectroscopic data for this compound was consistent with that previously reported.[4]

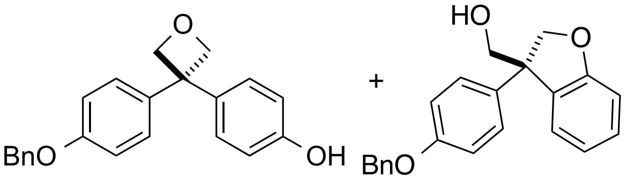

4-{3-[4-(Benzyloxy)phenyl]oxetan-3-yl}phenol, 2a and **{3-[4-(Benzyloxy)phenyl]-2,3-dihydro-1-benzofuran-3-yl}methanol (3a)**

Lithium bis(trifluoromethanesulfonimide) (7.9 mg, 0.0275 mmol) and tetrabutylammonium hexafluorophosphate (5.3 mg, 0.0138 mmol) were added to a solution of oxetanol **1** (64 mg, 0.25 mmol) and phenol (117 mg, 1.25 mmol) in chloroform (0.5 mL). The reaction mixture was stirred at 40 °C for 1 h then quenched with sat. aq. $NaHCO_3$. Dichloromethane (3 × 5 mL) was added and the layers separated. The organic extracts were combined, dried over Na_2SO_4, filtered and concentrated *in vacuo*. Purification by flash column chromatography (30% EtOAc/hexane) afforded dihydrobenzofuran **3a** (16 mg, 19%) as a colorless gum followed by oxetane **2a** (50 mg, 60%) as a white solid.

Dihydrobenzofuran **3a**: R_f = 0.30 (25% EtOAc/hexane); IR (film)/cm^{-1} 3462 (br. OH), 2882, 1609, 1511, 1481, 1458, 1382, 1239, 1184, 1123, 1017, 973, 827, 753, 698; ^{1}H NMR (400 MHz, $CDCl_3$) δ 7.48–7.30 (m, 5 H, 5 × Ar-CH), 7.30–7.17 (m, 4 H, 4 × Ar-CH), 7.00–6.84 (m, 4 H, 4 × Ar-CH), 5.06 (s, 2 H, OCH_2Ph), 4.77 (d, *J* = 8.9 Hz, 1 H, OC*H*HC_q), 4.58 (d, *J* = 8.9 Hz, 1 H, OCH*H*C_q), 4.12 (d, *J* = 11.1 Hz, 1 H, C*H*HOH), 4.04 (d, *J* = 11.1 Hz, 1 H, CH*H*OH); ^{13}C NMR (101 MHz, $CDCl_3$) δ 160.8 ($Ar_{(DHBF)}$-C_q-OCH_2), 157.8 (Ar-C_q-OBn), 136.9 (Ph-C_q), 134.9 (Ar-C_q-C_q), 130.1 ($Ar_{(DHBF)}$-C_q-C_q), 129.3 (Ar-CH), 128.6 (2 × Ar-CH), 128.1 (2 × Ar-CH), 128.0

(Ar-CH), 127.5 (2 × Ar-CH), 125.2 (Ar-CH), 120.8 (Ar-CH), 115.1 (2 × Ar-CH), 110.2 (Ar-CH), 81.3 (O*C*H_2C_q), 70.0 ($PhCH_2$), 67.7 (CH_2OH), 55.8 (C_q); HRMS (CI^+) *m/z* calcd for $C_{22}H_{24}NO_3^+$ $[M+NH_4]^+$: 350.1756, Found: 350.1767.

Oxetane **2a**: R_f = 0.23 (25% EtOAc/hexane); mp = 164–166 °C; IR (film)/cm^{-1} 3325 (br. OH), 2881, 2045, 1608, 1591, 1511, 1455, 1433, 1384, 1269, 1228, 1207, 1176, 1111, 1015, 973, 930, 836, 806, 741, 696; ^1H NMR (400 MHz, $CDCl_3$) δ 7.49–7.30 (m, 5 H, 5 × Ar-CH), 7.17–7.11 (m, 2 H, 2 × Ar-CH), 7.11–7.05 (m, 2 H, 2 × Ar-CH), 7.01–6.94 (m, 2 H, 2 × $Ar_{(phenol)}$-CH), 6.85–6.78 (m, 2 H, 2 × $Ar_{(phenol)}$-CH), 5.21 (s, 4 H, CH_2OCH_2), 5.15 (s, 1 H, OH), 5.07 (s, 2 H, CH_2Ph); ^{13}C NMR (101 MHz, $CDCl_3$) δ 157.4 (Ar-C_q-OBn), 154.3 (Ar-C_q-OH), 138.4 ($Ar_{(phenol)}$-C_q-C_q), 138.2 ($Ar_{(p\text{-}OBn)}$-C_q-C_q), 136.9 (Ph-C_q), 128.6 (2 × Ar-CH), 128.0 (Ar-CH), 127.8 (2 × Ar-CH), 127.6 (2 × Ar-CH), 127.5 (2 × Ar-CH), 115.3 (2 × $Ar_{(phenol)}$-CH), 114.8 (2 × $Ar_{(phenol)}$-CH), 85.0 (CH_2OCH_2), 70.1 (CH_2Ph), 50.3 (C_q); HRMS (CI^+) *m/z* calcd for $C_{22}H_{24}NO_3^+$ $[M+NH_4]^+$: 350.1756, Found: 350.1772.

Reaction was also conducted on a 7.50 mmol scale (1.92 g of oxetanol **1**). Purification by flash column chromatography (3% EtOAc/CH_2Cl_2) afforded dihydrobenzofuran **3a** (524 mg, 21%) and oxetane **2a** (1.41 g, 57%).

Synthesis of Friedel-Crafts Cyclization Precursors 5–10

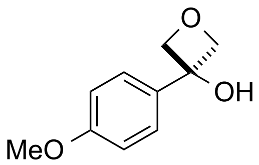

3-(4-Methoxyphenyl)oxetan-3-ol (5)

n-BuLi (2.24 M in THF, 5.80 mL, 13.0 mmol) was added dropwise over 5 min to a solution of 4-bromoanisole (1.63 mL, 13.0 mmol) in THF (45 mL) at –78 °C. The reaction mixture was stirred at –78 °C for a further 10 min. Oxetan-3-one (0.65 mL, 10.0 mmol) in THF (5 mL) was added dropwise to the reaction mixture. Following a further 10 min at –78 °C the reaction mixture was warmed to rt then quenched with water (40 mL). The layers were separated and the aqueous portion extracted with diethylether (3 × 30 mL). The organic extracts were combined, washed with brine, dried over Na_2SO_4, filtered and concentrated *in vacuo*. Purification by flash column chromatography (30% EtOAc/hexane) afforded oxetanol **5** (1.58 g, 88%) as a white solid. R_f = 0.23 (50% EtOAc/hexane); mp = 52–53 °C; IR (film)/cm^{-1} 3315 (br. OH), 2995, 2950, 2882, 1607, 1581, 1511, 1461, 1439, 1301, 1236, 1179, 1150, 1107, 1053, 1029, 969, 951, 875, 840, 816; ^{1}H NMR (400 MHz, $CDCl_3$) δ 7.53–7.51 (m, 2 H, 2 × Ar-CH), 6.99–6.96 (m, 2 H, 2 × Ar-CH), 4.95 (d, *J* = 6.9 Hz, 2 H, C*H*HOC*H*H), 4.93 (d, *J* = 6.9 Hz, 2 H, CH*H*OCH*H*), 3.86 (s, 3 H, CH_3), 2.43 (s, 1 H, OH); ^{13}C NMR (101 MHz, $CDCl_3$) δ 159.3 (Ar-C_q-O), 134.5 (Ar-C_q-C_q), 125.9 (2 × Ar-CH), 114.0 (2 × Ar-CH), 85.6 (2 × CH_2), 75.7 (C_q), 55.4 (CH_3); HRMS (ESI-TOF) *m/z* calcd for $C_{10}H_{11}O_2^+$ [M-OH]$^+$: 163.0759, Found: 163.0752. Compound was previously reported in the literature but no spectroscopic data was provided.[5]

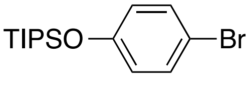

4-Bromophenoxytris(propan-2-yl)silane[6]

2,6-Lutidine (1.70 mL, 14.4 mmol) was added dropwise to a solution of 4-bromophenol (2.08 g, 12.0 mmol), triisopropylsilyl trifluoromethanesulfonate (4.05 g, 13.2 mmol) in dichloromethane (16 mL). The reaction mixture was stirred at 0 °C for 2 h then concentrated *in vacuo*. The residue was taken up in EtOAc (30 mL) and water (30 mL) was added. The layers were separated and the organic portion was washed with water (20 mL) then brine (20 mL). The organic extract was dried over Na_2SO_4, filtered and concentrated *in vacuo*. Purification by flash column chromatography (100% hexane) afforded the silane (3.69 g, 93%) as a colorless oil. R_f = 0.5 (100% hexane); IR (film)/cm^{-1} 2945, 2867, 1586, 1486, 1463, 1261, 1070, 1007, 996, 907, 882, 826, 731, 683; ^{1}H NMR (400 MHz, $CDCl_3$) δ 7.35–7.29 (m, 2 H, 2 × Ar-CH), 6.80–6.73 (m, 2 H, 2 × Ar-CH), 1.33–1.18 (m, 3 H, 3 × CH), 1.11 (d, *J* = 7.4 Hz, 18 H, 6 × CH_3); ^{13}C NMR (101 MHz, $CDCl_3$) δ 155.3 (Ar-C_q-O), 132.2 (2 × Ar-CH), 121.7 (2 × Ar-CH), 113.2 (Ar-C_q-Br), 17.9 (6 × CH_3), 12.6 (3 × CH). The observed spectroscopic data for this compound was consistent with that previously reported.[7]

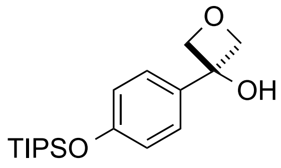

3-(4-{[Tris(propan-2-yl)silyl]oxy}phenyl)oxetan-3-ol (6)

n-BuLi (2.24 M in THF, 4.46 mL, 10.0 mmol) was added dropwise over 5 min to a solution of 4-Bromophenoxytris(propan-2-yl)silane (3.29 g, 10.0 mmol) in THF (35 mL) at –78 °C. The reaction mixture was stirred at –78 °C for a further 10 min. Oxetan-3-one (0.49 mL, 7.70 mmol) in THF (2.5 mL) was added dropwise to the reaction mixture. Following a further 10 min at –78 °C the reaction mixture was warmed to rt then quenched with water (30 mL). The layers were separated and the aqueous portion extracted with diethylether (3 × 30 mL). The organic extracts were combined, washed with brine, dried over Na_2SO_4, filtered and concentrated *in vacuo*. Purification by flash column chromatography (30% EtOAc/hexane) afforded oxetanol **6** (1.96 g, 79%) as a white solid. R_f = 0.29 (30% EtOAc/hexane); mp = 63–64 °C; IR (film)/cm^{-1} 3404 (br. OH), 2945, 2868, 2852, 1605, 1513, 1461, 1421, 1251, 1171, 1104, 1076, 1009, 999, 977, 953, 908, 881, 843, 820, 738, 727, 688; ^{1}H NMR (400 MHz, $CDCl_3$) δ 7.44–7.40 (m, 2 H, 2 × Ar-CH), 6.94–6.90 (m, 2 H, 2 × Ar-CH), 4.93 (d, *J* = 7.0 Hz, 2 H, C*H*HOC*H*H), 4.90 (d, *J* = 7.0 Hz, 2 H, CH*H*OCH*H*), 2.41 (s, 1 H, OH), 1.31–1.22 (m, 3 H, 3 × CH), 1.11 (d, *J* = 7.4 Hz, 18 H, 6 × CH_3); ^{13}C NMR (101 MHz, $CDCl_3$) δ 155.8 (Ar-C_q-O), 134.7 (Ar-C_q-C_q), 125.8 (2 × Ar-CH), 119.9 (2 × Ar-CH), 85.5 (2 × CH_2), 75.7 (C_q), 17.9 (6 × CH_3), 12.6 (3 × CH); HRMS (ESI-TOF) *m/z* calcd for $C_{18}H_{29}O_2Si^+$ [M-OH]$^+$: 305.1937, Found: 305.1935.

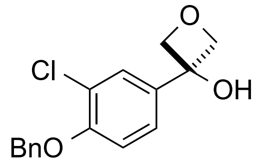

3-[4-(Benzyloxy)-3-chlorophenyl]oxetan-3-ol (7)

n-BuLi (2.50 M in THF, 14.8 mL, 37.0 mmol) was added dropwise to a solution of 1-(benzyloxy)-4-bromo-2-chlorobenzene (10.0 g, 33.6 mmol) in THF (100 mL) at –78 °C. The reaction mixture was stirred at –30 °C for 1 h. Oxetan-3-one (2.82 mL, 43.7 mmol) in THF (10 mL) was added dropwise to the reaction mixture at 0 ºC. The reaction mixture was warmed to rt and stirred for 16 h. The reaction mixture was filtered through celite, washed with EtOAc (100 mL) and concentrated *in vacuo*. Purification by flash column chromatography (0–20% EtOAc/heptane) afforded oxetanol **7** (4.50 g, 46%) as a white solid. R_f = 0.18 (25% EtOAc/Petroleum ether); mp = 84–87 °C; IR (film)/cm^{-1} 3363 (br. OH), 2970, 1739, 1606, 1507, 1454, 1430, 1385, 1294, 1264, 1219, 1055, 1005, 956, 906, 888, 853, 833, 809, 741, 722, 694, 654; ^{1}H NMR (400 MHz, $CDCl_3$) δ 7.64 (d, *J* = 2.3 Hz, 1 H, Ar-CH), 7.55–7.17 (m, 6 H, 6 × Ar-CH), 7.00 (d, *J* = 8.5 Hz, 1 H, Ar-CH), 5.20 (s, 2 H, $PhCH_2$), 4.88 (d, *J* = 6.7 Hz, 2 H, C*H*HOC*H*H), 4.86 (d, *J* = 6.7 Hz, 2 H, CH*H*OCH*H*), 2.66 (s, 1 H, OH); ^{13}C NMR (101 MHz, $CDCl_3$) δ 153.7 (Ar-C_q-OBn), 136.3 (Ph-C_q), 135.9 (Ar-C_q-C_q), 128.6 (2 × Ar-CH), 128.1 (Ar-CH), 127.0 (2 × Ar-CH), 126.9 (Ar-CH), 123.8 (Ar-CH), 123.6 (Ar-C_q-Cl), 114.0 (Ar-CH), 85.6 (CH_2OCH_2), 75.2 (C_q), 70.9 ($PhCH_2$); HRMS (ESI-TOF) *m/z* calcd for $C_{16}H_{15}O_3ClNa^+$ $[M+Na]^+$:313.0602, Found: 313.0598. The observed spectroscopic data for this compound was consistent with that previously reported.[8]

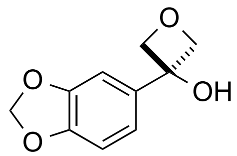

3-(2H-1,3-Benzodioxol-5-yl)oxetan-3-ol (8)

n-BuLi (2.50 M in THF, 16.4 mL, 16.4 mmol) was added dropwise over 10 min to a solution of 5-bromo-2H-1,3-benzodioxole (3.00 g, 14.9 mmol) in THF (30 mL) at –70 °C. The reaction mixture was stirred at –70 °C for 1 h. Oxetan-3-one (0.96 mL, 15.0 mmol) in THF (10 mL) was added dropwise to the reaction mixture at –70 ºC. The reaction mixture was warmed to rt and stirred for 12 h then quenched with sat. aq NH_4Cl (10 mL). The layers were separated and the aqueous portion extracted with EtOAc (3 × 50 mL). The organic extracts were combined, dried over Na_2SO_4, filtered and concentrated *in vacuo*. Purification by flash column chromatography (5–50% EtOAc/Petroleum ether) afforded oxetanol **8** (2.10 g, 73%) as a pale yellow solid. R_f = 0.13 (30% EtOAc/hexane); mp = 88–91 °C; IR (film)/cm^{-1} 3333 (br. OH), 2971, 2875, 1736, 1485, 1435, 1248, 1175, 1099, 1035, 960, 938, 857, 811, 755, 726, 667; ^{1}H NMR (400 MHz, $CDCl_3$) δ 7.08–7.02 (m, 2 H, 2 × Ar-CH), 6.84 (d, *J* = 8.0 Hz, 1 H, Ar-CH), 5.99 (s, 2 H, OCH_2O), 4.88 (s, 4 H, CH_2OCH_2), 2.69 (s, 1 H, OH); ^{13}C NMR (101 MHz, $CDCl_3$) δ Ar-C_q-OCH_2O), 147.3 (Ar-C_q-OCH_2O), 136.4 (Ar-C_q-C_q), 117.9 (Ar-CH), 108.2 (Ar-CH), 105.5 (Ar-CH), 101.2 (OCH_2O), 85.5 (CH_2OCH_2), 75.8 (C_q); HRMS (ESI-TOF) *m/z* calcd for $C_{10}H_9O_3^+$ $[M–OH]^+$: 177.0552, Found: 177.0550.

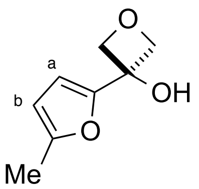

3-(5-Methylfuran-2-yl)oxetan-3-ol (9)

n-BuLi (2.50 M in THF, 14.0 mL, 35.1 mmol) was added dropwise over 5 min to a solution of 2-methylfuran (3.00 g, 36.5 mmol) in THF (30 mL) at –10 °C. The reaction mixture was stirred at 0 °C for a further 1 h. Oxetan-3-one (2.04 mL, 31.9 mmol) in THF (15 mL) was added dropwise to the reaction mixture at –10 °C. Following a further 15 h at 15 °C the reaction mixture was warmed to rt then quenched with sat. aq NH_4Cl (10 mL). The layers were separated and the aqueous portion extracted with EtOAc (3 × 50 mL). The organic extracts were combined, dried over Na_2SO_4, filtered and concentrated *in vacuo*. Purification by flash column chromatography (0–50% EtOAc/Petroleum ether) afforded oxetanol **9** (2.30 g, 47%) as a colorless oil. R_f = 0.64 (50% Petroleum ether/EtOAc); IR (film)/cm^{-1} 3366 (br. OH), 2951, 2879, 1738, 1565, 1217, 1174, 1140, 1087, 1019, 970. 953, 942, 866, 784, 674; ^{1}H NMR (400 MHz, $CDCl_3$) δ 6.29 (d, *J* = 3.3 Hz, 1 H, Ar-CH_a), 6.01–5.98 (m, 1 H, Ar-CH_b), 4.93 (d, *J* = 6.5 Hz, 2 H, C*H*HOC*H*H), 4.84 (dd, *J* = 6.5, 1.3 Hz, 2 H, CH*H*OCH*H*), 2.74 (s, 1 H, OH), 2.33 (s, 3 H, CH_3); ^{13}C NMR (101 MHz, $CDCl_3$) δ 152.5 (Ar-C_q), 152.4 (Ar-C_q), 106.9. (Ar-CH), 106.4 (Ar-CH), 83.1 (CH_2OCH_2), 72.1 (C_q), 13.5 (CH_3); HRMS (ESI-TOF) *m/z* calcd for $C_8H_9O_2^+$ $[M-H_2O]^+$: 137.0597, Found: 137.0597.

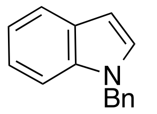

1-Benzyl-1H-indole[9]

Indole (1.17 g, 10.0 mmol) in DMF (10 mL) was added to a solution of sodium hydride (60% in mineral oil, 480 mg, 12.0 mmol) in DMF (10 mL) at 0 °C. The reaction mixture was stirred at 25 °C for 30 min then benzyl bromide (1.80 mL, 15.0 mmol) was added dropwise. The resulting solution was stirred at 25 °C for a further 12 h then quenched with water (20 mL). Dichloromethane (15 mL) was added and the layers separated. The organic extract was washed with water (3 × 20 mL), dried over Na_2SO_4, filtered and concentrated *in vacuo*. Purification by flash column chromatography (3% EtOAc/hexane) afforded 1-benzyl-1H-indole (1.90 g, 91%) as a pale yellow solid. R_f = 0.32 (3% EtOAc/hexane); mp = 39–41 °C [Lit; 41 °C][10]; IR (film)/cm^{-1} 3025, 1738, 1508, 1485, 1463, 1455, 1440, 1354, 1336, 1316, 1178, 1078, 765, 740, 715, 695; ^{1}H NMR (400 MHz, $CDCl_3$) δ 7.70 (d, *J* = 7.7 Hz, 1 H, Ar-CH), 7.49–7.09 (m, 9 H, 9 × Ar-CH), 6.60 (dd, *J* = 2.9, 1.7 Hz, 1 H, $Ar_{(indole-3H)}$-CH), 5.37 (CH_2); ^{13}C NMR (101 MHz, $CDCl_3$) δ 137.5 (Ph-C_q), 136.3 ($Ar_{(indole)}$-C_q), 128.7 (2 × $Ar_{(Bn)}$-CH), 128.4 ($Ar_{(indole)}$-C_q), 128.2 ($Ar_{(indole)}$-CH), 127.6 (Ph-CH), 126.7 (2 × $Ar_{(Bn)}$-CH), 121.7 ($Ar_{(indole)}$-CH), 120.9 ($Ar_{(indole)}$-CH), 119.6 ($Ar_{(indole)}$-CH), 109.7 ($Ar_{(indole)}$-CH), 101.7 ($Ar_{(indole)}$-CH), 50.1 (Ph-CH_2). The observed spectroscopic data for this compound was consistent with that previously reported.[11]

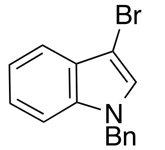

1-Benzyl-3-bromo-1H-indole[12]

1,3-Dibromo-5,5-dimethylhydantoin (786 mg, 2.75 mmol) was added portionwise over 10 min to a solution of 1-benzyl-1H-indole (1.04 g, 5.00 mmol) in 1,4-dioxane (17 mL) at 10 °C. The resulting solution was stirred for 5 minutes and then sat. aq. $NaHCO_3$ (30 mL) was added followed by EtOAc (15 mL). The layers were separated and the aqueous portion was extracted with EtOAc (3 × 15 mL). The organic extract was dried over Na_2SO_4, filtered and concentrated *in vacuo*. Purification by flash column chromatography (100% hexane) afforded 1-Benzyl-3-bromo-1H-indole (877 mg, 61%) as a pink oil. R_f = 0.27 (30% EtOAc/hexane); IR (film)/cm^{-1} 3253, 2963, 1739, 1609, 1500, 1464, 1382, 1270, 1252, 1230, 1125, 1062, 1015, 978, 939, 823, 739, 724, 694; ^{1}H NMR (400 MHz, $CDCl_3$) δ 7.62 (d, *J* = 6.9 Hz, 1 H, Ar-CH), 7.48–7.02 (m, 9 H, 9 × Ar-CH), 5.31 (s, 2 H, CH_2); ^{13}C NMR (101 MHz, $CDCl_3$) δ 136.7 (Ph-C_q), 135.9 ($Ar_{(indole)}$-C_q), 128.9 (2 × $Ar_{(Bn)}$-CH), 127.8 (Ph-CH), 127.5 ($Ar_{(indole)}$-C_q), 127.0 ($Ar_{(indole)}$-CH), 126.9 (2 × $Ar_{(Bn)}$-CH), 122.8 ($Ar_{(indole)}$-CH), 120.3 ($Ar_{(indole)}$-CH), 119.4 ($Ar_{(indole)}$-CH), 109.9 ($Ar_{(indole)}$-CH), 90.2 ($Ar_{(indole)}$-C_q-Br), 50.1 ($PhCH_2$). The observed spectroscopic data for this compound was consistent with that previously reported.[13]

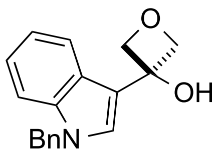

3-(1-Benzyl-1H-indol-3-yl)oxetan-3-ol (10)

n-BuLi (2.45 M in THF, 0.97 mL, 2.40 mmol) was added dropwise over 5 min to a solution of 1-Benzyl-3-bromo-1H-indole (859 mg, 3.00 mmol) in THF (15 mL) at –78 °C. The reaction mixture was stirred at –78 °C for a further 5 min. Oxetan-3-one (0.13 mL, 2.00 mmol) was added dropwise to the reaction mixture. Following a further 30 min at –78 °C the reaction mixture was warmed to rt then quenched with water (15 mL). The layers were separated and the aqueous portion extracted with diethyl ether (3 × 30 mL). The organic extracts were combined, washed with brine (30 mL), dried over Na_2SO_4, filtered and concentrated *in vacuo*. Purification by flash column chromatography (40% EtOAc/hexane) afforded oxetanol **10** (504 mg, 90%) as a beige solid. R_f = 0.19 (40% EtOAc/hexane); mp = 118–120 °C; IR (film)/cm^{-1} 3278 (br. OH), 2986, 2876, 1739, 1553, 1496, 1467, 1451, 1243, 1198, 1179, 1140, 1087, 1071, 956, 863, 830, 742, 695; ^{1}H NMR (400 MHz, $CDCl_3$) δ 7.80 (dt, *J* = 7.9, 1.0 Hz, 1 H, Ar-CH), 7.36–7.14 (m, 9 H, 9 × Ar-CH), 5.34 (s, 2 H, CH_2Ph), 5.09 (d, *J* = 6.9 Hz, 2 H, C*H*HOC*H*H), 5.03 (d, *J* = 6.9 Hz, 2 H, CH*H*OCH*H*), 2.49 (s, 1 H, OH); ^{13}C NMR (101 MHz, $CDCl_3$) δ 137.4 (Ph-C_q), 136.9 ($Ar_{(indole)}$-C_q), 128.9 (2 × Ar-CH), 127.8 (Ar-CH), 126.9 (2 × Ar-CH), 125.5 ($Ar_{(indole)}$-C_q), 125.2 (Ar-CH), 122.5 (Ar-CH), 120.0 (Ar-CH), 119.9 (Ar-CH), 117.1 (Ar-C_q-C_q), 110.1 (Ar-CH), 84.7 (CH_2OCH_2), 73.1 (C_q), 50.1 ($PhCH_2$); FTMS (+ p NSI) *m/z* calcd for $C_{18}H_{18}O_2N^+$ $[M+H]^+$: 280.1332, Found: 280.1330.

Synthesis of Diaryloxetanes 2a–j, 11–16

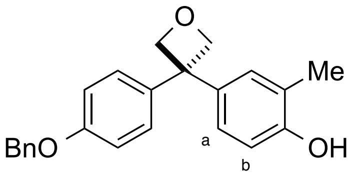

4-{3-[4-(Benzyloxy)phenyl]oxetan-3-yl}-2-methylphenol (2b)

Lithium bis(trifluoromethanesulfonimide) (7.9 mg, 0.0275 mmol) and tetrabutylammonium hexafluorophosphate (5.3 mg, 0.0138 mmol) were added to a solution of oxetanol **1** (64 mg, 0.25 mmol) and 2-methylphenol (135 mg, 1.25 mmol) in chloroform (0.5 mL). The reaction mixture was stirred at 40 °C for 1 h then sat. aq. $NaHCO_3$ (15 mL) was added followed by dichloromethane (15 mL). The layers were separated and the aqueous portion was extracted with dichloromethane (2 × 15 mL). The organic extracts were combined, dried over Na_2SO_4, filtered and concentrated *in vacuo*. Purification by flash column chromatography (20% EtOAc/hexane) afforded oxetane **2b** (63 mg, 73%) as a white solid. R_f = 0.11 (20% EtOAc/hexane); mp = 179–181 °C; IR (film)/cm^{-1} 3307 (br. OH), 1738, 1609, 1511, 1455, 1382, 1298, 1236, 1181, 1120, 1013, 972, 933, 910, 842, 810, 737, 695; ^{1}H NMR (400 MHz, $CDCl_3$) δ 7.49–7.29 (m, 5 H, 5 × Ar-CH), 7.21–7.10 (m, 2 H, 2 × Ar-CH), 7.04–6.91 (m, 3 H, 3 × Ar-CH), 6.88 (dd, *J* = 8.3, 2.4 Hz, 1 H, Ar-CH_a), 6.75 (d, *J* = 8.3 Hz, 1 H, Ar-CH_b), 5.21 (d, *J* = 5.6 Hz, 2 H, C*H*HOC*H*H), 5.18 (d, *J* = 5.6 Hz, 2 H, CH*H*OCH*H*), 5.07 (s, 2 H, $PhCH_2$), 4.70 (s, 1 H, OH), 2.25 (s, 3 H, CH_3). ^{13}C NMR (101 MHz, $(CD_3)_2SO$) δ 157.1 (Ar-C_q-OBn), 154.2 (Ar-C_q-OH), 139.8 ($Ar_{(phenol)}$-C_q-C_q), 137.6 ($Ar_{(p\text{-}OBn)}$-C_q-C_q), 137.2 (Ph-C_q), 128.9 (2 × Ar-CH), 128.8 (Ar-CH), 128.3 (Ar-CH), 128.1 (2 × Ar-CH), 127.7 (2 × Ar-CH), 124.8 (Ar-CH_a), 124.2 (Ar-C_q-CH_3), 115.1 (2 × Ar-CH), 114.8 (Ar-CH_b), 83.8 (CH_2OCH_2), 69.7 ($PhCH_2$), 50.6 (C_q), 16.7 (CH_3); FTMS (+ p NSI) m/z calcd for $C_{23}H_{26}O_3N^+$ $[M+NH_4]^+$: 364.1907, Found: 364.1913.

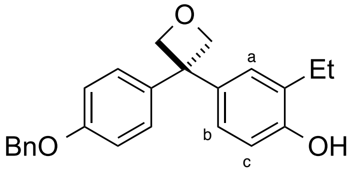

4-{3-[4-(Benzyloxy)phenyl]oxetan-3-yl}-2-ethylphenol (2c)

Lithium bis(trifluoromethanesulfonimide) (7.9 mg, 0.0275 mmol) and tetrabutylammonium hexafluorophosphate (5.3 mg, 0.0138 mmol) were added to a solution of oxetanol **1** (64 mg, 0.25 mmol) and 2-ethylphenol (0.15 mL, 1.25 mmol) in chloroform (0.5 mL). The reaction mixture was stirred at 40 °C for 1 h then sat. aq. $NaHCO_3$ (15 mL) was added followed by dichloromethane (15 mL). The layers were separated and the aqueous portion was extracted with dichloromethane (2 × 15 mL). The organic extracts were combined, dried over Na_2SO_4, filtered and concentrated *in vacuo*. Purification by flash column chromatography (25% EtOAc/hexane) afforded oxetane **2c** (60 mg, 66%) as a white solid. R_f = 0.29 (30% EtOAc/hexane); mp = 152–153 °C; IR (film)/cm^{-1} 3343 (br. OH), 2972, 2889, 1608, 1509, 1455, 1422, 1382, 1276, 1235, 1181, 1157, 1120, 1011, 970, 930, 842, 811, 735, 695; ^{1}H NMR (400 MHz, $CDCl_3$) δ 7.49–7.29 (m, 5 H, 5 × Ar-CH), 7.18–7.11 (m, 2 H, 2 × Ar-CH), 7.02 (d, *J* = 2.4 Hz, 1 H, Ar-CH_a), 7.00–6.93 (m, 2 H, 2 × Ar-CH), 6.85 (dd, *J* = 8.2, 2.4 Hz, 1 H, Ar-CH_b), 6.73 (d, *J* = 8.2 Hz, 1 H, Ar-CH_c), 5.22 (d, *J* = 5.6 Hz, 2 H, C*H*HOC*H*H), 5.19 (d, *J* = 5.6 Hz, 2 H, CH*H*OCH*H*), 5.07 (s, 2 H, Ph-CH_2), 4.71 (s, 1 H, OH), 2.63 (q, *J* = 7.6 Hz, C$H_2$$CH_3$), 1.22 (t, *J* = 7.6 Hz, CH_2CH_3); ^{13}C NMR (101 MHz, $CDCl_3$) δ 157.4 (Ar-C_q-OBn), 152.1 (Ar-C_q-OH), 138.6 ($Ar_{(phenol)}$-C_q-C_q), 138.0 ($Ar_{(p\text{-}OBn)}$-C_q-C_q), 136.9 (Ph-C_q), 130.2 (Ar-C_q-CH_2CH_3), 128.6 (2 × Ar-CH), 128.0 (Ar-CH), 127.6 (2 × Ar-CH), 127.48 (Ar-CH_a), 127.45 (2 × Ar-CH), 125.1 (Ar-CH_b), 115.0 (Ar-CH_c), 114.8 (2 × Ar-CH), 85.1 (CH_2OCH_2), 70.1 (Ph-CH_2), 50.3 (C_q), 23.2 (*C*H_2CH_3), 15.7 (CH_3); FTMS (+ p NSI) *m/z* calcd for $C_{24}H_{28}O_3N^+$ $[M+NH_4]^+$: 378.2064, Found: 378.2062.

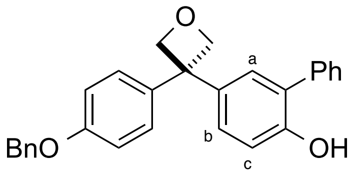

4-{3-[4-(Benzyloxy)phenyl]oxetan-3-yl}-2-phenylphenol (2d)

Lithium bis(trifluoromethanesulfonimide) (7.9 mg, 0.0275 mmol) and tetrabutylammonium hexafluorophosphate (5.3 mg, 0.0138 mmol) were added to a solution of oxetanol **1** (64 mg, 0.25 mmol) and 2-phenylphenol (213 mg, 1.25 mmol) in chloroform (0.5 mL). The reaction mixture was stirred at 40 °C for 1 h then sat. aq. $NaHCO_3$ (15 mL) was added followed by dichloromethane (15 mL). The layers were separated and the aqueous portion was extracted with EtOAc (3 × 15 mL). The organic extracts were combined, dried over Na_2SO_4, filtered and concentrated *in vacuo*. Purification by flash column chromatography (25% EtOAc/hexane) afforded oxetane **2d** (49 mg, 48%) as a white solid. R_f = 0.17 (25% EtOAc/hexane); mp = 175–178 °C; IR (film)/cm^{-1} 3307 (br. OH), 2964, 2888, 1739, 1608, 1579, 1511, 1492, 1454, 1406, 1379, 1294, 1273, 1235, 1119, 1009, 971, 930, 810, 748, 734,

696; ^{1}H NMR (400 MHz, $(CD_3)_2SO$) δ 9.49 (s, 1 H, OH), 7.55–7.44 (m, 2 H, 2 × Ar-CH), 7.47–7.26 (m, 8 H, 8 × Ar-CH), 7.26–7.21 (m, 2 H, 2 × Ar-CH), 7.14 (d, *J* = 2.4 Hz, 1 H, Ar-CH_a), 7.06 (dd, *J* = 8.3, 2.4 Hz, 1 H, Ar-CH_b), 6.98 (m, 2 H, 2 × Ar-CH), 6.91 (d, *J* = 8.3 Hz, 1 H, Ar-CH_c), 5.14 (d, *J* = 6.0 Hz, 2 H, C*H*HOC*H*H), 5.10–5.08 (m, 4 H, CH*H*OCH*H* + $PhCH_2$); ^{13}C NMR (101 MHz, $(CD_3)_2SO$) δ 7.1 (Ar-C_q-OBn), 153.1 (Ar-C_q-OH), 139.6 ($Ar_{(p\text{-OBn})}$-C_q-C_q), 139.0 (Ar-C_q-Ph), 138.0 ($Ar_{(\text{phenol})}$-C_q-C_q), 137.6 (Ph-C_q-CH_2), 129.6 (2 × Ar-CH), 128.9 (2 × Ar-CH), 128.5 (Ar-CH_a), 128.4 (2 × Ar-CH), 128.3 (Ar-CH), 128.1 (2 × Ar-CH), 127.7 (2 × Ar-CH), 127.0 (Ar-CH), 126.7 (Ar-CH_b), 116.4 (Ar-CH_c), 115.2 (2 × Ar-CH), 83.8 (CH_2OCH_2), 69.7 ($PhCH_2$), 50.7 (C_q); FTMS (+ p NSI) *m/z* calcd for $C_{28}H_{28}O_3N^+$ $[M+NH_4]^+$: 426.2064, Found: 426.2063.

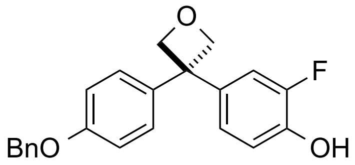

4-{3-[4-(Benzyloxy)phenyl]oxetan-3-yl}-2-fluorophenol (2e)

Lithium bis(trifluoromethanesulfonimide) (7.9 mg, 0.0275 mmol) and tetrabutylammonium hexafluorophosphate (5.3 mg, 0.0138 mmol) were added to a solution of oxetanol **1** (64 mg, 0.25 mmol) and 2-fluorophenol (140 mg, 1.25 mmol) in chloroform (0.5 mL). The reaction mixture was stirred at 40 °C for 1 h then sat. aq. $NaHCO_3$ (15 mL) was added followed by dichloromethane (15 mL). The layers were separated and the aqueous portion was extracted with EtOAc (3 × 15 mL). The organic extracts were combined, dried over Na_2SO_4, filtered and concentrated *in vacuo*. Purification by flash column chromatography (30% EtOAc/hexane) afforded oxetane **2e** (12 mg, 13%) as a white solid. R_f = 0.26 (30% EtOAc/hexane); mp = 153–156 °C; IR (film)/cm^{-1} 3244 (br. OH), 2885, 1609, 1512, 1455, 1382, 1297, 1243, 1180, 1119, 1011, 831, 740, 697; ^{1}H NMR (400 MHz, $CHCl_3$) δ 7.50–7.30 (m, 6 H, 6 × Ar-CH), 7.11 (d, *J* = 8.7 Hz, 2 H, 2 × Ar-CH), 7.04–6.87 (m, 4 H, 4 × Ar-CH), 5.29 (d, *J* = 3.9 Hz, 1 H, OH), 5.22 (d, *J* = 5.7 Hz, 2 H, C*H*HOC*H*H), 5.14 (d, *J* = 5.7 Hz, 2 H, CH*H*OCH*H*), 5.08 (s, 2 H, $PhCH_2$); ^{13}C NMR (101 MHz, $CHCl_3$) δ 157.6 (Ar-C_q-OBn), 150.6 (d, $J_{C\text{-}F}$ = 237.9 Hz, Ar-C_q-F), 142.1 (d, $J_{C\text{-}F}$ = 14.3 Hz, Ar-C_q-OH), 139.2 (d, $J_{C\text{-}F}$ = 4.8 Hz, $Ar_{(\text{phenol})}$-C_q-C_q), 137.5 ($Ar_{(\text{p-OBn})}$-C_q-C_q), 136.9 (Ph-C_q), 128.6 (2 × Ar-CH), 128.0 (Ar-CH), 127.6 (2 × Ar-CH), 127.5 (2 × Ar-CH), 122.8 (d, $J_{C\text{-}F}$ = 3.4 Hz, Ar-CH), 117.2 (Ar-CH), 115.0 (2 × Ar-CH), 114.4 (d, $J_{C\text{-}F}$ = 18.5 Hz, Ar-CH), 84.7 (CH_2OCH_2), 70.1 ($PhCH_2$), 50.4 (C_q); FTMS (+ p NSI) *m/z* calcd for $C_{22}H_{23}FO_3N^+$ $[M+NH_4]^+$: 368.1656, Found: 368.1654.

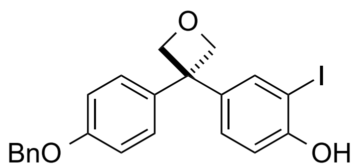

4-{3-[4-(Benzyloxy)phenyl]oxetan-3-yl}-2-iodophenol (2f)

Lithium bis(trifluoromethanesulfonimide) (7.9 mg, 0.0275 mmol) and tetrabutylammonium hexafluorophosphate (5.3 mg, 0.0138 mmol) were added to a solution of oxetanol **1** (64 mg, 0.25 mmol) and 2-iodophenol (275 mg, 1.25 mmol) in chloroform (0.5 mL). The reaction mixture was stirred at 40 °C for 1 h then sat. aq. $NaHCO_3$ (15 mL) was added followed by dichloromethane (15 mL). The layers were separated and the aqueous portion was extracted with dichloromethane (2 × 15 mL). The organic extracts were combined, dried over Na_2SO_4, filtered and concentrated *in vacuo*. Purification by flash column chromatography (30% EtOAc/hexane) afforded oxetane **2f** (21 mg, 18%) as a white solid. R_f = 0.24 (30% EtOAc/hexane); mp = 190–194 °C; IR (film)/cm^{-1} 3249 (br. OH), 3030, 2971, 1739, 1609, 1511, 1366, 1230, 1218, 1182, 1025, 829, 735, 697; ^{1}H NMR (400 MHz, $CDCl_3$) δ 7.51 (d, *J* = 2.3 Hz, 1 H, Ar-CH), 7.48–7.30 (m, 5 H, 5 × Ar-CH), 7.13–7.07 (m, 3 H, 3 × Ar-CH), 7.02–6.94 (m, 3 H, 3 × Ar-CH), 5.31 (s, 1 H, OH), 5.19 (d, *J* = 5.7 Hz, 2 H, C*H*HOC*H*H), 5.14 (d, *J* = 5.7 Hz, 2 H, CH*H*OCH*H*), 5.08 (s, 2 H, $PhCH_2$); ^{13}C NMR (101 MHz, $CDCl_3$) δ 157.6 (Ar-C_q-OBn), 153.5 (Ar-C_q-OH), 140.4 ($Ar_{(p\text{-OBn})}$-C_q-C_q), 137.6 (Ph-C_q), 136.9 ($Ar_{(\text{phenol-}}$$C_q$-$C_q$), 136.1 (Ar-CH), 128.61 (2 × Ar-CH), 128.55 (Ar-CH), 128.0 (Ar-CH), 127.6 (2 × Ar-CH), 127.5 (2 × Ar-CH), 114.96 (2 × Ar-CH), 114.94 (Ar-CH), 85.9 (Ar-C_q-I), 84.6 (CH_2OCH_2), 70.1 ($PhCH_2$), 50.0 (C_q); FTMS (+p NSI) *m/z* calcd for $C_{22}H_{23}O_3NI^+$ $[M+NH_4]^+$: 476.0717, Found: 476.0711.

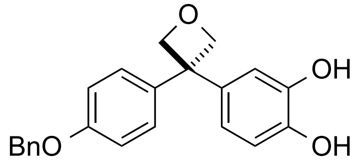

4-{3-[4-(Benzyloxy)phenyl]oxetan-3-yl}benzene-1,2-diol (2g)

Lithium bis(trifluoromethanesulfonimide) (7.9 mg, 0.0275 mmol) and tetrabutylammonium hexafluorophosphate (5.3 mg, 0.0138 mmol) were added to a solution of oxetanol **1** (64 mg, 0.25 mmol) and 1,2-dihydroxybenzene (138 mg, 1.25 mmol) in chloroform (0.5 mL). The reaction mixture was stirred at 40 °C for 1 h then sat. aq. $NaHCO_3$ (15 mL) was added followed by dichloromethane (15 mL). The layers were

separated and the aqueous portion was extracted with EtOAc (3 × 15 mL). The organic extracts were combined, dried over Na_2SO_4, filtered and concentrated *in vacuo*. Purification by flash column chromatography (45% EtOAc/hexane) afforded oxetane **2g** (55 mg, 63%) as a white solid. R_f = 0.10 (40% EtOAc/hexane); mp = 204–207 °C; IR (film)/cm^{-1} 3453 (br. OH), 2971, 1737, 1609, 1533, 1513, 1437, 1384, 1289, 1264, 1237, 1206, 1180, 1119, 1011, 968, 934, 864, 843, 811, 787, 741, 697; ^{1}H NMR (400 MHz, $(CD_3)_2SO$) δ 8.84 (s, 2 H, 2 × OH), 7.46–7.32 (m, 5 H, 5 × Ar-CH), 7.16 (d, *J* = 8.2 Hz, 2 H, 2 × Ar-CH), 6.99 (d, *J* = 8.5 Hz, 2 H, 2 × Ar-CH), 6.68 (d, *J* = 8.2 Hz, 1 H, Ar-CH), 6.60 (d, *J* = 2.5 Hz, 1 H, Ar-CH), 6.51 (dd, *J* = 8.5, 2.5 Hz, 1 H, Ar-CH), 5.09 (s, 2 H, $PhCH_2$), 5.04 (d, *J* = 6.0 Hz, 2 H, C*H*HOC*H*H), 5.02 (d, *J* = 6.0 Hz, 2 H, CH*H*OCH*H*); ^{13}C NMR (101 MHz, $(CD_3)_2SO$) δ 157.1 (Ar-C_q-O-CH_2), 145.6 (Ar-C_q-OH), 144.1 (Ar-C_q-OH), 139.5 ($Ar_{(p\text{-OBn})}$-C_q-C_q), 137.9 ($Ar_{(phenol)}$-C_q-C_q), 137.6 (Ph-C_q), 128.9 (2 × Ar-CH), 128.3 (Ar-CH), 128.1 (2 × Ar-CH), 127.8 (2 × Ar-CH), 117.3 (Ar-CH), 115.8 (Ar-CH), 115.1 (2 × Ar-CH), 114.2 (Ar-CH), 83.8 (CH_2OCH_2), 69.7 ($PhCH_2$), 50.5 (C_q); FTMS (+ p NSI) *m/z* calcd for $C_{22}H_{24}O_4N^+$ $[M+NH_4]^+$: 366.1700, Found: 366.1700.

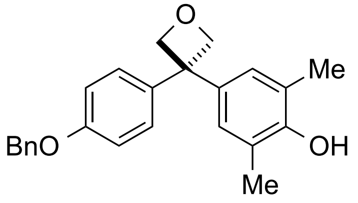

4-{3-[4-(Benzyloxy)phenyl]oxetan-3-yl}-2,6-dimethylphenol (2h)

Lithium bis(trifluoromethanesulfonimide) (7.9 mg, 0.0275 mmol) and tetrabutylammonium hexafluorophosphate (5.3 mg, 0.0138 mmol) were added to a solution of oxetanol **1** (64 mg, 0.25 mmol) and 2,5-dimethylphenol (153 mg, 1.25 mmol) in chloroform (0.5 mL). The reaction mixture was stirred at 40 °C for 1 h then sat. aq. $NaHCO_3$ (15 mL) was added followed by dichloromethane (15 mL). The layers were separated and the aqueous portion was extracted with EtOAc (3 × 15 mL). The organic extracts were combined, dried over Na_2SO_4, filtered and concentrated *in vacuo*. Purification by flash column chromatography (20% EtOAc/hexane) afforded oxetane **2h** (64 mg, 74%) as a white solid. R_f = 0.21 (20% EtOAc/hexane); mp = 166–167 °C; IR (film)/cm^{-1} 3291 (br. OH), 2960, 2886, 1607, 1579, 1509, 1489, 1456, 1381, 1290, 1320, 1310, 1243, 1226, 1178, 1121, 1014, 976, 952, 900, 864, 834, 814, 732, 967, 747, 600; ^{1}H NMR (400 MHz, $CHCl_3$) δ 7.47–7.29 (m, 5 H, 5 × Ar-CH), 7.18–7.09 (m, 2 H, 2 × Ar-CH), 6.98–6.92 (m, 2 H, 2 × Ar-CH), 6.80 (s, 2 H, Ar-CH), 5.21 (d, *J* = 5.6 Hz, 2 H, C*H*HOC*H*H), 5.16 (d, *J* = 5.6 Hz, 2 H, CH*H*OCH*H*), 5.07 (s, 2 H, $PhCH_2$), 4.60–4.55 (m, 1 H, OH), 2.24 (s, 6 H, 2 × CH_3); ^{13}C NMR (101 MHz, $CHCl_3$) δ 157.3 (Ar-C_q-OBn), 150.8 (Ar-C_q-OH), 138.7 ($Ar_{(phenol)}$-C_q-C_q), 137.5 ($Ar_{(p\text{-OBn})}$-C_q-C_q), 136.9 (Ph-C_q), 128.6 (2 × Ar-CH), 128.0 (Ar-CH), 127.6 (2 × Ar-CH), 127.5 (2 × Ar-CH), 126.7 (2 × Ar-CH), 123.1 (2 × Ar-C_q-CH_3), 114.8 (2 × Ar-CH), 85.0 (CH_2OCH_2), 70.1 ($PhCH_2$), 50.3 (C_q), 16.1 (2 × CH_3); FTMS (+ p NSI) *m/z* calcd for $C_{24}H_{28}O_3N^+$ $[M+NH_4]^+$: 378.2064, Found: 378.2060.

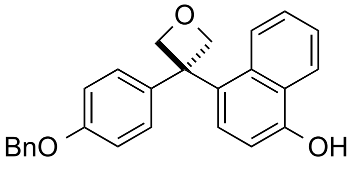

{3-[4-(Benzyloxy)phenyl]-2H,3H-naphtho[1,2-b]furan-3-yl}methanol (2i)

Lithium bis(trifluoromethanesulfonimide) (7.9 mg, 0.0275 mmol) and tetrabutylammonium hexafluorophosphate (5.3 mg, 0.0138 mmol) were added to a solution of oxetanol **1** (64 mg, 0.25 mmol) and 1-napthol (180 mg, 1.25 mmol) in chloroform (0.5 mL). The reaction mixture was stirred at 40 °C for 1 h then sat. aq. $NaHCO_3$ (15 mL) was added followed by dichloromethane (15 mL). The layers were separated and the aqueous portion was extracted with dichloromethane (2 × 15 mL). The organic extracts were combined, dried over Na_2SO_4, filtered and concentrated *in vacuo*. Purification by flash column chromatography (30% EtOAc/hexane) afforded oxetane **2i** (42 mg, 44%) as a cream solid. R_f = 0.13 (30% EtOAc/hexane); mp = 221–224 °C; IR (film)/cm^{-1} 3223 (br. OH), 2967, 1887, 1625, 1605, 1588, 1507, 1467, 1454, 1373, 1251, 1282, 1236, 1183, 1151, 1121, 1053, 1027, 1014, 981, 949, 918, 864, 833, 813, 762, 743, 713, 698; ^{1}H NMR (400 MHz, $(CD_3)_2SO$) δ 10.21 (s, 1H, OH), 8.20, (dd, *J* = 8.3, 1.4 Hz, 1 H, Ar-H), 7.45–7.21 (m, 10 H, 10 × Ar-CH), 6.99–6.87 (m, 4 H, 4 × Ar-CH), 5.31 (d, *J* = 5.6 Hz, 2 H, C*H*HOC*H*H), 5.07 (d, *J* = 5.6 Hz, 2 H, CH*H*OCH*H*), 5.04 (s, 2 H, $PhCH_2$). ^{13}C NMR (101 MHz, $(CD_3)_2SO$) δ 157.4 (Ar-C_q-OBn), 152.9 (Ar-C_q-OH), 139.1 ($Ar_{(p\text{-OBn})}$-C_q-C_q), 137.6 (Ph-C_q), 131.6 ($Ar_{(napthol)}$-C_q-C_q), 131.3 ($Ar_{(napthol)}$-C_q), 128.9 (2 × Ar-CH), 128.3 (Ar-CH), 128.1 (2 × Ar-CH), 127.4 (2 × Ar-CH), 126.7 (Ar-CH), 126.1 (Ar-CH), 125.8 ($Ar_{(napthol)}$-C_q), 125.0 (Ar-CH), 124.7 (Ar-CH), 123.4 (Ar-CH), 115.0 (2 × Ar-CH), 107.6 (Ar-CH), 84.1 (CH_2OCH_2), 69.6 ($PhCH_2$), 50.6 (C_q); FTMS (+ p NSI) m/z calcd for $C_{26}H_{26}O_3N^+$ $[M+NH_4]^+$: 400.1907, Found: 400.1905.

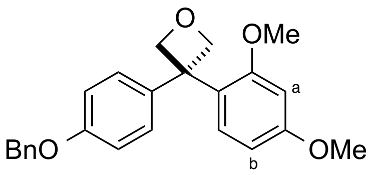

3-[4-(Benzyloxy)phenyl]-3-(2,4-dimethoxyphenyl)oxetane (2j)

Lithium bis(trifluoromethanesulfonimide) (7.9 mg, 0.0275 mmol) and tetrabutylammonium hexafluorophosphate (5.3 mg, 0.0138 mmol) were added to a solution of oxetanol **1** (64 mg, 0.25 mmol) and dimethoxybenzene (0.16 mL, 1.25 mmol) in chloroform (0.5 mL). The reaction mixture was stirred at 40 °C for 1 h then sat. aq. $NaHCO_3$ (15 mL) was added followed by dichloromethane (15 mL). The layers were separated and the aqueous portion was extracted with dichloromethane (2 × 15 mL). The organic extracts were combined, dried over Na_2SO_4, filtered and concentrated *in vacuo*. Purification by flash column chromatography (30% EtOAc/hexane) afforded oxetane **2j** (16 mg, 17%) as a colorless oil. R_f = 0.35 (30% EtOAc/hexane); IR (film)/cm^{-1} 2954, 2876, 2836, 1609, 1583, 1509, 1454, 1438, 1417, 1381, 1287, 1241, 1207, 1180, 1157, 1140, 1118, 1076, 1056, 1026, 981, 945, 911, 827, 732, 696; ^{1}H NMR (400 MHz, $CDCl_3$) δ 7.53–7.28 (m, 7 H, 7 × Ar-CH), 7.04–6.87 (m, 3 H, 3 × Ar-CH), 6.55 (dd, *J* = 8.3, 2.4 Hz, 1 H, Ar-CH_a), 6.49 (d, *J* = 2.4 Hz, 1 H, Ar-CH_b), 5.32 (d, *J* = 5.9 Hz, 2 H, C*H*HOC*H*H), 4.97 (d, *J* = 5.9 Hz, 2 H, CH*H*OCH*H*), 3.84 (s, 3 H, OCH_3), 3.67 (s, 3 H, OCH_3). ^{13}C NMR (101 MHz, $CDCl_3$) δ 159.9 (Ar-C_q-OCH_3), 157.7 (Ar-C_q-OCH_3), 157.3 (Ar-C_q-OBn), 139.0 ($Ar_{(OMe)}$-C_q-C_q), 137.1 (Ph-C_q), 128.5 (2 × Ar-CH), 127.9 (Ar-CH), 127.8 (Ar-CH), 127.4 (2 × Ar-CH), 126.9 (2 × Ar-CH) 125.4 ($Ar_{(OBn)}$-C_q-C_q), 114.4 (2 × Ar-CH), 104.1 (Ar-CH_b), 99.1 (Ar-CH_a), 84.5 (*C*H_2O*C*H_2), 70.0 ($PhCH_2$), 55.4 (OCH_3), 55.2 (OCH_3), 49.0 (C_q); HRMS (ESI-TOF) *m/z* calcd for $C_{24}H_{25}O_4^+$ $[M+H]^+$: 377.1752, Found: 377.1753.

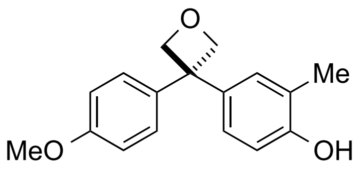

4-[3-(4-Methoxyphenyl)oxetan-3-yl]-2-methylphenol (11)

Lithium bis(trifluoromethanesulfonimide) (7.9 mg, 0.0275 mmol) and tetrabutylammonium hexafluorophosphate (5.3 mg, 0.0138 mmol) were added to a solution of oxetanol **5** (45 mg, 0.25 mmol) and 2-methylphenol (135 mg, 1.25 mmol) in chloroform (0.5 mL). The reaction mixture was stirred at 40 °C for 1 h then sat. aq. $NaHCO_3$ (15 mL) was added followed by dichloromethane (15 mL). The layers were separated and the aqueous portion was extracted with dichloromethane (2 × 15 mL). The organic extracts were combined, dried over Na_2SO_4, filtered and concentrated *in vacuo*. Purification by flash column chromatography (25% EtOAc/hexane) afforded oxetane **11** (52 mg, 77%) as a white solid. R_f = 0.20 (25% EtOAc/hexane); mp = 141–143 °C; IR (film)/cm^{-1} 3236 (br. OH), 2963, 1739, 1610, 1579, 1507, 1461, 1413, 1365, 1294, 1242, 1217, 1179, 1117, 1030, 976, 941, 901, 833, 819, 766, 727; ^{1}H NMR (400 MHz, $CDCl_3$) δ 7.19–7.10 (m, 2 H, 2 × Ar-CH), 7.00 (d, *J* = 2.4 Hz,1 H, Ar-CH), 6.94–6.82 (m, 3 H, 3 × Ar-CH), 6.73 (d, *J* = 8.3 Hz, 1 H, Ar-CH), 5.22 (d, *J* = 5.6 Hz, 2 H, C*H*HOC*H*H), 5.20 (d, *J* = 5.6 Hz, 2 H, CH*H*OCH*H*), 5.06 (s, 1 H, OH), 3.82 (OCH_3), 2.25 (s, 3 H, $ArCH_3$); ^{13}C NMR (101 MHz, $(CD_3)_2SO$) δ 158.0 (Ar-C_q-OMe), 154.2 (Ar-C_q-OH), 139.5 ($Ar_{(OMe)}$-C_q-C_q), 137.2 ($Ar_{(phenol)}$-C_q-C_q), 128.8 (Ar-CH), 127.7 (2 × Ar-CH), 124.8 (Ar-CH), 124.2 (Ar-C_q-CH_3), 114.8 (Ar-CH), 114.2 (2 × Ar-CH), 83.8 (CH_2OCH_2), 55.5 (OCH_3), 50.5 (C_q), 16.6 ($ArCH_3$); FTMS (+ p NSI) *m/z* calcd for $C_{17}H_{19}O_3^+$ $[M+H]^+$: 271.1329, Found: 271.1331.

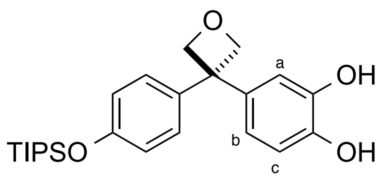

4-[3-(4-{[Tris(propan-2-yl)silyl]oxy}phenyl)oxetan-3-yl]benzene-1,2-diol (12)

Lithium bis(trifluoromethanesulfonimide) (7.9 mg, 0.0275 mmol) and tetrabutylammonium hexafluorophosphate (5.3 mg, 0.0138 mmol) were added to a solution of oxetanol **6** (81 mg, 0.25 mmol) and 2-hydroxyphenol (138 mg, 1.25 mmol) in chloroform (0.5 mL). The reaction mixture was stirred at 40 °C for 1 h then sat. aq. $NaHCO_3$ (15 mL) was added followed by dichloromethane (15 mL). The layers were separated and the aqueous portion was extracted with EtOAc (3 × 15 mL). The organic extract was dried over Na_2SO_4, filtered and concentrated *in vacuo*. Purification by flash column chromatography (25% EtOAc/hexane) afforded oxetane **12** (41 mg, 39%) as a white solid. R_f = 0.12 (25% EtOAc/hexane); mp = 162–163 °C; IR (film)/cm^{-1} 3384 (br. OH), 2945, 2866, 1741, 1606, 1508, 1462, 1382, 1263, 1174, 1122, 914, 882, 843, 811, 787, 755, 735, 707, 687; ^{1}H NMR (400 MHz, $(CD_3)_2SO$) δ 8.84 (s, 1 H, OH), 8.83 (s, 1 H, OH), 7.14–7.08 (m, 2 H, 2 × Ar-CH), 6.86–6.79 (m, 2 H, 2 × Ar-CH), 6.67 (d, *J* = 8.1 Hz, 1 H,

Ar-CH_c), 6.59 (d, *J* = 2.2 Hz, 1 H, Ar-CH_a), 6.46 (dd, *J* = 8.1. 2.2 Hz, 1 H, Ar-CH_b), 5.01 (s, 4 H, CH_2OCH_2), 1.30–1.16 (m, 3 H, 3 × C*H*($CH_3)_2$), 1.05 (d, *J* = 7.3 Hz, 18 Hz, 3 x ($CH_3)_2$); ^{13}C NMR (101 MHz, $(CD_3)_2SO$) δ 154.1 (Ar-C_q-OTIPS), 145.6 (Ar-C_q-OH), 144.1 (Ar-C_q-OH), 139.8 ($Ar_{(p\text{-OTIPS})}$-C_q-C_q), 137.8 ($Ar_{(phenol)}$-C_q-C_q), 127.9 (2 × $Ar_{(p\text{-OTIPS})}$-CH), 119.7 (2 × $Ar_{(p\text{-OTIPS})}$-CH), 117.4 ($Ar_{(phenol)}$-CH), 115.8 ($Ar_{(phenol)}$-CH), 114.3 ($Ar_{(phenol)}$-CH), 83.9 (CH_2OCH_2), 50.5 (C_q), 18.2 (6 × CH_3), 12.5 (3 × CH); FTMS (+ p NSI) *m/z* calcd for $C_{24}H_{35}O_4Si^+$ $[M+H]^+$: 415.2299, Found: 415.2302.

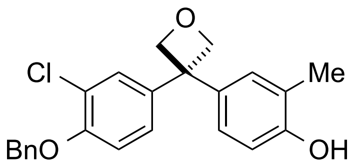

4-{3-[4-(Benzyloxy)-3-chlorophenyl]oxetan-3-yl}-2-methylphenol (13)

Lithium bis(trifluoromethanesulfonimide) (7.9 mg, 0.0275 mmol) and tetrabutylammonium hexafluorophosphate (5.3 mg, 0.0138 mmol) were added to a solution of oxetanol **7** (73 mg, 0.25 mmol) and 2-methylphenol (135 mg, 1.25 mmol) in chloroform (0.5 mL). The reaction mixture was stirred at 40 °C for 1 h then sat. aq. $NaHCO_3$ (15 mL) was added followed by dichloromethane (15 mL). The layers were separated and the aqueous portion was extracted with dichloromethane (2 × 15 mL). The organic extracts were combined, dried over Na_2SO_4, filtered and concentrated *in vacuo*. Purification by flash column chromatography (30% EtOAc/hexane) afforded oxetane **13** (32 mg, 33%) as a white solid. R_f = 0.27 (30% EtOAc/hexane); mp = 151–152 °C; IR (film)/cm^{-1} 3253 (br. OH), 2963, 1739, 1609, 1500, 1464, 1382, 1270, 1252, 1230, 1125, 1062, 1015, 978, 939, 823, 739, 724, 694; ^{1}H NMR (400 MHz, $(CD_3)_2SO$) δ 9.25 (s, 1 H, OH), 7.51–7.28 (m, 6 H, 6 × Ar-CH), 7.22–7.13 (m, 2 H, 2 × Ar-CH), 6.98 (d, *J* = 1.6 Hz, 1 H, Ar-CH), 6.87 (d, *J* = 8.3 Hz, 1 H, Ar-CH), 6.72 (dd, *J* = 8.3, 1.6 Hz, 1 H, Ar-CH), 5.19 (s, 2 H, $PhCH_2$), 5.05 (d, *J* = 6.1 Hz, 2 H, C*H*HOC*H*H), 5.02 (d, *J* = 6.1 Hz, 2 H, CH*H*OCH*H*), 2.09 (s, 3 H, CH_3); ^{13}C NMR (101 MHz, $(CD_3)_2SO$) δ 154.3 (Ar-C_q-OBn), 152.3 (Ar-C_q-OH), 141.1 ($Ar_{(phenol)}$-C_q-C_q), 137.1 ($Ar_{(p\text{-OBn})}$-C_q-C_q), 136.5 (Ph-C_q), 129.0 (2 × Ar-CH), 128.8 (Ar-CH), 128.4 (Ar-CH), 128.1 (Ar-CH), 127.9 (2 × Ar-CH), 126.3 (Ar-CH), 124.8 (Ar-CH), 124.4 (Ar-C_q-CH_3), 121.9 (Ar-C_q-Cl), 114.9 (Ar-CH), 114.7 (Ar-CH), 83.5 (CH_2OCH_2), 70.5 ($PhCH_2$), 50.6 (C_q), 16.6 (CH_3); FTMS (+ p NSI) *m/z* calcd for $C_{23}H_{25}O_3N^{35}Cl^+$ $[M+NH_4]^+$: 398.1517, Found: 398.1517.

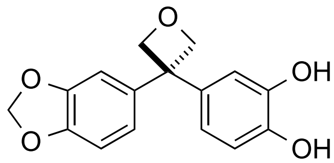

4-[3-(2H-1,3-Benzodioxol-5-yl)oxetan-3-yl]benzene-1,2-diol (14)

Lithium bis(trifluoromethanesulfonimide) (7.9 mg, 0.0275 mmol) and tetrabutylammonium hexafluorophosphate (5.3 mg, 0.0138 mmol) were added to a solution of oxetanol **8** (49 mg, 0.25 mmol) and 2-hydroxyphenol (138 mg, 1.25 mmol) in chloroform (0.5 mL). The reaction mixture was stirred at 40 °C for 1 h then sat. aq. $NaHCO_3$ (15 mL) was added followed by dichloromethane (15 mL). The layers were separated and the aqueous portion was extracted with EtOAc (3 × 15 mL). The organic extracts were combined, dried over Na_2SO_4, filtered and concentrated *in vacuo*. Purification by flash column chromatography (40% EtOAc/hexane) afforded oxetane **14** (36 mg, 50%) as a light brown solid. R_f = 0.19 (40% EtOAc/hexane); mp = 176–180 °C; IR (film)/cm^{-1} 3473 (br. OH), 3187, 1737, 1610, 1532, 1499, 1433, 1363, 1269, 1231, 1200, 1175, 1116, 1033, 971, 930, 893, 806, 755; ^{1}H NMR (400 MHz, $(CD_3)_2SO$) δ 8.85 (s, 1 H, OH), 8.84 (s, 1 H, OH), 6.89–6.79 (m, 2 H, 2 × Ar-CH), 6.67 (dd, *J* = 8.1, 2.2 Hz, 2 H, 2 × Ar-CH), 6.61 (d, *J* = 2.2 Hz, 1 H, Ar-CH), 6.52 (dd, *J* = 8.1, 2.2 Hz, 1 H, Ar-CH), 5.98 (s, 2 H, OCH_2O), 5.02 (d, *J* = 5.9 Hz, 2 H, C*H*HOC*H*H), 4.98 (d, *J* = 5.9 Hz, 2 H, CH*H*OCH*H*); ^{13}C NMR (101 MHz, $(CD_3)_2SO$) δ 147.8 (Ar-C_q-OCH_2O), 145.9 (Ar-C_q-OCH_2O), 145.6 (Ar-C_q-OH), 144.2 (Ar-C_q-OH), 141.2 ($Ar_{(m/p\text{-OH})}$-C_q), 137.9 ($Ar_{(sesamol)}$-C_q), 119.7 (Ar-CH), 117.3 (Ar-CH), 115.8 (Ar-CH), 114.2 (Ar-CH), 108.4 (Ar-CH), 107.5 (Ar-CH), 101.3 (OCH_2O), 83.7 (*C*H_2O*C*H_2), 51.0 (C_q); FTMS (+ p NSI) *m/z* calcd for $C_{16}H_{18}NO_5^+$ $[M+NH_4]^+$: 304.1179, Found: 304.1179.

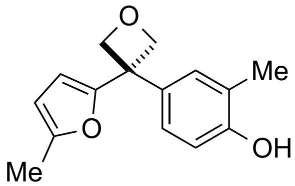

2-Methyl-4-[3-(5-methylfuran-2-yl)oxetan-3-yl]phenol (15)

Lithium bis(trifluoromethanesulfonimide) (7.9 mg, 0.0275 mmol) and tetrabutylammonium hexafluorophosphate (5.3 mg, 0.0138 mmol) were added to a solution of oxetanol **9** (39 mg, 0.25 mmol) and 2-methylphenol (135 mg, 1.25 mmol) in chloroform (0.5 mL). The reaction mixture was stirred at 40 °C for 1 h then sat. aq. $NaHCO_3$ (15 mL) was added followed by dichloromethane (15 mL). The layers were separated and the aqueous portion was extracted with dichloromethane (2 × 15 mL). The

organic extracts were combined, dried over Na_2SO_4, filtered and concentrated *in vacuo*. Purification by flash column chromatography (20% EtOAc/hexane) afforded oxetane **15** (35 mg, 58%) as a yellow gum. R_f = 0.17 (20% EtOAc/hexane); IR (film)/cm^{-1} 3307 (br. OH), 2956, 2886, 1739, 1612, 1559, 1509, 1457, 1416, 1355, 1257, 1217, 1124, 977, 949, 907, 814, 785, 729; ^{1}H NMR (400 MHz, $CDCl_3$) δ 6.99 (d, *J* = 2.4 Hz, 1 H, $Ar_{(phenol)}$-CH), 6.91 (dd, *J* = 8.1, 2.4, Hz, 1 H, $Ar_{(phenol)}$-CH), 6.75 (d, *J* = 8.1 Hz, 1 H, $Ar_{(phenol)}$-CH), 5.88 (dd, *J* = 3.1, 1.1 Hz, 1 H, $Ar_{(furan)}$-CH), 5.69 (d, *J* = 3.1 Hz, 1 H, $Ar_{(furan)}$-CH), 5.58 (s, 1 H, OH), 5.25 (d, *J* = 5.6 Hz, 2 H, C*H*HOC*H*H), 5.15 (d, J = 5.6 Hz, 2 H, CH*H*OCH*H*), 2.32 (d, *J* = 1.0 Hz, 3 H, $Ar_{(furan)}CH_3$), 2.26 (s, 3 H, $Ar_{(phenol)}CH_3$); ^{13}C NMR (101 MHz, $CDCl_3$) δ 155.7 ($Ar_{(furan)}$-*C*$_q$-C$_q$), 153.0 (Ar-C$_q$-OH), 151.9 ($Ar_{(furan)}$-*C*$_q$-CH$_3$), 134.7 ($Ar_{(phenol)}$-*C*$_q$-C$_q$), 129.1 ($Ar_{(phenol)}$-CH), 125.1 ($Ar_{(phenol)}$-CH), 124.0 ($Ar_{(phenol)}$-*C*$_q$-CH$_3$), 114.7 ($Ar_{(phenol)}$-CH), 107.6 ($Ar_{(furan)}$-CH), 106.0 ($Ar_{(furan)}$-CH), 82.2 (CH_2OCH_2), 46.9 (C$_q$), 15.9 ($Ar_{(phenol)}CH_3$), 13.6 ($Ar_{(furan)}CH_3$); FTMS (+ p NSI) m/z calcd for $C_{15}H_{16}O_3Na^+$ $[M+Na]^+$: 267.0992, Found: 267.0992.

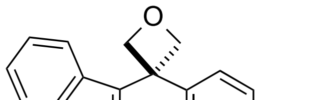

4-[3-(1-Benzyl-1H-indol-3-yl)oxetan-3-yl]phenol (16a)

Lithium bis(trifluoromethanesulfonimide) (7.9 mg, 0.0275 mmol) and tetrabutylammonium hexafluorophosphate (5.3 mg, 0.0138 mmol) were added to a solution of oxetanol **10** (70 mg, 0.25 mmol) and phenol (118 mg, 1.25 mmol) in chloroform (0.5 mL). The reaction mixture was stirred at 40 °C for 1 h then sat. aq. $NaHCO_3$ (15 mL) was added followed by dichloromethane (15 mL). The layers were separated and the aqueous portion was extracted with dichloromethane (2 × 15 mL). The organic extracts were combined, dried over Na_2SO_4, filtered and concentrated *in vacuo*. Purification by flash column chromatography (30% EtOAc/hexane) afforded oxetane **16a** (48 mg, 54%) as a light brown solid. R_f = 0.19 (30% EtOAc/hexane); mp = 180–182 °C; IR (film)/cm^{-1} 3278 (br. OH), 2957, 1738, 1611, 1509, 1480, 1467, 1449, 1351, 1268, 1232, 1175, 972, 919, 837, 741, 712, 700; ^{1}H NMR (400 MHz, $CDCl_3$) δ 7.67 (dt, *J* = 8.0, 1.0 Hz, 1 H, Ar-CH), 7.37–7.18 (m, 7 H, 7 × Ar-CH), 7.16–7.08 (m, 3 H, 3 × Ar-CH), 6.87–6.80 (m, 2 H, 2 × Ar-CH), 6.70 (s, 1 H, Ar-CH), 5.82 (s, 1 H, OH), 5.46 (d, *J* = 5.6 Hz, 2 H, C*H*HOC*H*H), 5.34 (d, *J* = 5.6 Hz, 2 H, CH*H*OCH*H*), 5.29 (s, 2 H, $PhCH_2$); ^{13}C NMR (101 MHz, $CDCl_3$) δ 154.4 (Ar-C$_q$-OH), 137.32 (Ph-C$_q$), 137.25 ($Ar_{(phenol)}$-C$_q$ and $Ar_{(indole)}$-C$_q$), 128.7 (2 × Ar-CH), 127.8 (2 × Ar-CH), 127.6 (Ar-CH), 126.6 (2 × Ar-CH), 126.5 (Ar-CH), 126.4 ($Ar_{(indole)}$-C$_q$), 122.1 (Ar-CH), 120.2 ($Ar_{(indole)}$-*C*$_q$-C$_q$), 120.1 (Ar-CH), 119.5 (Ar-CH), 115.2 (2 × Ar-CH), 110.1 (Ar-CH), 84.5 (CH_2OCH_2), 50.0 ($PhCH_2$), 46.0 (C$_q$); FTMS (+ p NSI) *m/z* calcd for $C_{24}H_{22}NO_2^+$ $[M+H]^+$: 356.1645, Found: 356.1644.

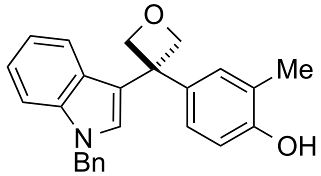

4-[3-(1-Benzyl-1H-indol-3-yl)oxetan-3-yl]-2-methylphenol (16b)

Lithium bis(trifluoromethanesulfonimide) (7.9 mg, 0.0275 mmol) and tetrabutylammonium hexafluorophosphate (5.3 mg, 0.0138 mmol) were added to a solution of oxetanol **10** (70 mg, 0.25 mmol) and 2-methylphenol (135 mg, 1.25 mmol) in chloroform (0.5 mL). The reaction mixture was stirred at 40 °C for 1 h then sat. aq. $NaHCO_3$ (15 mL) was added followed by dichloromethane (15 mL). The layers were separated and the aqueous portion was extracted with dichloromethane (2 × 15 mL). The organic extracts were combined, dried over Na_2SO_4, filtered and concentrated *in vacuo*. Purification by flash column chromatography (30% EtOAc/hexane) afforded oxetane **16b** (66 mg, 71%) as a white solid. R_f = 0.29 (30% EtOAc/hexane); mp = 189–191 °C; IR (film)/cm^{-1} 3196 (br. OH), 2961, 1738, 1612, 1513, 1465, 1451, 1376, 1354, 1334, 1268, 1237, 1194, 1177, 1125, 966, 940, 817, 768, 729, 693; ^{1}H NMR (400 MHz, $CDCl_3$) δ 7.67 (dt, *J* = 8.0, 1.0 Hz, 1 H, Ar-CH), 7.37–7.23 (m, 4 H, 4 × Ar-CH), 7.23–7.13 (m, 2 H, 2 × Ar-CH), 7.14–7.05 (m, 3 H, 3 × Ar-CH), 6.99 (dd, *J* = 8.0, 2.4 Hz, 1 H, Ar-CH), 6.74 (d, *J* = 8.0 Hz, 1 H, Ar-CH), 6.66 (s, 1 H, Ar-CH), 5.39 (d, *J* = 5.5 Hz, 2 H, C*H*HOC*H*H), 5.33–5.21 (m, 4 H, $PhCH_2$ + CH*H*OCH*H*), 4.76 (s, 1 H, OH), 2.26 (s, 3 H, CH_3); ^{13}C NMR (101 MHz, $CDCl_3$) δ 152.4 (Ar-C$_q$-OH), 137.7 ($Ar_{(phenol)}$-*C*$_q$-*C*$_q$), 137.4 (Ph-*C*$_q$), 137.3 ($Ar_{(indole)}$-C$_q$), 129.1 (Ar-CH), 128.7 (2 × Ar-CH), 127.6 (Ar-CH), 126.6 (2 × Ar-CH), 126.52 (Ar-CH), 126.50 ($Ar_{(indole)}$-C$_q$), 125.3 (Ar-CH), 123.7 (Ar-*C*$_q$-CH$_3$), 122.1 (Ar-CH), 120.5 ($Ar_{(indole)}$-*C*$_q$-C$_q$), 120.2 (Ar-CH), 119.5 (Ar-CH), 114.7 (Ar-CH), 110.0 (Ar-CH), 84.4 (*C*H$_2$O*C*H$_2$), 50.0 ($PhCH_2$), 46.1 (C$_q$), 16.0 (CH_3); FTMS (+ p NSI) *m/z* calcd for $C_{25}H_{24}NO_2^+$ $[M+H]^+$: 370.1802, Found: 370.1802.

Synthesis of Dihydrobenzofurans 3k–y, 17–21

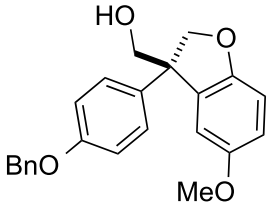

{3-[4-(Benzyloxy)phenyl]-5-methoxy-2,3-dihydro-1-benzofuran-3-yl}methanol, (3k)
Lithium bis(trifluoromethanesulfonimide) (7.9 mg, 0.0275 mmol) and tetrabutylammonium hexafluorophosphate (5.3 mg, 0.0138 mmol) were added to a solution of oxetanol **1** (64 mg, 0.25 mmol) and 4-methoxyphenol (155 mg, 1.25 mmol) in chloroform (0.5 mL). The reaction mixture was stirred at 40 °C for 20 h then sat. aq. $NaHCO_3$ (15 mL) was added followed by dichloromethane (15 mL). The layers were separated and the aqueous portion was extracted with dichloromethane (2 × 15 mL). The organic extracts were combined, washed with aq. NaOH (1 M, 15 mL) then dried over Na_2SO_4, filtered and concentrated *in vacuo*. Purification by flash column chromatography (30% EtOAc/hexane) afforded dihydrobenzofuran **3k** (66 mg, 73%) as a colorless gum. R_f = 0.18 (30% EtOAc/hexane); IR (film)/cm^{-1} 3442 (br. OH), 3034, 2941, 1608, 1510, 1486, 1466, 1381, 1242, 1199, 1179, 1025, 974, 907, 828, 804, 727, 696; ^{1}H NMR (400 MHz, $CDCl_3$) δ 7.50–7.31 (m, 5 H, 5 × Ar-CH), 7.31–7.21 (m, 2 H, 2 × Ar-CH), 7.02–6.92 (m, 2 H, 2 × Ar-CH), 6.86–6.76 (m, 3 H, 3 × Ar-CH), 5.08 (s, 2 H, $PhCH_2$), 4.75 (d, *J* = 8.8 Hz, 1 H, OC*H*HC$_q$), 4.56 (d, *J* = 8.8 Hz, 1 H, OCH*H*C$_q$), 4.13 (dd, *J* = 11.1, 3.6 Hz, 1 H, C*H*HOH), 4.05 (dd, *J* = 11.1, 7.0 Hz, 1 H, CH*H*OH), 3.79 (s, 3 H, CH_3); ^{13}C NMR (101 MHz, $CDCl_3$) δ 157.8 (Ar-C$_q$-OBn), 154.8 (Ar$_{(DHBF)}$-*C$_q$*-OCH$_2$), 154.3 (Ar-*C$_q$*-OCH$_3$), 136.9 (Ph-C$_q$), 134.7 (Ar$_{(p\text{-}OBn)}$-*C$_q$*-C$_q$), 131.2 (Ar$_{(DHBF)}$-*C$_q$*-C$_q$), 128.6 (2 × Ar-CH), 128.1 (2 × Ar-CH), 128.0 (Ar-CH), 127.4 (2 × Ar-CH), 115.1 (2 × Ar-CH), 114.2 (Ar-CH), 111.2 (Ar-CH), 110.1 (Ar-CH), 81.5 (O*C*H$_2$C$_q$), 70.0 ($PhCH_2$), 67.4 (CH_2OH), 56.1 (C$_q$), 56.0 (CH_3); FTMS (+ p NSI) *m/z* calcd for $C_{23}H_{26}O_4N^+$ $[M+NH_4]^+$: 380.1856, Found: 380.1857.

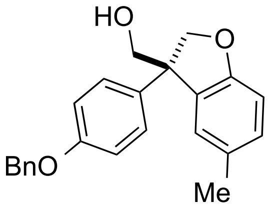

{3-[4-(Benzyloxy)phenyl]-5-methyl-2,3-dihydro-1-benzofuran-3-yl}methanol (3l)
Lithium bis(trifluoromethanesulfonimide) (7.9 mg, 0.0275 mmol) and tetrabutylammonium hexafluorophosphate (5.3 mg, 0.0138 mmol) were added to a solution of oxetanol **1** (64 mg, 0.25 mmol) and 4-methylphenol (135 mg, 1.25 mmol) in chloroform (0.5 mL). The reaction mixture was stirred at 40 °C for 20 h then sat. aq. $NaHCO_3$ (15 mL) was added followed by dichloromethane (15 mL). The layers were separated and the aqueous portion was extracted with dichloromethane (2 × 15 mL). The organic extracts were combined, washed with aq. NaOH (1 M, 15 mL) then dried over Na_2SO_4, filtered and concentrated *in vacuo*. Purification by flash column chromatography (30% EtOAc/hexane) afforded dihydrobenzofuran **3l** (61 mg, 81%) as a colorless gum. R_f = 0.33 (30% EtOAc/hexane); IR (film)/cm^{-1} 3420 (br. OH), 3034, 2868, 1608, 1580, 1489, 1454, 1381, 1295, 1241, 1182, 1127, 1037, 1024, 971, 827, 809, 732, 696; ^{1}H NMR (400 MHz, $CDCl_3$) δ 7.52–7.32 (m, 5 H, 5 × Ar-CH), 7.29–7.21 (m, 2 H, 2 × Ar-CH), 7.10–6.94 (m, 4 H, 4 × Ar-CH), 6.81 (d, *J* = 8.1 Hz, 1 H, 1 × Ar-CH), 5.09 (s, 2 H, $PhCH_2$), 4.77 (d, *J* = 8.8 Hz, 1 H, OC*H*HC$_q$), 4.58 (d, *J* = 8.8 Hz, 1 H, OCH*H*C$_q$), 4.12 (dd, *J* = 11.1, 3.9 Hz, 1 H, C*H*HOH), 4.04 (dd, *J* = 11.1, 7.5 Hz, 1 H, CH*H*OH), 2.35 (s, 3 H, CH_3), 1.74 (s, 1 H, OH); ^{13}C NMR (101 MHz, $CDCl_3$) δ 158.6 (Ar$_{(DHBF)}$-*C$_q$*-OCH$_2$), 157.6 (Ar-C$_q$-OBn), 136.8 (Ph-C$_q$), 135.0 (Ar-*C$_q$*-C$_q$), 130.1 (Ar$_{(DHBF)}$-*C$_q$*-C$_q$), 130.0 (Ar*C$_q$*-CH$_3$), 129.6 (Ar-CH), 128.5 (2 × Ar-CH), 128.0 (2 × Ar-CH), 127.9 (Ar-CH), 127.4 (2 × Ar-CH), 125.5 (Ar-CH), 115.0 (2 × Ar-CH), 109.7 (Ar-CH), 81.3 (O*C*H$_2$C$_q$), 69.9 ($PhCH_2$), 67.5 (CH_2OH), 55.7 (C$_q$), 20.9 (CH_3); FTMS (+ p NSI) m/z calcd for $C_{23}H_{26}O_3N^+$ $[M+NH_4]^+$: 364.1907, Found: 364.1909.

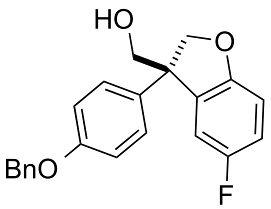

{3-[4-(Benzyloxy)phenyl]-5-fluoro-2,3-dihydro-1-benzofuran-3-yl}methanol (3m)
Lithium bis(trifluoromethanesulfonimide) (7.9 mg, 0.0275 mmol) and tetrabutylammonium hexafluorophosphate (5.3 mg, 0.0138 mmol) were added to a solution of oxetanol **1** (64 mg, 0.25 mmol) and 4-fluorophenol (140 mg, 1.25 mmol) in chloroform (0.5 mL). The reaction mixture was stirred at 40 °C for 20 h then sat. aq. $NaHCO_3$ (15 mL) was added followed by dichloromethane (15 mL). The layers were separated and the aqueous portion was extracted with dichloromethane (2 × 15 mL). The organic extracts were combined, washed with aq. NaOH (1 M, 15 mL) then dried over Na_2SO_4, filtered and concentrated *in vacuo*. Purification by flash column chromatography (25%

EtOAc/hexane) afforded dihydrobenzofuran **3m** (25 mg, 29%) as a colorless gum. R_f = 0.11 (20% EtOAc/hexane); mp = 92–94 °C; IR (film)/cm^{-1} 3439 (br. OH), 2875, 1609, 1501, 1511, 1485, 1382, 1296, 1246, 1183, 1025, 978, 863, 828, 813, 775, 738, 697; ^{1}H NMR (400 MHz, $CDCl_3$) δ 7.47–7.29 (m, 5 H, 5 × Ar-CH), 7.26–7.19 (m, 2 H, 2 × Ar-CH), 7.01–6.87 (m, 4 H, 4 × Ar-CH), 6.83–6.75 (m, 1 H, 1 × Ar-CH), 5.07 (s, 2 H, $PhCH_2$), 4.76 (d, *J* = 9.0 Hz, 1 H, OC*H*HC$_q$), 4.58 (d, *J* = 9.0 Hz, 1 H, OCH*H*C$_q$), 4.12 (dd, *J* = 11.1, 4.9 Hz, 1 H, C*H*HOH), 4.03 (dd, *J* = 11.1, 7.6 Hz, 1 H, CH*H*OH); ^{13}C NMR (101 MHz, $CDCl_3$) δ 157.9 (Ar-C$_q$-OBn), 157.5 (d, $J_{C\text{-}F}$ = 240.5 Hz, Ar-*C*$_q$-F), 156.5 (Ar$_{(DHBF)}$-*C*$_q$-OCH$_2$), 136.8 (Ph-C$_q$), 134.2 (Ar$_{(p\text{-}OBn)}$-*C*$_q$-C$_q$), 131.8 (d, $J_{C\text{-}F}$ = 7.8 Hz, Ar$_{(DHBF)}$-*C*$_q$-C$_q$), 128.6 (2 × Ar-CH), 128.01 (2 × Ar-CH), 127.97 (Ar-CH), 127.4 (2 × Ar-CH), 115.5 (d, $J_{C\text{-}F}$ = 23.9 Hz, Ar-CH), 115.2 (2 × Ar-CH), 112.4 (d, $J_{C\text{-}F}$ = 25.4 Hz, Ar-CH), 110.3 (d, $J_{C\text{-}F}$ = 8.2 Hz, Ar-CH), 81.8 (O*C*H$_2$C$_q$), 70.0 ($PhCH_2$), 67.5 (CH_2OH), 56.0 (C$_q$);^{19}F NMR (377 MHz, $CDCl_3$) δ –123.5; FTMS (+ p NSI) *m/z* calcd for $C_{22}H_{23}O_3NF^+$ $[M+NH_4]^+$: 368.1656, Found: 368.1659.

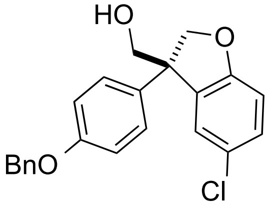

{3-[4-(Benzyloxy)phenyl]-5-chloro-2,3-dihydro-1-benzofuran-3-yl}methanol (3n) Lithium bis(trifluoromethanesulfonimide) (7.9 mg, 0.0275 mmol) and tetrabutylammonium hexafluorophosphate (5.3 mg, 0.0138 mmol) were added to a solution of oxetanol **1** (64 mg, 0.25 mmol) and 4-chlorophenol (161 mg, 1.25 mmol) in chloroform (0.5 mL). The reaction mixture was stirred at 40 °C for 20 h then sat. aq. $NaHCO_3$ (15 mL) was added followed by dichloromethane (15 mL). The layers were separated and the aqueous portion was extracted with dichloromethane (2 × 15 mL). The organic extracts were combined, washed with aq. NaOH (1 M, 15 mL) then dried over Na_2SO_4, filtered and concentrated *in vacuo*. Purification by flash column chromatography (20% EtOAc/hexane) afforded dihydrobenzofuran **3n** (26 mg, 28%) as a colorless gum. R_f = 0.18 (20% EtOAc/hexane); IR (film)/cm^{-1} 3420 (br. OH), 2880, 1609, 1512, 1479, 1381, 1297, 1244, 1183, 1025, 975, 827, 736, 696; ^{1}H NMR (400 MHz, $CDCl_3$) δ 7.48–7.30 (m, 5 H, 5 × Ar-CH), 7.27–7.13 (m, 4 H, 4 × Ar-CH), 7.02–6.94 (m, 2 H, 2 × Ar-CH), 6.86–6.79 (m, 1 H, Ar-CH), 5.07 (s, 2 H, $PhCH_2$), 4.77 (d, *J* = 9.0 Hz, 1 H, OC*H*HC$_q$), 4.59 (d, *J* = 9.0 Hz, 1 H, OCH*H*C$_q$), 4.11 (dd, *J* = 11.2, 4.6 Hz, 1 H, CHHOH), 4.03 (dd, *J* = 11.2, 7.1 Hz, 1 H, CHHOH); ^{13}C NMR (101 MHz, $CDCl_3$) δ 159.3 (Ar$_{(DHBF)}$-*C*$_q$-OCH$_2$), 157.9 (Ar$_{(p\text{-}OBn)}$-*C*$_q$-C$_q$), 136.8 (Ph-*C*$_q$-C$_q$), 134.1 (Ar-*C*$_q$-C$_q$), 132.4 (Ar-*C*$_q$-C$_q$), 129.1 (Ar-CH), 128.6 (2 × Ar-CH), 128.1 (Ar-CH), 128.0 (2 × Ar-CH), 127.5 (2 × Ar-CH), 125.5 (Ar-C-Cl), 125.4 (Ar-CH), 115.2 (2 × Ar-CH), 111.2 (Ar-CH), 81.9 (O*C*H$_2$C$_q$), 70.1 ($PhCH_2$), 67.5 (CH_2OH), 55.9 (C$_q$); FTMS (+ p NSI) m/z calcd for $C_{22}H_{23}ClO_3N^+$ $[M+NH_4]^+$: 384.1361, Found: 384.1356.

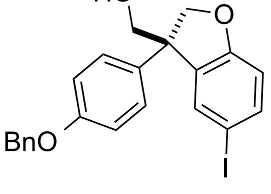

{3-[4-(Benzyloxy)phenyl]-5-iodo-2,3-dihydro-1-benzofuran-3-yl}methanol (3o) Lithium bis(trifluoromethanesulfonimide) (7.9 mg, 0.0275 mmol) and tetrabutylammonium hexafluorophosphate (5.3 mg, 0.0138 mmol) were added to a solution of oxetanol **1** (64 mg, 0.25 mmol) and 4-iodophenol (275 mg, 1.25 mmol) in chloroform (0.5 mL). The reaction mixture was stirred at 40 °C for 20 h then sat. aq. $NaHCO_3$ (15 mL) was added followed by dichloromethane (15 mL). The layers were separated and the aqueous portion was extracted with dichloromethane (2 × 15 mL). The organic extracts were combined, washed with aq. NaOH (1 M, 15 mL) then dried over Na_2SO_4, filtered and concentrated *in vacuo*. Purification by flash column chromatography (30% EtOAc/pentane) afforded dihydrobenzofuran **3o** (27 mg, 25%) as a colorless gum. R_f = 0.26 (20% EtOAc/hexane); IR (film)/cm^{-1} 3429 (br. OH), 3032, 2880, 1609, 1582, 1476, 1456, 1382, 1293, 1238, 1183, 1130, 1025, 971, 909, 827, 811, 734, 697, 669; ^{1}H NMR (400 MHz, $CDCl_3$) δ 7.55–7.30 (m, 7 H, 7 × Ar-CH), 7.24–7.15 (m, 2 H, 2 × Ar-CH), 7.02–6.92 (m, 2 H, 2 × Ar-CH), 6.73–6.62 (m, 1 H, Ar-CH), 5.07 (s, 2 H, $PhCH_2$), 4.75 (d, *J* = 9.0 Hz, 1 H, OC*H*HC$_q$), 4.57 (d, *J* = 9.0 Hz, 1 H, OCH*H*C$_q$), 4.09 (d, *J* = 11.1 Hz, 1 H, C*H*HOH), 4.02 (d, *J* = 11.1 Hz, 1 H, CH*H*OH); ^{13}C NMR (101 MHz, $CDCl_3$) δ 160.6 (Ar$_{(DHBF)}$-*C*$_q$-OCH$_2$), 157.9 (Ar-C$_q$-OBn), 137.9 (Ar-CH), 136.8 (Ph-C$_q$), 134.1 (Ar-*C*$_q$-C$_q$), 133.9 (Ar-CH), 133.6 (Ar-*C*$_q$-C$_q$), 128.6 (2 × Ar-CH), 128.01 (Ar-CH), 127.97 (2 × Ar-CH), 127.4 (2 × Ar-CH), 115.2 (2 × Ar-CH), 112.5 (Ar-CH), 82.1 (Ar-C$_q$-I), 81.7 (O*C*H$_2$C$_q$), 70.0 ($PhCH_2$), 67.5 (CH_2OH), 55.7 (C$_q$); FTMS (+ p NSI) *m/z* calcd for $C_{22}H_{23}O_3NI^+$ $[M+NH_4]^+$: 476.0717, Found: 476.0709.

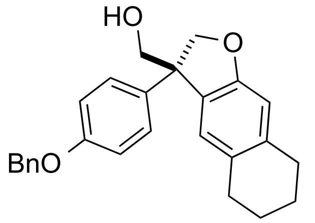

{3-[4-(Benzyloxy)phenyl]-2H,3H,5H,6H,7H,8H-naphtho[2,3-b]furan-3-yl}methanol (3p)

Lithium bis(trifluoromethanesulfonimide) (7.9 mg, 0.0275 mmol) and tetrabutylammonium hexafluorophosphate (5.3 mg, 0.0138 mmol) were added to a solution of oxetanol **1** (64 mg, 0.25 mmol) and 5,6,7,8-tetrahydro-2-naphthol (185 mg, 1.25 mmol) in chloroform (0.5 mL). The reaction mixture was stirred at 40 °C for 1 h then sat. aq. $NaHCO_3$ (15 mL) was added followed by dichloromethane (15 mL). The layers were separated and the aqueous portion was extracted with dichloromethane (2 × 15 mL). The organic extracts were combined, dried over Na_2SO_4, filtered and concentrated *in vacuo*. Purification by flash column chromatography (20% EtOAc/hexane) afforded dihydrobenzofuran **3p** (88 mg, 91%) as a yellow gum. R_f = 0.17 (20% EtOAc/hexane); IR (film)/cm^{-1} 3469 (br. OH), 2930, 1739, 1609, 1510, 1487, 1242, 1182, 1024, 975, 905, 855, 828, 726, 696; ^{1}H NMR (400 MHz, $CHCl_3$) δ 7.51–7.33 (m, 5 H, 5 × Ar-CH), 7.32–7.24 (m, 2 H, 2 × Ar-CH), 7.04–6.93 (m, 2 H, 2 × Ar-CH), 6.91 (s, 1 H, Ar-CH), 6.64 (s, 1 H, Ar-CH), 5.09 (s, 2 H, $PhCH_2$), 4.74 (d, *J* = 8.8 Hz, 1 H, OC*H*HC_q), 4.56 (d, *J* = 8.8 Hz, 1 H, OCH*H*C_q), 4.10 (dd, *J* = 11.1, 4.8 Hz, 1 H, C*H*HOH), 4.02 (dd, *J* = 11.1, 7.5 Hz, 1 H, CH*H*OH), 2.86–2.77 (m, 2 H, ArC*H*$_2$$CH_2$), 2.77–2.66 (m, 2 H, ArC*H*$_2$$CH_2$), 1.88–1.76 (m, 4 H, 2 × ArCH_2C*H*$_2$), 1.75–1.64 (m, 1 H, OH); ^{13}C NMR (101 MHz, $CHCl_3$) δ 158.7 (Ar$_{(DHBF)}$-C_q-OCH_2), 157.7 (Ar-C_q-OBn), 138.3 (Ar-C_q-CH_2CH_2), 136.9 (Ph-C_q), 135.1 (Ar$_{(p\text{-OBn})}$-C_q-C_q), 129.4 (Ar-C_q-CH_2CH_2),128.6 (2 × Ar-CH), 128.1 (2 × Ar-CH), 128.0 (Ar-CH), 127.7 (Ar$_{(DHBF)}$-C_q-C_q), 127.4 (2 × Ar-CH), 125.3 (Ar-CH), 114.9 (2 × Ar-CH), 110.0 (Ar-CH), 81.2 (O*C*H_2C_q), 70.0 ($PhCH_2$), 67.7 (CH_2OH), 55.6 (C_q), 29.9 (Ar*C*H_2CH_2), 29.2 (Ar*C*H_2CH_2), 23.4 ($ArCH_2$*C*H_2), 23.1 ($ArCH_2$*C*H_2); FTMS (+ p NSI) *m/z* calcd for $C_{26}H_{30}O_3N^+$ $[M+NH_4]^+$: 404.2220, Found: 404.2217.

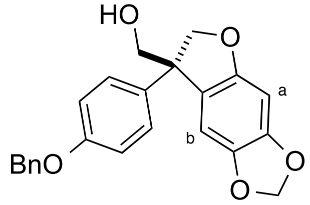

{12-[4-(Benzyloxy)phenyl]-4,6,10-trioxatricyclo[7.3.0.0^{3,7}]dodeca-1(9),2,7-trien-12-yl}methanol (3q)

Lithium bis(trifluoromethanesulfonimide) (7.9 mg, 0.0275 mmol) and tetrabutylammonium hexafluorophosphate (5.3 mg, 0.0138 mmol) were added to a solution of oxetanol **1** (64 mg, 0.25 mmol) and sesamol (173 mg, 1.25 mmol) in chloroform (0.5 mL). The reaction mixture was stirred at 40 °C for 1 h then sat. aq. $NaHCO_3$ (15 mL) was added followed by dichloromethane (15 mL). The layers were separated and the aqueous portion was extracted with dichloromethane (2 × 15 mL). The organic extracts were combined, dried over Na_2SO_4, filtered and concentrated *in vacuo*. Purification by flash column chromatography (20% EtOAc/hexane) afforded oxetane **3q** (89 mg, 94%) as a white solid. R_f = 0.06 (20% EtOAc/hexane); mp = 110–111 °C; IR (film)/cm^{-1} 3441 (br. OH), 2885, 1609, 1580, 1510, 1474, 1452, 1382, 1291, 1244, 1182, 1151, 1034, 1012, 938, 861, 828, 769, 735, 698; ^{1}H NMR (400 MHz, $CHCl_3$) δ 7.48–7.29 (m, 5 H, 5 × Ar-CH), 7.24–7.16 (m, 2 H, 2 × Ar-CH), 6.98–6.92 (m, 2 H, 2 × Ar-CH), 6.65 (s, 1 H, Ar-CH_a), 6.47 (s, 1 H, Ar-CH_b), 5.95 (dd, *J* = 7.8, 1.3 Hz, 2 H, OCH_2O), 5.06 (s, 2 H, $PhCH_2$), 4.73 (d, *J* = 8.9 Hz, 1 H, OC*H*HC_q), 4.54 (d, *J* = 8.9 Hz, 1 H, OCH*H*C_q), 4.06 (dd, *J* = 11.1, 4.5 Hz, 1 H, C*H*HOH), 3.99 (dd, *J* = 11.1, 7.8 Hz, 1 H, CH*H*OH), 1.61 (dd, *J* = 7.8, 4.5 Hz, 1 H, OH); ^{13}C NMR (101 MHz, $CHCl_3$) δ 157.8 (Ar-C_q-OBn), 155.6 (Ar$_{(DHBF)}$-C_q-OCH_2C_q), 148.3 (Ar$_{(DHBF)}$-C_q-OCH_2O), 141.8 (Ar$_{(DHBF)}$-C_q-OCH_2O), 136.9 (Ph-C_q), 135.1 (Ar$_{(p\text{-OBn})}$-C_q-C_q), 128.6 (2 × Ar-CH), 128.0 (3 × Ar-CH), 127.5 (2 × Ar-CH), 121.0 (Ar$_{(DHBF)}$-C_q-C_q), 115.1 (2 × Ar-CH), 105.0 (Ar-CH_a), 101.4 (OCH_2O), 93.5 (Ar-CH_b), 82.0 (O*C*H_2C_q), 70.0 ($PhCH_2$), 67.5 (CH_2OH), 55.7 (C_q); FTMS (+ p NSI) *m/z* calcd for $C_{23}H_{21}O_5^+$ $[M+H]^+$: 377.1384, Found: 377.1380.

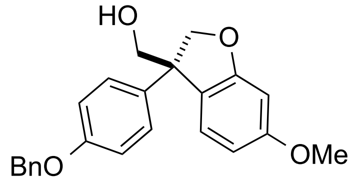

{3-[4-(Benzyloxy)phenyl]-6-methoxy-2,3-dihydro-1-benzofuran-3-yl}methanol (3r)

Lithium bis(trifluoromethanesulfonimide) (7.9 mg, 0.0275 mmol) and tetrabutylammonium hexafluorophosphate (5.3 mg, 0.0138 mmol) were added to a solution of oxetanol **1** (64 mg, 0.25 mmol) and 3-methoxyphenol (0.14 mL, 1.25 mmol) in chloroform (0.5 mL). The reaction mixture was stirred at 40 °C for 1 h then sat. aq. $NaHCO_3$ (15 mL) was added followed by dichloromethane (15 mL). The layers were separated and the aqueous portion was extracted with dichloromethane (2 × 15 mL). The organic extracts were combined, dried over Na_2SO_4, filtered and concentrated *in vacuo*. Purification by flash column

chromatography (40% EtOAc/hexane) afforded oxetane **3r** (41 mg, 46%) as a pale yellow gum. R_f = 0.18 (40% EtOAc/hexane); IR (film)/cm^{-1} 3451 (br. OH), 2943, 2836, 1739, 1619, 1595, 1510, 1496, 1466, 1454, 1444, 1379, 1332, 1278, 1241, 1183, 1147, 1085, 1024, 980, 910, 827, 697; ^{1}H NMR (400 MHz, $CHCl_3$) δ 7.47–7.30 (m, 5 H, 5 × Ar-CH), 7.26–7.18 (m, 2 H, 2 × Ar-CH), 7.11–7.05 (m, 1 H, Ar-CH), 6.99–6.91 (m, 2 H, 2 × Ar-CH), 6.52 (dd, *J* = 8.2, 2.2 Hz, 1 H, Ar-CH), 6.49 (d, *J* = 2.2 Hz, 1 H, Ar-CH), 5.06 (s, 2 H, $PhCH_2$), 4.79 (d, *J* = 8.8 Hz, 1 H, OC*H*HC$_q$), 4.58 (d, *J* = 8.8 Hz, 1 H, OCH*H*C$_q$), 4.06 (d, *J* = 11.1, 1 H, C*H*HOH), 3.99 (d, *J* = 11.1 Hz, 1 H, CH*H*OH), 3.81 (s, 3 H, CH_3); ^{13}C NMR (101 MHz, $CHCl_3$) δ 162.1 (Ar$_{(DHBF)}$-*C$_q$*-OCH_2), 161.2 (Ar$_{(DHBF)}$-*C$_q$*-OCH_3), 157.7 (Ar-C$_q$-OBn), 136.9 (Ph-C$_q$), 135.2 (Ar$_{(p\text{-}OBn)}$-*C$_q$*-C$_q$), 128.6 (2 × Ar-CH), 128.0 (3 × Ar-CH), 127.4 (2 × Ar-CH), 125.3 (Ar-CH), 121.9 (Ar$_{(DHBF)}$-*C$_q$*-C$_q$), 115.0 (2 × Ar-CH), 106.7 (Ar-CH), 96.5 (Ar-CH), 82.0 (O*C*H$_2$C$_q$), 70.0 ($PhCH_2$), 67.8 (CH_2OH), 55.5 (CH_3), 55.2 (C$_q$); FTMS (+ p NSI) *m/z* calcd for $C_{23}H_{23}O_4^+$ $[M+H]^+$: 363.1591, Found: 363.1591.

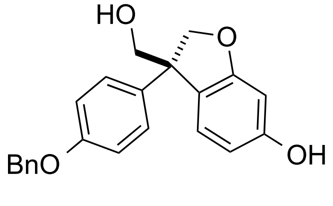

3-[4-(Benzyloxy)phenyl]-3-(hydroxymethyl)-2,3-dihydro-1-benzofuran-6-ol (3s)

Lithium bis(trifluoromethanesulfonimide) (7.9 mg, 0.0275 mmol) and tetrabutylammonium hexafluorophosphate (5.3 mg, 0.0138 mmol) were added to a solution of oxetanol **1** (64 mg, 0.25 mmol) and resourcinol (138 mg, 1.25 mmol) in chloroform (0.5 mL). The reaction mixture was stirred at 40 °C for 1 h then sat. aq. $NaHCO_3$ (15 mL) was added followed by dichloromethane (15 mL). The layers were separated and the aqueous portion was extracted with dichloromethane (2 × 15 mL). The organic extracts were combined, dried over Na_2SO_4, filtered and concentrated *in vacuo*. Purification by flash column chromatography (35% EtOAc/hexane) afforded oxetane **3s** (64 mg, 74%) as a white solid. R_f = 0.09 (35% EtOAc/hexane); mp = 185–187 °C; IR (film)/cm^{-1} 3356 (br. OH) 3045, 2874, 1611, 1514, 1479, 1453, 1386, 1286, 1243, 1173, 1189, 1152, 1121, 1094, 1080, 1039, 1014, 1005, 987, 916, 834, 810, 748, 698; ^{1}H NMR (400 MHz, $(CD_3)_2SO$) δ 9.34 (s, 1 H, Ar-OH), 7.52–7.28 (m, 5 H, 5 × Ar-CH), 7.27–7.19 (m, 2 H, 2 × Ar-CH), 6.99–6.87 (m, 3 H, 3 × Ar-CH), 6.35–6.25 (m, 1 H, Ar-CH), 6.25–6.19 (m, 1 H, Ar-CH), 5.07 (s, 2 H, $PhCH_2$), 5.03 (m, 1 H, CH_2O*H*) 4.68 (d, *J* = 8.9 Hz, 1 H, OC*H*HC$_q$), 4.46 (d, *J* = 8.9 Hz, 1 H, OCH*H*C$_q$), 3.84 (dd, *J* = 10.5, 5.0 Hz, 1 H, C*H*HOH), 3.75 (dd, *J* = 10.5, 5.0 Hz, 1 H, CH*H*OH); ^{13}C NMR (101 MHz, $(CD_3)_2SO$) δ 161.5 (Ar$_{(DHBF)}$-*C$_q$*-OCH_2), 158.6 (Ar$_{(DHBF)}$-*C$_q$*-OH), 157.2, (Ar-C$_q$-OBn), 137.7 (Ph-C$_q$), 137.2 (Ar$_{(p\text{-}OBn)}$-*C$_q$*-C$_q$), 128.9 (2 × Ar-CH), 128.6 (2 × Ar-CH), 128.2 (Ar-CH), 128.1 (2 × Ar-CH), 125.8 (Ar-CH), 122.6 (Ar$_{(DHBF)}$-*C$_q$*-C$_q$), 114.9 (2 × Ar-CH), 107.7 (Ar-CH), 97.6 (Ar-CH), 81.5 (O*C*H$_2$C$_q$), 69.6 ($PhCH_2$), 66.8 (CH_2OH), 54.9 (C$_q$); FTMS (+ p NSI) *m/z* calcd for $C_{22}H_{21}O_4^+$ $[M+H]^+$: 349.1434, Found: 349.1436.

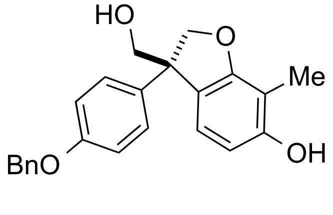

3-[4-(Benzyloxy)phenyl]-3-(hydroxymethyl)-7-methyl-2,3-dihydro-1-benzo furan-6-ol (3t)

Lithium bis(trifluoromethanesulfonimide) (7.9 mg, 0.0275 mmol) or calcium bis(trifluoromethanesulfonimide) (8.3 mg, 0.0138 mmol) and tetrabutylammonium hexafluorophosphate (5.3 mg, 0.0138 mmol) were added to a solution of oxetanol **1** (64 mg, 0.25 mmol) and 2-methylresourcinol (155 mg, 1.25 mmol) in chloroform (0.5 mL). The reaction mixture was stirred at 40 °C for 1 h then sat. aq. $NaHCO_3$ (15 mL) was added followed by dichloromethane (15 mL). The layers were separated and the aqueous portion was extracted with dichloromethane (2 × 15 mL). The organic extracts were combined, dried over Na_2SO_4, filtered and concentrated *in vacuo*. Purification by flash column chromatography (30% EtOAc/hexane) afforded dihydrobenzofuran **3t** (61 mg, 67%) as a pale orange solid. R_f = 0.15 (30% EtOAc/hexane); mp = 150–152 °C; IR (film)/cm^{-1} 3467 (br. OH), 1738, 1597, 1511, 1468, 1454, 1431, 1383, 1241, 1217, 1183, 1137, 1124, 1078, 1056, 1015, 983, 840, 816, 799, 745, 696; ^{1}H NMR (400 MHz, $(CD_3)_2SO$) δ 9.20 (s, 1 H, Ar-OH), 7.49–7.27 (m, 5 H, 5 × Ar-CH), 7.26–7.17 (m, 2 H, 2 × Ar-CH), 6.97–6.86 (m, 2 H, 2 × Ar-CH), 6.75 (d, *J* = 8.0 Hz, 1 H, Ar-CH), 6.34 (d, *J* = 8.0 Hz, 1 H, Ar-CH), 5.06 (s, 2 H, $PhCH_2$), 4.99 (t, *J* = 5.1 Hz, 1 H, CH_2O*H*), 4.67 (d, *J* = 8.9 Hz, 1 H, OC*H*HC$_q$), 4.47 (d, *J* = 8.9 Hz, 1 H, OCH*H*C$_q$), 3.84 (dd, *J* = 10.7, 5.1 Hz, 1 H, C*H*HOH), 3.74 (dd, *J* = 10.7, 5.1 Hz, 1 H, CH*H*OH), 1.95 (s, 3 H, CH_3); ^{13}C NMR (101 MHz, $(CD_3)_2SO$) δ 159.8 (Ar$_{(DHBF)}$-*C$_q$*-OCH_2), 157.1 (Ar-C$_q$-OBn), 156.3 (Ar$_{(DHBF)}$-C$_q$-OH), 137.7 (Ph-C$_q$), 137.3 (Ar$_{(p\text{-}OBn)}$-*C$_q$*-C$_q$), 128.8 (2 × Ar-CH), 128.6 (2 × Ar-CH), 128.2 (Ar-CH), 128.0 (2 × Ar-CH),

122.2 (Ar-CH), 122.0 (Ar$_{(DHBF)}$-*C$_q$*-C$_q$), 114.8 (2 × Ar-CH), 107.3 (Ar-CH), 106.2 (Ar-*C$_q$*-CH$_3$), 81.2 (O*C*H$_2$C$_q$), 69.6 (PhCH$_2$), 66.9 (CH$_2$OH), 55.4 (C$_q$), 9.1 (CH$_3$); FTMS (+ p NSI) *m/z* calcd for $C_{23}H_{23}O_4^+$ [M+H]$^+$: 363.1591, Found: 363.1581.

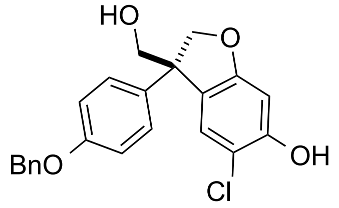

3-[4-(Benzyloxy)phenyl]-5-chloro-3-(hydroxymethyl)-2,3-dihydro-1-benzofuran-6-ol (3u)

Lithium bis(trifluoromethanesulfonimide) (7.9 mg, 0.0275 mmol) and tetrabutylammonium hexafluorophosphate (5.3 mg, 0.0138 mmol) were added to a solution of oxetanol **1** (64 mg, 0.25 mmol) and 4-chlororesourcinol (181 mg, 1.25 mmol) in chloroform (0.5 mL). The reaction mixture was stirred at 40 °C for 20 h then sat. aq. $NaHCO_3$ (15 mL) was added followed by dichloromethane (15 mL). The layers were separated and the aqueous portion was extracted with dichloromethane (2 × 15 mL). The organic extracts were combined, dried over Na_2SO_4, filtered and concentrated *in vacuo*. Purification by flash column chromatography (30% EtOAc/hexane) afforded dihydrobenzofuran **3u** (48 mg, 50%) as a pale brown solid. R_f = 0.10 (30% EtOAc/hexane); mp = 140–141 °C; IR (film)/cm^{-1} 3427 (br. OH), 2946, 2901, 1739, 1600, 1583, 1510, 1490, 1468, 1456, 1428, 1378, 1286, 1245, 1223, 1185, 1139, 1091, 1041, 1012, 987, 873, 837, 801, 765, 747, 727, 695, 684; ^{1}H NMR (400 MHz, (CD$_3$)$_2$SO) δ 10.09 (s, 1 H, Ar-OH), 7.48–7.27 (m, 5 H, 5 × Ar-CH), 7.26–7.16 (m, 2 H, 2 × Ar-CH), 7.08 (s, 1 H, Ar-CH), 7.00–6.90 (m, 2 H, 2 × Ar-CH), 6.44 (s, 1 H, Ar-CH), 5.09 (t, *J* = 5.0 Hz, 1 H, CH$_2$O*H*), 5.08 (s, 2 H, PhCH$_2$), 4.69 (d, *J* = 8.9 Hz, 1 H, OC*H*HC$_q$), 4.50 (d, *J* = 8.9 Hz, 1 H OCH*H*C$_q$), 3.84 (dd, *J* = 10.9, 5.0 Hz, 1 H, C*H*HOH), 3.80 (dd, *J* = 10.9, 5.0 Hz, 1 H, CH*H*OH); ^{13}C NMR (101 MHz, (CD$_3$)$_2$SO) δ 160.0 (Ar$_{(DHBF)}$-*C$_q$*-OCH$_2$), 157.3 (Ar-C$_q$-OBn), 153.7 (Ar$_{(DHBF)}$-*C$_q$*-OH), 137.6 (Ph-C$_q$), 136.5 (Ar$_{(p\text{-}OBn)}$-*C$_q$*-C$_q$), 128.9 (2 × Ar-CH), 128.4 (2 × Ar-CH), 128.3 (Ar-CH), 128.1 (2 × Ar-CH), 126.2 (Ar-CH), 124.1 (Ar$_{(DHBF)}$-*C$_q$*-C$_q$), 115.0 (2 × Ar-CH), 111.3 (Ar-C$_q$-Cl), 98.6 (Ar-CH), 81.8 (O*C*H$_2$C$_q$), 69.6 (PhCH$_2$), 66.4 (CH$_2$OH), 55.3 (C$_q$); FTMS (+ p NSI) *m/z* calcd for $C_{22}H_{23}O_4NCl^+$ [M+NH$_4$]$^+$: 400.1310, Found: 400.1300.

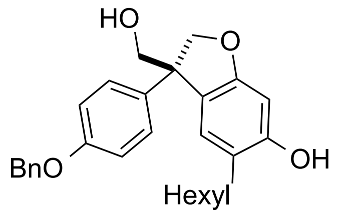

3-[4-(Benzyloxy)phenyl]-5-hexyl-3-(hydroxymethyl)-2,3-dihydro-1-benzofuran-6-ol (3v)

Lithium bis(trifluoromethanesulfonimide) (7.9 mg, 0.0275 mmol) and tetrabutylammonium hexafluorophosphate (5.3 mg, 0.0138 mmol) were added to a solution of oxetanol **1** (64 mg, 0.25 mmol) and 4-hexylresourcinol (243 mg, 1.25 mmol) in chloroform (0.5 mL). The reaction mixture was stirred at 40 °C for 20 h then sat. aq. $NaHCO_3$ (15 mL) was added followed by dichloromethane (15 mL). The layers were separated and the aqueous portion was extracted with dichloromethane (2 × 15 mL). The organic extracts were combined, dried over Na_2SO_4, filtered and concentrated *in vacuo*. Purification by flash column chromatography (25% EtOAc/hexane) afforded dihydrobenzofuran **3v** (60 mg, 55%) as a dark red gum. R_f = 0.18 (30% EtOAc/hexane); IR (film)/cm^{-1} 3400 (br. OH), 2927, 2858, 1739, 1610, 1510, 1497, 1455, 1379, 1296, 1243, 1180, 1023, 905, 829, 726, 697; ^{1}H NMR (400 MHz, CHCl$_3$) δ 7.54–7.29 (m, 5 H, 5 × Ar-CH), 7.30–7.14 (m, 2 H, 2 × Ar-CH), 7.02–6.85 (m, 3 H, 3 × Ar-CH), 6.35 (s, 1 H, Ar-CH), 5.26 (s, 1 H, Ar-OH), 5.06 (s, 2 H, PhCH$_2$), 4.74 (d, *J* = 8.8 Hz, 1 H, OC*H*HC$_q$), 4.53 (d, *J* = 8.8 Hz, 1 H, OCH*H*C$_q$), 4.06 (d, *J* = 11.1 Hz, 1 H, C*H*HOH), 4.00 (d, *J* = 11.1 Hz, 1 H, CH*H*OH), 2.54 (sep, *J* = 7.2 Hz, 2 H, CH$_2$), 1.56 (quin, *J* = 7.2 Hz, 2 H, CH$_2$), 1.45–1.20 (m, 6 H, CH$_2$CH$_2$CH$_2$), 0.97–0.81 (m, 3 H, CH$_3$); ^{13}C NMR (101 MHz, CHCl$_3$) δ 159.9 (Ar$_{(DHBF)}$-*C$_q$*-OCH$_2$), 157.7 (Ar-C$_q$-OBn), 154.5 (Ar$_{(DHBF)}$-*C$_q$*-OH), 136.8 (Ph-C$_q$), 135.3 (Ar$_{(p\text{-}OBn)}$-*C$_q$*-C$_q$), 128.6 (2 × Ar-CH), 128.0 (2 × Ar-CH), 128.0 (Ar-CH), 127.4 (2 × Ar-CH), 125.8 (Ar-CH), 121.2 (Ar$_{(DHBF)}$-*C$_q$*-Hexyl), 121.1 (Ar$_{(DHBF)}$-*C$_q$*-C$_q$), 115.0 (2 × Ar-CH), 97.8 (Ar-CH), 81.8 (O*C*H$_2$C$_q$), 70.0 (PhCH$_2$), 67.7 (CH$_2$OH), 55.4 (C$_q$), 31.7 (CH$_2$), 30.0 (CH$_2$), 29.8 (CH$_2$), 29.2 (CH$_2$), 22.7 (CH$_2$), 14.1 (CH$_3$); FTMS (+ p NSI) *m/z* calcd for $C_{28}H_{33}O_4^+$ [M+H]$^+$: 433.2373, Found: 433.2371.

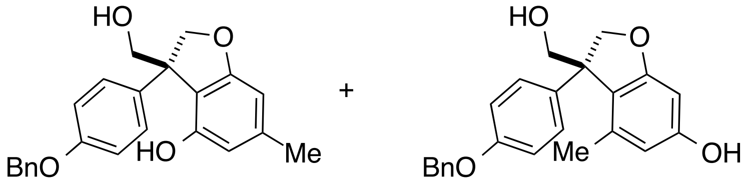

3-[4-(Benzyloxy)phenyl]-3-(hydroxymethyl)-6-methyl-2,3-dihydro-1-benzofuran-4-ol (3w) and **3-[4-(benzyloxy)phenyl]-3-(hydroxymethyl)-4-methyl-2,3-dihydro-1-benzofuran-6-ol (3w')**

Lithium bis(trifluoromethanesulfonimide) (7.9 mg, 0.0275 mmol) and tetrabutylammonium hexafluorophosphate (5.3 mg, 0.0138 mmol) were added to a solution of oxetanol **1** (64 mg, 0.25 mmol) and orcinol (155 mg, 1.25 mmol) in chloroform (0.5 mL). The reaction mixture was stirred at 40 °C for 1 h then sat. aq. $NaHCO_3$ (15 mL) was added followed by dichloromethane (15 mL). The layers were separated and the aqueous portion was extracted with dichloromethane (2 × 15 mL). The organic extracts were combined, dried over Na_2SO_4, filtered and concentrated *in vacuo*. Purification by flash column chromatography (30% EtOAc/hexane) afforded dihydrobenzofuran **3w** (21 mg, 23%) as a white solid followed by dihydroxybenzofuran **3w'** (46 mg, 50%) as a white solid.

Dihydrobenzofuran **3w (minor)**: R_f = 0.22 (30% EtOAc/hexane); mp =162–164 °C; IR (film)/cm^{-1} 3429 (br. OH), 2928, 1630, 1588, 1511, 1387, 1363, 1292, 1244, 1214, 1182, 1108, 1065, 1013, 996, 934, 831, 816, 747, 699, 668; ^{1}H NMR (400 MHz, $CDCl_3$) δ 8.23 (s, 1 H, Ar-OH), 7.48–7.29 (m, 5 H, 5 × Ar-CH), 7.16–7.06 (m, 2 H, 2 × Ar-CH), 7.00–6.90 (m, 2 H, 2 × Ar-CH), 6.40 (d, *J* = 1.4 Hz, 1 H, Ar-CH), 6.26 (d, *J* = 1.4 Hz, 1 H, Ar-CH), 5.04 (s, 2 H, $PhCH_2$), 4.46 (d, *J* = 8.8 Hz, 1 H, OC*H*HC_q), 4.34 (dd, *J* = 10.6, 3.2 Hz, 1 H, C*H*HOH), 4.29 (d, *J* = 8.8 Hz, 1 H, OCH*H*C_q), 4.10 (dd, *J* = 10.6, 7.7 Hz, 1 H, CH*H*OH), 2.50 (dd, *J* = 7.7, 3.2 Hz, 1 H, CH_2O*H*), 2.32 (s, 3 H, CH_3); ^{13}C NMR (101 MHz, $CDCl_3$) δ 161.7 ($Ar_{(DHBF)}$-C_q-OCH_2), 158.0 (Ar-C_q-OBn), 154.0 ($Ar_{(DHBF)}$-C_q-OH), 141.4 ($Ar_{(DHBF)}$-C_q-CH_3), 136.8 (Ph-C_q), 133.4 ($Ar_{(p\text{-}OBn)}$-C_q-C_q), 128.6 (2 × Ar-CH), 128.0 (3 × Ar-CH), 127.5 (2 × Ar-CH), 115.5 (2 × Ar-CH), 112.9 ($Ar_{(DHBF)}$-C_q-C_q), 110.2 (Ar-CH), 102.9 (Ar-CH), 106.2 (Ar-C_q-CH_3), 80.9 (O*C*H_2C_q), 70.1 ($PhCH_2$), 68.8 (CH_2OH), 56.4 (C_q), 21.6 (CH_3); FTMS (+ p NSI) *m/z* calcd for $C_{23}H_{23}O_4^+$ $[M+H]^+$: 363.1591, Found: 363.1593.

Dihydrobenzofuran **3w' (major)**: R_f = 0.05 (30% EtOAc/hexane); mp =173–175 °C; IR (film)/cm^{-1} 3484 (br. OH), 3293, 2956, 2898, 1739, 1608, 1597, 1510, 1476, 1453, 1381, 1251, 1186, 1176, 1133, 1055, 1044, 1024, 996, 978, 831, 809 741, 696; ^{1}H NMR (400 MHz, $(CD_3)_2SO$) δ 9.21 (s, 1 H, Ar-OH), 7.51–7.26 (m, 5 H, 5 × Ar-CH), 7.24–7.11 (m, 2 H, 2 × Ar-CH), 7.02–6.86 (m, 2 H, 2 × Ar-CH), 6.07 (d, *J* = 2.1 Hz, 1 H, Ar-CH), 6.03 (d, *J* = 2.1 Hz, 1 H, Ar-CH), 5.12 (t, *J* = 5.1 Hz, 1 H, CH_2O*H*), 5.07 (s, 2 H, $PhCH_2$), 4.73 (d, *J* = 8.8 Hz, 1 H, OC*H*HC_q), 4.24 (d, *J* = 8.8 Hz, 1 H, OCH*H*C_q), 4.07 (dd, *J* = 11.0, 5.1 Hz, 1 H, C*H*HOH), 3.81 (dd, *J* = 11.0, 5.1 Hz, 1 H, CH*H*OH), 1.76 (s, 3 H, CH_3); ^{13}C NMR (101 MHz, $(CD_3)_2SO$) δ 161.8 ($Ar_{(DHBF)}$-C_q-OCH_2), 158.4 ($Ar_{(DHBF)}$-C_q-OH), 157.1 (Ar-C_q-OBn), 137.6 (Ph-C_q), 137.1 ($Ar_{(p\text{-}OBn)}$-C_q-C_q), 135.8 ($Ar_{(DHBF)}$-C_q-CH_3), 128.9 (2 × Ar-CH), 128.7 (2 × Ar-CH), 128.3 (Ar-CH), 128.2 (2 × Ar-CH), 121.0 ($Ar_{(DHBF)}$-C_q-C_q), 114.9 (2 × Ar-CH), 110.0 (Ar-CH), 95.3 (Ar-CH), 82.5 (O*C*H_2C_q), 69.6 ($PhCH_2$), 64.3 (CH_2OH), 55.4 (C_q), 18.8 (CH_3); FTMS (+ p NSI) *m/z* calcd for $C_{23}H_{23}O_4^+$ $[M+H]^+$: 363.1591, Found: 363.1593.

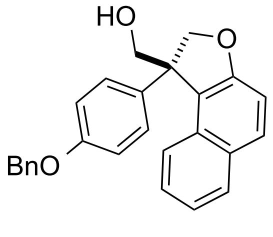

{1-[4-(Benzyloxy)phenyl]-1H,2H-naphtho[2,1-b]furan-1-yl}methanol (3x)

Lithium bis(trifluoromethanesulfonimide) (7.9 mg, 0.0275 mmol) and tetrabutylammonium hexafluorophosphate (5.3 mg, 0.0138 mmol) were added to a solution of oxetanol **1** (64 mg, 0.25 mmol) and 2-napthol (180 mg, 1.25 mmol) in chloroform (0.5 mL). The reaction mixture was stirred at 40 °C for 1 h then sat. aq. $NaHCO_3$ (15 mL) was added followed by dichloromethane (15 mL). The layers were separated and the aqueous portion was extracted with EtOAc (2 × 15 mL). The organic extracts were combined, dried over Na_2SO_4, filtered and concentrated *in vacuo*. Purification by flash column chromatography (25–30% EtOAc/hexane) afforded dihydrobenzofuran **3x** (94 mg, 98%) as a pale yellow gum. R_f = 0.31 (30% EtOAc/hexane); IR (film)/cm^{-1} 3431 (br. OH), 3063, 2887, 1626, 1609, 1598, 1579, 1510, 1455, 1374, 1294, 1244, 1183, 1063, 1012, 967, 909, 828, 811, 779, 698; ^{1}H NMR (400 MHz, $CHCl_3$) δ 7.87–7.83 (m, 1 H, Ar-CH), 7.81 (d, *J* = 8.9 Hz, 1 H, Ar-CH), 7.49–7.32 (m, 6 H, 6 × Ar-CH), 7.32–7.24 (m, 4 H, 4 × Ar-CH), 7.22 (d, *J* = 8.8 Hz, 1 H, Ar-CH), 7.00–6.92 (m, 2 H, 2 × Ar-CH), 5.07 (s, 2 H,

PhCH$_2$), 4.98 (d, *J* = 8.9 Hz, 1 H, OC*H*HC$_q$), 4.61 (d, *J* = 8.9 Hz, 1 H, OCH*H*C$_q$), 4.51 (d, *J* = 11.0 Hz, 1 H, C*H*HOH), 4.32 (dd, *J* = 11.0, 7.3 Hz, 1 H, CH*H*OH), 1.52 (d, *J* = 7.3 Hz, 1 H, OH); ^{13}C NMR (101 MHz, CHCl$_3$) δ 159.4 (Ar$_{(DHBF)}$-*C$_q$*-OCH$_2$), 157.6 (Ar-C$_q$-OBn), 136.8 (Ph-C$_q$), 135.4 (Ar$_{(p\text{-OBn})}$-*C$_q$*-C$_q$), 131.0 (Ar-CH), 130.6 (Ar$_{(napthol)}$-C$_q$), 130.0 (Ar$_{(napthol)}$-C$_q$), 129.2 (Ar-CH), 128.6 (2 × Ar-CH), 128.2 (2 × Ar-CH), 128.0 (Ar-CH), 127.5 (2 × Ar-CH), 126.8 (Ar-CH), 122.9 (Ar-CH), 122.5 (Ar-CH), 119.6 (Ar$_{(DHBF)}$-*C$_q$*-C$_q$), 115.0 (2 × Ar-CH), 112.5 (Ar-CH), 83.4 (O*C*H$_2$C$_q$), 70.0 (PhCH$_2$), 66.4 (CH$_2$OH), 57.1 (C$_q$); FTMS (+ p NSI) *m/z* calcd for $C_{26}H_{26}O_3^+$ [M+H]$^+$: 400.1907, Found: 400.1898.

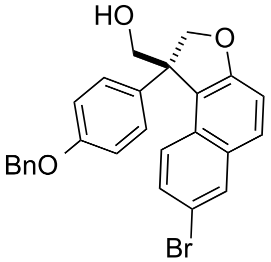

{1-[4-(Benzyloxy)phenyl]-7-bromo-1H,2H-naphtho[2,1-b]furan-1-yl}methanol (3y)

Lithium bis(trifluoromethanesulfonimide) (7.9 mg, 0.0275 mmol) and tetrabutylammonium hexafluorophosphate (5.3 mg, 0.0138 mmol) were added to a solution of oxetanol **1** (64 mg, 0.25 mmol) and 6-bromo-2-napthol (279 mg, 1.25 mmol) in chloroform (0.5 mL). The reaction mixture was stirred at 40 °C for 1 h then sat. aq. NaHCO$_3$ (15 mL) was added followed by dichloromethane (15 mL). The layers were separated and the aqueous portion was extracted with EtOAc (2 × 15 mL). The organic extracts were combined, dried over Na$_2$SO$_4$, filtered and concentrated *in vacuo*. Purification by flash column chromatography (20% EtOAc/hexane) afforded dihydrobenzofuran **3y** (102 mg, 89%) as a white solid. R$_f$ = 0.15 (20% EtOAc/hexane); mp = 115–117 °C; IR (film)/cm^{-1} 3456 (br. OH), 3060, 3033, 2937, 2863, 1840, 1618, 1580, 1506, 1453, 1376, 1354, 1297, 1182, 1127, 1072, 1015, 1004, 963, 880, 827, 802, 747, 727, 697; ^{1}H NMR (400 MHz, CHCl$_3$) δ 7.98 (d, *J* = 2.0 Hz, 1 H, Ar-CH), 7.70 (d, *J* = 8.8 Hz, 1 H, Ar-CH), 7.49–7.30 (m, 6 H, 6 × Ar-CH), 7.29–7.18 (m, 4 H, 4 × Ar-CH), 6.99–6.89 (m, 2 H, 2 × Ar-CH), 5.06 (s, 2 H, PhCH$_2$), 4.95 (d, *J* = 9.0 Hz, 1 H, OC*H*HC$_q$), 4.59 (d, *J* = 9.0 Hz, 1 H, OCH*H*C$_q$), 4.46 (dd, *J* = 11.1, 4.3 Hz, 1 H, C*H*HOH), 4.30 (dd, *J* = 11.1, 7.7 Hz, 1 H, CH*H*OH), 1.53 (dd, *J* = 7.7, 4.3 Hz, 1 H, OH); ^{13}C NMR (101 MHz, CHCl$_3$) δ 159.6 (Ar$_{(DHBF)}$-*C$_q$*-OCH$_2$), 157.7 (Ar-C$_q$-OBn), 136.8 (Ph-C$_q$), 135.0 (Ar$_{(p\text{-OBn})}$-*C$_q$*-C$_q$), 131.2 (Ar$_{(napthol)}$-C$_q$), 131.1 (Ar-CH), 130.1 (Ar-CH), 130.0 (Ar-CH), 129.1 (Ar$_{(napthol)}$-C$_q$), 128.6 (2 × Ar-CH), 128.2 (2 × Ar-CH), 128.0 (Ar-CH), 127.5 (2 × Ar-CH), 124.3 (Ar-CH), 120.5 (Ar$_{(DHBF)}$-*C$_q$*-C$_q$), 116.4 (Ar-C$_q$-Br), 115.1 (2 × Ar-CH), 113.5 (Ar-CH), 83.4 (O*C*H$_2$C$_q$), 70.0 (PhCH$_2$), 66.4 (CH$_2$OH), 56.9 (C$_q$); FTMS (+ p NSI) *m/z* calcd for $C_{26}H_{25}BrO_3^+$ [M+NH$_4$]$^+$: 478.1012, Found: 478.0999.

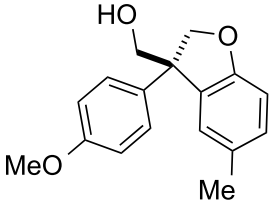

[3-(4-Methoxyphenyl)-5-methyl-2,3-dihydro-1-benzofuran-3-yl]methanol (17)

Lithium bis(trifluoromethanesulfonimide) (7.9 mg, 0.0275 mmol) or calcium bis(trifluoromethanesulfonimide) (8.3 mg, 0.0138 mmol) and tetrabutylammonium hexafluorophosphate (5.3 mg, 0.0138 mmol) were added to a solution of oxetanol **5** (45 mg, 0.25 mmol) and 4-methylphenol (135 mg, 1.25 mmol) in chloroform (0.5 mL). The reaction mixture was stirred at 40 °C for 6 h then sat. aq. NaHCO$_3$ (15 mL) was added followed by dichloromethane (15 mL). The layers were separated and the aqueous portion was extracted with dichloromethane (2 × 15 mL). The organic extracts were combined, washed with aq. NaOH (1 M, 15 mL) then dried over Na$_2$SO$_4$, filtered and concentrated *in vacuo*. Purification by flash column chromatography (30% EtOAc/hexane) afforded dihydrobenzofuran **17** (61 mg, 71%) as a pale yellow gum. R$_f$ = 0.25 (30% EtOAc/hexane); IR (film)/cm^{-1} 3433 (br. OH), 2934, 2837, 1610, 1581, 1512, 1489, 1464, 1297, 1247, 1183, 1127, 1078, 1033, 973, 829, 810, 794, 768, 731; ^{1}H NMR (400 MHz, CDCl$_3$) δ 7.35–7.21 (m, 2 H, 2 × Ar-CH), 7.05 (dt, *J* = 8.1, 1.2 Hz, 1 H, Ar-CH), 7.00 (d, *J* = 1.8 Hz, 1 H, Ar-CH), 6.93–6.85 (m, 2 H, 2 × Ar-CH), 6.81 (d, *J* = 8.1 Hz, 1 H, 1 × Ar-CH), 4.75 (d, *J* = 8.9 Hz, 1 H, OC*H*HC$_q$), 4.57 (d, *J* = 8.9 Hz, 1 H, OCH*H*C$_q$), 4.11 (dd, *J* = 11.2, 4.5 Hz, 1 H, C*H*HOH), 4.03 (dd, *J* = 11.2, 7.2 Hz, 1 H, CH*H*OH), 3.82 (s, 3 H, OCH$_3$), 2.33 (s, 3 H, CH$_3$), 1.69 (br. s, 1 H, OH); ^{13}C NMR (101 MHz, CDCl$_3$) δ 158.6 (Ar-C$_q$-OMe), 158.4 (Ar$_{(DHBF)}$-C$_q$-OCH$_2$), 134.7 (Ar$_{(p\text{-OMe})}$-*C$_q$*-C$_q$), 130.2 (Ar$_{(DHBF)}$-*C$_q$*-C$_q$), 130.0 (Ar-*C$_q$*-CH$_3$), 129.6 (Ar-CH), 128.0 (2 × Ar-CH), 125.5 (Ar-CH), 114.1 (2 × Ar-CH), 109.7 (Ar-CH), 81.3 (O*C*H$_2$C$_q$), 67.5 (CH$_2$OH), 55.7 (C$_q$), 55.2 (OCH$_3$), 20.9 (CH$_3$); FTMS (+ p NSI) *m/z* calcd for $C_{17}H_{22}O_3N^+$ [M+NH$_4$]$^+$: 288.1594, Found: 288.1596.

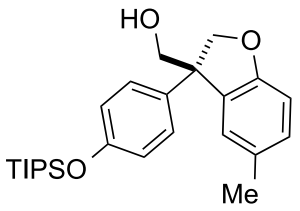

[5-Methyl-3-(4-{[tris(propan-2-yl)silyl]oxy}phenyl)-2,3-dihydro-1-benzofuran-3-yl]methanol (18)

Lithium bis(trifluoromethanesulfonimide) (7.9 mg, 0.0275 mmol) and tetrabutylammonium hexafluorophosphate (5.3 mg, 0.0138 mmol) were added to a solution of oxetanol **6** (81 mg, 0.25 mmol) and 4-methylphenol (135 mg, 1.25 mmol) in chloroform (0.5 mL). The reaction mixture was stirred at 40 °C for 20 h then sat. aq. $NaHCO_3$ (15 mL) was added followed by dichloromethane (15 mL). The layers were separated and the aqueous portion was extracted with dichloromethane (2 × 15 mL). The organic extracts were combined, washed with aq. NaOH (1 M, 15 mL) then dried over Na_2SO_4, filtered and concentrated *in vacuo*. Purification by flash column chromatography (20% EtOAc/hexane) afforded dihydrobenzofuran **18** (70 mg, 67%). R_f = 0.19 (20% EtOAc/hexane); IR (film)/cm^{-1} 3436 (br. OH), 2944, 2867, 1606, 1510, 1491, 1463, 1264, 1177, 1040, 975, 912, 882, 835, 808, 749, 725, 682; ^{1}H NMR (400 MHz, $CDCl_3$) δ 7.20–7.11 (m, 2 H, 2 × Ar-CH), 7.06–6.96 (m, 2 H, 2 × Ar-CH), 6.91–6.83 (m, 2 H, 2 × Ar-CH), 6.79 (d, *J* = 8.1 Hz, 1 H, Ar-CH), 4.75 (d, *J* = 8.8 Hz, 1 H, OC*H*HC_q), 4.56 (d, *J* = 8.8 Hz, 1 H, OCH*H*C_q), 4.06 (d, *J* = 11.0 Hz, 1 H, C*H*HOH), 4.00 (dd, *J* = 11.0, 5.1 Hz, 1 H, CH*H*OH), 2.32 (Ar-CH_3), 1.58 (d, *J* = 5.1 Hz, 1 H, OH), 1.26 (ddd, *J* = 13.6, 10.1, 6.5 Hz, 3 H, 3 × C*H*$(CH_3)_2$), 1.11 (d, *J* = 7.3 Hz, 18 H, 3 x $(CH_3)_2$); ^{13}C NMR (101 MHz, $CDCl_3$) δ 158.7 ($Ar_{(DHBF)}$-C_q-OCH_2), 155.0 (Ar-C_q-OTIPS), 134.9 ($Ar_{(p\text{-OTIPS})}$-C_q-C_q), 130.1 ($Ar_{(DHBF)}$-C_q-CH_3), 130.0 ($Ar_{(DHBF)}$-C_q-C_q), 129.6 ($Ar_{(DHBF)}$-CH), 127.9 (2 × $Ar_{(p\text{-OTIPS})}$-CH), 125.6 ($Ar_{(DHBF)}$-CH), 120.0 (2 × $Ar_{(p\text{-OTIPS})}$-CH), 109.7 ($Ar_{(DHBF)}$-CH), 81.4 (O*C*H_2C_q), 67.7 (CH_2OH), 55.8 (C_q), 20.9 (Ar-CH_3), 17.9 (3 × $(CH_3)_2$), 12.6 (3 × *C*H$(CH_3)_2$); FTMS (+ p NSI) *m/z* calcd for $C_{25}H_{40}O_3NSi^+$ $[M+NH_4]^+$: 430.2759, Found: 430.2765.

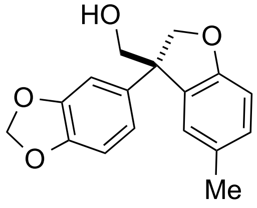

[3-(2H-1,3-Benzodioxol-5-yl)-5-methyl-2,3-dihydro-1-benzofuran-3-yl]methanol (19)

Lithium bis(trifluoromethanesulfonimide) (7.9 mg, 0.0275 mmol) and tetrabutylammonium hexafluorophosphate (5.3 mg, 0.0138 mmol) were added to a solution of oxetanol **8** (49 mg, 0.25 mmol) and 4-methylphenol (135 mg, 1.25 mmol) in chloroform (0.5 mL). The reaction mixture was stirred at 40 °C for 20 h then sat. aq. $NaHCO_3$ (15 mL) was added followed by dichloromethane (15 mL). The layers were separated and the aqueous portion was extracted with dichloromethane (2 × 15 mL). The organic extracts were combined, washed with aq. NaOH (1 M, 15 mL) then dried over Na_2SO_4, filtered and concentrated *in vacuo*. Purification by flash column chromatography (25% EtOAc/hexane) afforded dihydrobenzofuran **19** (55 mg, 78%) as a white solid. R_f = 0.19 (25% EtOAc/hexane); IR (film)/cm^{-1} 3410 (br. OH), 2885, 1610, 1504, 1485, 1435, 1234, 1202, 1125, 1110, 1035, 1065, 972, 933, 807, 728; ^{1}H NMR (400 MHz, $CDCl_3$) δ 7.03 (dd, *J* = 8.3, 1.8 Hz, 1 H, Ar-CH), 6.98 (d, *J* = 1.8 Hz, 1 H, Ar-CH), 6.83–6.73 (m, 4 H, 4 × Ar-CH), 5.95 (s, 2 H, OCH_2O), 4.71 (d, *J* = 8.9 Hz, 1 H, OC*H*HC_q), 4.53 (d, *J* = 8.9 Hz, 1 H, OCH*H*C_q), 4.07 (dd, *J* = 11.3, 3.5 Hz, 1 H, C*H*HOH), 3.99 (dd, *J* = 11.3, 6.8 Hz, 1 H, CH*H*OH), 2.32 (s, 3 H, CH_3), 1.64 (br. s, 1 H, OH); ^{13}C NMR (101 MHz, $CDCl_3$) δ 158.6 ($Ar_{(DHBF)}$-C_q-OCH_2), 148.0 (Ar-C_q-OCH_2O), 146.4 (Ar-C_q-OCH_2O), 136.7 ($Ar_{(sesamol)}$-C_q-C_q), 130.1 (Ar-C_q-CH_3), 129.9 ($Ar_{(DHBF)}$-C_q-C_q), 129.8 (Ar-CH), 125.5 (Ar-CH), 120.0 (Ar-CH), 109.8 (Ar-CH), 108.2 (Ar-CH), 107.8 (Ar-CH), 101.1 (OCH_2O), 81.4 (O*C*H_2C_q), 67.6 (CH_2OH), 56.2 (C_q), 20.9 (CH_3); FTMS (+ p NSI) *m/z* calcd for $C_{17}H_{17}O_4^+$ $[M+H]^+$: 285.1121, Found: 285.1118.

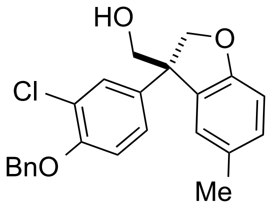

{3-[4-(Benzyloxy)-3-chlorophenyl]-5-methyl-2,3-dihydro-1-benzofuran-3-yl}-methanol (20)

Lithium bis(trifluoromethanesulfonimide) (7.9 mg, 0.0275 mmol) and tetrabutylammonium hexafluorophosphate (5.3 mg, 0.0138 mmol) were added to a solution of oxetanol **7** (73 mg, 0.25 mmol) and 4-methylphenol (135 mg, 1.25 mmol) in chloroform (0.5 mL). The reaction mixture was stirred at 40 °C for 20 h then sat. aq. $NaHCO_3$ (15 mL) was added followed by dichloromethane (15 mL). The layers were separated and the aqueous portion was extracted with dichloromethane (2 × 15 mL). The organic extracts were combined, dried over Na_2SO_4, filtered and concentrated *in vacuo*. Purification by flash column

chromatography (30% EtOAc/hexane) afforded dihydrobenzofuran **20** (39 mg, 41%) as a yellow gum. R_f = 0.28 (30% EtOAc/hexane); IR (film)/cm^{-1} 3423 (br. OH), 3032, 2926, 1739, 1603, 1490, 1454, 1381, 1293, 1255, 1240, 1125, 1061, 1023, 975, 907, 809, 731, 695; ^{1}H NMR (400 MHz, $CDCl_3$) δ 7.52–7.29 (m, 6 H, 6 × Ar-CH), 7.13 (dd, *J* = 8.7, 2.4 Hz, 1 H, Ar-CH), 7.05 (dd, *J* = 8.2, 1.8 Hz, 1 H, Ar-CH), 6.98–6.87 (m, 2 H, 2 × Ar-CH), 6.80 (d, *J* = 8.2 Hz, 1 H, Ar-CH), 5.16 (s, 2 H, $PhCH_2$), 4.72 (d, *J* = 9.0 Hz, 1 H, $OC\mathit{H}HC_q$), 4.52 (d, *J* = 9.0 Hz, 1 H, $OCH\mathit{H}C_q$), 4.09 (d, *J* = 11.1, 1 H, C*H*HOH), 4.00 (d, *J* = 11.1 Hz, 1 H, CH*H*OH), 2.32 (s, 3 H, CH_3), 1.59 (s, 1 H, OH) ^{13}C NMR (101 MHz, $CDCl_3$) δ 158.6 ($Ar_{(DHBF)}$-$\mathit{C_q}$-OCH_2), 153.0 (Ar-C_q-OBn), 136.4 (Ph-C_q + Ar-$\mathit{C_q}$-C_q), 130.4 (Ar-$\mathit{C_q}$-CH_3), 129.9 (Ar-CH), 129.6 ($Ar_{(DHBF)}$-$\mathit{C_q}$-C_q), 129.0 (Ar-CH), 128.6 (2 × Ar-CH), 128.0 (Ar-CH), 127.0 (2 × Ar-CH), 126.3 (Ar-CH), 125.3 (Ar-CH), 123.4 (Ar-$\mathit{C_q}$-Cl), 114.0 (Ar-CH), 109.9 (Ar-CH), 81.2 ($O\mathit{C}H_2C_q$), 70.8 (OCH_2Ph), 67.3 (CH_2OH), 55.6 (C_q), 20.9 (CH_3); FTMS (+ p NSI) *m/z* calcd for $C_{23}H_{25}O_3N^{35}Cl^+$ $[M+NH_4]^+$: 398.1517, Found: 398.1515.

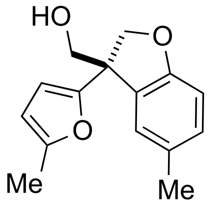

[5-Methyl-3-(5-methylfuran-2-yl)-2,3-dihydro-1-benzofuran-3-yl]methanol (21)

Lithium bis(trifluoromethanesulfonimide) (7.9 mg, 0.0275 mmol) and tetrabutylammonium hexafluorophosphate (5.3 mg, 0.0138 mmol) were added to a solution of oxetanol **9** (39 mg, 0.25 mmol) and 4-methylphenol (135 mg, 1.25 mmol) in chloroform (0.5 mL). The reaction mixture was stirred at 40 °C for 1 h then sat. aq. $NaHCO_3$ (15 mL) was added followed by dichloromethane (15 mL). The layers were separated and the aqueous portion was extracted with dichloromethane (2 × 15 mL). The organic extracts were combined, dried over Na_2SO_4, filtered and concentrated *in vacuo*. Purification by flash column chromatography (20% EtOAc/hexane) afforded dihydrobenzofuran **21** (40 mg, 66%) as a colorless gum. R_f = 0.25 (20% EtOAc/hexane); IR (film)/cm^{-1} 3409 (br. OH), 2921, 1739, 1611, 1560, 1490, 1380, 1217, 1043, 1021, 972, 809, 783; ^{1}H NMR (400 MHz, $CDCl_3$) δ 7.07 (d, *J* = 1.8 Hz, 1 H, $Ar_{(DHBF)}$-CH), 7.01 (dd, J = 8.1, 1.8 Hz, 1 H, $Ar_{(DHBF)}$-CH), 6.76 (d, *J* = 8.1 Hz, 1 H, $Ar_{(DHBF)}$-CH), 6.05 (d, *J* = 3.1 Hz, 1 H $Ar_{(furan)}$-CH), 5.92 (m, 1 H, $Ar_{(furan)}$-CH), 4.73 (d, *J* = 9.0 Hz, 1 H, $OC\mathit{H}HC_q$), 4.68 (d, *J* = 9.0 Hz, 1 H, $OCH\mathit{H}C_q$), 3.99 (dd, *J* = 11.2, 6.3 Hz, 1 H, C*H*HOH), 3.92 (dd, *J* = 11.2, 6.3 Hz, 1 H, CH*H*OH), 2.31 (s, 3 H, $Ar_{(phenol)}CH_3$), 2.29 (d, *J* = 1.0 Hz, 3 H, $Ar_{(furan)}CH_3$), 1.80 (t, *J* = 6.3 Hz, 1 H, OH); ^{13}C NMR (101 MHz, $CDCl_3$) δ 158.1 ($Ar_{(DHBF)}$-$\mathit{C_q}$-OCH_2), 153.2 ($Ar_{(furan)}$-$\mathit{C_q}$-C_q), 152.1 ($Ar_{(furan)}$-$\mathit{C_q}$-CH_3), 130.0 ($Ar_{(DHBF)}$-$\mathit{C_q}$-CH_3), 129.9 ($Ar_{(DHBF)}$-CH), 128.4 ($Ar_{(DHBF)}$-$\mathit{C_q}$-C_q), 125.0 ($Ar_{(DHBF)}$-CH), 109.7 ($Ar_{(DHBF)}$-CH), 107.5 ($Ar_{(furan)}$-CH), 106.2 ($Ar_{(furan)}$-CH), 78.0 ($O\mathit{C}H_2C_q$), 67.0 (CH_2OH), 53.2 (C_q), 20.8 ($Ar_{(DHBF)}CH_3$), 13.6 ($Ar_{(furan)}CH_3$); FTMS (+ p NSI) *m/z* calcd for $C_{15}H_{16}O_3Na^+$ $[M+Na]^+$: 267.0992, Found: 267.0993.

Synthesis of Diaryloxetanes 2z–2ab and Dihydrobenzofurans 3z–3ab

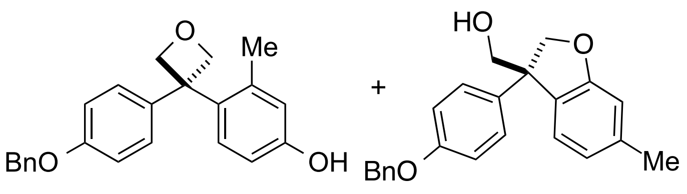

4-{3-[4-(Benzyloxy)phenyl]oxetan-3-yl}-3-methyl phenol (2z) and **{3-[4-(Benzyloxy)phenyl]-6-methyl -2,3-dihydro-1-benzofuran-3-yl}methanol (3z)**

Lithium bis(trifluoromethanesulfonimide) (7.9 mg, 0.0275 mmol) and tetrabutylammonium hexafluorophosphate (5.3 mg, 0.0138 mmol) were added to a solution of oxetanol **1** (64 mg, 0.25 mmol) and 3-methylphenol (0.13 mL, 1.25 mmol) in chloroform (0.5 mL). The reaction mixture was stirred at 40 °C for 1 h then sat. aq. $NaHCO_3$ (15 mL) was added followed by dichloromethane (15 mL). The layers were separated and the aqueous portion was extracted with dichloromethane (2 × 15 mL). The organic extracts were combined, dried over Na_2SO_4, filtered and concentrated *in vacuo*. Purification by flash column chromatography (20% EtOAc/hexane) afforded dihydrobenzofuran **3z** (51 mg, 59%) as a white solid followed by oxetane **2z** (24 mg, 27%) as a white solid.

Dihydrobenzofuran **3z**: R_f = 0.23 (20% EtOAc/hexane); mp = 80–82 °C; IR (film)/cm^{-1} 3427 (br. OH), 3034, 2923, 1608, 1590, 1509, 1497, 1454, 1425, 1380, 1294, 1242, 1182, 1129, 1115, 1080, 1009, 972, 911, 861, 828, 807, 731, 696; ^{1}H NMR (400 MHz, $CHCl_3$) δ 7.52–7.31 (m, 5 H, 5 × Ar-CH), 7.29–7.20 (m, 2 H, 2 × Ar-CH), 7.13–7.05 (m, 1 H, Ar-CH), 7.02–6.92 (m, 2 H, 2 × Ar-CH), 6.83–6.77 (m, 1 H, Ar-CH), 6.77–6.71 (m, 1 H, Ar-CH), 5.08 (s, 2 H, $PhCH_2$), 4.78 (d, *J* = 8.9 Hz, 1 H, OC*H*HC_q), 4.58 (d, *J* = 8.9 Hz, 1 H, OCH*H*C_q), 4.09 (dd, *J* = 11.2, 4.0 Hz, 1 H, C*H*HOH), 4.02 (dd, *J* = 11.2, 7.3 Hz, 1 H, CH*H*OH), 2.38 (s, 3 H, CH_3) 1.65 (br. s, 1 H, OH); ^{13}C NMR (101 MHz, $CHCl_3$) δ 161.0 ($Ar_{(DHBF)}$-C_q-OCH_2), 157.7 (Ar-C_q-OBn), 139.5 ($Ar_{(DHBF)}$-C_q-CH_3), 136.9 (Ph-C_q), 135.1 ($Ar_{(p\text{-}OBn)}$-C_q-C_q), 128.6 (2 × Ar-CH), 128.0 (2 × Ar-CH), 127.9 (Ar-CH), 127.4 (2 × Ar-CH), 127.0 ($Ar_{(DHBF)}$-C_q-C_q), 124.7 (Ar-CH), 121.6 (Ar-CH), 115.0 (2 × Ar-CH), 110.8 (Ar-CH), 81.4 (OCH_2C_q), 70.0 ($PhCH_2$), 67.7 (CH_2OH), 55.5 (C_q), 21.5 (CH_3); FTMS (+ p NSI) *m/z* calcd for $C_{23}H_{26}O_3N^+$ $[M+NH_4]^+$: 364.1907, Found: 364.1906.

Oxetane **2z**: R_f = 0.14 (20% EtOAc/hexane); mp = 151–154 °C; IR (film)/cm^{-1} 3297 (br. OH), 3031, 2960, 1877, 1608, 1580, 1507, 1455, 1380, 1295, 1243, 1182, 1116, 1010, 977, 927, 860, 830, 735, 697; ^{1}H NMR (400 MHz, $CDCl_3$) δ 7.49–7.25 (m, 7 H, 7 × Ar-CH), 6.99–6.86 (m, 3 H, 3 × Ar-CH), 6.73 (dd, *J* = 8.3, 2.7 Hz, 1 H, Ar-H), 6.69 (d, *J* = 2.7 Hz, 1 H, Ar-H), 5.41 (d, *J* = 5.5 Hz, 2 H, C*H*HOC*H*H), 5.10 (s, 1 H, OH), 5.06 (s, 2 H, $PhCH_2$), 4.92 (d, *J* = 5.5 Hz, 2 H, CH*H*OCH*H*), 1.80 (s, 3 H, CH_3); ^{13}C NMR (101 MHz, $CDCl_3$) δ 157.6 (Ar-C_q-OBn), 154.5 (Ar-C_q-OH), 138.2 ($Ar_{(p\text{-}OBn)}$-C_q-C_q), 137.6 (Ar-C_q-CH_3), 136.9 (Ph-C_q), 134.5 ($Ar_{(phenol)}$-C_q-C_q), 128.6 (2 × Ar-CH), 128.4 (Ar-CH), 128.0 (Ar-CH), 127.5 (2 × Ar-CH), 126.9 (2 × Ar-CH), 118.0 (Ar-CH), 114.7 (2 × Ar-CH), 112.6 (Ar-CH), 84.7 (CH_2OCH_2), 70.0 (Ph-CH_2), 50.6 (C_q), 19.7 (CH_3); FTMS (+ p NSI) *m/z* calcd for $C_{23}H_{26}O_3N^+$ $[M+NH_4]^+$: 364.1907, Found: 364.1908.

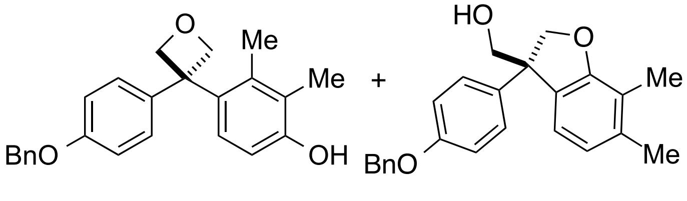

4-{3-[4-(Benzyloxy)phenyl]oxetan-3-yl}-2,3-di methylphenol (2aa) and **{3-[4-(benzyloxy)phenyl]-6,7-dimethyl-2,3-dihydro-1-benzofuran-3-yl} methanol (3aa)**

Lithium bis(trifluoromethanesulfonimide) (7.9 mg, 0.0275 mmol) and tetrabutylammonium hexafluorophosphate (5.3 mg, 0.0138 mmol) were added to a solution of oxetanol **1** (64 mg, 0.25 mmol) and 2,3-dimethylphenol (153 mg, 1.25 mmol) in chloroform (0.5 mL). The reaction mixture was stirred at 40 °C for 1 h then sat. aq. $NaHCO_3$ (15 mL) was added followed by dichloromethane (15 mL). The layers were separated and the aqueous portion was extracted with dichloromethane (2 × 15 mL). The organic extracts were combined, dried over Na_2SO_4, filtered and concentrated *in vacuo*. Purification by flash column chromatography (3% EtOAc/CH_2Cl_2) afforded dihydrobenzofuran **3aa** (18 mg, 20%) as a yellow gum followed by oxetane **2aa** (46 mg, 51%) as a white solid.

Dihydrobenzofuran **3aa**: R_f = 0.30 (3% EtOAc/CH_2Cl_2); IR (film)/cm^{-1} 3381 (br. OH), 3035, 2920, 1608, 1589, 1510, 1454, 1381, 1242, 1182, 1079, 1021, 976, 908, 828, 805, 730, 696; ^{1}H NMR (400 MHz, $CHCl_3$) δ 7.52–7.15 (m, 7 H, 7 × Ar-CH), 7.02–6.87 (m, 3 H, 3 × Ar-CH), 6.79 (d, *J* = 7.6 Hz, 1 H, Ar-CH), 5.07 (s, 2 H, $PhCH_2$), 4.78 (d, *J* = 8.9 Hz, 1 H, OC*H*HC$_q$), 4.60 (d, *J* = 8.9 Hz, 1 H, OCH*H*C$_q$), 4.09 (d, *J* = 11.1 Hz, 1 H, C*H*HOH), 4.02 (d, *J* = 11.1 Hz, 1 H, CH*H*OH), 2.30 (s, 3 H, CH_3), 2.19 (s, 3 H, CH_3); ^{13}C NMR (101 MHz, $CHCl_3$) δ 159.4 ($Ar_{(DHBF)}$-*C$_q$*-OCH_2), 157.7 (Ar-C$_q$-OBn), 138.0 (Ar-*C$_q$*-CH_3), 136.9 (Ph-C$_q$), 135.3 ($Ar_{(p\text{-}OBn)}$-*C$_q$*-C$_q$), 128.6 (2 × Ar-CH), 128.1 (2 × Ar-CH), 128.0 (Ar-CH), 127.4 (2 × Ar-CH), 126.6 ($Ar_{(DHBF)}$-*C$_q$*-C$_q$), 122.2 (Ar-CH), 121.6 (Ar-CH), 119.1 (Ar-*C$_q$*-CH_3), 115.0 (2 × Ar-CH), 81.1 (O*C*H_2C$_q$), 70.0 ($PhCH_2$), 67.7 (CH_2OH), 56.1 (C$_q$), 19.4 (CH_3), 11.7 (CH_3); FTMS (+ p NSI) *m/z* calcd for $C_{24}H_{25}O_3^+$ $[M+H]^+$: 361.1798, Found; 361.1800.

Oxetane **2aa**: R_f = 0.16 (3% EtOAc/CH_2Cl_2); mp = 185–187 °C; IR (film)/cm^{-1} 3306 (br. OH), 1587, 1507, 1457, 1382, 1281, 1242, 1182, 1064, 1017, 970, 919, 864, 834, 813, 748, 736, 698; ^{1}H NMR (400 MHz, $(CD_3)_2SO$) δ 9.21 (s, 1 H, O-H), 7.50–7.27 (m, 5 H, 5 × Ar-CH), 7.27–7.17 (m, 2 H, 2 × Ar-CH), 7.01–6.89 (m, 2 H, 2 × Ar-CH), 6.73 (q, *J* = 8.3 Hz, 1 H, Ar-CH), 5.16 (d, *J* = 5.7 Hz, 2 H, C*H*HOC*H*H), 5.07 (s, 2 H, $PhCH_2$), 4.80 (d, *J* = 5.7 Hz, 2 H, CH*H*OCH*H*), 2.03 (s, 3 H, CH_3), 1.62 (s, 3 H, CH_3); ^{13}C NMR (101 MHz, $(CD_3)_2SO$) δ 157.4 (Ar-*C$_q$*-OBn), 154.2 (Ar-C$_q$-OH), 139.2 ($Ar_{(p\text{-}OBn)}$-*C$_q$*-C$_q$), 137.6 (Ph-C$_q$), 135.5 (Ar-*C$_q$*-CH_3), 133.7 ($Ar_{(phenol)}$-*C$_q$*-C$_q$), 128.9 (2 × Ar-CH), 128.3 (Ar-CH), 128.1 (2 × Ar-CH), 127.2 (2 × Ar-CH), 125.2 (Ar-CH), 123.8 (Ar-*C$_q$*-CH_3), 115.0 (2 × Ar-CH), 112.4 (Ar-CH), 84.1 (CH_2OCH_2), 69.7 ($PhCH_2$), 50.9 (C$_q$), 16.8 (CH_3), 12.3 (CH_3); FTMS (+ p NSI) *m/z* calcd for $C_{24}H_{28}O_3N^+$ $[M+NH_4]^+$: 378.2064, Found: 378.2063.

4-{3-[4-(Benzyloxy)phenyl]oxetan-3-yl}-3-fluorophenol (2ab) and **{3-[4-(benzyloxy)phenyl]-6-fluoro-2,3-dihydro-1-benzofuran-3-yl}methanol (3ab major)** and **{3-[4-(benzyloxy)phenyl]-4-fluoro-2,3-dihydro-1-benzofuran-3-yl}methanol (3ab minor)**

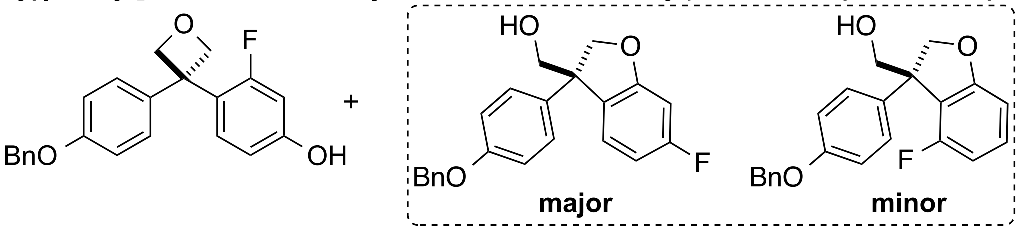

Lithium bis(trifluoromethanesulfonimide) (7.9 mg, 0.0275 mmol) and tetrabutylammonium hexafluorophosphate (5.3 mg, 0.0138 mmol) were added to a solution of oxetanol **1** (64 mg, 0.25 mmol) and 3-fluorophenol (0.11 mL, 1.25 mmol) in chloroform (0.5 mL). The reaction mixture was stirred at 40 °C for 1 h then sat. aq. $NaHCO_3$ (15 mL) was added followed by dichloromethane (15 mL). The layers were separated and the aqueous portion was extracted with dichloromethane (2 × 15 mL). The organic extracts were combined, dried over Na_2SO_4, filtered and concentrated *in vacuo*. Purification by flash column chromatography (30% EtOAc/hexane) afforded a mixture dihydrobenzofurans **3ab** (24 mg, 28%, 85:15) as a colorless gum followed by oxetane **2ab** (39 mg, 44%) as a white solid.

Dihydrobenzofurans **3ab (a, major)** and **(b, minor)**: R_f = 0.37 (30% EtOAc/hexane); IR (film)/cm^{-1} 3422 (br. OH), 3034, 2875, 1608, 1510, 1491, 1454, 1437, 1294, 1183, 1133, 1107, 1085, 1018, 982, 908, 829, 806, 731, 697; ^{1}H NMR (400 MHz, $CHCl_3$) δ 7.51–7.29 (m, 5 H^a + 5 H^b, 5 × Ar-CH^a + 5 × Ar-CH^b), 7.24–7.17 (m, 2 H^a + 3 H^b, 2 × Ar-CH^a + 3 × Ar-CH^b), 7.13 (dd, *J* = 8.2, 5.7 Hz, 1 H^a, Ar-CH^a), 7.03–6.84 (m, 2 H^a + 2 H^b, 2 × Ar-CH^a + 2 × Ar-CH^b), 6.76–6.50 (m, 2 H^a + 2 H^b, 2 × Ar-CH^a + 2 × Ar-CH^b), 5.06 (s, 2 H^a + 2 H^b, $PhCH_2^a$ + $PhCH_2^b$), 4.86 (d, *J* = 9.0 Hz, 1 H^b, OC*H*HC$_q^b$), 4.80 (d, *J* = 9.0 Hz, 1 H, OC*H*HC$_q^a$), 4.64 (d, *J* = 9.0 Hz, 1 H^b, OCH*H*C$_q^b$), 4.64 (d, *J* = 9.0 Hz, 1 H^a, OCH*H*C$_q^a$), 4.26 (d, *J* = 11.0 Hz, 1 H, C*H*HOH^b), 4.15 (dd, *J* = 11.0, 4.2 Hz, 1 H, CH*H*OH^b), 4.09 (d, *J* = 10.8 Hz, 1 H, C*H*HOH^a), 4.01 (dd, *J* = 10.8, 5.8 Hz, 1 H, CH*H*OH^a), 1.65 (s, 1 H, OH^b); ^{13}C NMR (101 MHz, $CHCl_3$) δ 163.8 (d, $J_{C\text{-}F}$ = 244.8 Hz, Ar-C$_q$-F), 161.9 (d, $J_{C\text{-}F}$ = 13.3 Hz, $Ar_{(DHBF)}$-*C$_q$*-OCH_2), 157.9 (Ar-C$_q$-OBn), 136.8 (Ph-C$_q$), 134.6 ($Ar_{(p\text{-}OBn)}$-*C$_q$*-C$_q$), 128.6 (2 × Ar-CH), 128.1 (Ar-CH), 128.0 (2 × Ar-CH), 127.5 (2 × Ar-CH), 125.9 (d, $J_{C\text{-}F}$ = 2.4 Hz, $Ar_{(DHBF)}$-*C$_q$*-C$_q$) 125.6 (d, $J_{C\text{-}F}$ = 10.8 Hz, Ar-CH), 115.1 (2 × Ar-CH), 107.6 (d, $J_{C\text{-}F}$ = 22.8 Hz, Ar-CH), 98.6 (d, $J_{C\text{-}F}$ = 26.5 Hz, Ar-CH), 82.4

(O*C*H_2C_q), 70.1 ($PhCH_2$), 67.7 (CH_2OH), 55.1 (C_q); ^{19}F NMR (377 MHz, $CDCl_3$) δ –112.6 (a, major), –117.1 (b, minor); FTMS (+ p NSI) *m/z* calcd for $C_{22}H_{23}O_3NF^+$ $[M+NH_4]^+$:368.1656, Found: 368.1656.

Oxetane **2ab**: R_f = 0.27 (30% EtOAc/hexane); mp = 185–187 °C; IR (film)/cm^{-1} 3276 (br. OH), 2954, 2882, 1738, 1623, 1610, 1599, 1511, 1455, 1388, 1348, 1298, 1280, 1245, 1188, 1161, 1116, 1013, 968, 942, 846, 834, 816, 746; ^{1}H NMR (400 MHz, $CDCl_3$) δ 7.49–7.24 (m, 7 H, 7 × Ar-CH), 7.02–6.87 (m, 3 H, 3 × Ar-CH), 6.66 (dd, *J* = 8.3, 2.5 Hz, 1 H, Ar-CH), 6.58 (dd, *J* = 11.6, 2.5 Hz, 1 H, Ar-CH), 5.36 (dd, *J* = 5.6, 1.3 Hz, 2 H, C*H*HOC*H*H), 5.30 (s, 1 H, OH), 5.06 (s, 2 H, $PhCH_2$), 5.06 (d, *J* = 5.6 Hz, 4 H, CH*H*OCH*H* + $PhCH_2$); ^{13}C NMR (101 MHz, $CDCl_3$) δ 160.8 (d, $J_{C\text{-}F}$ = 246.8 Hz, Ar-C_q-F), 157.7 (Ar-C_q-OBn), 156.0 (d, $J_{C\text{-}F}$ = 11.6 Hz, Ar-C_q-OH), 137.7 ($Ar_{(p\text{-}OBn)}$-C_q-C_q), 136.9 (Ph-C_q), 128.6 (2 × Ar-CH), 128.5 (d, $J_{C\text{-}F}$ = 6.7 Hz, Ar-CH), 128.0 (Ar-CH), 127.4 (2 × Ar-CH), 126.8 (2 × Ar-CH), 124.0 (d, $J_{C\text{-}F}$ = 14.5 Hz, $Ar_{(phenol)}$-C_q-C_q), 114.8 (2 × Ar-CH), 111.3 (d, $J_{C\text{-}F}$ = 2.8 Hz, Ar-CH), 103.8 (d, $J_{C\text{-}F}$ = 24.7 Hz, Ar-CH), 84.1 (CH_2OCH_2), 70.1 ($PhCH_2$), 48.0 (C_q); ^{19}F NMR (377 MHz, $CDCl_3$) δ –113.1; FTMS (+ p NSI) *m/z* calcd for $C_{22}H_{23}O_3NF^+$ $[M+NH_4]^+$: 368.1656, Found: 368.1657.

Functionalization of Oxetane 2a

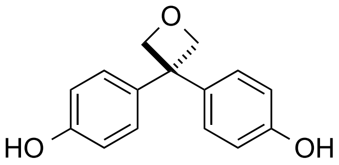

4-[3-(4-Hydroxyphenyl)oxetan-3-yl]phenol (22)

Palladium on carbon (10% w/w, 17 mg) was added to a solution of oxetane **2a** (166 mg, 0.5 mmol) in ethanol (4.5 mL). The reaction mixture was stirred under an atmosphere of hydrogen at 25 °C overnight. The reaction mixture was filtered through celite, washed through with ethanol and then concentrated *in vacuo* to afford oxetane **22** (121 mg, 100%) as a white solid. R_f = 0.35 (50% EtOAc/hexane); mp = 237–239 °C; IR (film)/cm^{-1} 3183 (br. OH), 2953, 2880, 2506, 2365, 1611, 1514, 1438, 1266, 1182, 1111, 969, 929, 841, 818, 752; ^{1}H NMR (400 MHz, CD_3OD) δ 7.11–7.00 (m, 4 H, 4 × Ar-CH), 6.83–6.73 (m, 4 H, 4 × Ar-CH), 5.18 (s, 4 H, CH_2OCH_2); ^{13}C NMR (101 MHz, CD_3OD) δ 155.6 (Ar-C_q-OH), 137.2 (Ar-C_q-C_q), 127.2 (2 × Ar-CH), 114.8 (2 × Ar-CH), 84.8 (CH_2OCH_2), 50.2 (C_q); FTMS (+ p NSI) m/z calcd for $C_{15}H_{18}O_3N^+$ $[M+NH_4]^+$: 260.1281, Found: 260.1283.

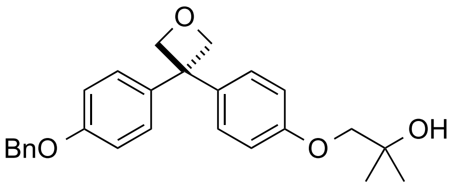

1-(4-{3-[4-(Benzyloxy)phenyl]oxetan-3-yl}phenoxy)-2-methylpropan-2-ol (23)

1,1-Dimethyloxirane (44 μL, 0.50 mmol) was added to a solution of oxetane **2a** (83 mg, 0.25 mmol), K_2CO_3 (48 mg, 0.35 mmol) in DMF (0.5 mL). The reaction mixture was stirred at 100 °C for 40 h then water (20 mL) was added followed by diethylether (20 mL). The layers were separated and the aqueous portion was extracted with diethylether (20 mL). The organic extracts were combined, washed with brine (2 × 15 mL), dried over Na_2SO_4, filtered and concentrated *in vacuo*. Purification by flash column chromatography (30% EtOAc/hexane) afforded oxetane **23** (77 mg, 77%) as a white solid. R_f = 0.13 (30% EtOAc/hexane); mp = 115–117 °C; IR (film)/cm^{-1} 3377 (br. OH), 2974, 1739, 1609, 1509, 1227, 1179, 1041, 1011, 977, 939, 833, 817, 745, 698; ^{1}H NMR (400 MHz, $CDCl_3$) δ 7.49–7.30 (m, 5 H, 5 × Ar-CH), 7.21–7.09 (m, 4 H, 4 × Ar-CH), 7.01–6.94 (m, 2 H, 2 × Ar-CH), 6.94–6.88 (m, 2 H, 2 × Ar-CH), 5.20 (s, 4 H, CH_2OCH_2), 5.07 (s, 2 H, $PhCH_2$), 3.80 ($CH_2C_q(CH_3)_2$), 2.24 (s, 1 H, OH), 1.36 (s, 6 H, 2 × CH_3); ^{13}C NMR (101 MHz, $CDCl_3$) δ 157.5 (Ar-C_q-O), 157.4 (Ar-C_q-O), 138.6 (Ar-C_q-C_q), 138.4 (Ar-C_q-C_q), 137.0 (Ph-C_q), 128.6 (2 × Ar-CH), 128.0 (Ar-CH), 127.7 (2 × Ar-CH), 127.6 (2 × Ar-CH), 127.5 (2 × Ar-CH), 114.8 (2 × Ar-CH), 114.6 (2 × Ar-CH), 84.9 (CH_2OCH_2), 76.1 ($C_q(CH_3)_2$), 70.1 ($PhCH_2$), 50.4 ($CH_2C_qCH_2$), 26.2 (2 × CH_3); FTMS (+ p NSI) m/z calcd for $C_{26}H_{32}O_4N^+$ $[M+NH_4]^+$: 422.2326, Found: 422.2323.

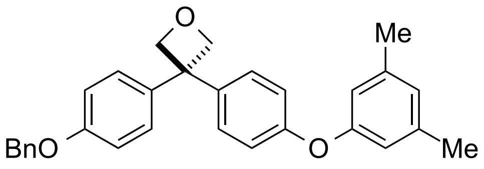

3-[4-(Benzyloxy)phenyl]-3-[4-(3,5-dimethylphenoxy)phenyl]oxetane (24)

Using conditions developed by Ma,[14] oxetane **2a** (99.7 mg, 0.30 mmol), *N,N*-dimethylglycine.HCl (7.9 mg, 0.055 mmol), CuI (2.9 mg, 0.015 mmol) and Cs_2CO_3 (196 mg, 0.60 mmol) were added to a reaction vial. The reaction vial was evacuated and then refilled with nitrogen (× 3). 5-Iodo-*m*-xylene (65 μL, 0.45 mmol) then 1,4-dioxane (0.6 mL) were added *via* syringe. The reaction mixture was stirred at 90 °C for 20 h then cooled to rt. Brine (20 mL) was added and the layers separated. The aqueous portion was extracted with EtOAc (3 × 15 mL). The organic extracts were combined, washed with brine (15 mL), dried over Na_2SO_4, filtered and concentrated *in vacuo*. Purification by flash column chromatography (15% EtOAc/hexane) afforded oxetane **24** (99 mg, 76%) as a white solid. R_f = 0.26 (15% EtOAc/hexane); mp = 154–157 °C; IR (film)/cm^{-1} 2881, 1586, 1504, 1295, 1232, 1219, 1167, 1026, 1013, 989, 833, 743, 696, 688; ^{1}H NMR (400 MHz, $CDCl_3$) δ 7.52–7.28 (m, 5 H, 5 × Ar-CH), 7.23–7.11 (m, 4 H, 4 × Ar-CH), 7.03–6.92 (m, 4 H, 4 × Ar-CH), 6.81–6.73 (m, 1 H, Ar-CH), 6.72–6.64 (m, 2 H, 2 × Ar-CH), 5.23 (s, 4 H, CH_2OCH_2), 5.08 (s, 2 H, $PhCH_2$), 2.31 (s, 6 H, 2 × CH_3); ^{13}C NMR (101 MHz, $CDCl_3$) δ 157.5 (Ar-C_q-OBn), 156.9 ($Ar_{(m\text{-xylene})}$-C_q-O), 156.1 ($Ar_{(p\text{-oxetane})}$-C_q-O), 140.5 (Ar-C_q-C_q), 139.6 (2 × Ar-C_q-CH_3), 138.2 (Ar-C_q-C_q), 136.9 (Ph-C_q), 128.6 (2 × Ar-CH), 128.0 (Ar-CH), 127.8 (2 × Ar-CH), 127.6 (2 × Ar-CH), 127.5 (2 × Ar-CH), 125.1 (Ar-CH), 118.6 (2 × Ar-CH), 116.7 (2 × Ar-CH), 114.8 (2 × Ar-CH), 84.8

(CH_2OCH_2), 70.1 ($PhCH_2$), 50.5 (C_q), 21.3 (2 × CH_3); FTMS (+ p NSI) m/z calcd for $C_{30}H_{32}O_3N^+$ $[M+NH_4]^+$: 454.2377, Found: 454.2368.

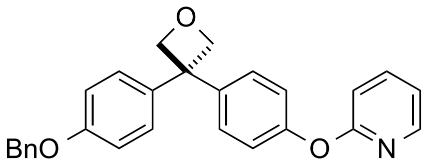

2-(4-{3-[4-(Benzyloxy)phenyl]oxetan-3-yl}phenoxy)pyridine (25)

Using Ullmann conditions developed by Buchwald,[15] oxetane **2a** (99.7 mg, 0.30 mmol), picolinic acid (3.7 mg, 0.03 mmol), CuI (2.9 mg, 0.015 mmol) and K_3PO_4 (127 mg, 0.60 mmol) were added to a reaction vial. The reaction vial was evacuated and then refilled with nitrogen (× 3). 2-Iodopyridine (38 μL, 0.36 mmol) then DMSO (0.6 mL) were added *via* syringe. The reaction mixture was stirred at 90 °C for 23 h then cooled to rt then water (20 mL) was added followed by EtOAc (15 mL). The layers were separated and the aqueous portion was extracted with EtOAc (2 × 15 mL). The organic extracts were combined, dried over Na_2SO_4, filtered and concentrated *in vacuo*. Purification by flash column chromatography (20% EtOAc/hexane) afforded oxetane **25** (103 mg, 84%) as a white solid. R_f = 0.35 (25% EtOAc/hexane); mp = 93–95 °C; IR (film)/cm^{-1} 2961, 2885, 1581, 1506, 1464, 1425, 1231, 1213, 1187, 1010, 987, 855, 750, 735, 702; ^{1}H NMR (400 MHz, $CDCl_3$) δ 8.24–8.20 (m, 1 H, $Ar_{(py)}$-CH), 7.74–7.66 (ddd, J = 8.2, 7.2, 2.0 Hz, 1 H, $Ar_{(py)}$-CH), 7.48–7.31 (m, 5 H, 5 × Ar-CH), 7.30–7.22 (m, 2 H, 2 × Ar-CH), 7.20–7.10 (m, 4 H, 4 × Ar-CH), 7.04–6.91 (m, 4 H, 4 × Ar-CH), 5.24 (s, 4 H, CH_2OCH_2), 5.09 (s, 2 H, $PhCH_2$); ^{13}C NMR (101 MHz, $CDCl_3$) δ 163.5 ($Ar_{(py)}$-C_q), 157.5 (Ar-C_q-OBn), 152.7 (Ar-C_q-OPy), 147.7 ($Ar_{(py)}$-CH), 142.2 (Ar-C_q-C_q), 139.4 ($Ar_{(py)}$-CH), 138.0 (Ar-C_q-C_q), 136.9 (Ph-C_q), 128.6 (2 × Ar-CH), 128.0 (Ar-CH), 127.8 (2 × Ar-CH), 127.7 (2 × Ar-CH), 127.5 (2 × Ar-CH), 121.0 (2 × Ar-CH), 118.5 ($Ar_{(py)}$-CH), 114.9 (2 × Ar-CH), 111.7 (Ar-CH), 84.8 (CH_2OCH_2), 70.1 ($PhCH_2$), 50.6 (C_q); FTMS (+ p NSI) m/z calcd for $C_{27}H_{24}O_3N^+$ $[M+H]^+$: 410.1751, Found: 410.1745.

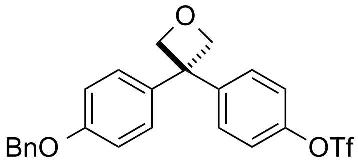

4-{3-[4-(Benzyloxy)phenyl]oxetan-3-yl}phenyl trifluoromethanesulfonate (26)

Pyridine (0.24 mL, 3.0 mmol) then triflic anhydride (0.30 mL, 1.8 mmol) were added to a solution of oxetane **2a** (499 mg, 1.5 mmol) in dichloromethane (3.0 mL). The reaction mixture was stirred at 25 °C for 3 h then water (20 mL) was added followed by dichloromethane (15 mL). The layers were separated and the aqueous portion was extracted with dichloromethane (2 × 15 mL). The organic extracts were combined, dried over Na_2SO_4, filtered and concentrated *in vacuo*. Purification by flash column chromatography (20% EtOAc/hexane) afforded oxetane **26** (584 mg, 84%) as a white solid. R_f = 0.36 (20% EtOAc/hexane); mp = 103–104 °C; IR (film)/cm^{-1} 2960, 2871, 1610, 1516, 1499, 1423, 1250, 1207, 1182, 1136, 1017, 897, 884, 845, 743, 696; ^{1}H NMR (400 MHz, ($CDCl_3$) δ 7.49–7.30 (m, 7 H, 7 × Ar-CH), 7.30–7.20 (m, 2 H, 2 × Ar-CH), 7.12–7.04 (m, 2 H, 2 × Ar-CH), 7.04–6.96 (m, 2 H, 2 × Ar-CH), 5.27 (d, J = 5.8 Hz, 2 H, C*H*HOC*H*H), 5.15 (d, J = 5.8 Hz, 2 H, CH*H*OCH*H*), 5.09 (s, 2 H, Ph-CH_2); ^{13}C NMR (101 MHz, $CDCl_3$) δ 157.4 (Ar-C_q-OBn), 148.1 (Ar-C_q-OTf), 146.8 (Ar-C_q-C_q), 136.81 (Ar-C_q-C_q or Ph-C_q), 136.77 (Ar-C_q-C_q or Ph-C_q), 128.6 (2 × Ar-CH), 128.4 (2 × Ar-CH), 128.0 (Ar-CH), 127.6 (2 × Ar-CH), 127.5 (2 × Ar-CH), 121.4 (2 × Ar-CH), 118.7 (q, J_{CF} = 319.3 Hz, CF_3), 115.1 (2 × Ar-CH), 84.4 (CH_2OCH_2), 70.1 ($PhCH_2$), 50.7 (C_q); ^{19}F NMR (377 MHz, $CDCl_3$) δ –72.8; (FTMS (+ p NSI) m/z calcd for $C_{23}H_{23}O_5NF_3S^+$ $[M+NH_4]^+$: 482.1244, Found: 482.1232.

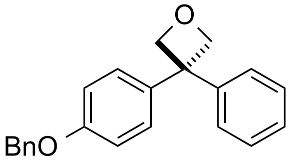

3-[4-(Benzyloxy)phenyl]-3-phenyloxetane (27)

Using conditions developed by Ortar,[16] Triethylamine (105 μL, 0.75 mmol) then formic acid (19 μL, 0.50 mmol) were added to a solution of oxetane **26** (116 mg, 0.25 mmol), $Pd(OAc)_2$ (1.1 mg, 0.005 mmol), 1,1'-bis(diphenylphosphino)ferrocene (2.8 mg, 0.005 mmol) in dimethylformamide (3.0 mL). The reaction mixture was stirred at 60 °C for 1 h then brine (20 mL) was added followed by diethylether (15 mL). The layers were separated and the aqueous portion was extracted with diethylether (2 × 15 mL). The organic extracts were combined, washed with brine (2 × 15 mL), dried over Na_2SO_4, filtered and concentrated *in vacuo*. Purification by flash column chromatography (15% EtOAc/hexane) afforded oxetane **27** (44 mg, 55%) as a white solid. R_f = 0.29 (15% EtOAc/hexane); mp = 130–132 °C; IR (film)/cm^{-1} 2953, 2938, 2874, 1509, 1497,

1383, 1239, 1184, 1009, 988, 832, 784, 763, 745, 699; ^{1}H NMR (400 MHz, ($CDCl_3$) δ 7.41–7.22 (m, 7 H, 7 × Ar-CH), 7.23–7.11 (m, 3 H, 3 × Ar-CH), 7.11–7.04 (m, 2 H, 2 × Ar-CH), 6.93–6.85 (m, 2 H, 2 × Ar-CH), 5.16 (d, *J* = 5.6 Hz, 2 H, C*H*HOC*H*H), 5.14 (d, *J* = 5.6 Hz, 2 H, CH*H*OCH*H*), 4.99 (s, 2 H, $PhCH_2$); ^{13}C NMR (101 MHz, $CDCl_3$) δ 157.4 (Ar-C_q-OBn), 145.9 (Ar-C_q-C_q), 138.2 (Ar-C_q-C_q), 136.9 (Ph-C_q), 128.6 (2 × Ar-CH), 128.5 (2 × Ar-CH), 128.0 (Ar-CH), 127.6 (2 × Ar-CH), 127.4 (2 × Ar-CH), 126.6 (Ar-CH), 126.5 (2 × Ar-CH), 114.8 (2 × Ar-CH), 84.7 (CH_2OCH_2), 70.0 ($PhCH_2$), 50.9 (C_q); FTMS (+ p NSI) m/z calcd for $C_{22}H_{24}O_2N^+$ $[M+NH_4]^+$: 334.1802, Found: 334.1804.

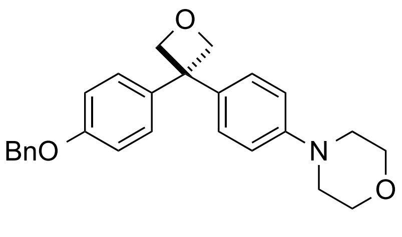

4-(4-{3-[4-(Benzyloxy)phenyl]oxetan-3-yl}phenyl)morpholine (28)

Using conditions developed by Buchwald,[17] oxetane **26** (232 mg, 0.50 mmol), $Pd(OAc)_2$ (1.1 mg, 0.005 mmol), JohnPhos (3.0 mg, 0.010 mmol) and K_3PO_4 (149 mg, 0.70 mmol) were added to a reaction vial. The reaction vial was evacuated and then refilled with nitrogen (× 3). Morpholine (53 μL, 0.60 mmol) then THF (1.0 mL) were added *via* syringe. The reaction mixture was stirred at 65 °C for 24 h then cooled to rt. Diethylether (15 mL) was added and the crude mixture was filtered through celite® and then concentrated *in vacuo*. Purification by flash column chromatography (30% EtOAc/hexane) afforded oxetane **28** (189 mg, 94%) as a white solid. R_f = 0.14 (30% EtOAc/hexane); mp = 131–133 °C; IR (film)/cm^{-1} 2955, 2188, 1736, 1609, 1513, 1451, 1380, 1229, 1183, 1119, 1017, 981, 921, 817, 749, 697; ^{1}H NMR (400 MHz, ($CDCl_3$) δ 7.54–7.28 (m, 5 H, 5 × Ar-CH), 7.22–7.07 (m, 4 H, 4 × Ar-CH), 7.00–6.86 (m, 4 H, 4 × Ar-CH), 5.22 (d, *J* = 5.7 Hz, 2 H, C*H*HOC*H*H), 5.20 (d, *J* = 5.7 Hz, 2 H, CH*H*OCH*H*), 5.07 (s, 2 H, $PhCH_2$), 3.97–3.82 (m, 4 H, CH_2C*H*$_2$OC*H*$_2$$CH_2$), 3.25–3.09 (m, 4 H, CH_2NCH_2); ^{13}C NMR (101 MHz, $CDCl_3$) δ 157.4 (Ar-C_q-OBn), 145.9 (Ar-C_q-N), 138.5 (Ar-C_q-C_q), 137.3 (Ar-C_q-C_q), 136.9 (Ph-C_q), 128.6 (2 × Ar-CH), 128.0 (Ar-CH), 127.6 (2 × Ar-CH), 127.5 (2 × Ar-CH), 127.3 (2 × Ar-CH), 115.5 (2 × Ar-CH), 114.8 (2 × Ar-CH), 84.9 (CH_2OCH_2), 70.1 ($PhCH_2$), 66.9 (CH_2*C*H_2O*C*H_2CH_2), 50.3 (C_q), 49.2 (CH_2NCH_2); FTMS (+ p NSI) m/z calcd for $C_{26}H_{28}O_3N^+$ $[M+H]^+$: 402.2064, Found: 402.2055.

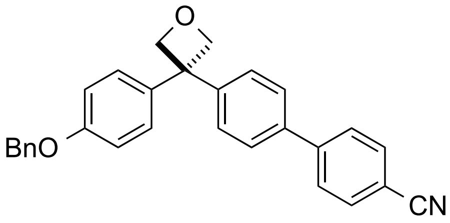

4-(4-{3-[4-(Benzyloxy)phenyl]oxetan-3-yl}phenyl)benzonitrile (29)

Oxetane **26** (139 mg, 0.30 mmol), 4-cyanoboronic acid (58.8 mg, 0.40 mmol), $Pd(OAc)_2$ (3.4 mg, 0.015 mmol), SPhos (12.3 mg, 0.03 mmol) and K_3PO_4 (82.9 mg, 0.60 mmol) were added to a reaction vial. The reaction vial was evacuated and then refilled with nitrogen (× 3). Dioxane/water (4:1, 3.0 mL) was added *via* syringe. The reaction mixture was stirred at 65 °C for 18 h then cooled to rt. Diethylether (15 mL) was added and the crude mixture was filtered through celite® and then concentrated *in vacuo*. Purification by flash column chromatography (20% EtOAc/hexane) afforded oxetane **29** (57 mg, 45%) as a white solid. R_f = 0.12 (20% EtOAc/hexane); mp = 169–172 °C; IR (film)/cm^{-1} 2871, 2224 (CN), 1738, 1607, 1517, 1492, 1256, 1239, 1178, 1028, 982, 856, 824, 737; ^{1}H NMR (400 MHz, ($CDCl_3$) δ 7.76–7.66 (m, 4 H, 4 × Ar-CH), 7.62–7.55 (m, 2 H, 2 × Ar-CH), 7.49–7.31 (m, 7 H, 7 × Ar-CH), 7.21–7.12 (m, 2 H, 2 × Ar-CH), 7.03–6.96 (m, 2 H, 2 × Ar-CH), 5.28 (d, *J* = 5.8 Hz, 2 H, C*H*HOC*H*H), 5.26 (d, *J* = 5.8 Hz, 2 H, CH*H*OCH*H*), 5.08 (s, 2 H, $PhCH_2$); ^{13}C NMR (101 MHz, $CDCl_3$) δ 157.6 (Ar-C_q-OBn), 146.7 (Ar-C_q-C_q), 145.1 (Ar-C_q-C_q), 138.5 (Ar-C_q-C_q), 137.7 (Ar-C_q-Ar), 137.5 (Ar-C_q-Ar), 136.9 (Ph-C_q), 132.6 (2 × Ar-CH), 128.6 (2 × Ar-CH), 128.0 (Ar-CH), 127.60 (2 × Ar-CH), 127.59 (2 × Ar-CH), 127.5 (2 × Ar-CH), 127.4 (2 × Ar-CH), 127.3 (2 × Ar-CH), 118.9 (Ar-C_q-CN), 114.9 (2 × Ar-CH), 84.5 (CH_2OCH_2), 70.1 ($PhCH_2$), 70.1 (CH_2*C*H_2O*C*H_2CH_2), 50.9 (C_q); FTMS (+ p NSI) m/z calcd for $C_{29}H_{27}O_2N_2^+$ $[M+H]^+$: 435.2067, Found: 435.2063.

References

[1] MKS Umetrics, *User Guide to Modde Version 11*, 2015.

[2] R. C. Huston, A. Neeley, B. L. Fayerweather, H. M. D'Arcy, F. H. Maxfield, M. M. Ballard, W. C. Lewis, *J. Am. Chem. Soc.*, 1933, **55**, 2146–2149.

[3] (a) S. Charma, D. Lacey, P. Wilson, *Molecular Crystals and Liquid Crystals*, 2003, **401**, 111. (b) M. Ebisawa, M. Ueno, Y. Oshima, Y. Kondo, *Tetrahedron Lett.*, 2007, **48**, 8918–8921.

[4] R. Sharma, S. Halder, S. Kumar, *Piramal Enterprises Ltd*. WO2014/170842 A2, 2014.

[5] D. F. Lewis, N. J. Turro, *J. Am. Chem. Soc.*, 1970, **92**, 311–320.

[6] H. Zheng, S. Ghanbari, S. Nakamura, D. G. Hall, *Angew. Chem. Int. Ed.*, 2012, **51**, 6187–6190.

[7] V. P. Liptak, W. D. Wulff, *Tetrahedron*, 2000, **56**, 10229–10247.

[8] R. Sharma, S. Halder, S. Kumar, *Piramal Enterprises Ltd*. WO2014/170842 A2, 2014.

[9] K. Nemoto, S. Tanaka, M. Konno, S. Onozawa, M. Chiba, Y. Tanaka, Y. Sasaki, R. Okubo, T. Hattori, *Tetrahedron*, 2016, **72**, 734–745.

[10] J. Hutton, W. A. Waters, *J. Chem. Soc.,* 1965, 4253–4256.

[11] S. Xu, X. Huang, X. Hong, B. Xu, *Org. Lett*. 2012, **14**, 4614–4617.

[12] J. Yan, T. Ni, F. Yan, *Tetrahedron Lett.*, 2015, **56**, 1096–1098.

[13] G. Li, E. Wang, H. Chen, H. Li, Y. Liu, P. G. Wang, *Tetrahedron*, 2008, **64**, 9033–9043.

[14] D. Ma, Q. Cai, *Org. Lett*., 2003, **5**, 3799–3802.

[15] D. Maiti, S. L. Buchwald, *J. Org. Chem.*, 2010, **75**, 1791–1794.

[16] S. Cacchi, P. G. Ciattini, E. Morera, G. Ortar, *Tetrahedron Lett.*, 1986, **27**, 5541–5544.

[17] J. P. Wolfe, H. Tomori, J. P. Sadigho, J. Yin, S. L. Buchwald, *J. Org. Chem.*, 2000, **65**, 1158–1174.

^{1}H and ^{13}C NMR spectra of selected compounds

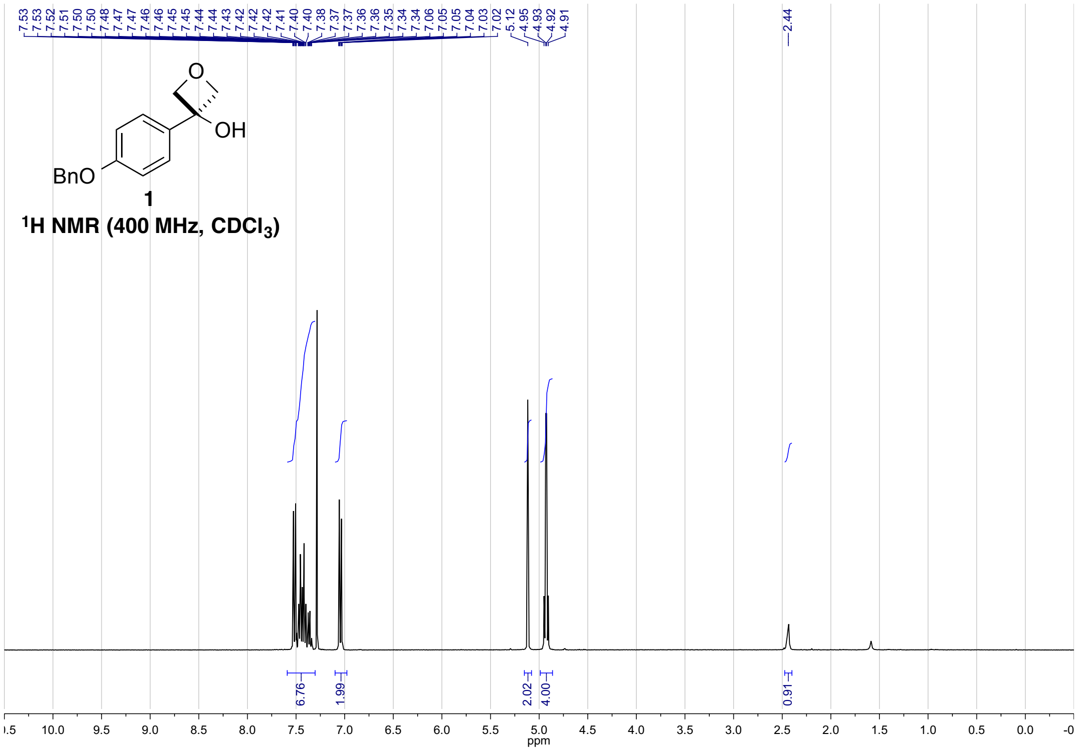
7.53 7.53 7.52 7.51 7.50 7.50 7.48 7.47 7.47 7.46 7.46 7.45 7.45 7.44 7.44 7.43 7.42 7.42 7.42 7.41 7.40 7.40 7.38 7.37 7.37 7.36 7.36 7.35 7.34 7.34 7.06 7.05 7.05 7.04 7.03 7.02 5.12 4.95 4.93 4.92 4.91
2.44
O
OH
BnO
1
^{1}H NMR (400 MHz, $CDCl_3$)
6.76 1.99 2.02 4.00 0.91
ppm

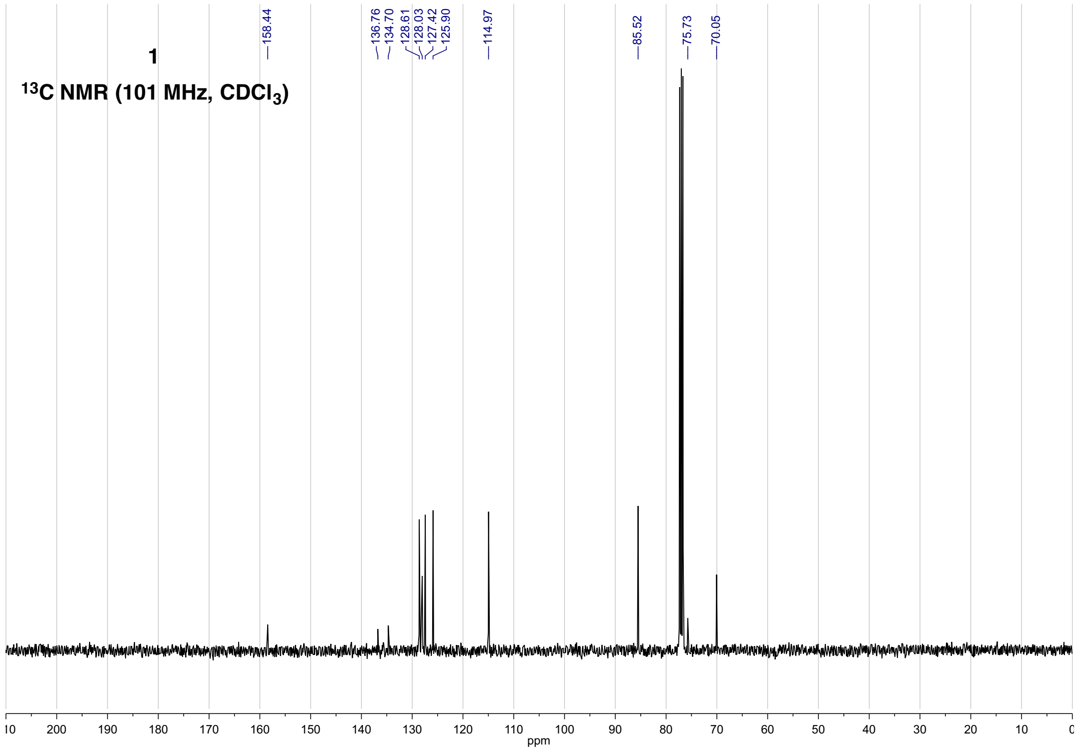
158.44
136.76 134.70 128.61 128.03 127.42 125.90
114.97
85.52
75.73
70.05
1
^{13}C NMR (101 MHz, $CDCl_3$)
ppm

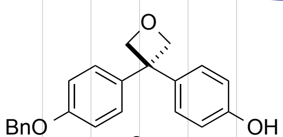

2a

^{1}H NMR (400 MHz, CDCl$_3$)

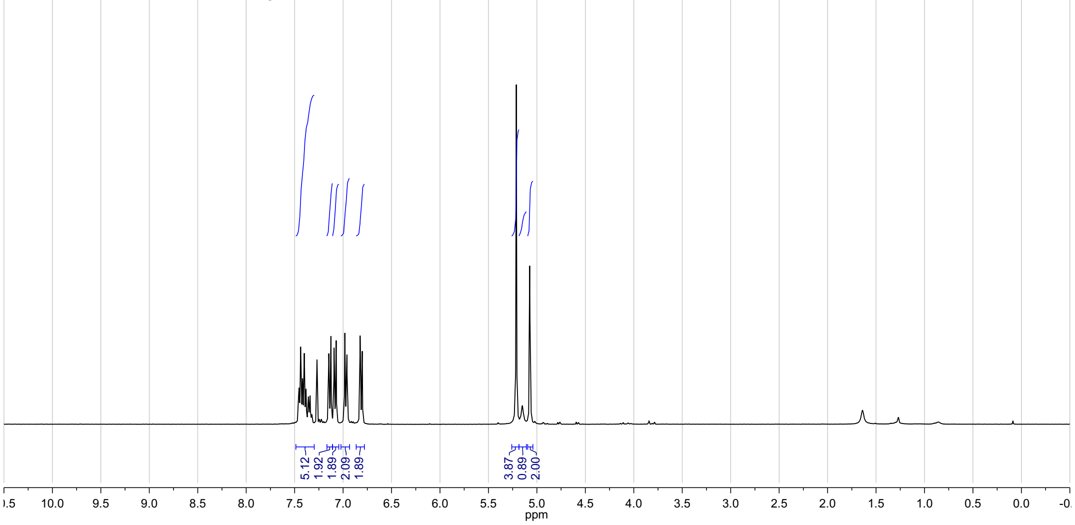

2a

^{13}C NMR (101 MHz, CDCl$_3$)

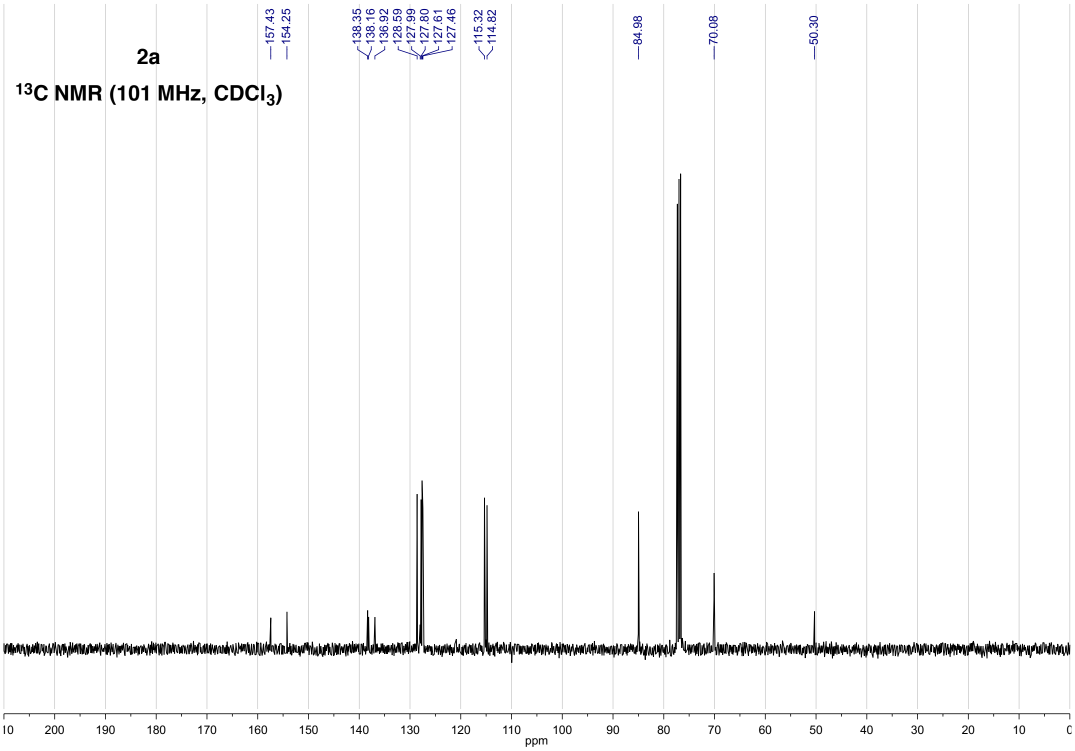

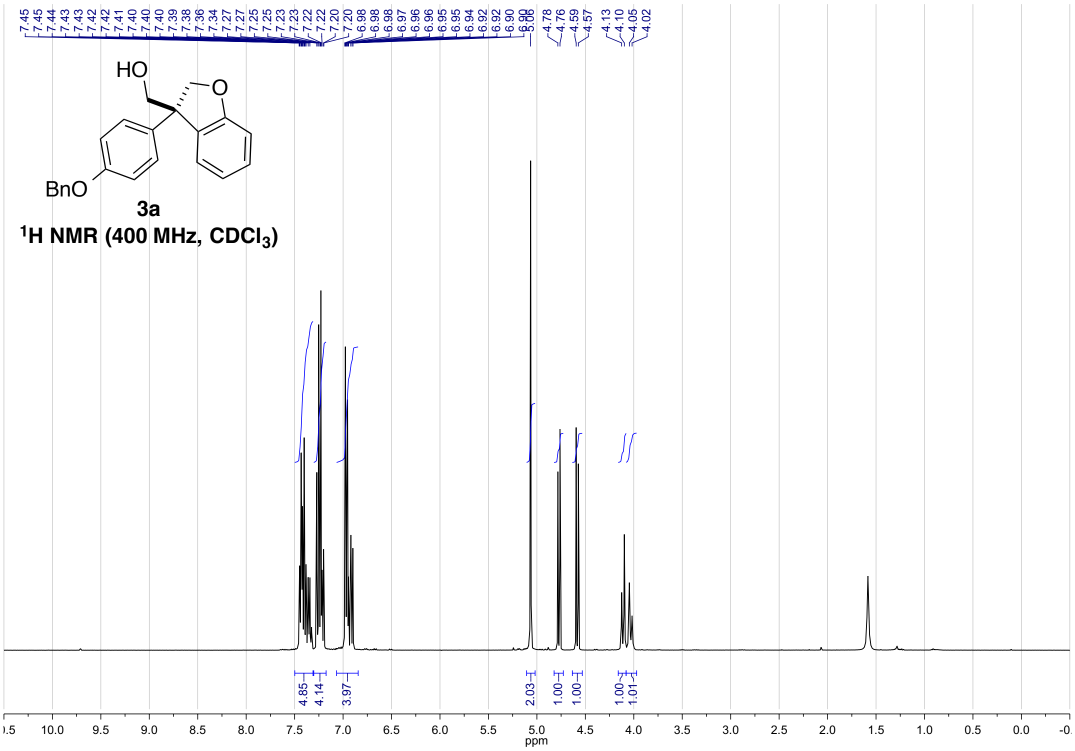
HO
O
BnO
3a
^{1}H NMR (400 MHz, CDCl$_3$)
ppm

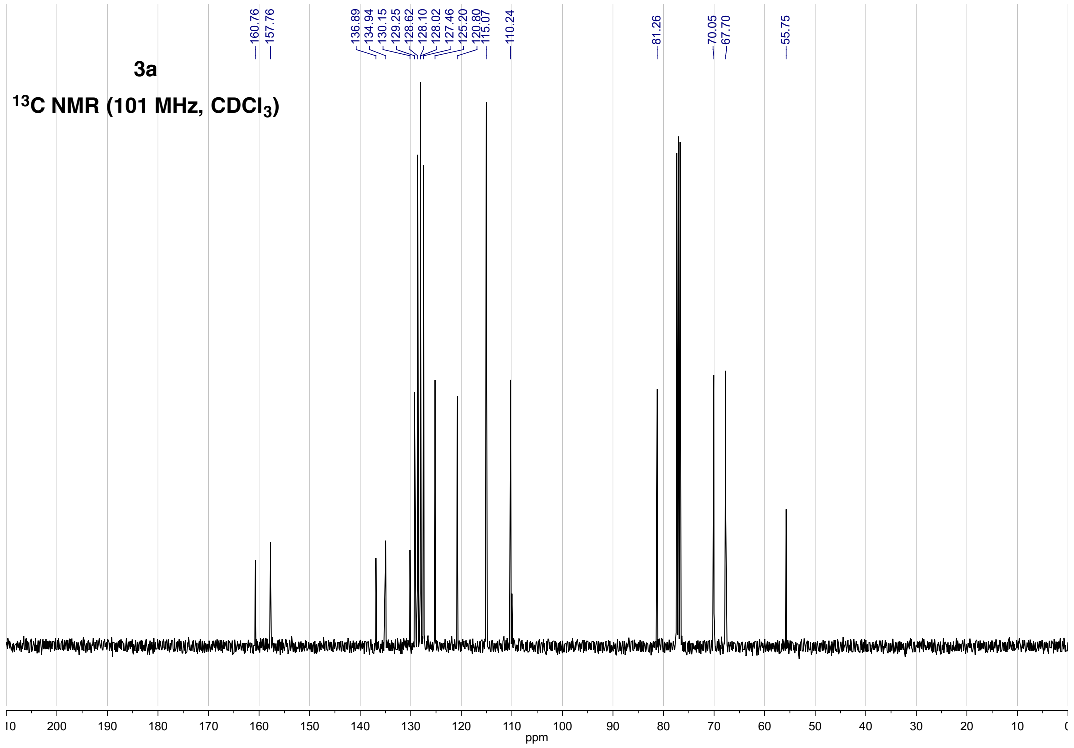
3a
^{13}C NMR (101 MHz, CDCl$_3$)
160.76
157.76
136.89
134.94
130.15
129.25
128.62
128.10
128.02
127.46
125.20
120.80
115.07
110.24
81.26
70.05
67.70
55.75
ppm

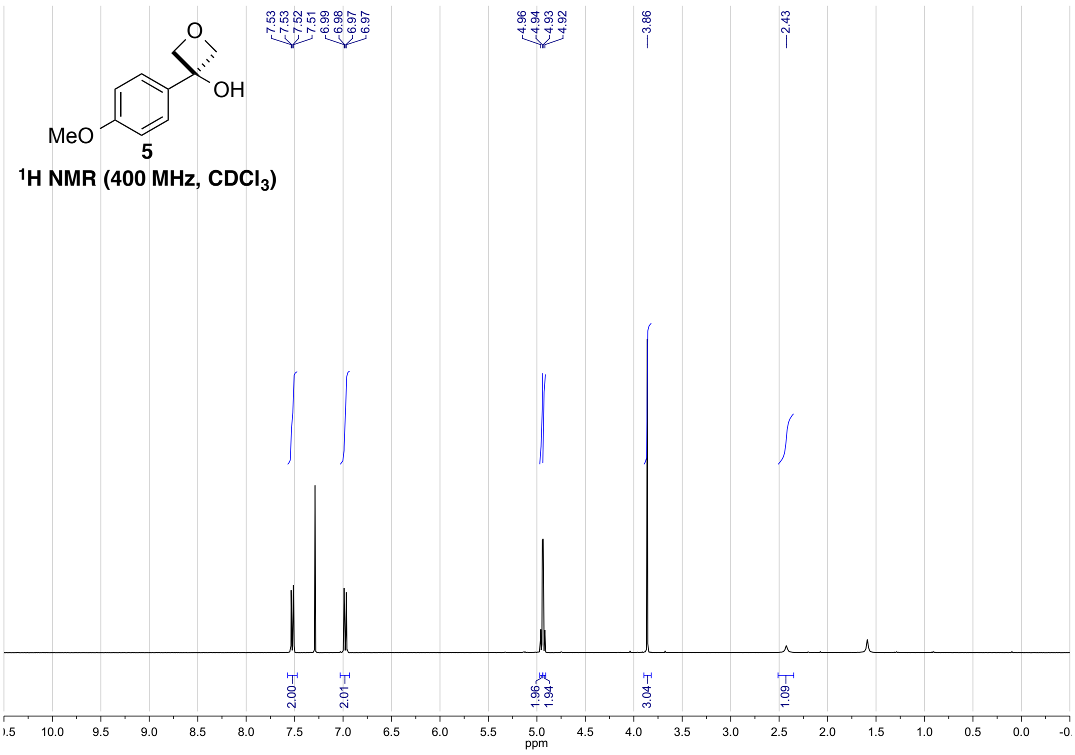
MeO
OH
O
5
1H NMR (400 MHz, CDCl3)
7.53
7.53
7.52
7.51
6.99
6.98
6.97
6.97
4.96
4.94
4.93
4.92
3.86
2.43
2.00
2.01
1.96
1.94
3.04
1.09
ppm

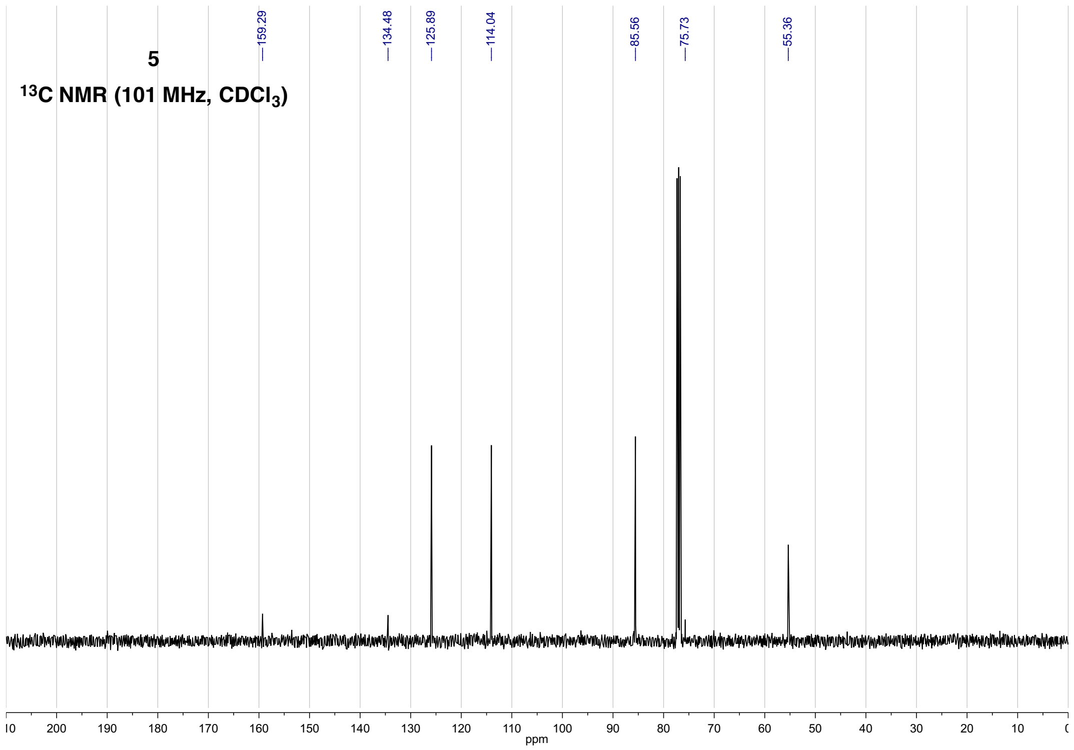
5
13C NMR (101 MHz, CDCl3)
159.29
134.48
125.89
114.04
85.56
75.73
55.36
ppm

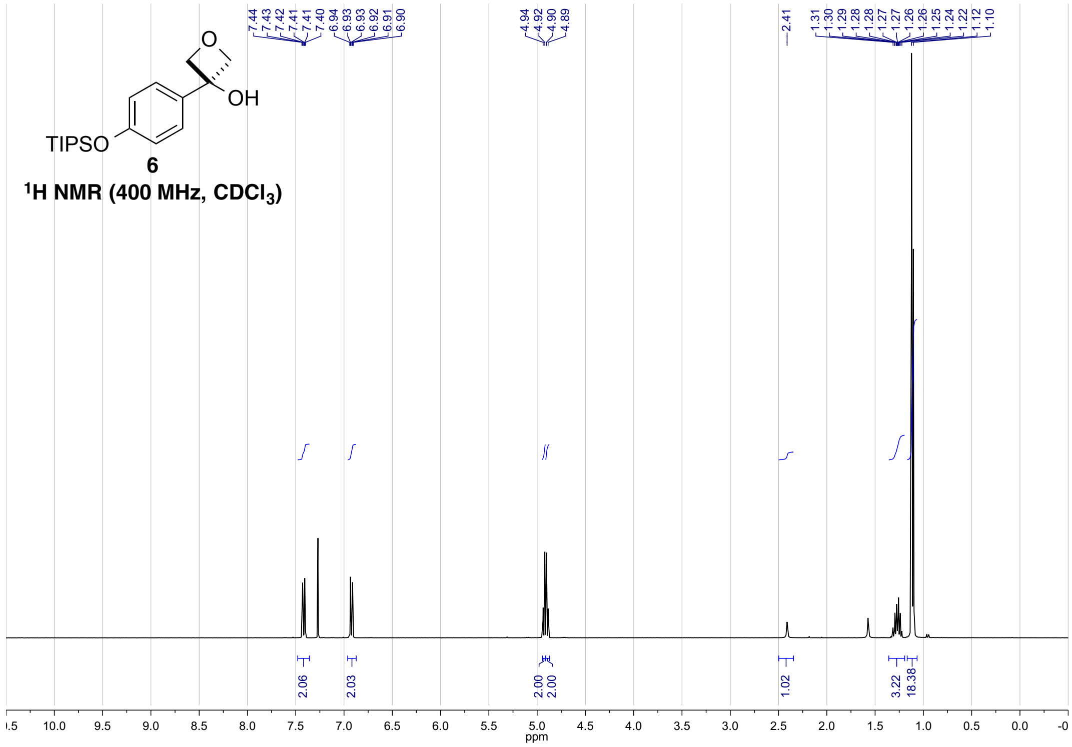
TIPSO
OH
O
6
^{1}H NMR (400 MHz, $CDCl_3$)
ppm

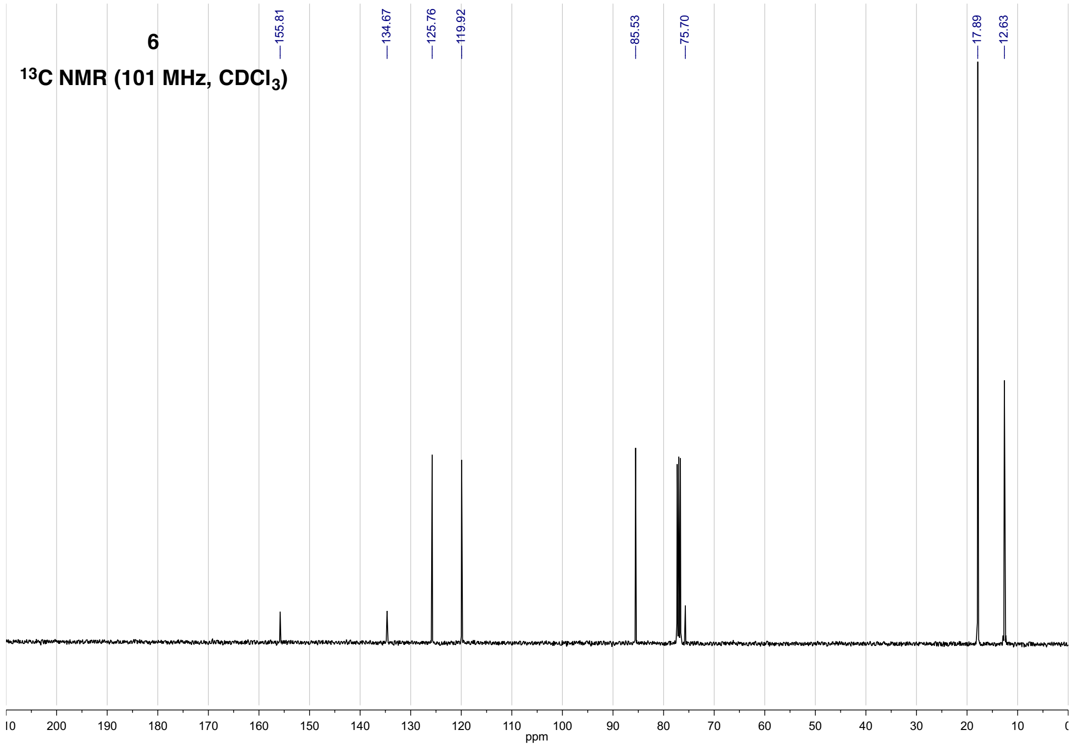
6
^{13}C NMR (101 MHz, $CDCl_3$)
ppm

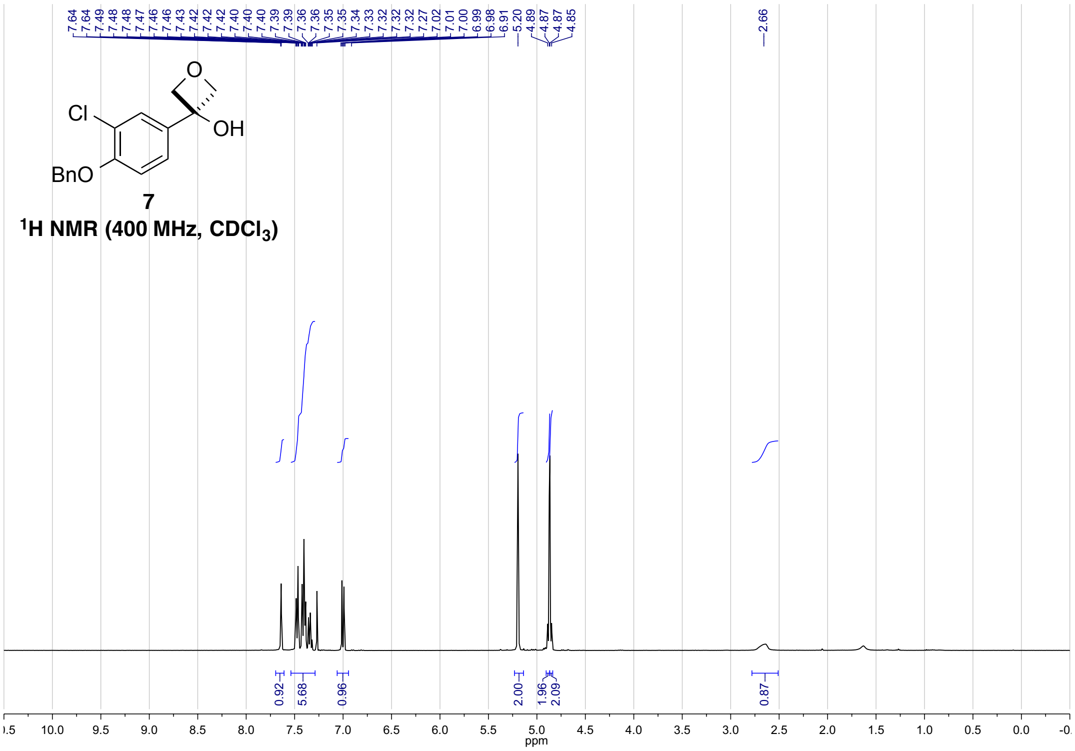
7.64
7.64
7.49
7.48
7.48
7.47
7.46
7.46
7.43
7.42
7.42
7.42
7.40
7.40
7.40
7.39
7.39
7.36
7.36
7.35
7.35
7.34
7.33
7.32
7.32
7.32
7.27
7.02
7.01
7.00
6.99
6.98
6.91
5.20
4.89
4.87
4.87
4.85
2.66
Cl
O
OH
BnO
7
¹H NMR (400 MHz, CDCl3)
0.92
5.68
0.96
2.00
1.96
2.09
0.87
ppm

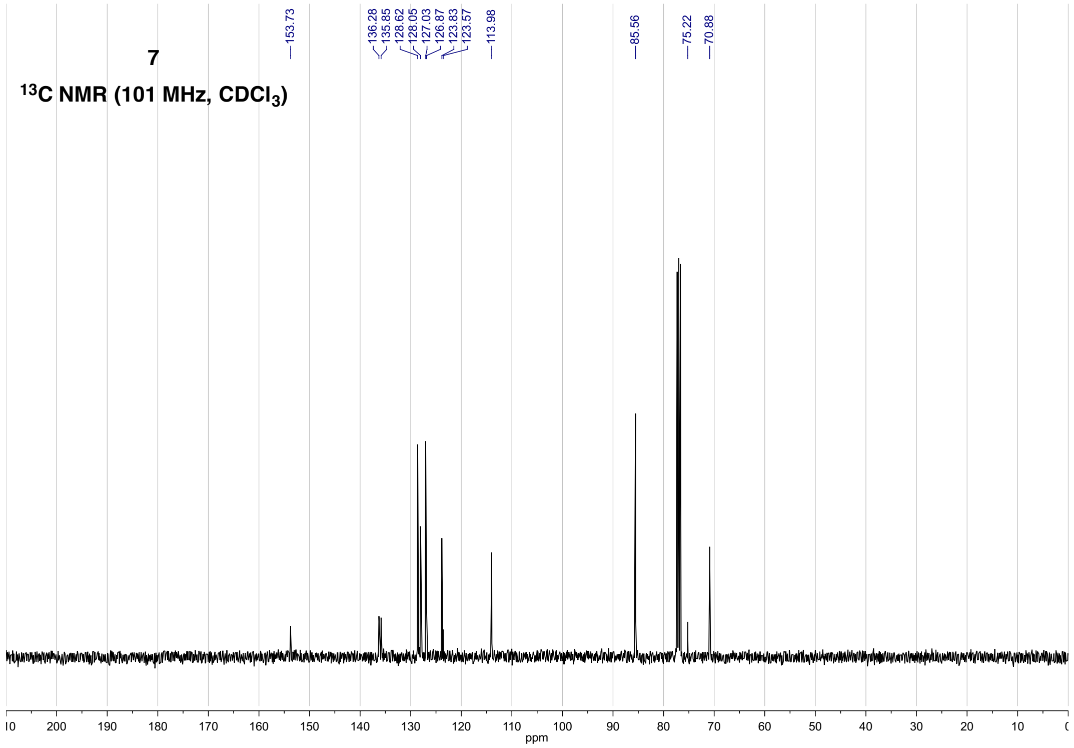
153.73
136.28
135.85
128.62
128.05
127.03
126.87
123.83
123.57
113.98
85.56
75.22
70.88
7
¹³C NMR (101 MHz, CDCl3)
ppm

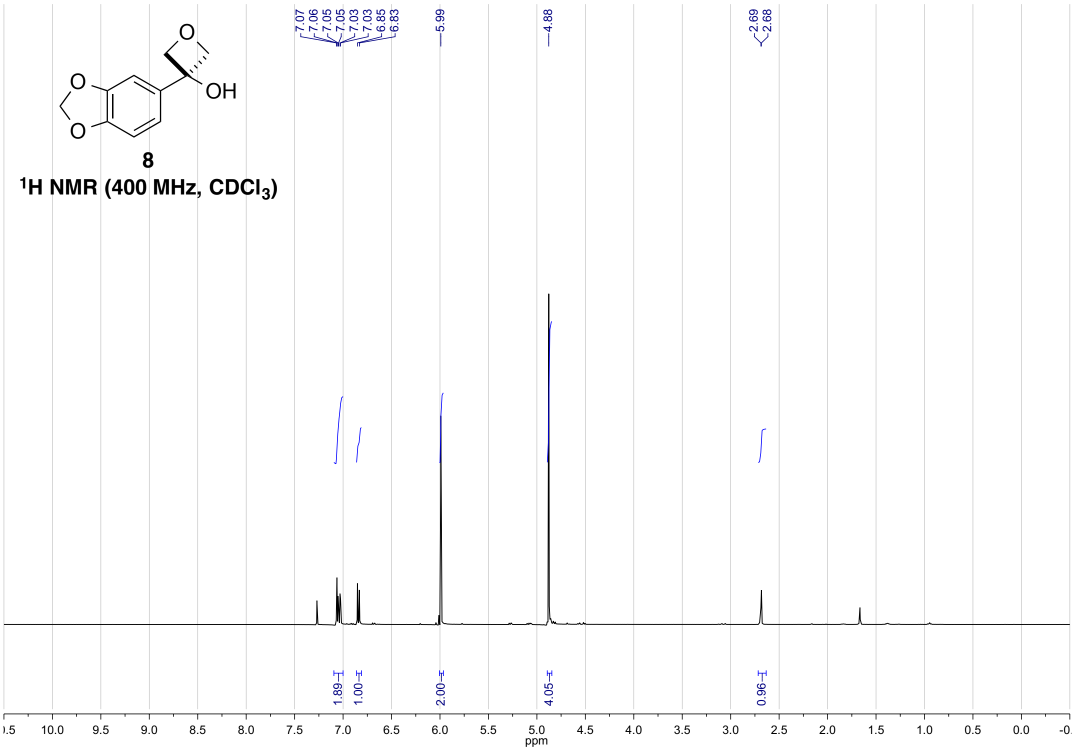
8
^{1}H NMR (400 MHz, $CDCl_3$)
7.07 7.06 7.05 7.05 7.03 7.03 6.85 6.83
5.99
4.88
2.69 2.68
1.89 1.00 2.00 4.05 0.96
ppm

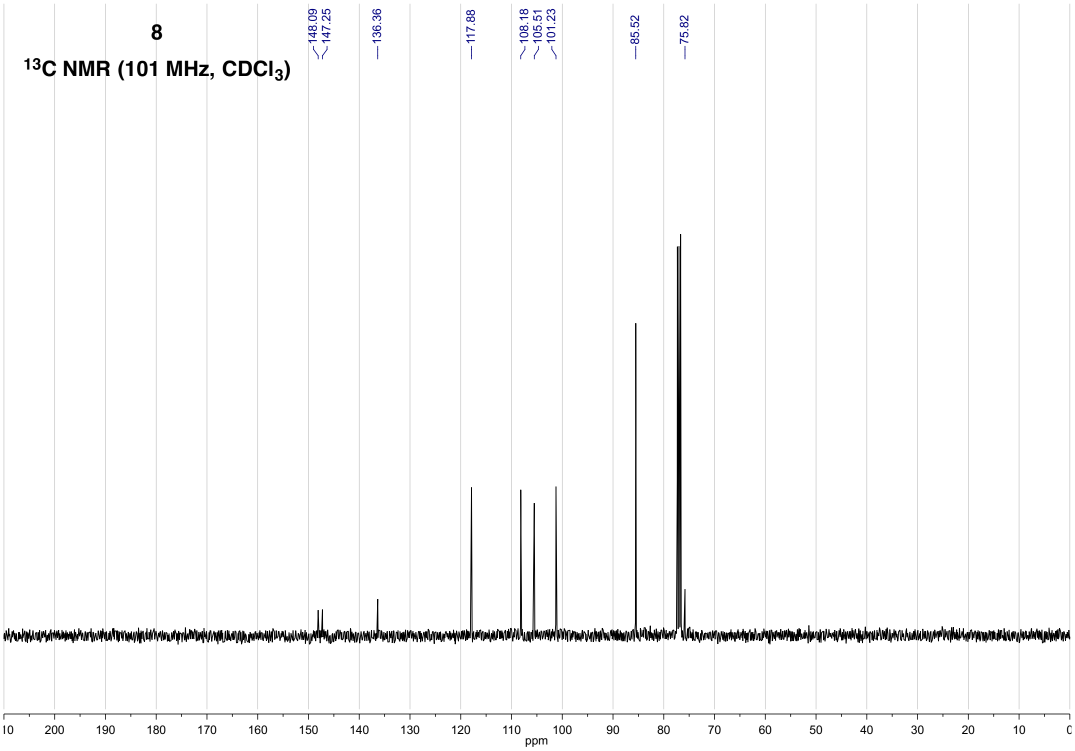
8
^{13}C NMR (101 MHz, $CDCl_3$)
148.09 147.25
136.36
117.88
108.18 105.51 101.23
85.52
75.82
ppm

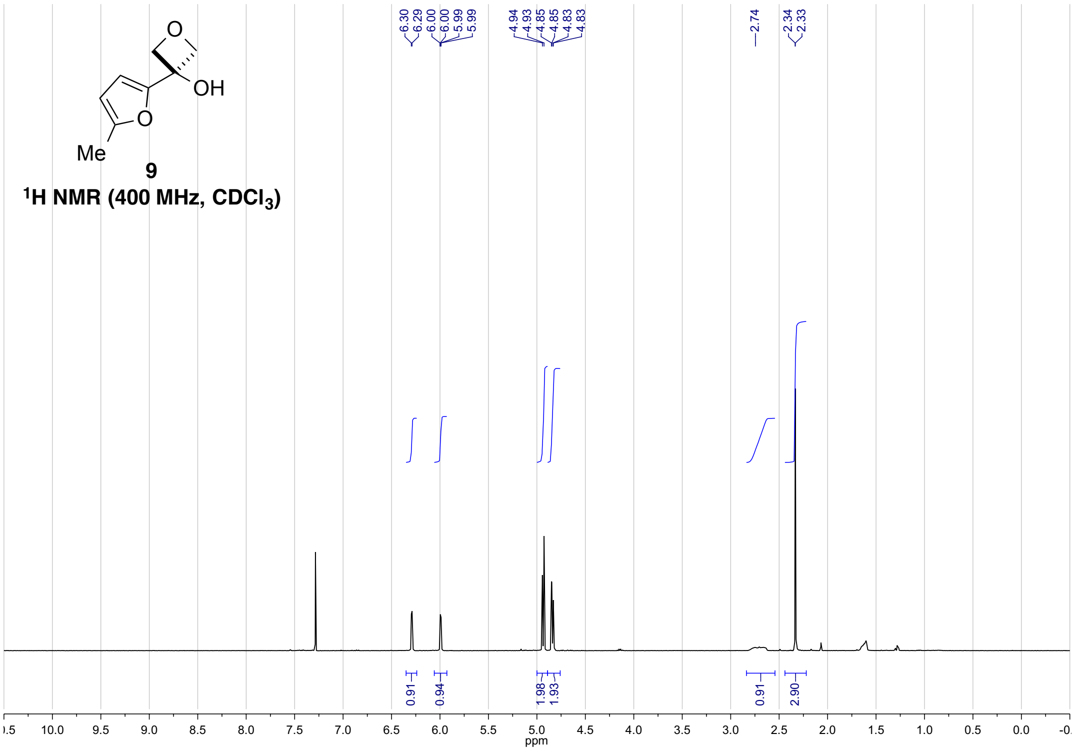
Me
9
1H NMR (400 MHz, CDCl3)
ppm

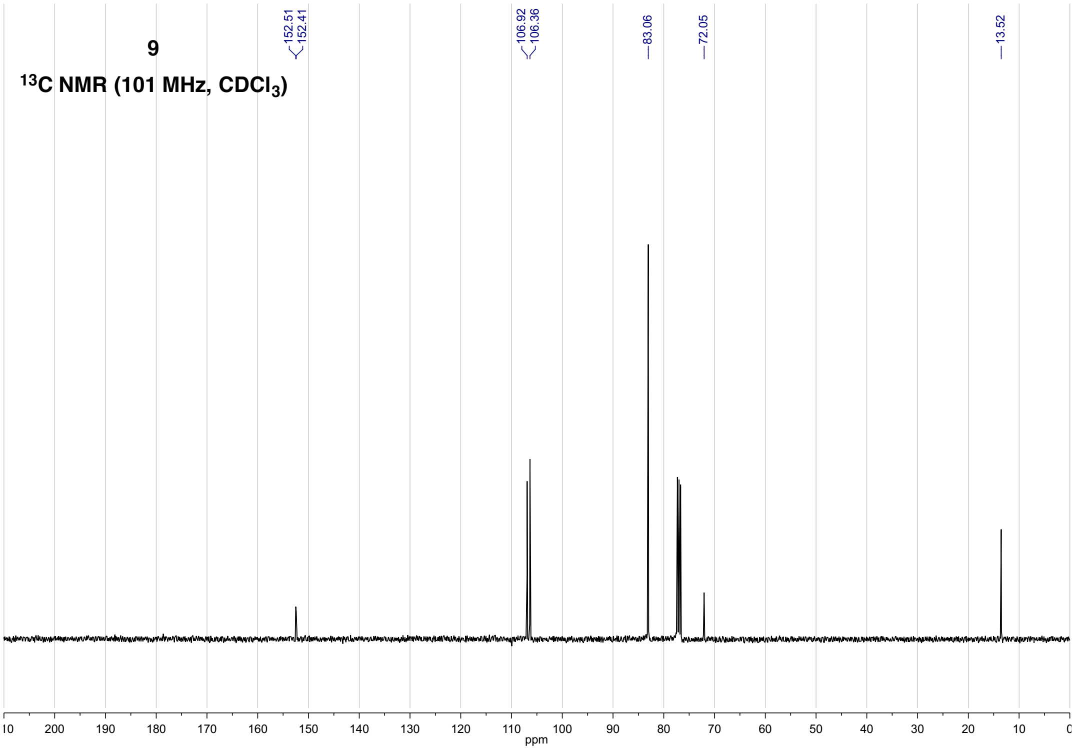
9
13C NMR (101 MHz, CDCl3)
ppm

10

^{1}H NMR (400 MHz, $CDCl_3$)

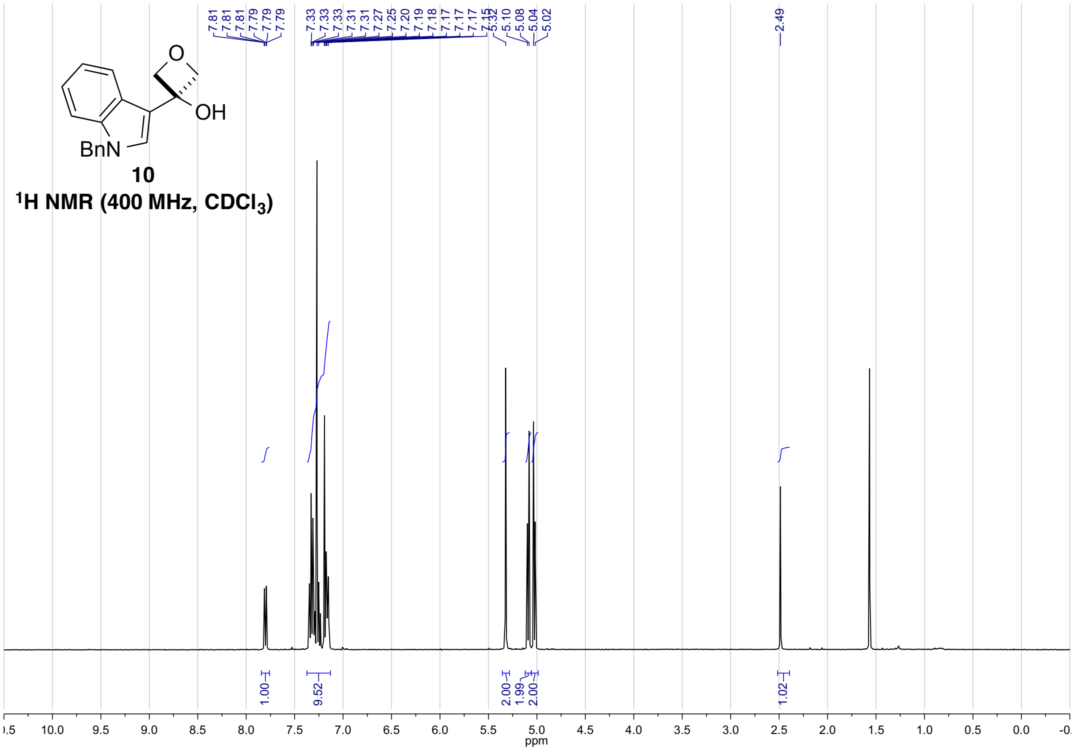

10

^{13}C NMR (101 MHz, $CDCl_3$)

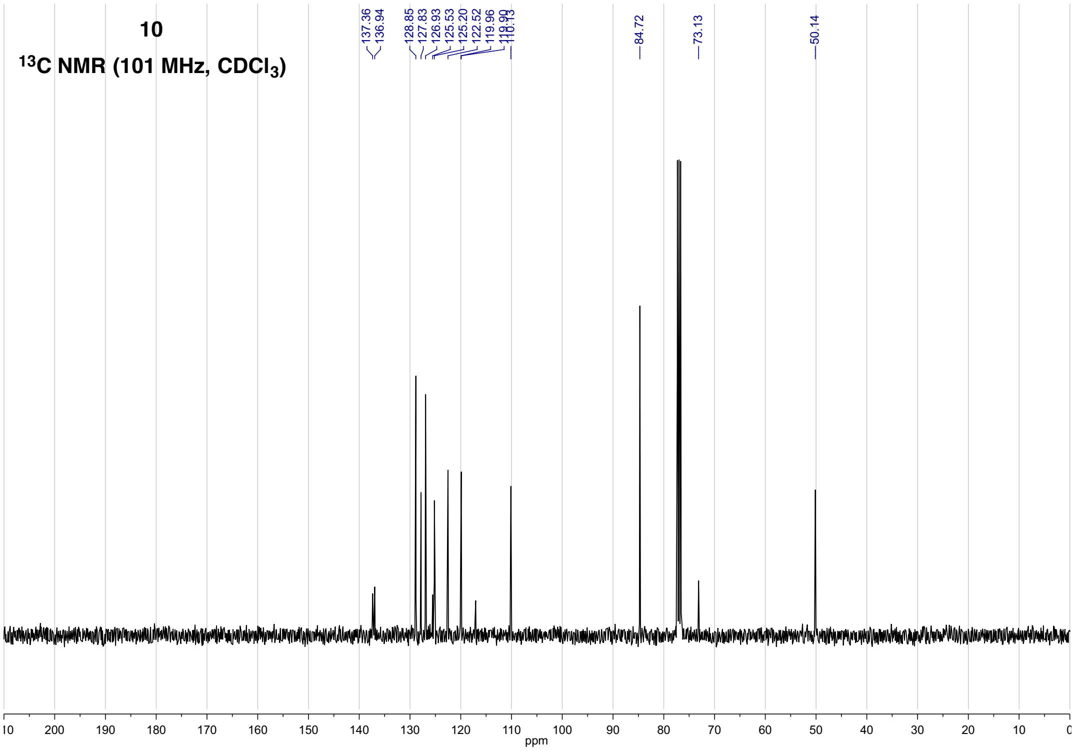

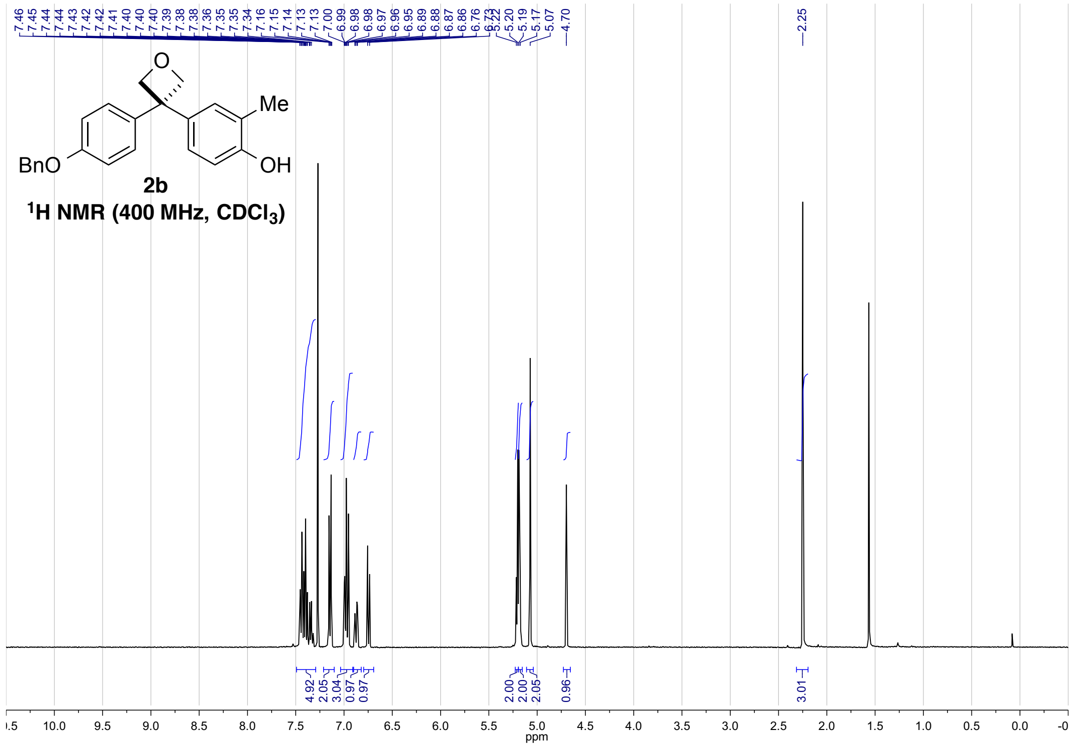
2b
1H NMR (400 MHz, CDCl3)
ppm

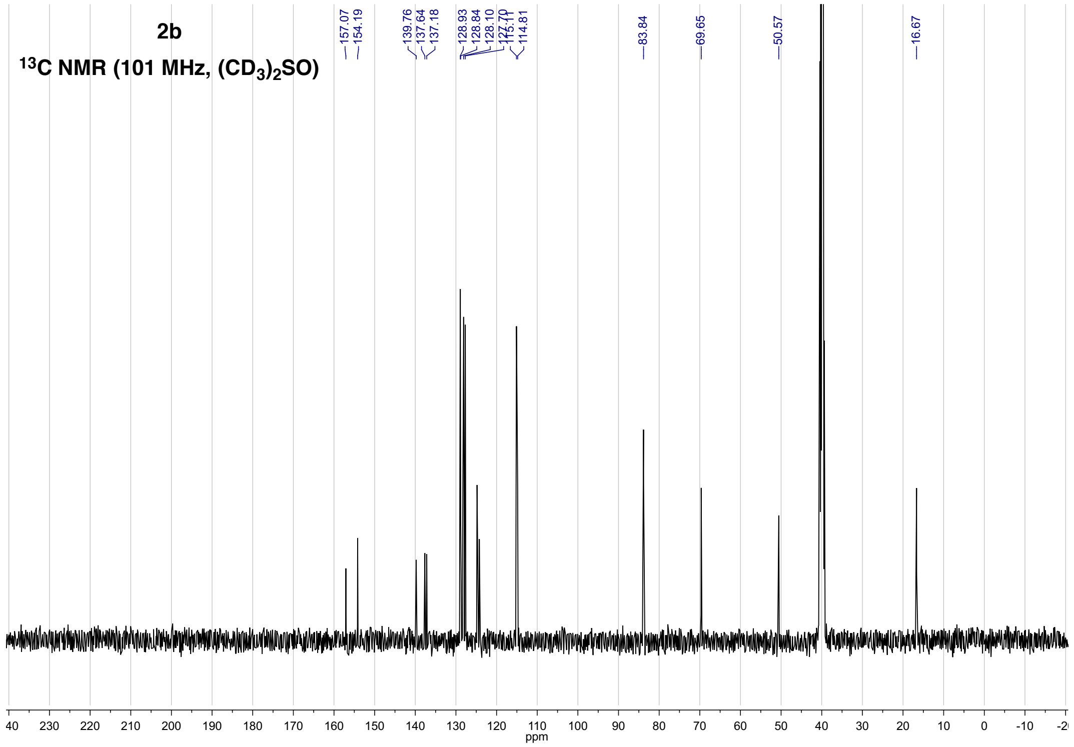
2b
13C NMR (101 MHz, (CD3)2SO)
ppm

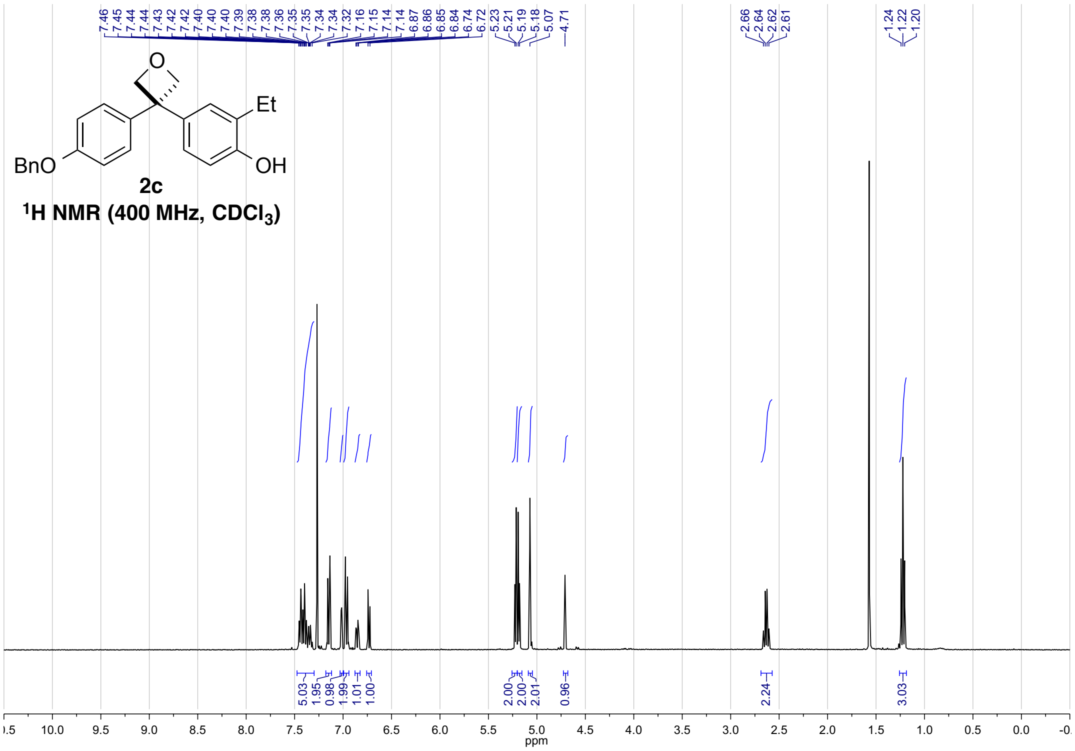
BnO
OH
Et
O
2c
¹H NMR (400 MHz, CDCl₃)
ppm

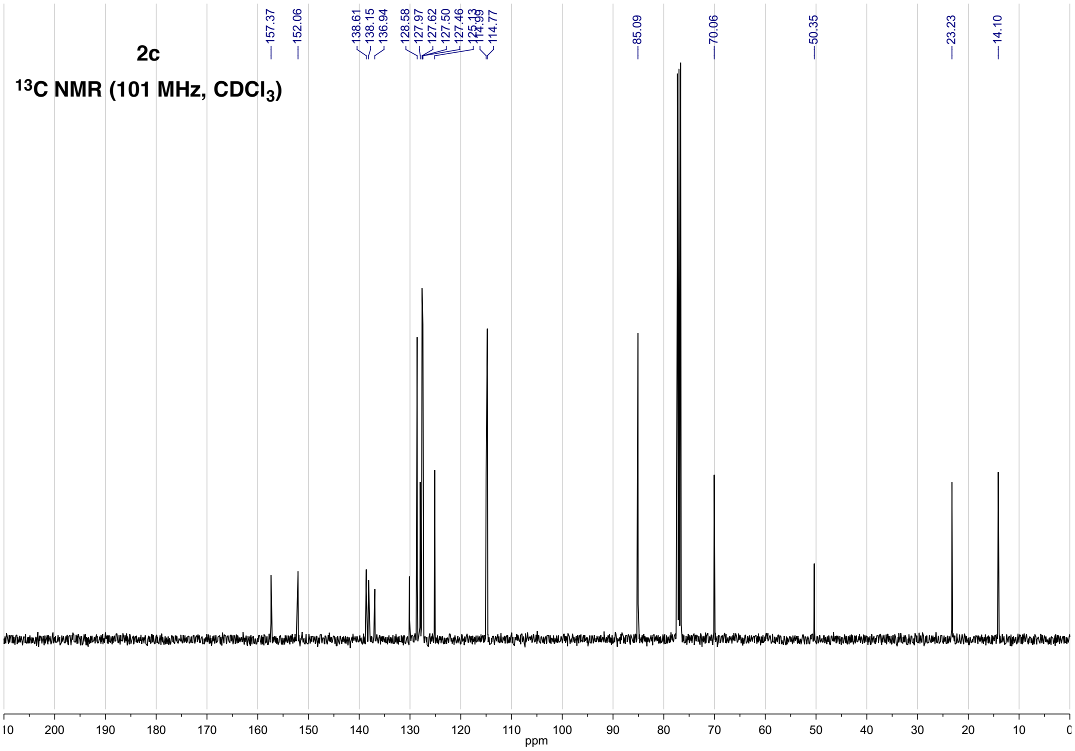
2c
¹³C NMR (101 MHz, CDCl₃)
ppm

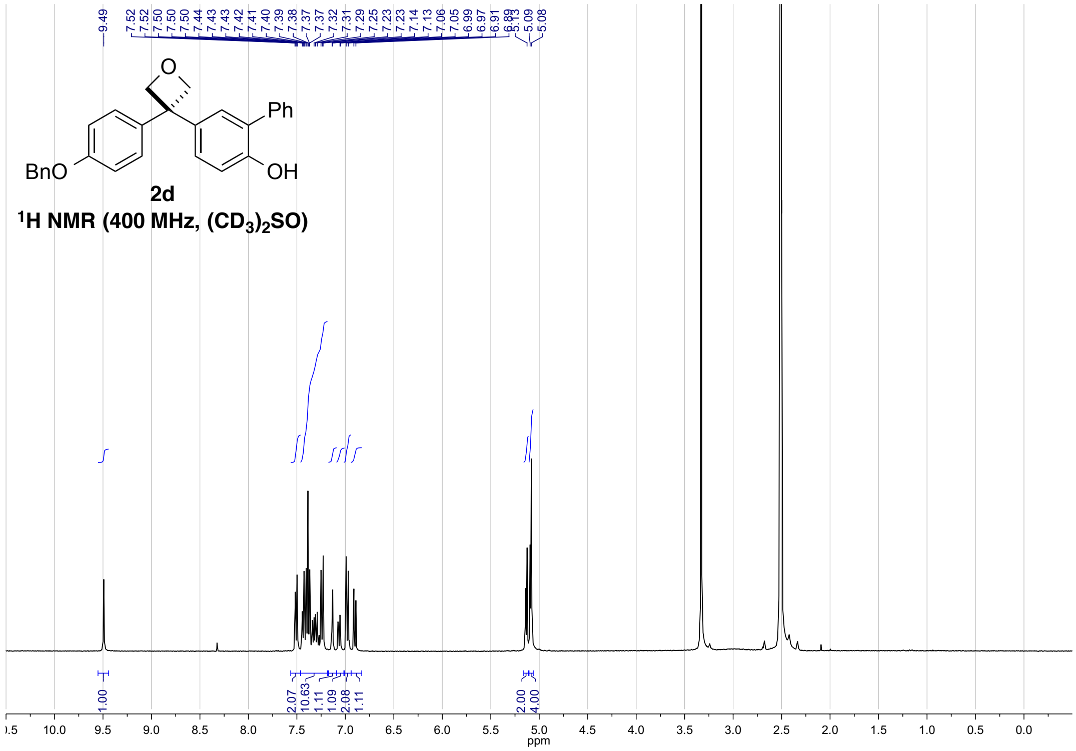
BnO
Ph
OH
O
2d
^{1}H NMR (400 MHz, $(CD_3)_2SO$)
9.49
7.52 7.52 7.50 7.50 7.50 7.44 7.43 7.43 7.42 7.41 7.40 7.39 7.38 7.37 7.37 7.32 7.31 7.29 7.25 7.23 7.23 7.14 7.13 7.06 7.05 6.99 6.97 6.91 6.89
5.13 5.09 5.08
1.00
2.07
10.63
1.11
1.09
2.08
1.11
2.00
4.00
ppm

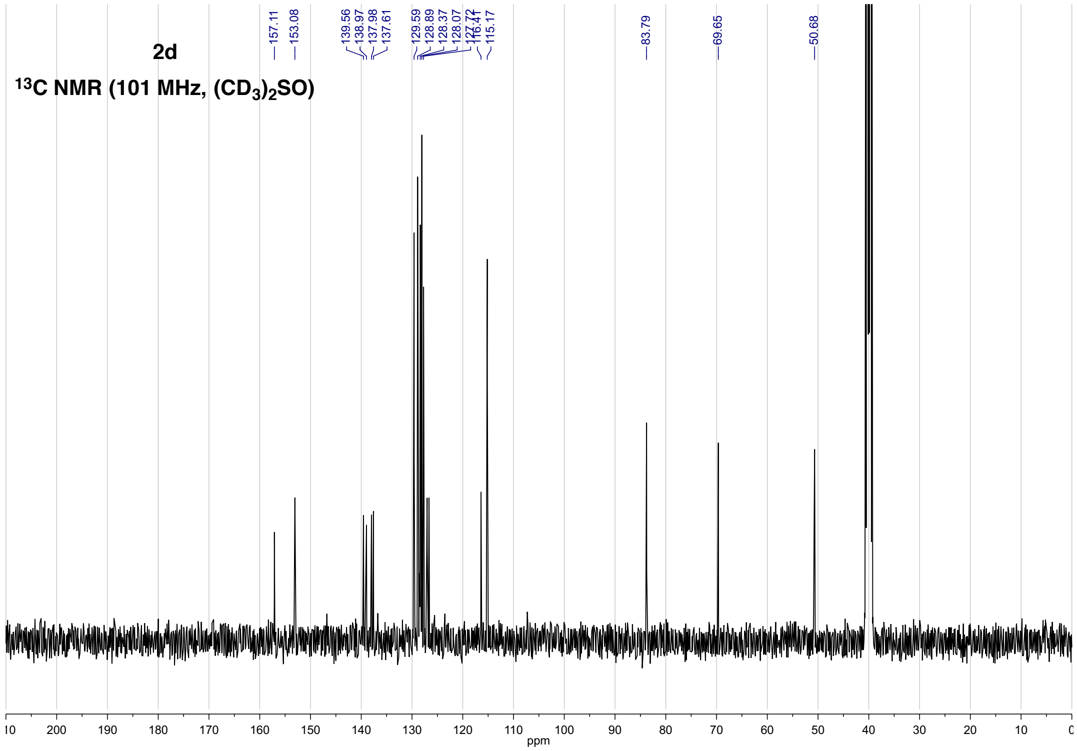
2d
^{13}C NMR (101 MHz, $(CD_3)_2SO$)
157.11
153.08
139.56 138.97 137.98 137.61
129.59 128.89 128.37 128.07 127.12 126.41 116.41 115.17
83.79
69.65
50.68
ppm

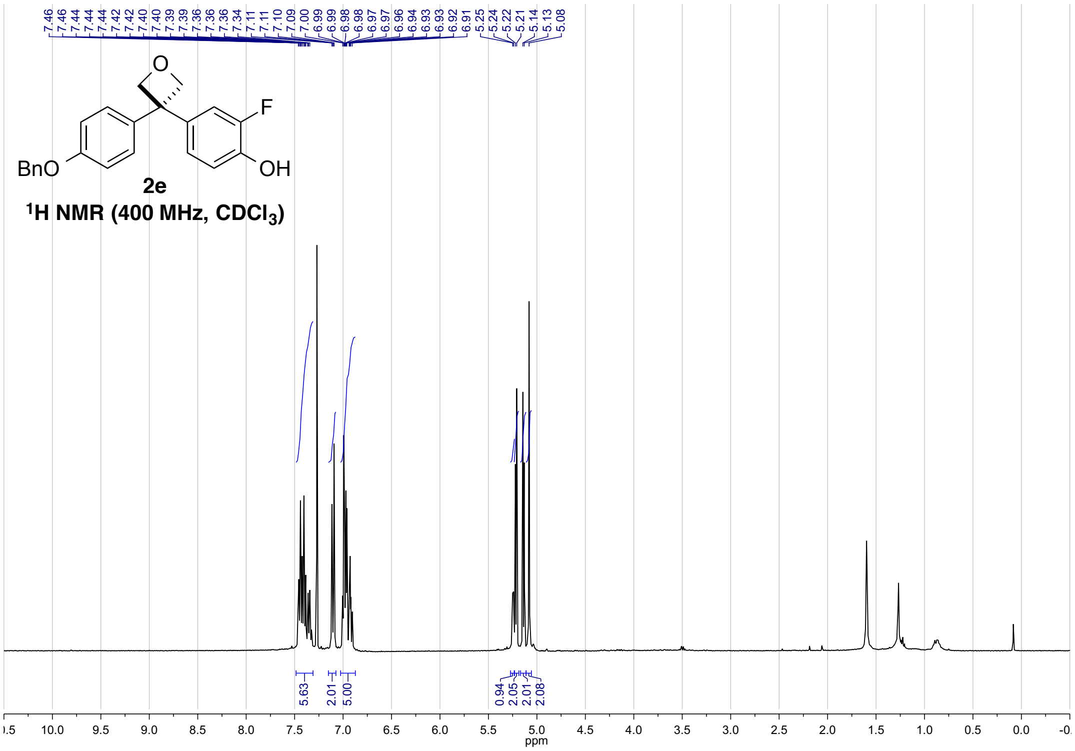
BnO
F
OH
O
2e
^{1}H NMR (400 MHz, CDCl$_3$)
ppm

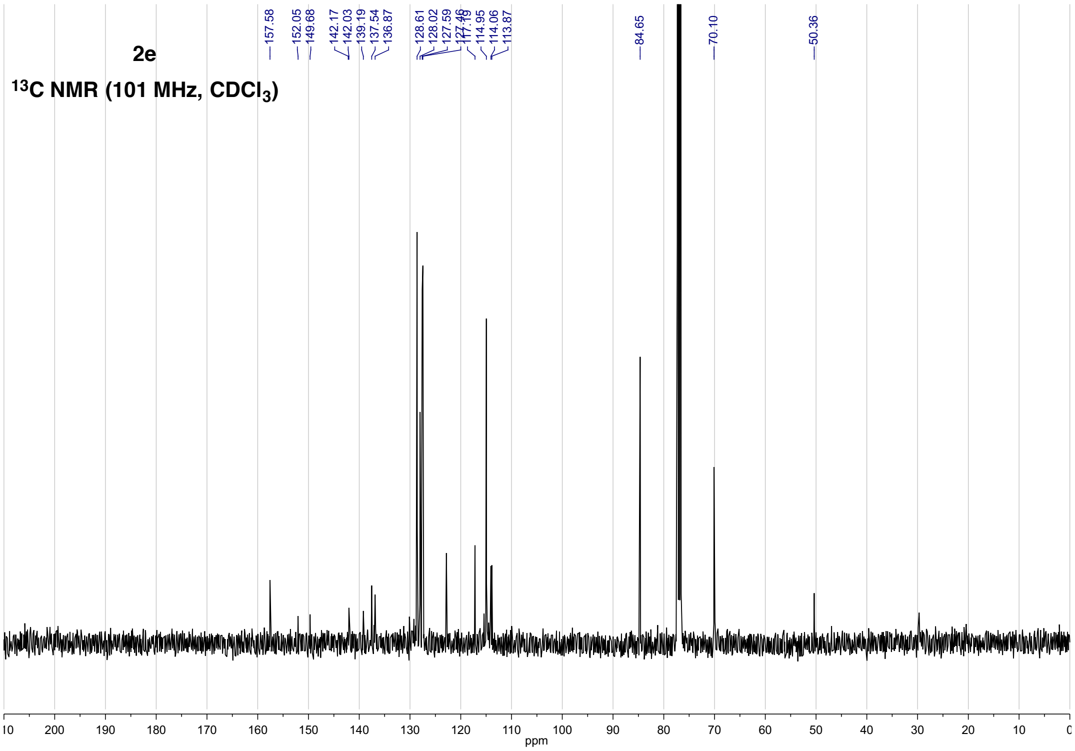
2e
^{13}C NMR (101 MHz, CDCl$_3$)
157.58
152.05
149.68
142.17
142.03
139.19
137.54
136.87
128.61
128.02
127.59
114.95
114.06
113.87
84.65
70.10
50.36
ppm

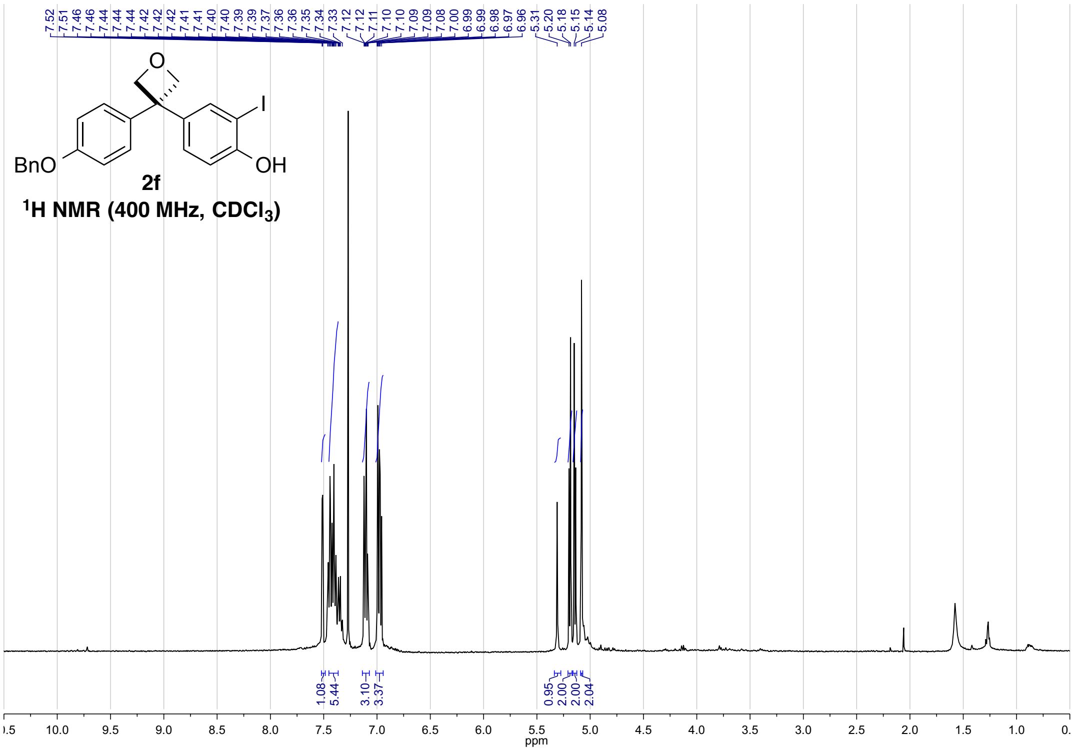
BnO
OH
I
O
2f
1H NMR (400 MHz, CDCl3)
ppm

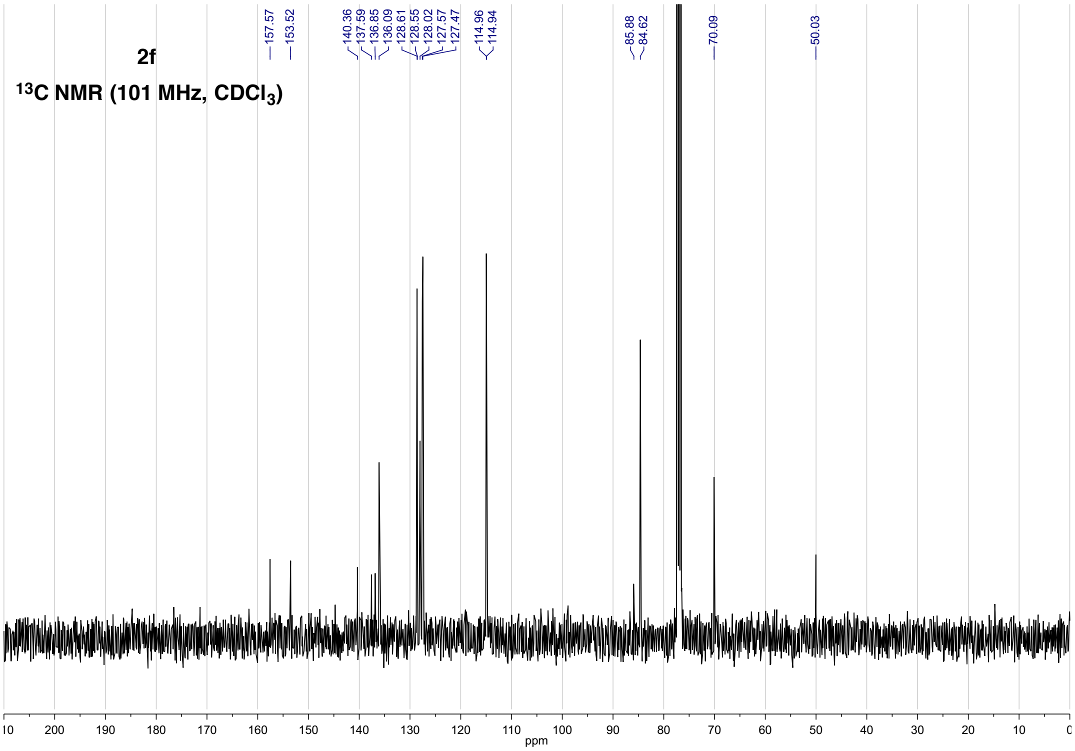
2f
13C NMR (101 MHz, CDCl3)
157.57
153.52
140.36
137.59
136.85
136.09
128.61
128.55
128.02
127.57
127.47
114.96
114.94
85.88
84.62
70.09
50.03
ppm

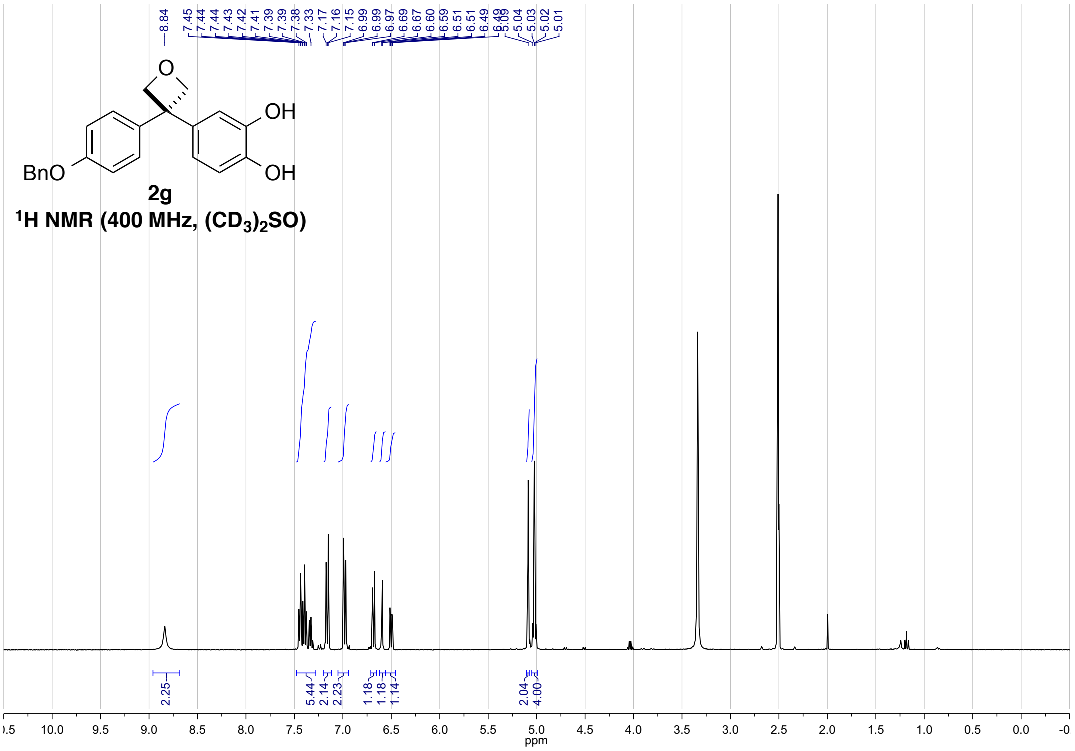
OH
OH
O
BnO
2g
^{1}H NMR (400 MHz, $(CD_3)_2SO$)
8.84
7.45
7.44
7.43
7.42
7.41
7.39
7.39
7.38
7.33
7.17
7.16
7.15
6.99
6.99
6.97
6.69
6.67
6.60
6.59
6.51
6.51
6.49
5.04
5.03
5.02
5.01
2.25
5.44
2.14
2.23
1.18
1.18
1.14
2.04
4.00
ppm

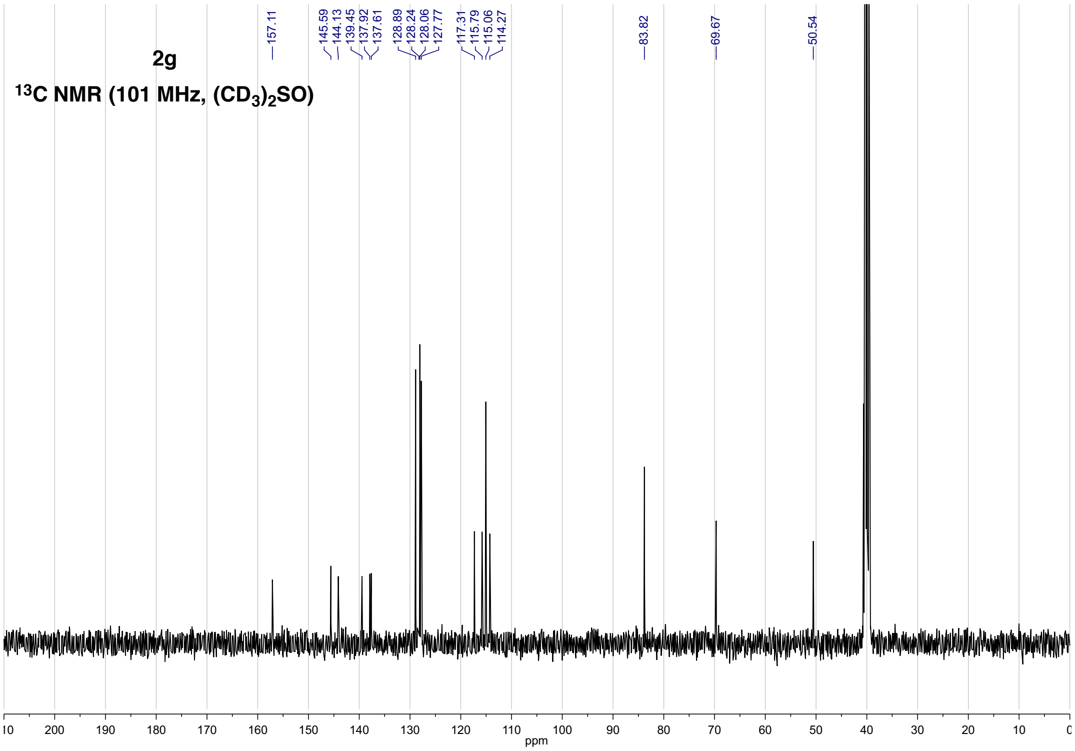
2g
^{13}C NMR (101 MHz, $(CD_3)_2SO$)
157.11
145.59
144.13
139.45
137.92
137.61
128.89
128.24
128.06
127.77
117.31
115.79
115.06
114.27
83.82
69.67
50.54
ppm

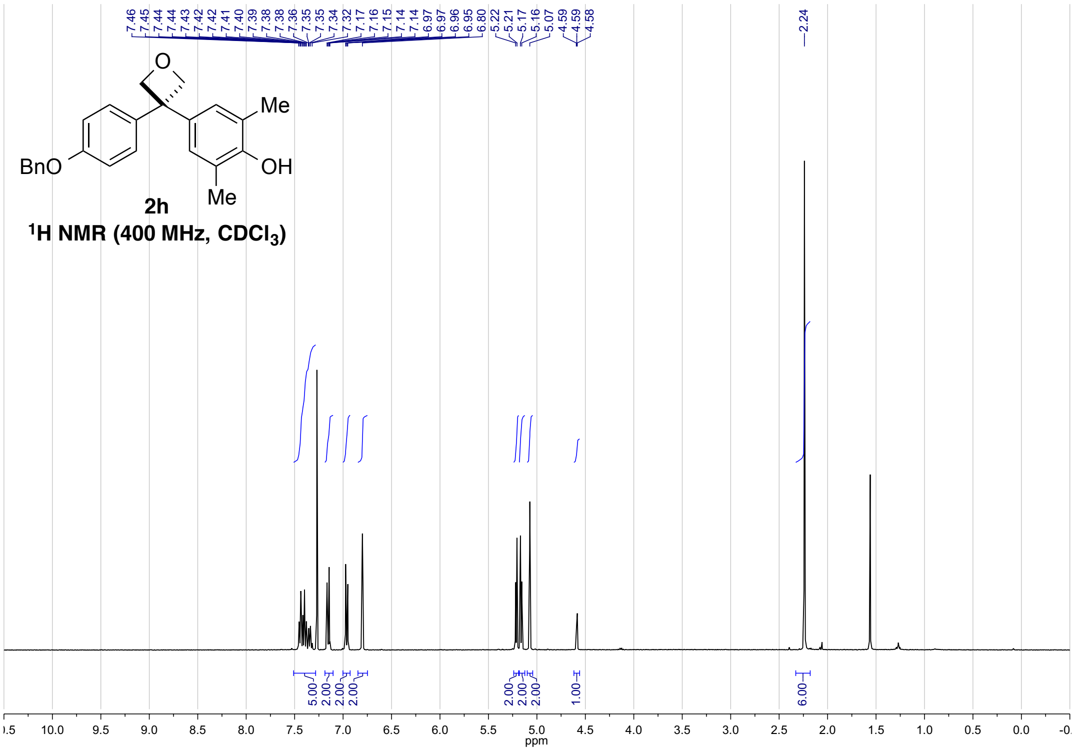
7.46 7.45 7.44 7.44 7.43 7.42 7.42 7.41 7.40 7.39 7.38 7.38 7.36 7.35 7.35 7.34 7.32 7.17 7.16 7.15 7.14 7.14 6.97 6.97 6.96 6.95 6.80 5.22 5.21 5.17 5.16 5.07 4.59 4.59 4.58
2.24
O
Me
BnO
OH
Me
2h
1H NMR (400 MHz, CDCl3)
5.00 2.00 2.00 2.00
2.00 2.00 2.00
1.00
6.00
ppm

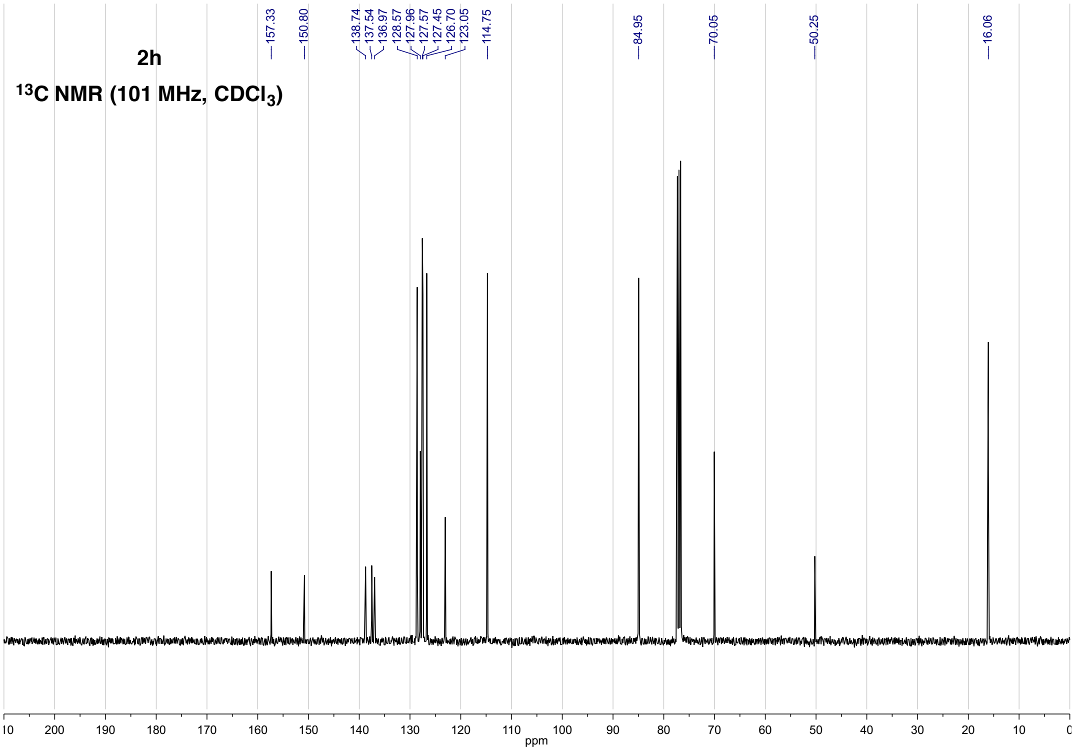
157.33
150.80
138.74 137.54 136.97 128.57 127.96 127.57 127.45 126.70 123.05
114.75
84.95
70.05
50.25
16.06
2h
13C NMR (101 MHz, CDCl3)
ppm

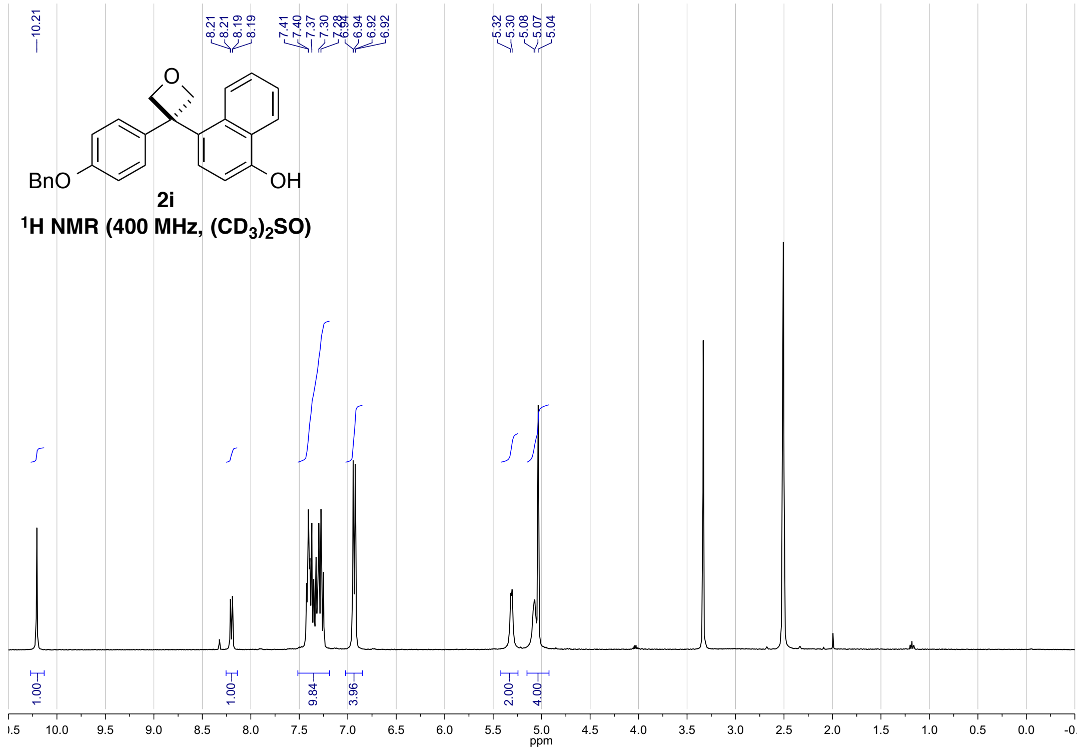

2i

^{1}H NMR (400 MHz, $(CD_3)_2SO$)

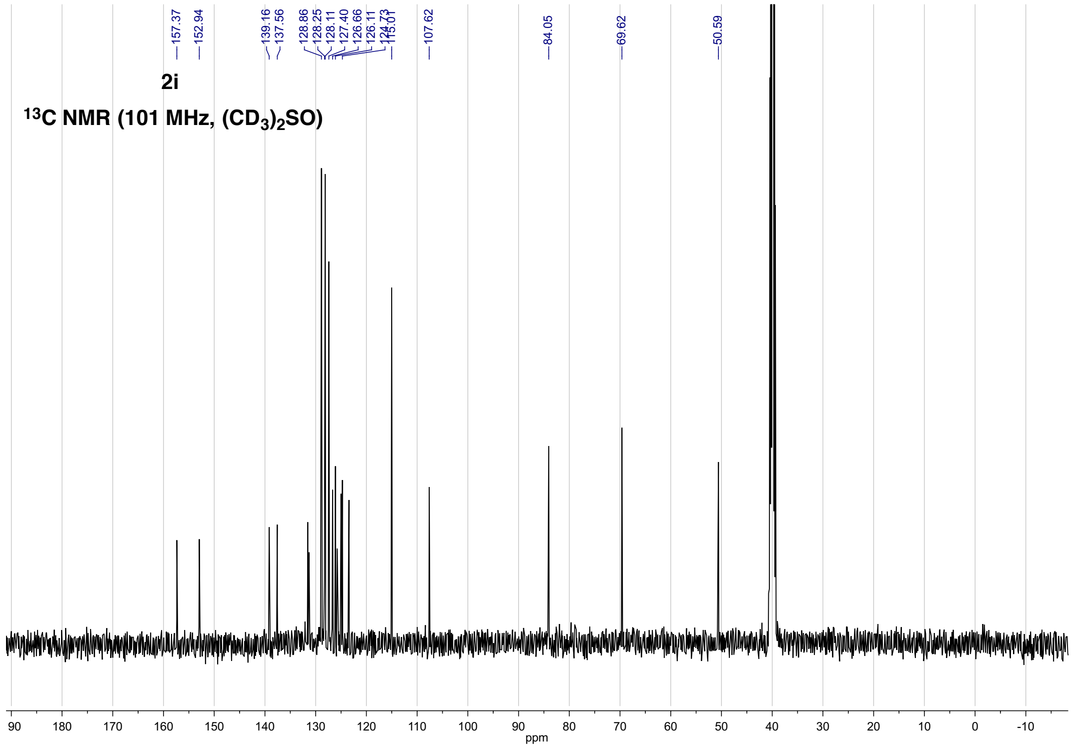

2i

^{13}C NMR (101 MHz, $(CD_3)_2SO$)

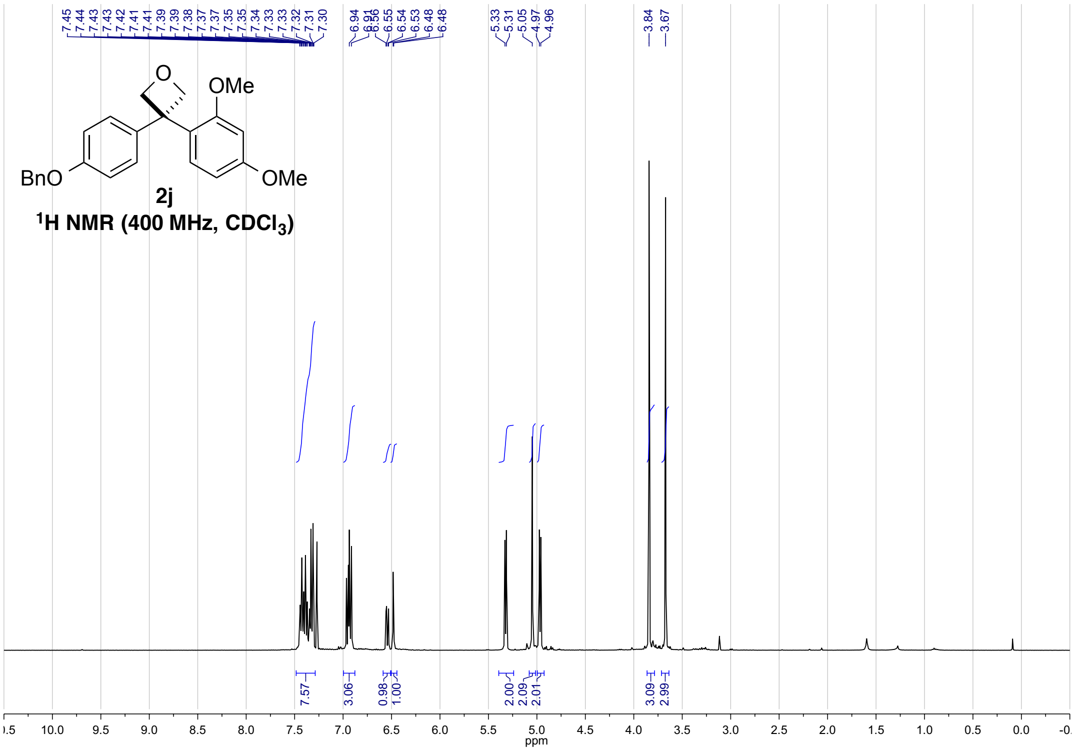
7.45 7.44 7.43 7.42 7.41 7.41 7.39 7.39 7.38 7.37 7.37 7.35 7.35 7.34 7.33 7.33 7.32 7.31 7.30
6.94 6.91 6.56 6.55 6.54 6.53 6.48 6.48
5.33 5.31 5.05 4.97 4.96
3.84 3.67
OMe
OMe
BnO
2j
¹H NMR (400 MHz, CDCl₃)
7.57 3.06 0.98 1.00 2.00 2.09 2.01 3.09 2.99
ppm

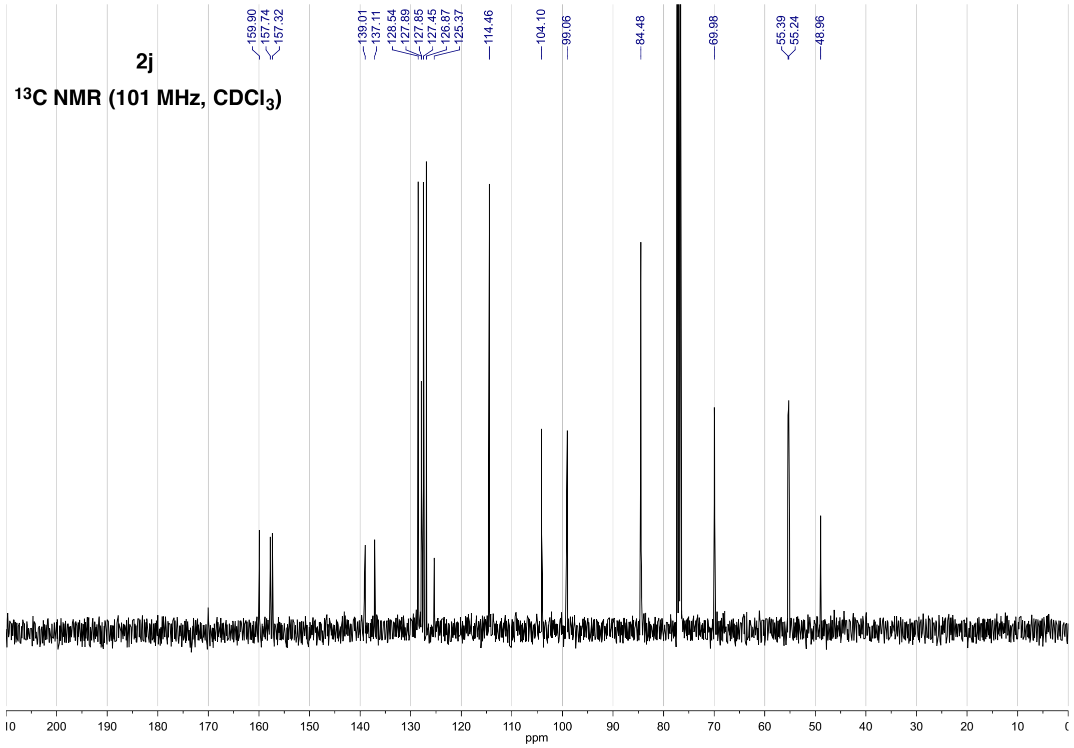
159.90 157.74 157.32 139.01 137.11 128.54 127.89 127.85 127.45 126.87 125.37 114.46 104.10 99.06 84.48 69.98 55.39 55.24 48.96
2j
¹³C NMR (101 MHz, CDCl₃)
ppm

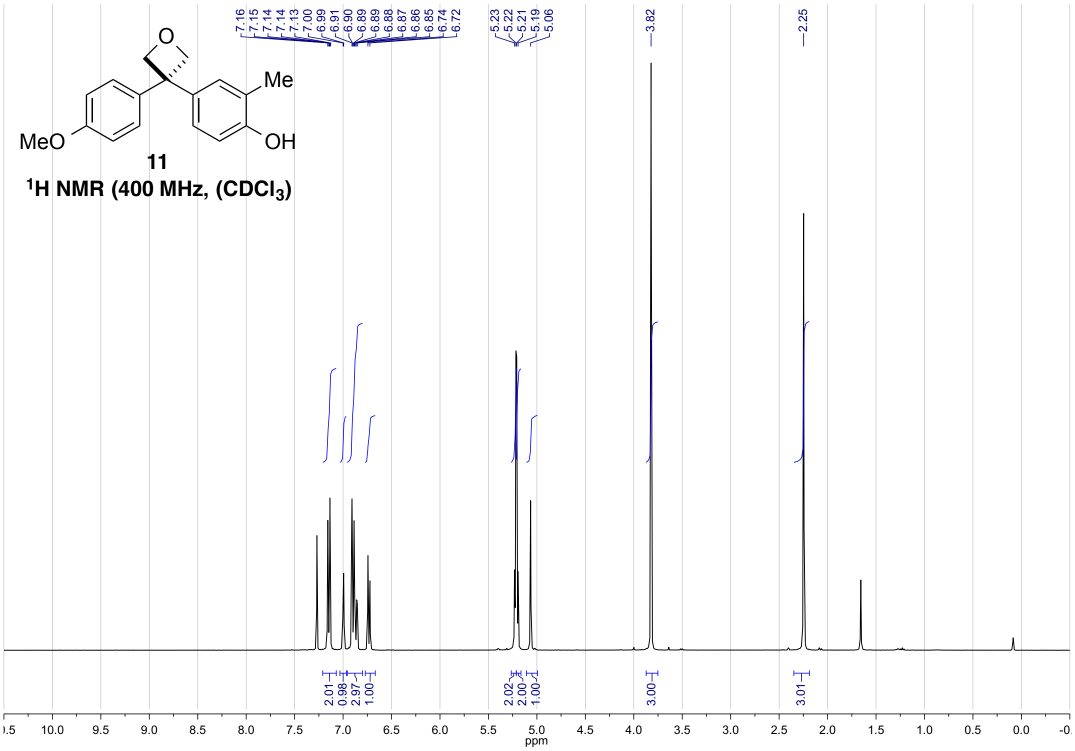

11

^{1}H NMR (400 MHz, (CDCl$_3$)

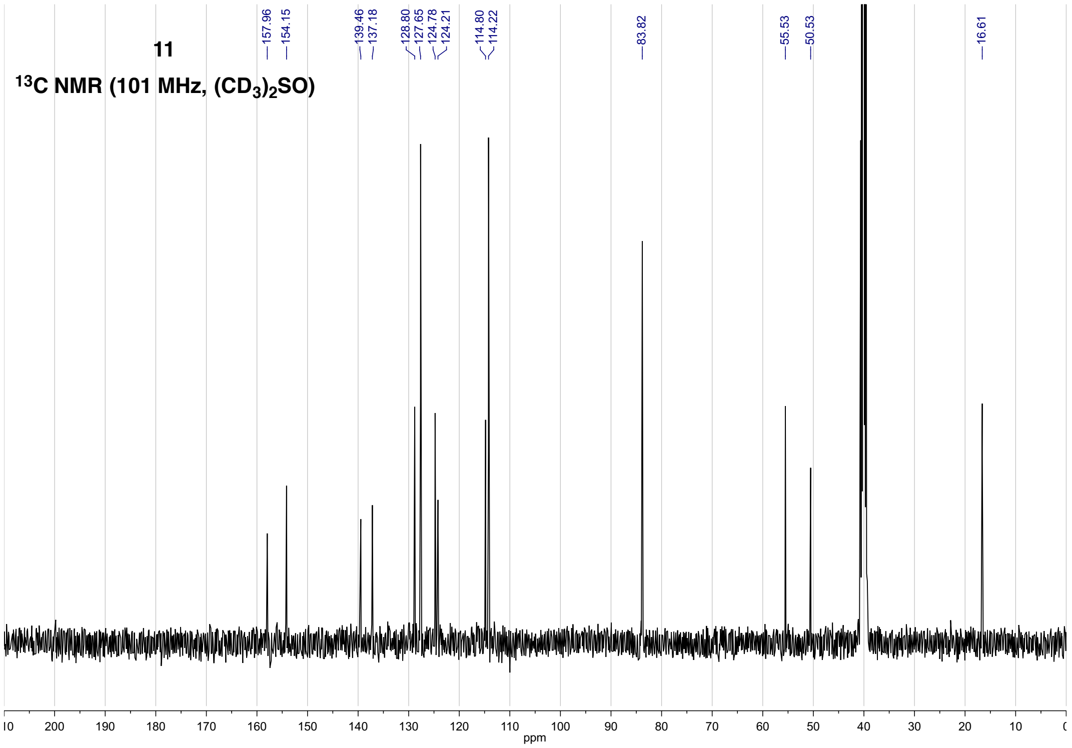

11

^{13}C NMR (101 MHz, (CD$_3$)$_2$SO)

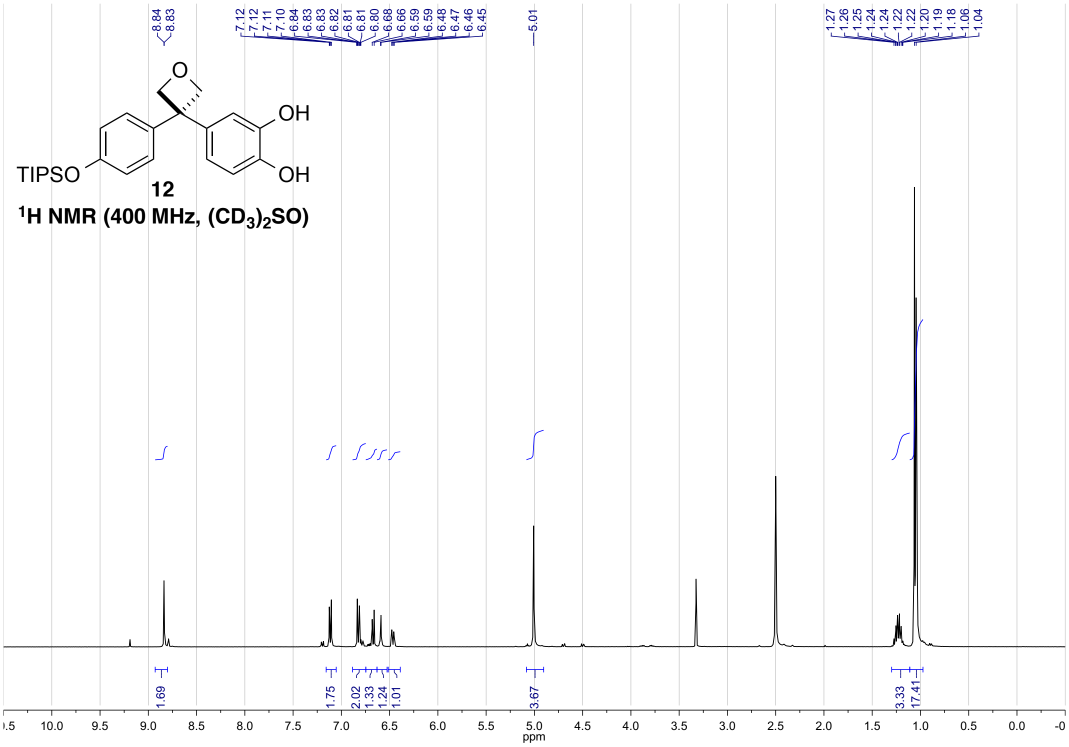
TIPSO
OH
OH
O
12
^{1}H NMR (400 MHz, $(CD_3)_2SO$)
8.84
8.83
7.12
7.12
7.11
7.10
6.84
6.83
6.83
6.82
6.81
6.81
6.80
6.68
6.66
6.59
6.59
6.48
6.47
6.46
6.45
5.01
1.27
1.26
1.25
1.24
1.24
1.22
1.22
1.20
1.19
1.18
1.06
1.04
1.69
1.75
2.02
1.33
1.24
1.01
3.67
3.33
17.41
ppm

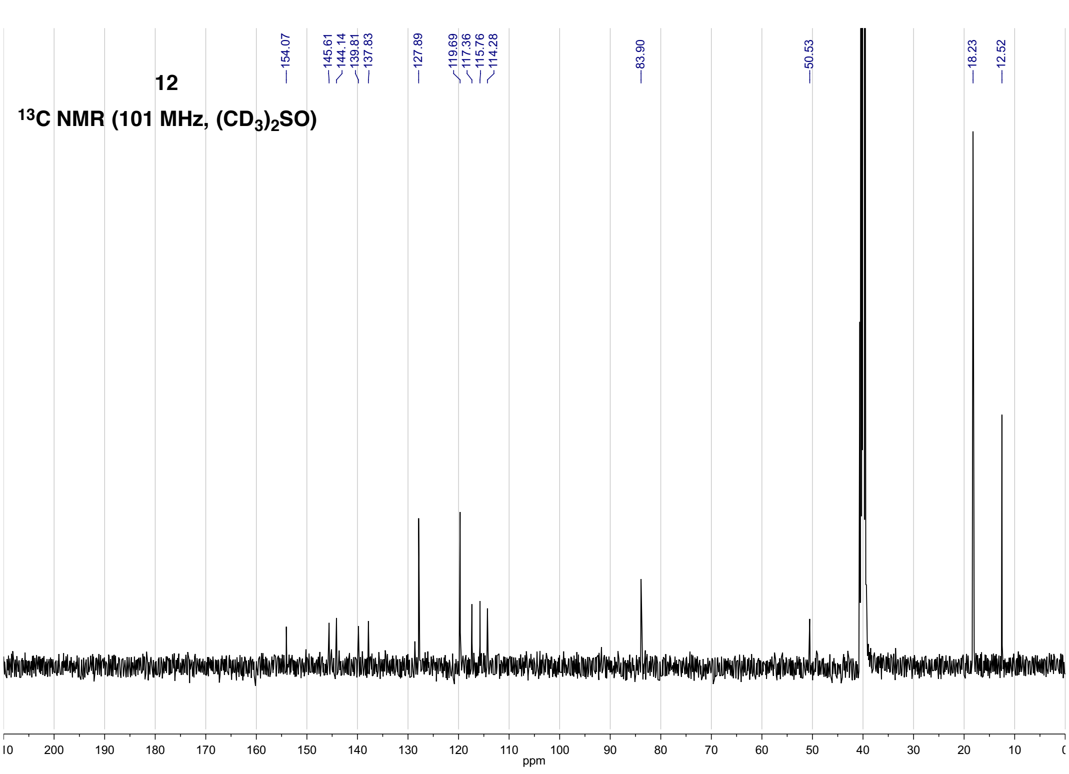
12
^{13}C NMR (101 MHz, $(CD_3)_2SO$)
154.07
145.61
144.14
139.81
137.83
127.89
119.69
117.36
115.76
114.28
83.90
50.53
18.23
12.52
ppm

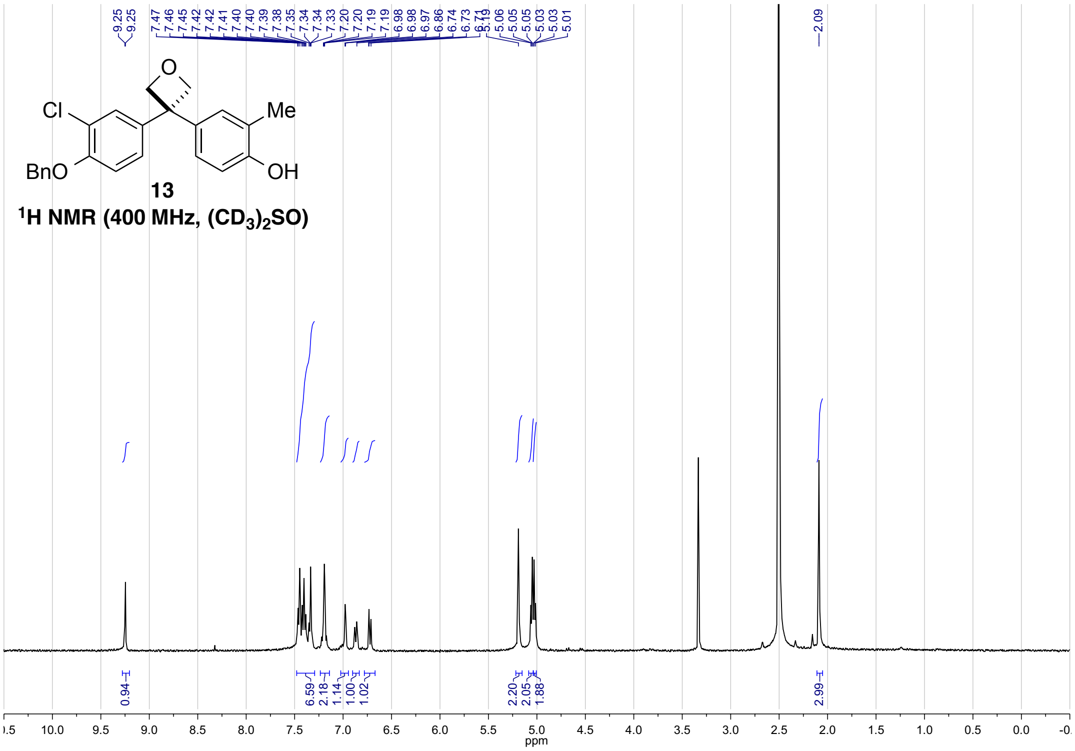

Cl
Me
O
BnO
OH
13
^{1}H NMR (400 MHz, $(CD_3)_2SO$)
ppm

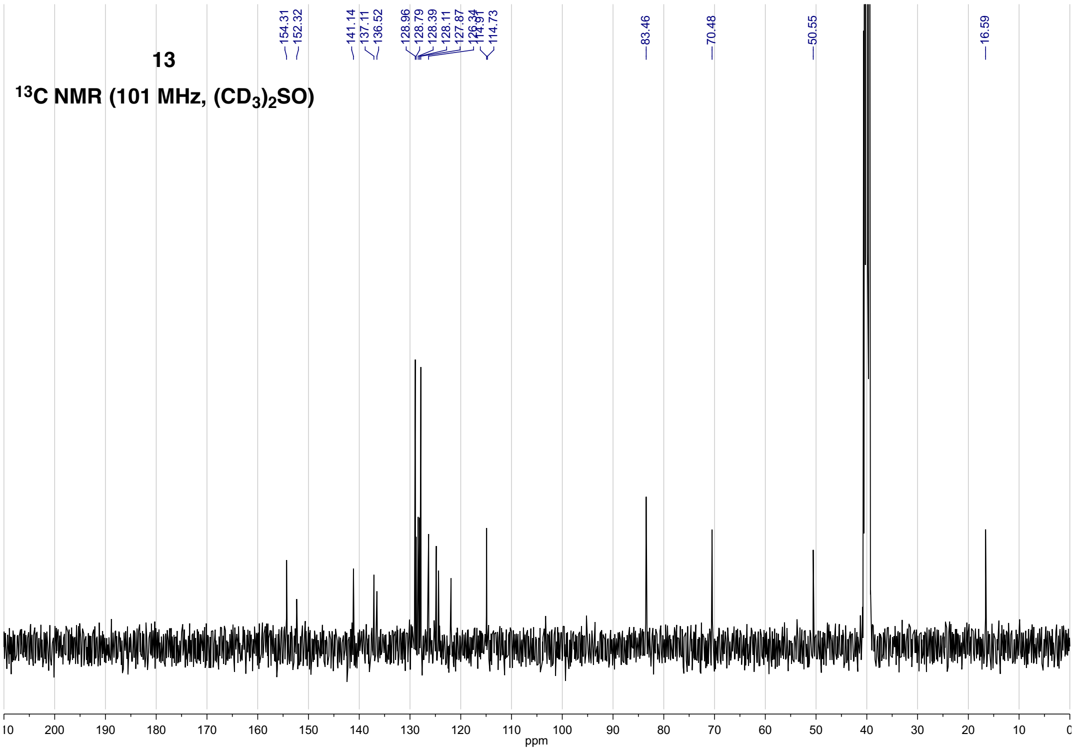

13
^{13}C NMR (101 MHz, $(CD_3)_2SO$)
ppm

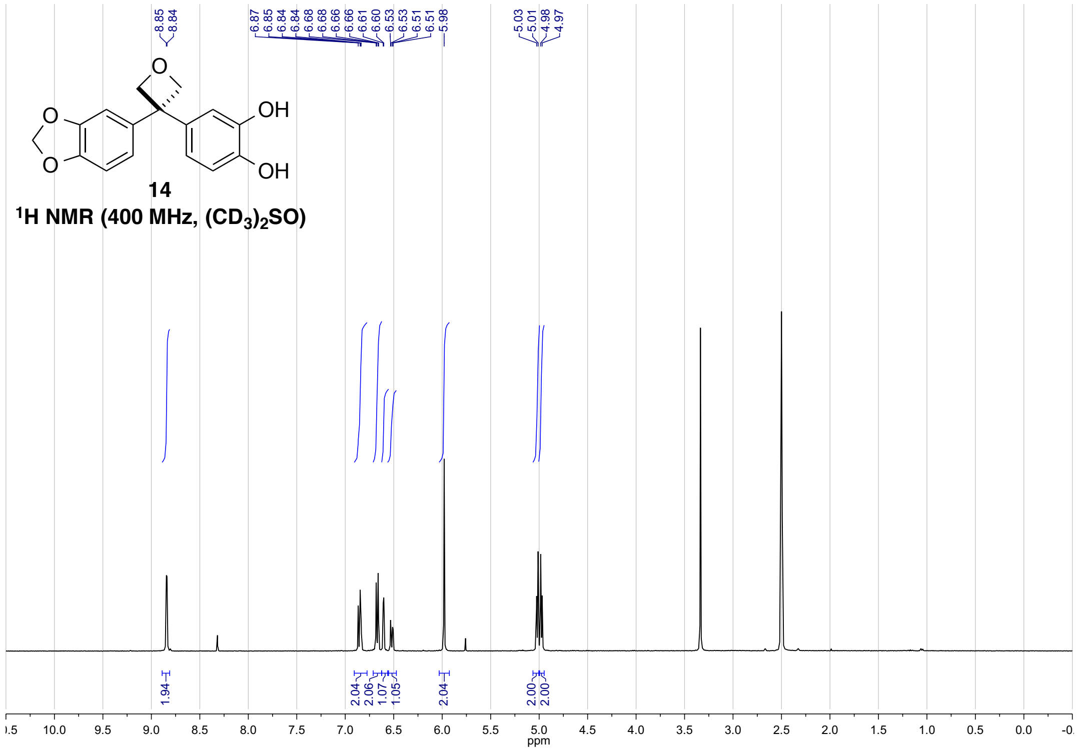
14
^{1}H NMR (400 MHz, $(CD_3)_2SO$)
ppm

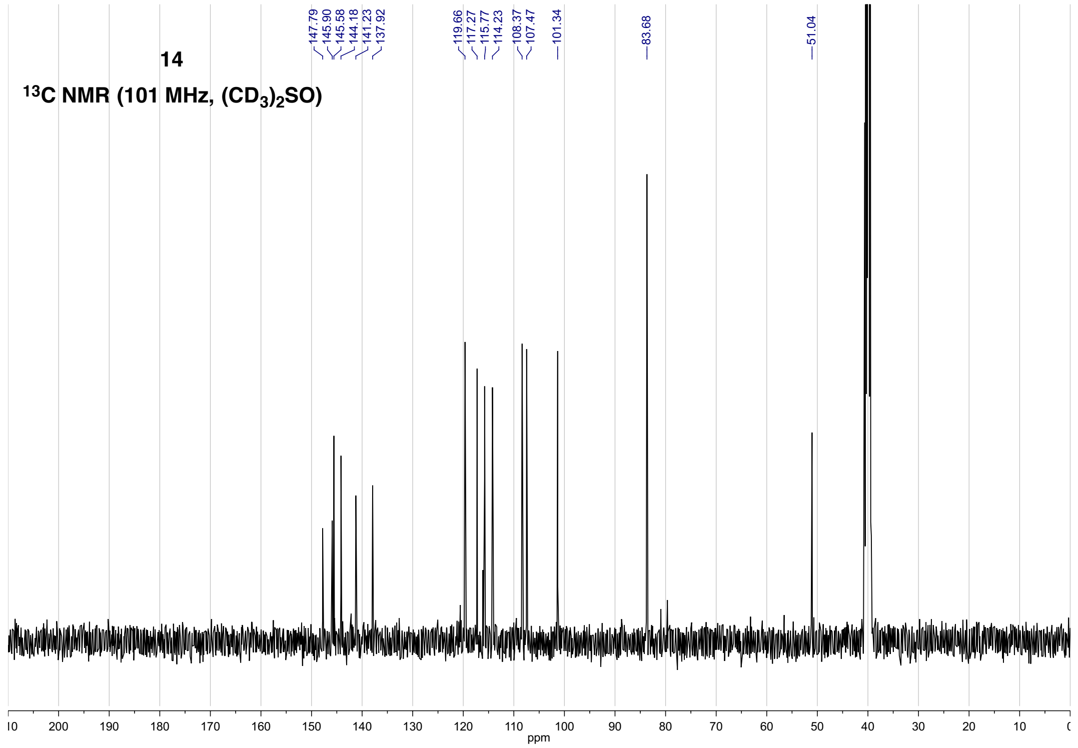
14
^{13}C NMR (101 MHz, $(CD_3)_2SO$)
ppm

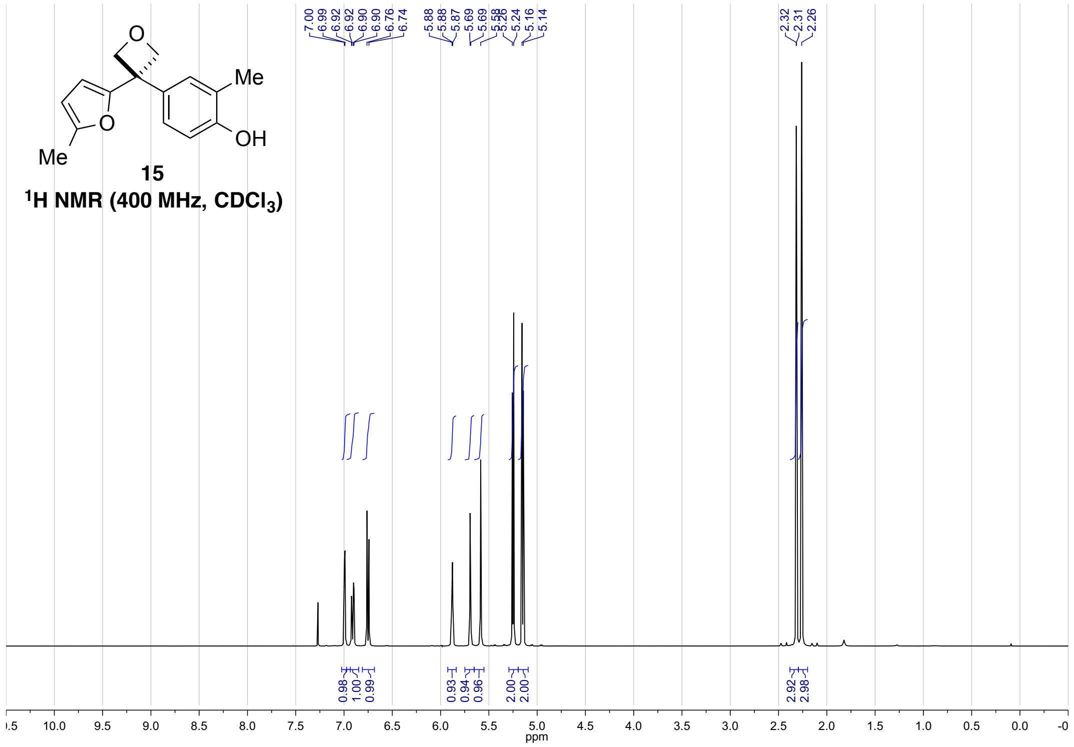
Me
OH
Me
15
1H NMR (400 MHz, CDCl3)
ppm

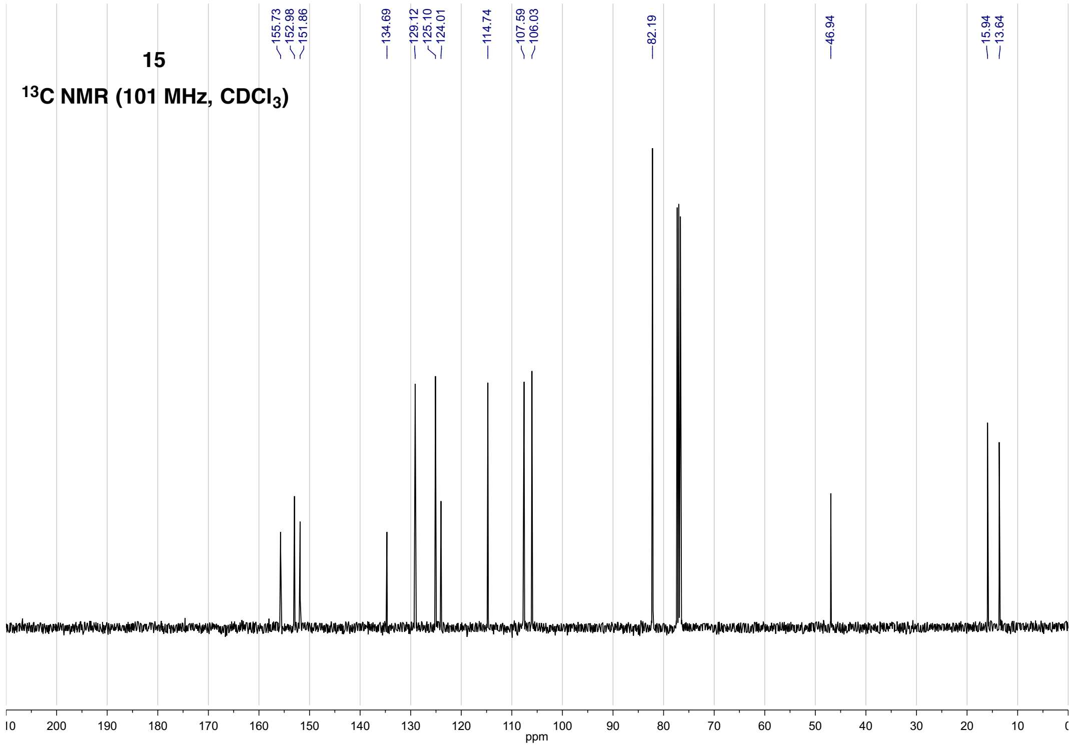
15
13C NMR (101 MHz, CDCl3)
ppm

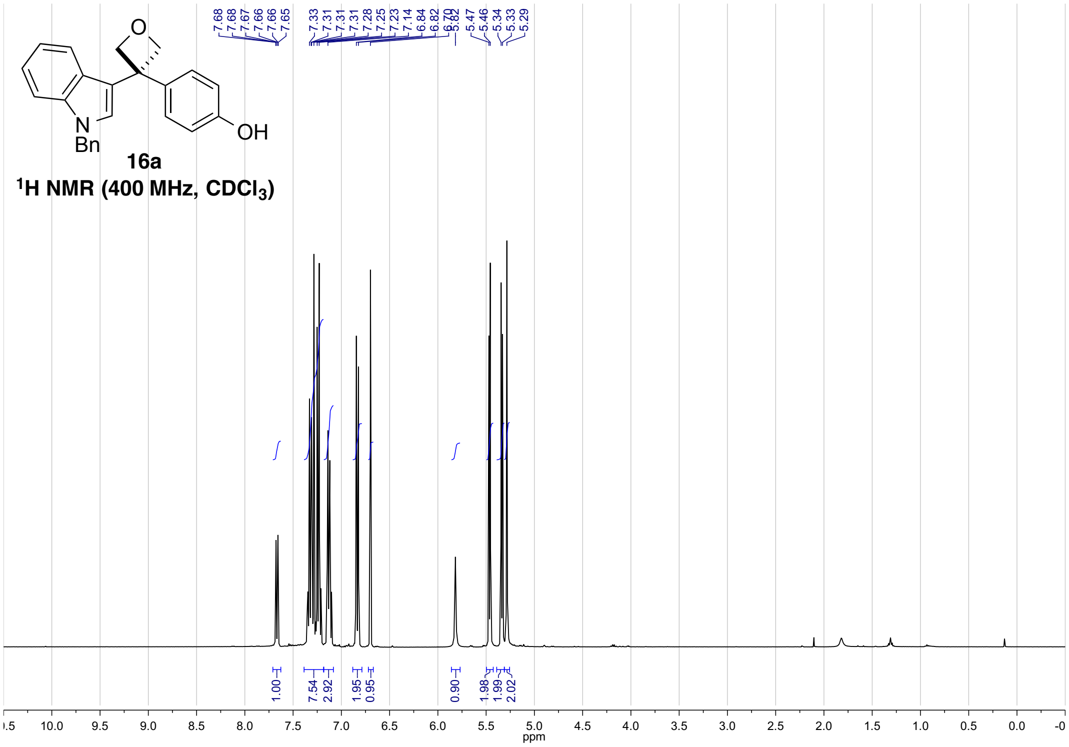
7.68
7.68
7.67
7.66
7.66
7.65
7.33
7.31
7.31
7.31
7.28
7.25
7.23
7.14
6.84
6.82
6.70
5.82
5.47
5.46
5.34
5.33
5.29
O
N
Bn
OH
16a
^{1}H NMR (400 MHz, $CDCl_3$)
1.00
7.54
2.92
1.95
0.95
0.90
1.98
1.99
2.02
ppm

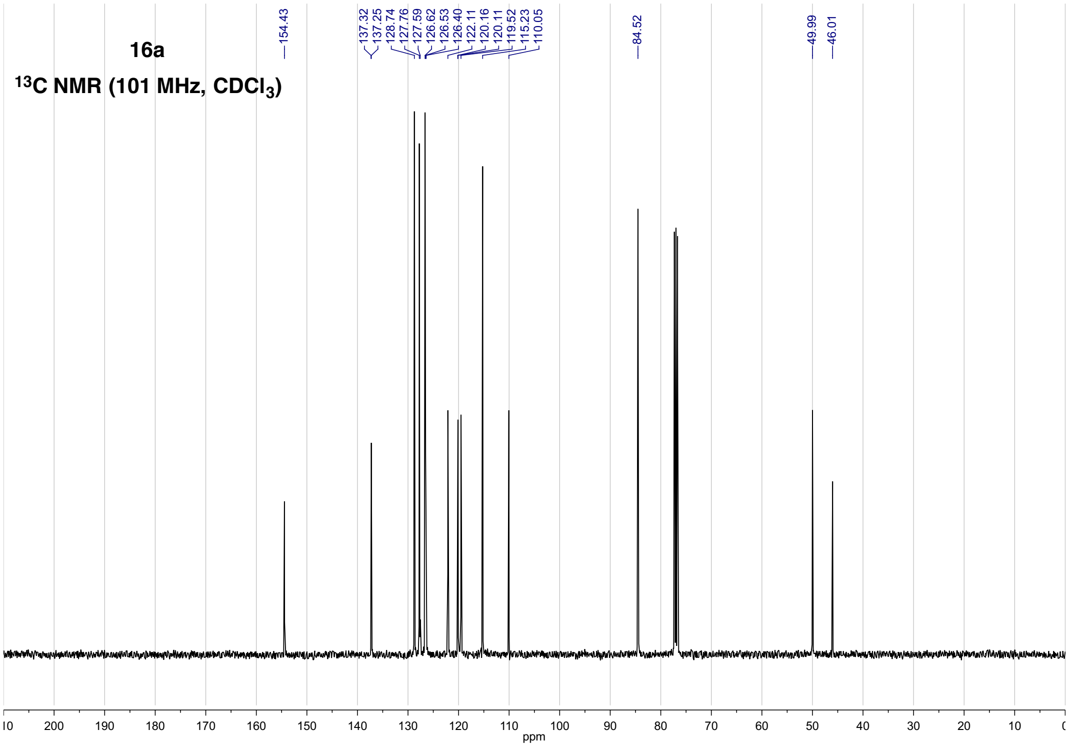
154.43
137.32
137.25
128.74
127.76
127.59
126.62
126.53
126.40
122.11
120.16
120.11
119.52
115.23
110.05
84.52
49.99
46.01
16a
^{13}C NMR (101 MHz, $CDCl_3$)
ppm

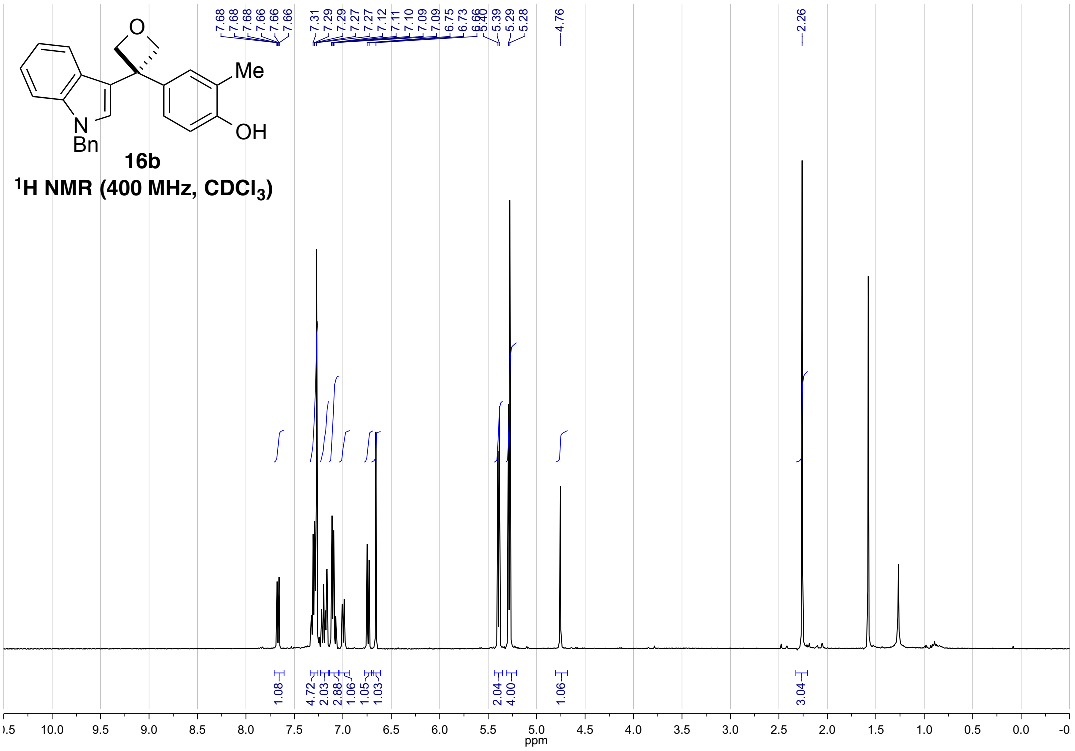
Me
OH
N
Bn
O
16b
¹H NMR (400 MHz, CDCl₃)
ppm

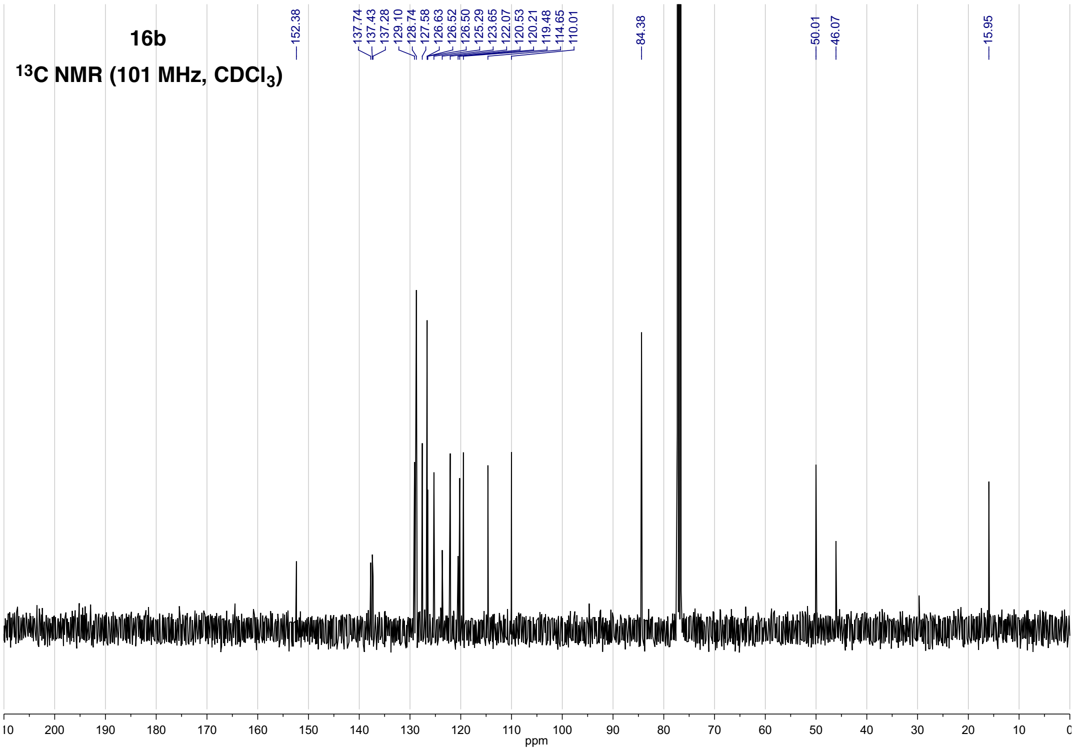
16b
¹³C NMR (101 MHz, CDCl₃)
ppm

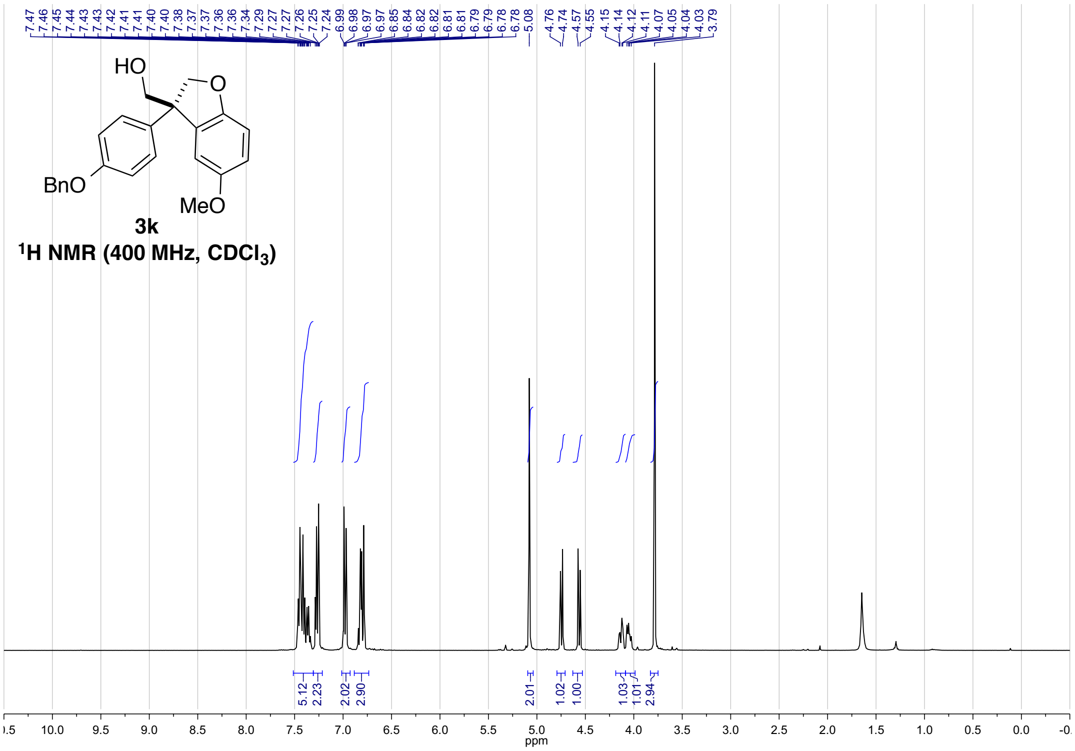
HO
O
BnO
MeO
3k
¹H NMR (400 MHz, CDCl₃)
ppm

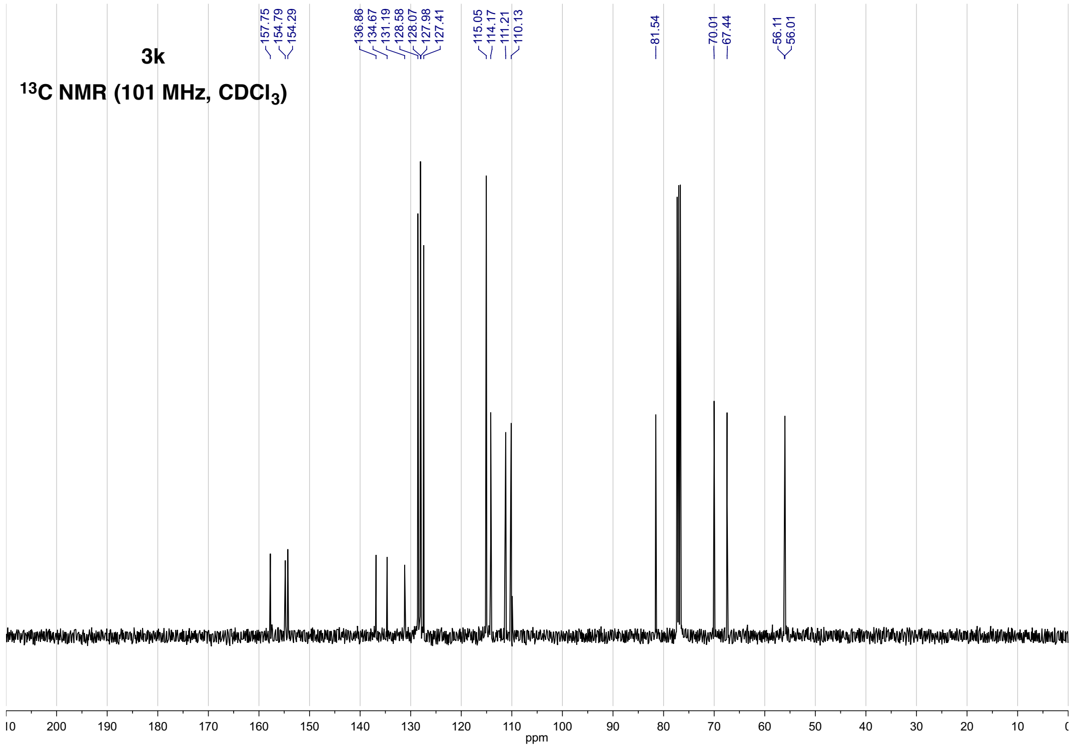
3k
¹³C NMR (101 MHz, CDCl₃)
ppm

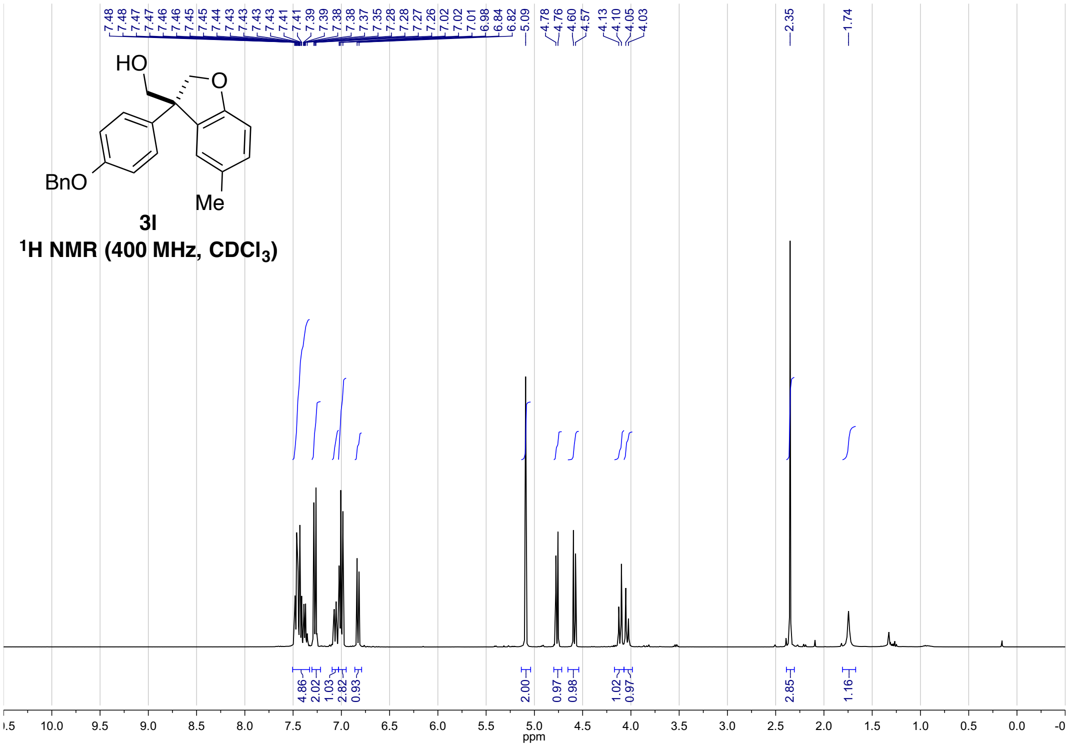
7.48 7.48 7.47 7.46 7.46 7.45 7.45 7.44 7.43 7.43 7.43 7.43 7.41 7.39 7.39 7.38 7.38 7.37 7.35 7.28 7.28 7.27 7.26 7.02 7.02 7.01 6.98 6.84 6.82 5.09 4.78 4.76 4.60 4.57 4.13 4.10 4.05 4.03 2.35 1.74
HO
O
BnO
Me
3l
1H NMR (400 MHz, CDCl3)
4.86 2.02 1.03 2.82 0.93 2.00 0.97 0.98 1.02 0.97 2.85 1.16
ppm

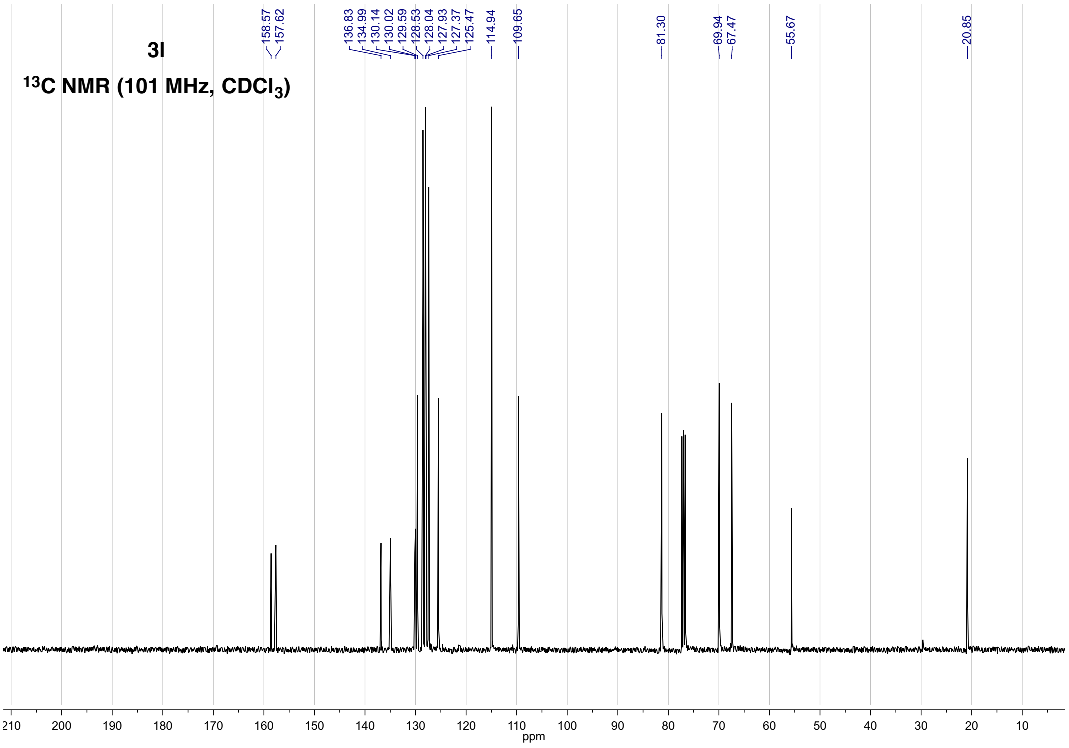
158.57 157.62 136.83 134.99 130.14 130.02 129.59 128.53 128.04 127.93 127.37 125.47 114.94 109.65 81.30 69.94 67.47 55.67 20.85
3l
13C NMR (101 MHz, CDCl3)
ppm

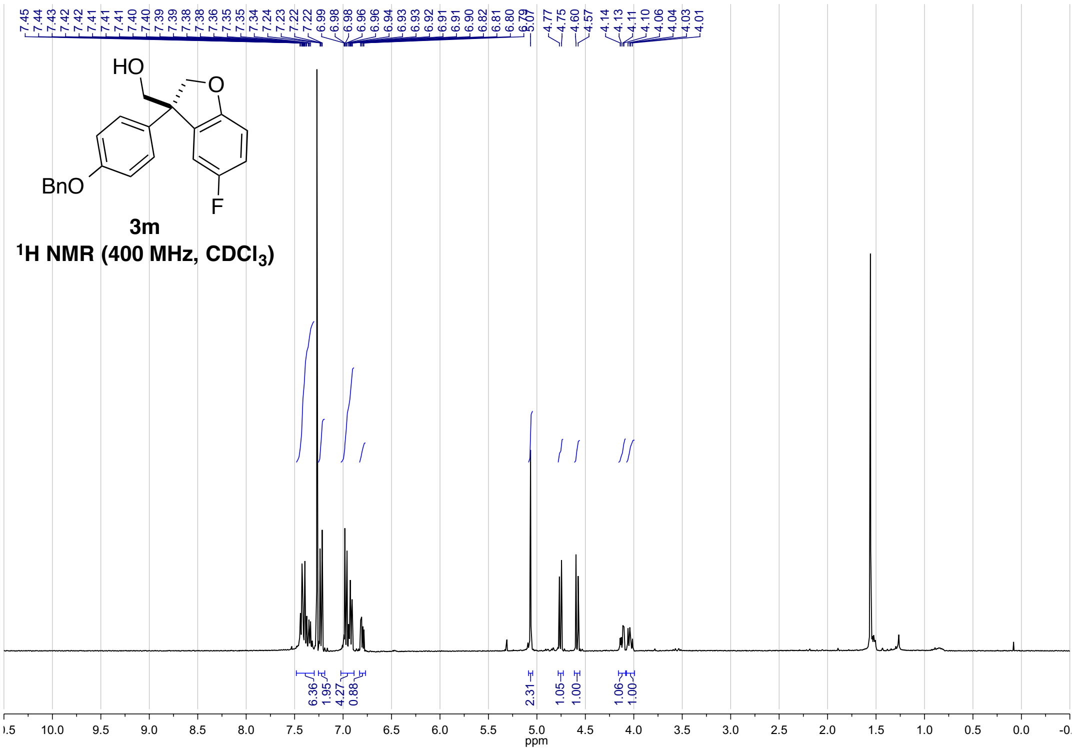
HO
O
BnO
F
3m
1H NMR (400 MHz, CDCl3)
ppm

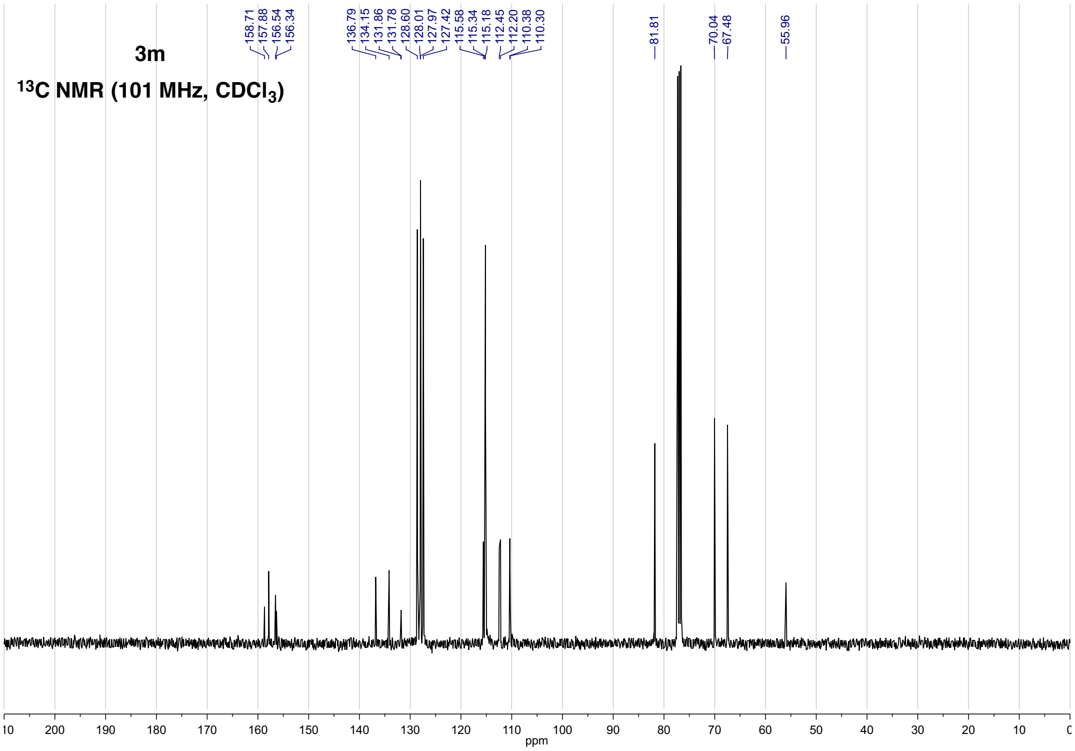
3m
13C NMR (101 MHz, CDCl3)
158.71
157.88
156.54
156.34
136.79
134.15
131.86
131.78
128.60
128.01
127.97
127.42
115.58
115.34
115.18
112.45
112.20
110.38
110.30
81.81
70.04
67.48
55.96
ppm

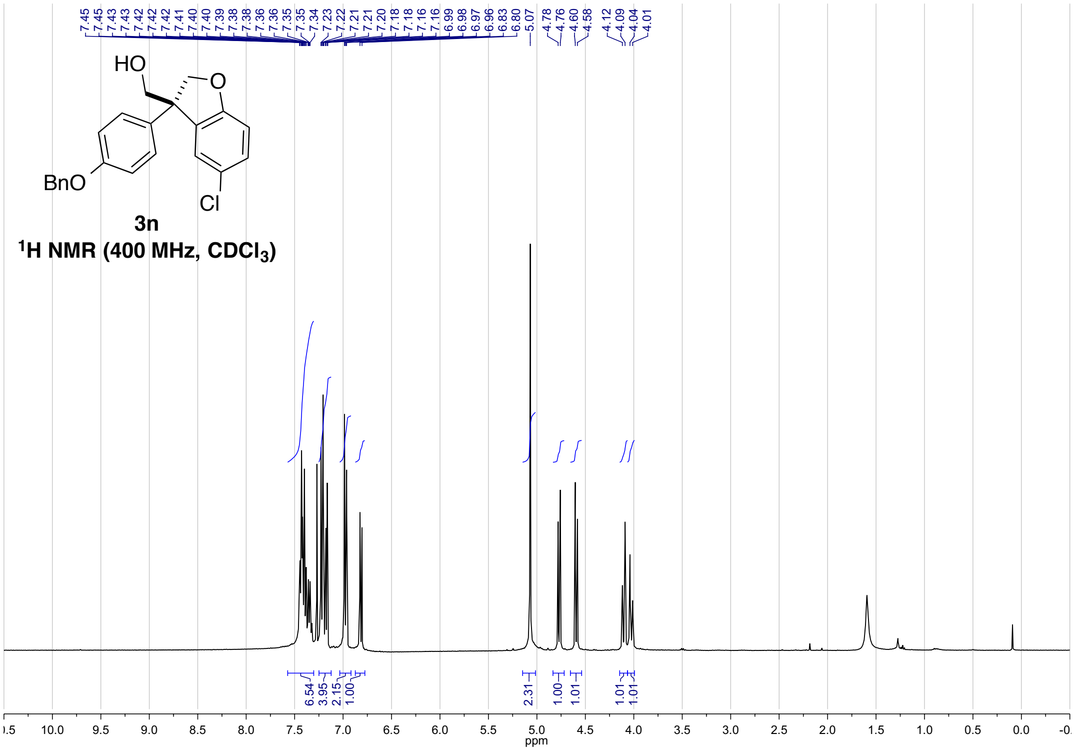

3n

^{1}H NMR (400 MHz, $CDCl_3$)

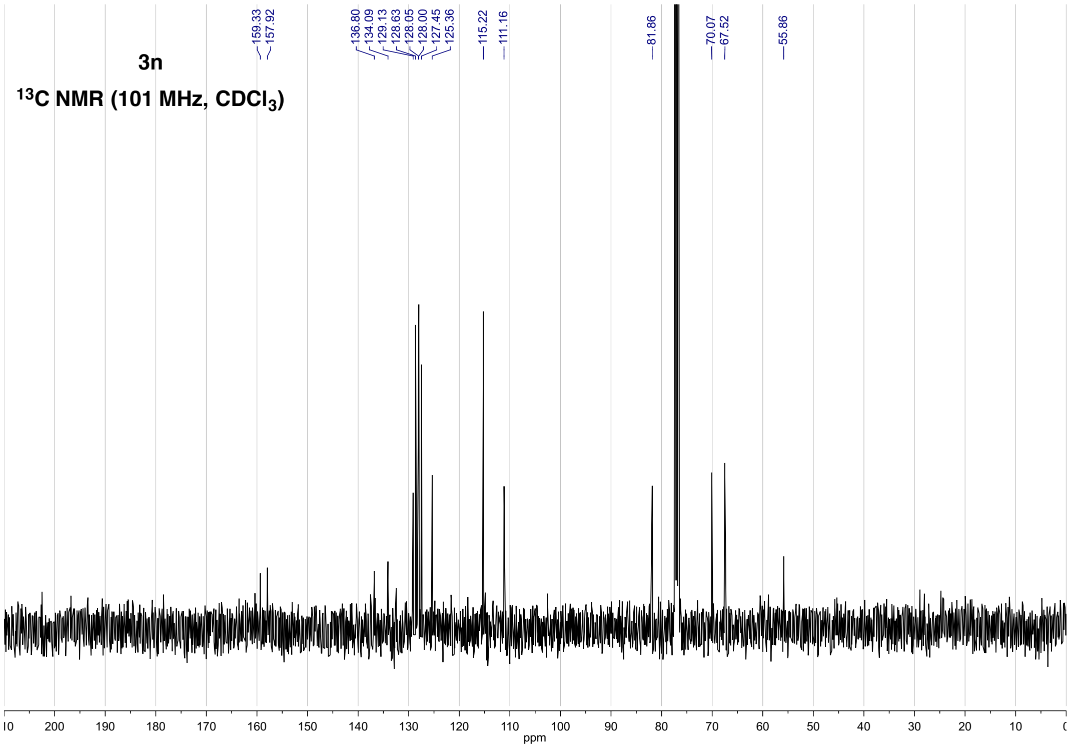

3n

^{13}C NMR (101 MHz, $CDCl_3$)

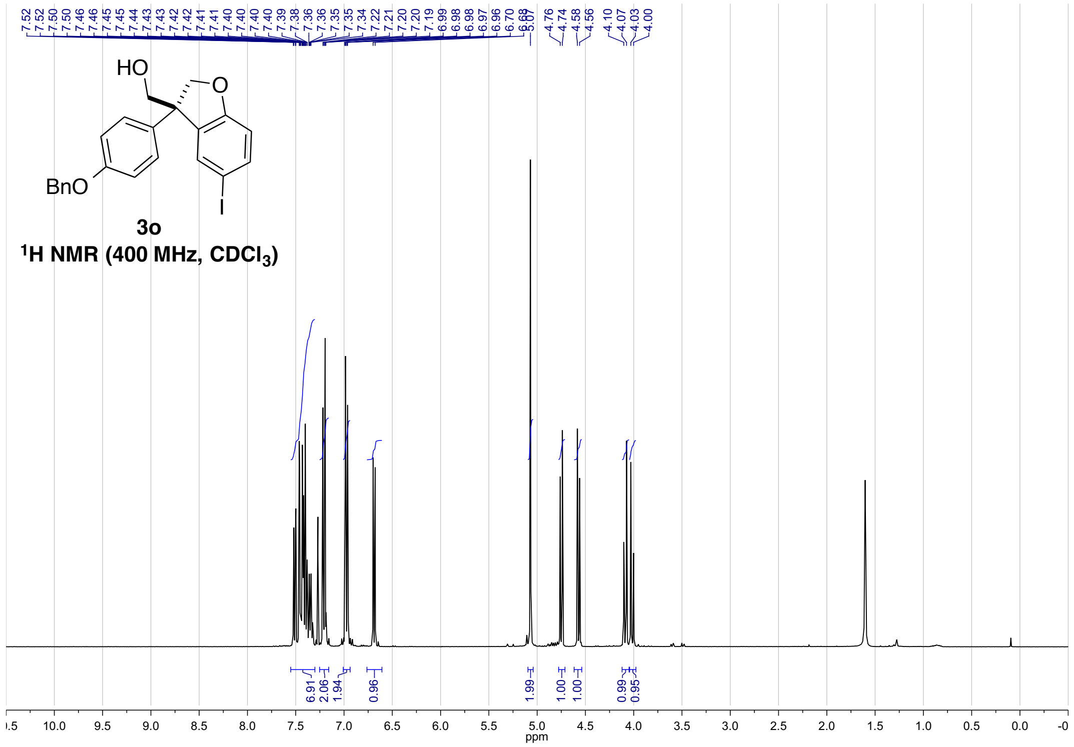
HO
O
BnO
I
3o
1H NMR (400 MHz, CDCl3)
ppm

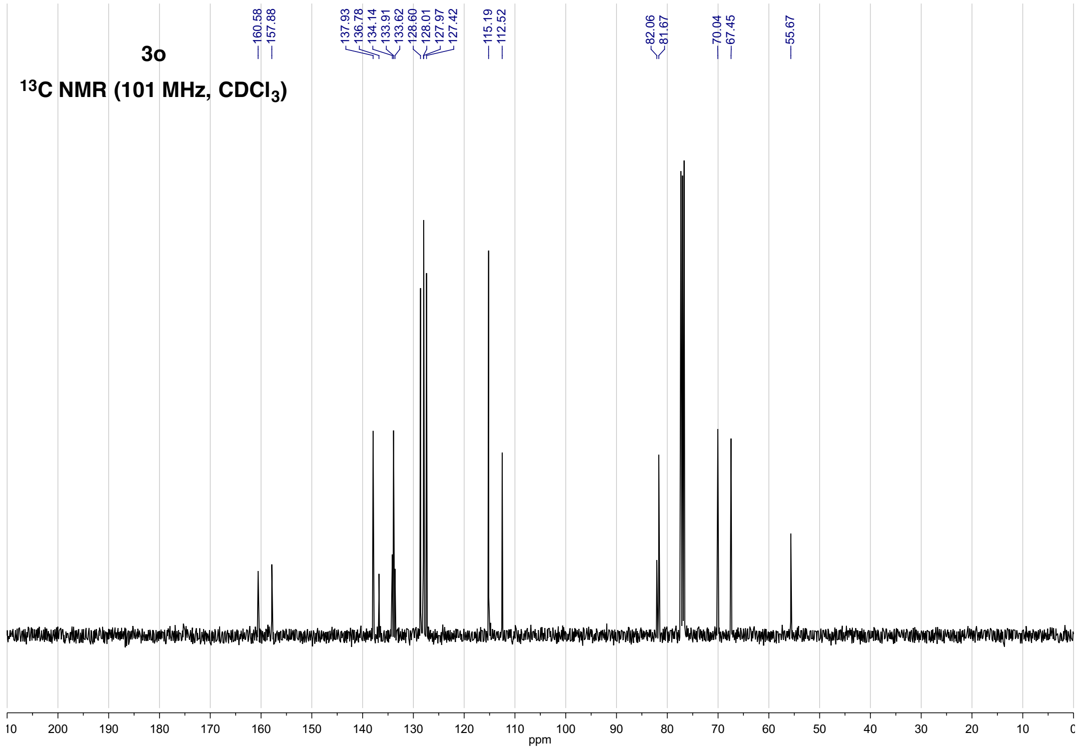
3o
13C NMR (101 MHz, CDCl3)
ppm

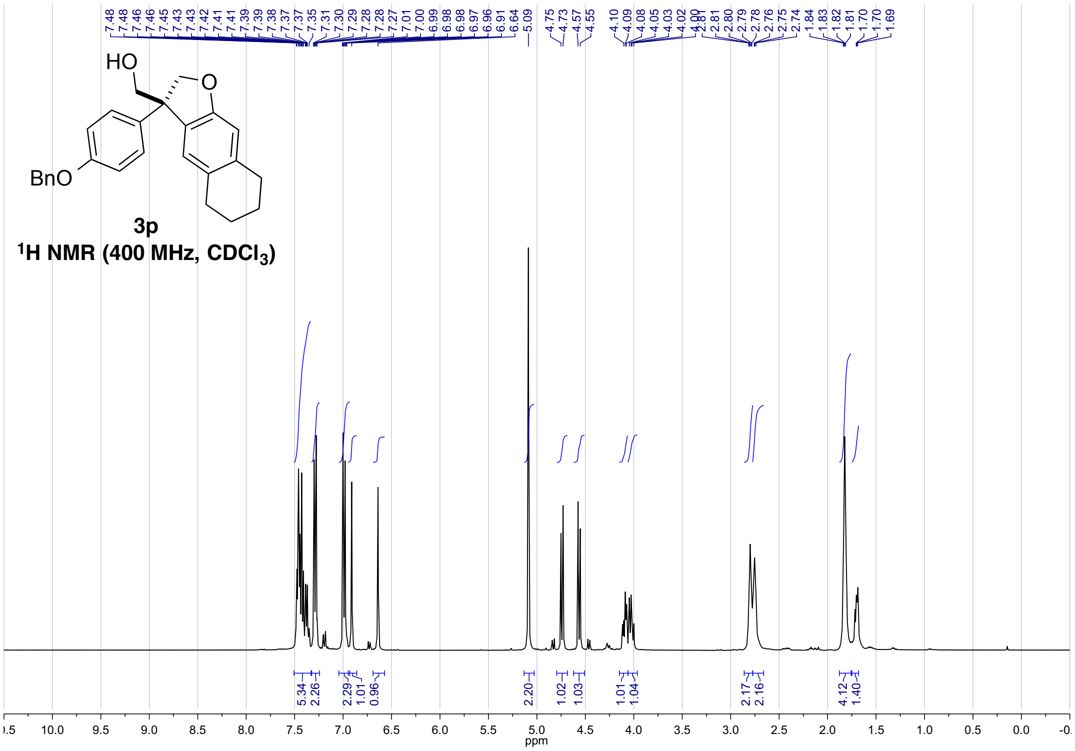
HO
O
BnO
3p
1H NMR (400 MHz, CDCl3)
ppm

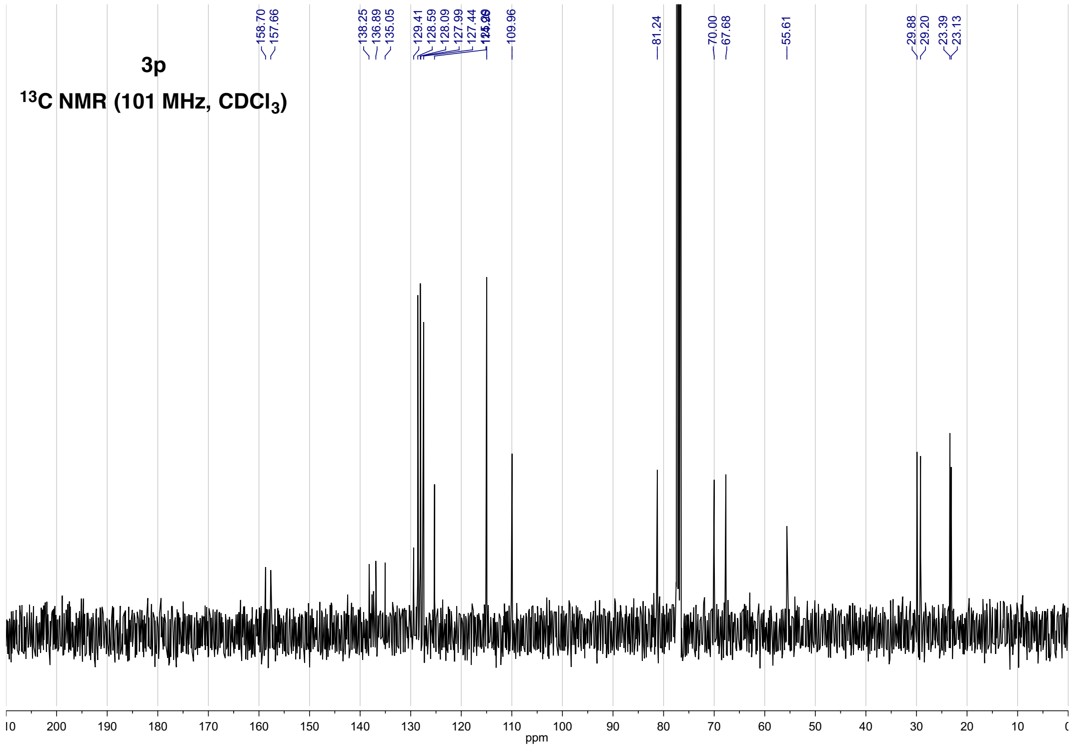
3p
13C NMR (101 MHz, CDCl3)
ppm

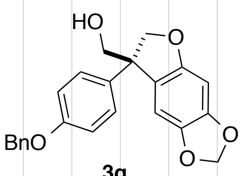

3q

^{1}H NMR (400 MHz, $CDCl_3$)

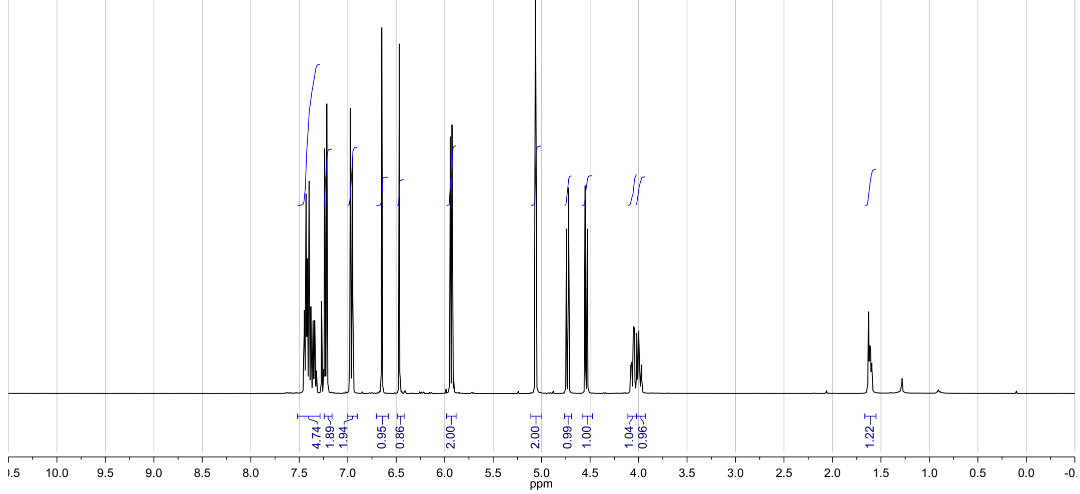

3q

^{13}C NMR (101 MHz, $CDCl_3$)

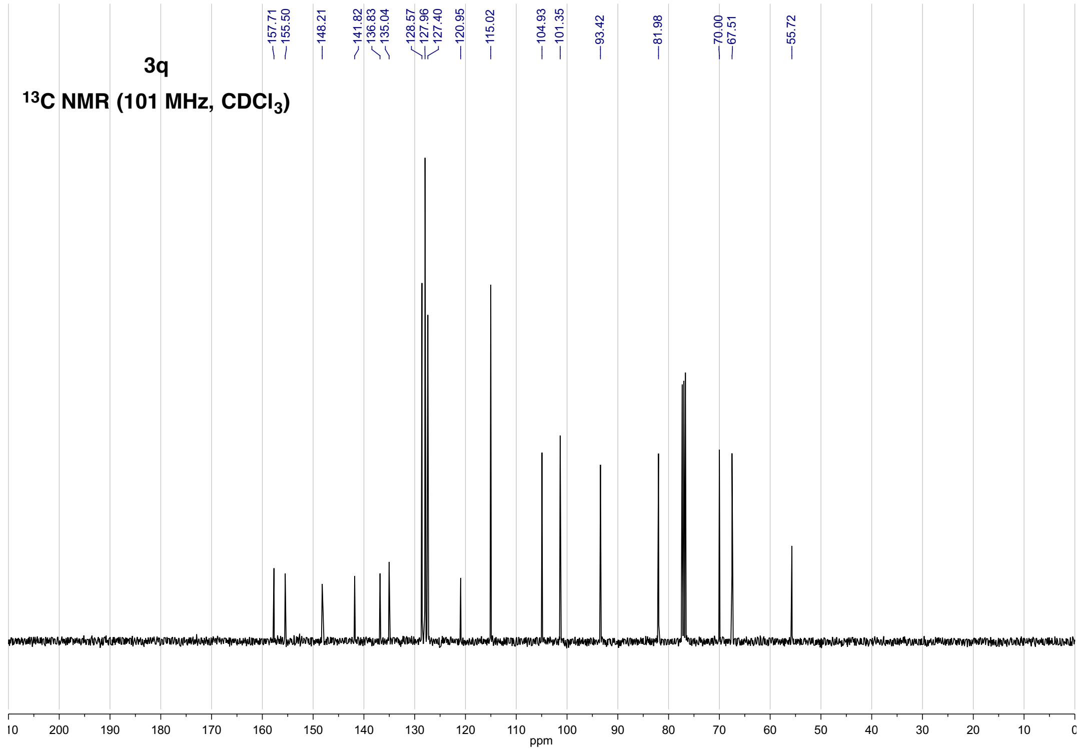

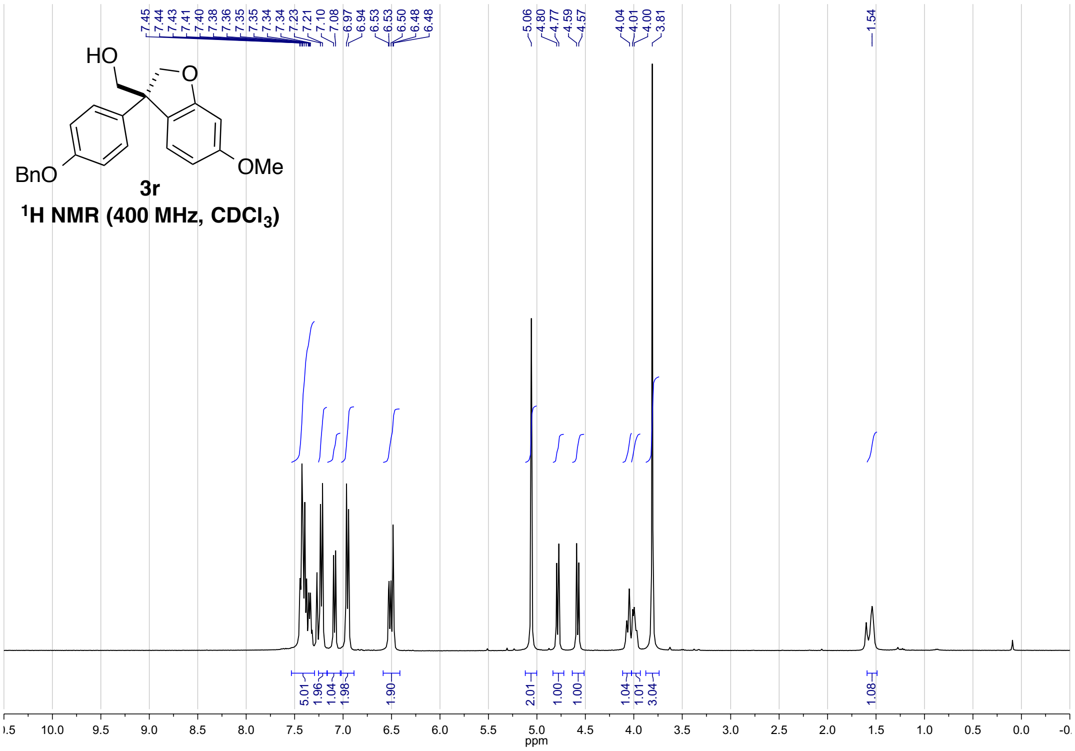
HO
O
BnO
OMe
3r
1H NMR (400 MHz, CDCl3)
7.45 7.44 7.43 7.41 7.40 7.38 7.36 7.35 7.35 7.34 7.34 7.23 7.21 7.10 7.08 6.97 6.94 6.53 6.53 6.50 6.48 6.48
5.06 4.80 4.77 4.59 4.57 4.04 4.01 4.00 3.81
1.54
5.01 1.96 1.04 1.98 1.90 2.01 1.00 1.00 1.04 1.01 3.04 1.08
ppm

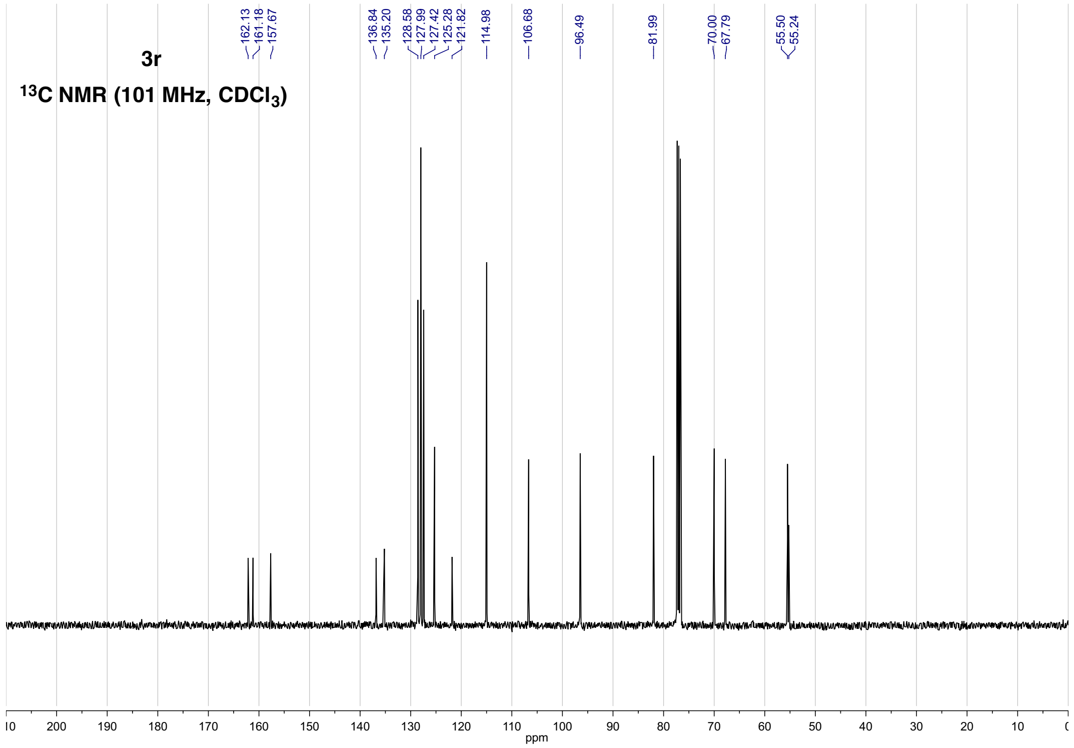
3r
13C NMR (101 MHz, CDCl3)
162.13 161.18 157.67 136.84 135.20 128.58 127.99 127.42 125.28 121.82 114.98 106.68 96.49 81.99 70.00 67.79 55.50 55.24
ppm

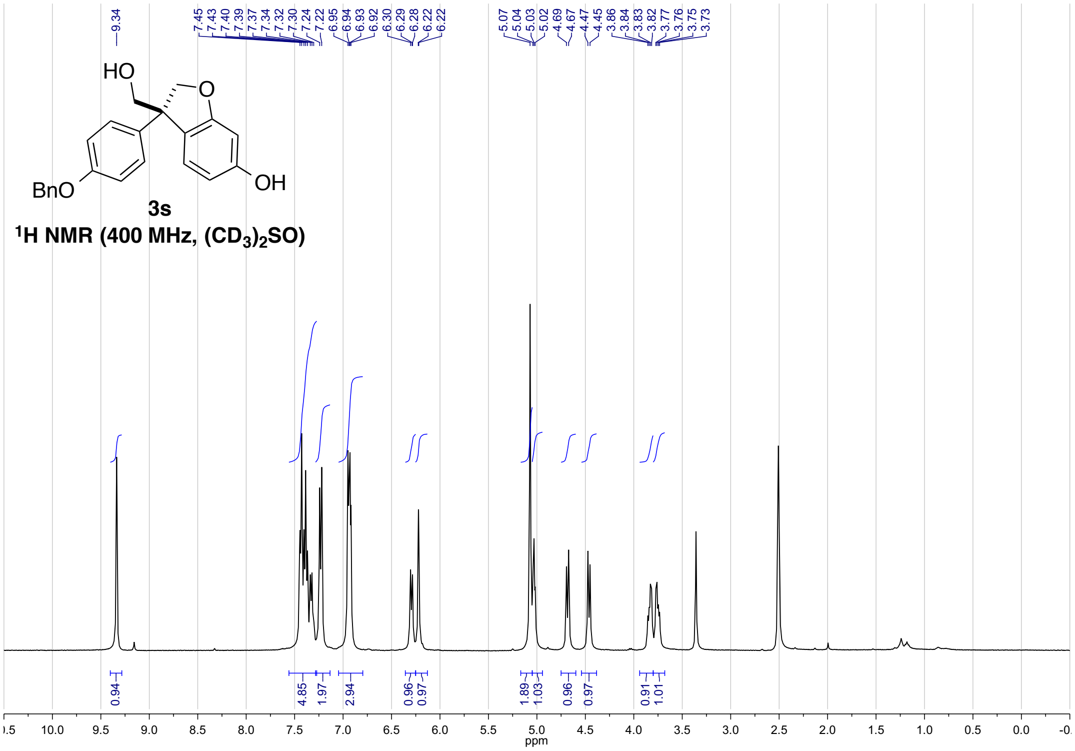
HO
O
BnO
OH
3s
^{1}H NMR (400 MHz, $(CD_3)_2SO$)
ppm

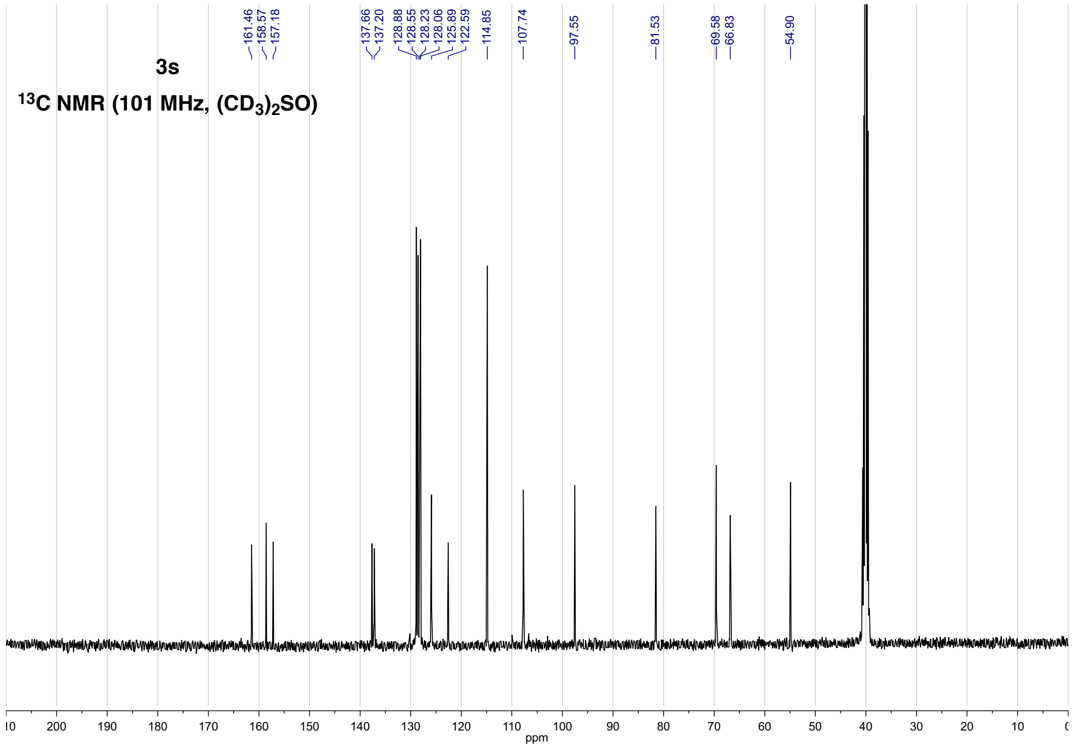
3s
^{13}C NMR (101 MHz, $(CD_3)_2SO$)
ppm

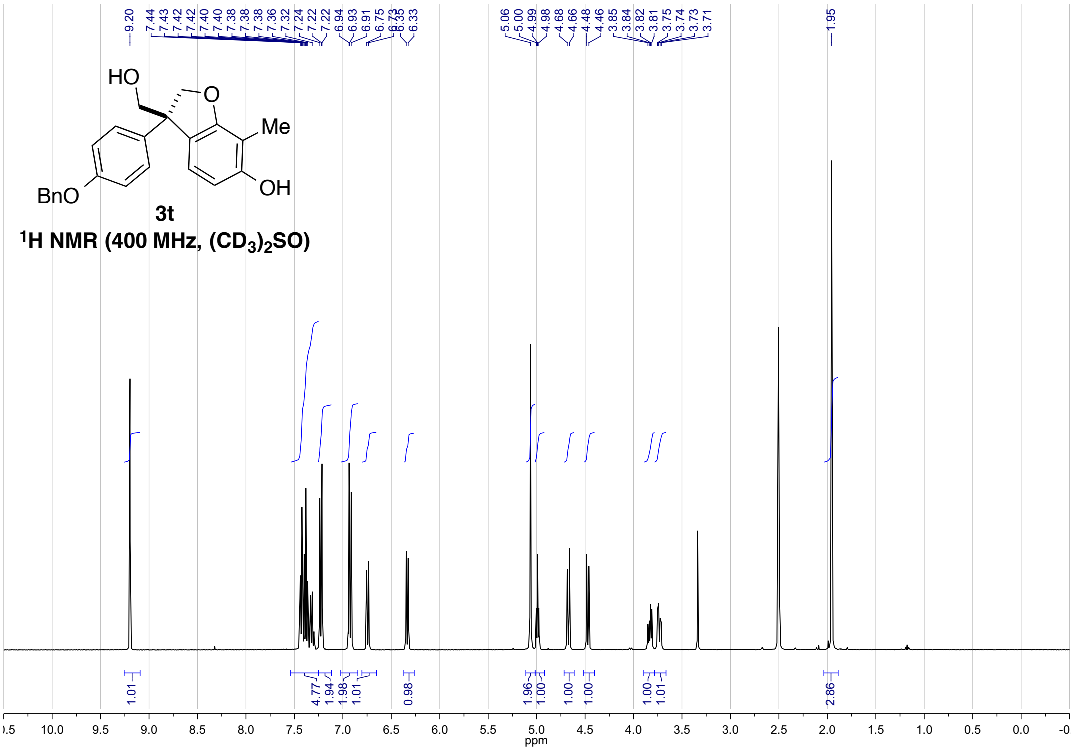
HO
O
Me
OH
BnO
3t
^{1}H NMR (400 MHz, $(CD_3)_2SO$)
ppm

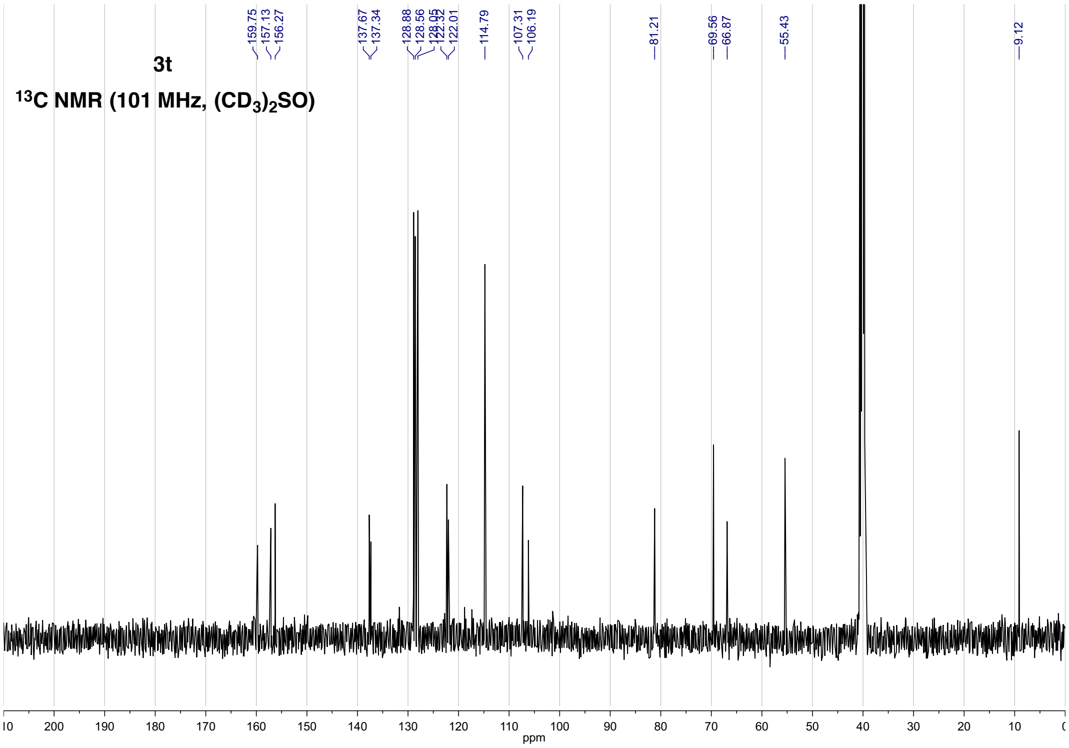
3t
^{13}C NMR (101 MHz, $(CD_3)_2SO$)
ppm

3u

^{1}H NMR (400 MHz, $(CD_3)_2SO$)

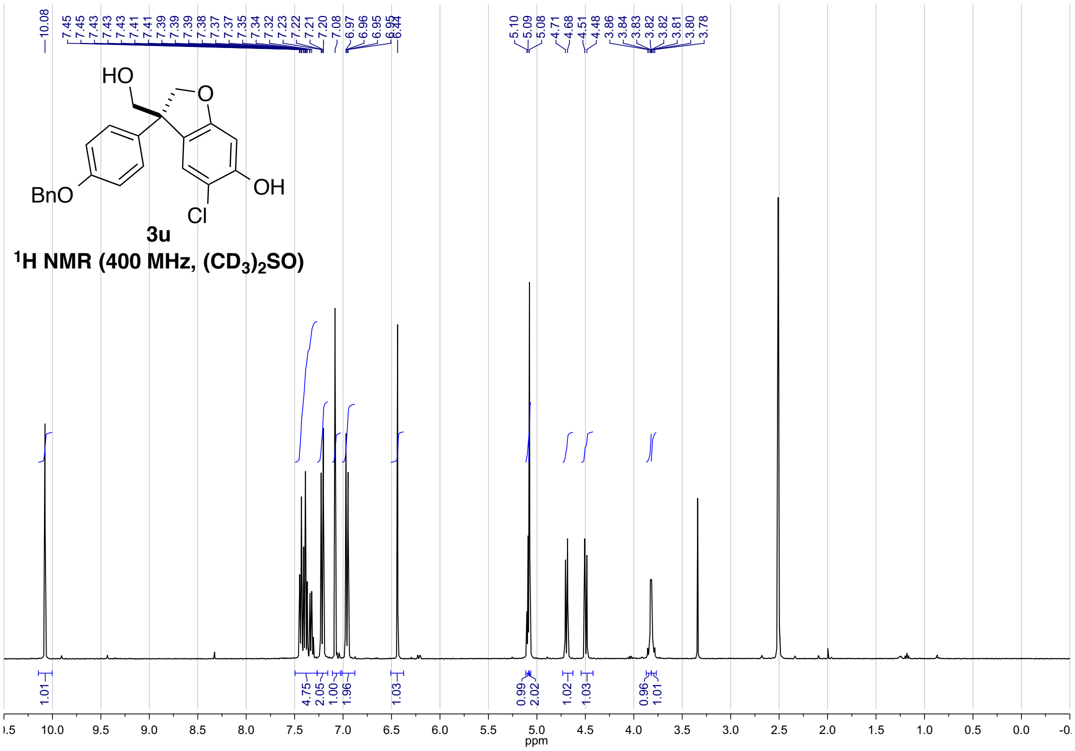

3u

^{13}C NMR (101 MHz, $(CD_3)_2SO$)

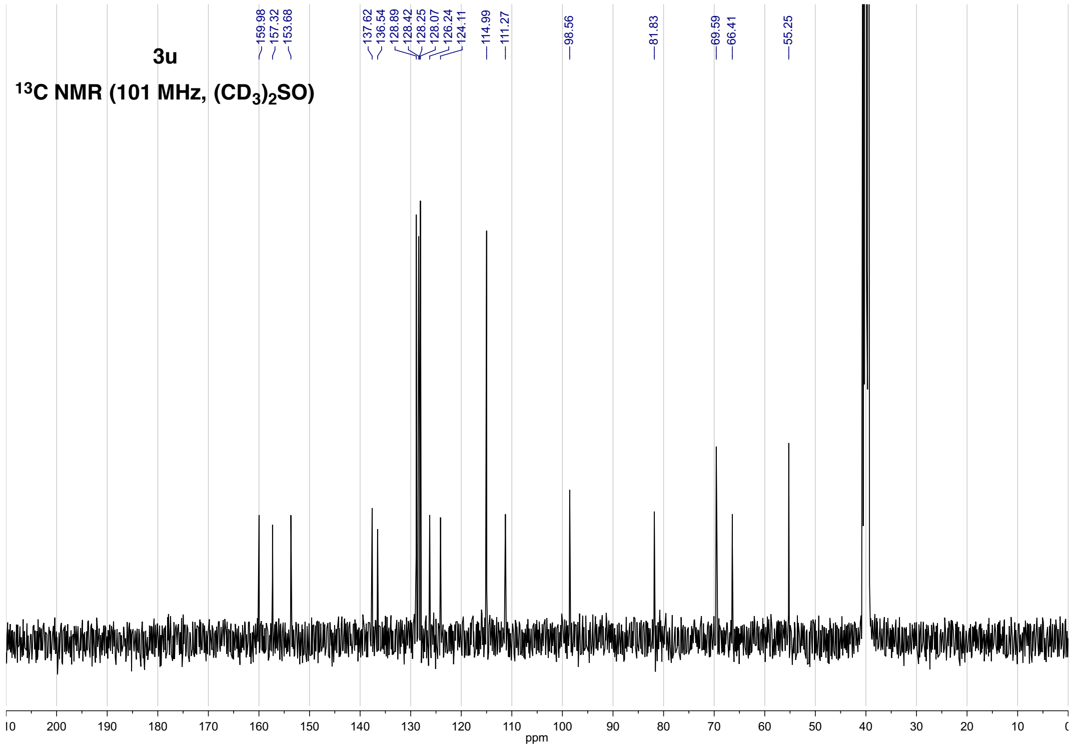

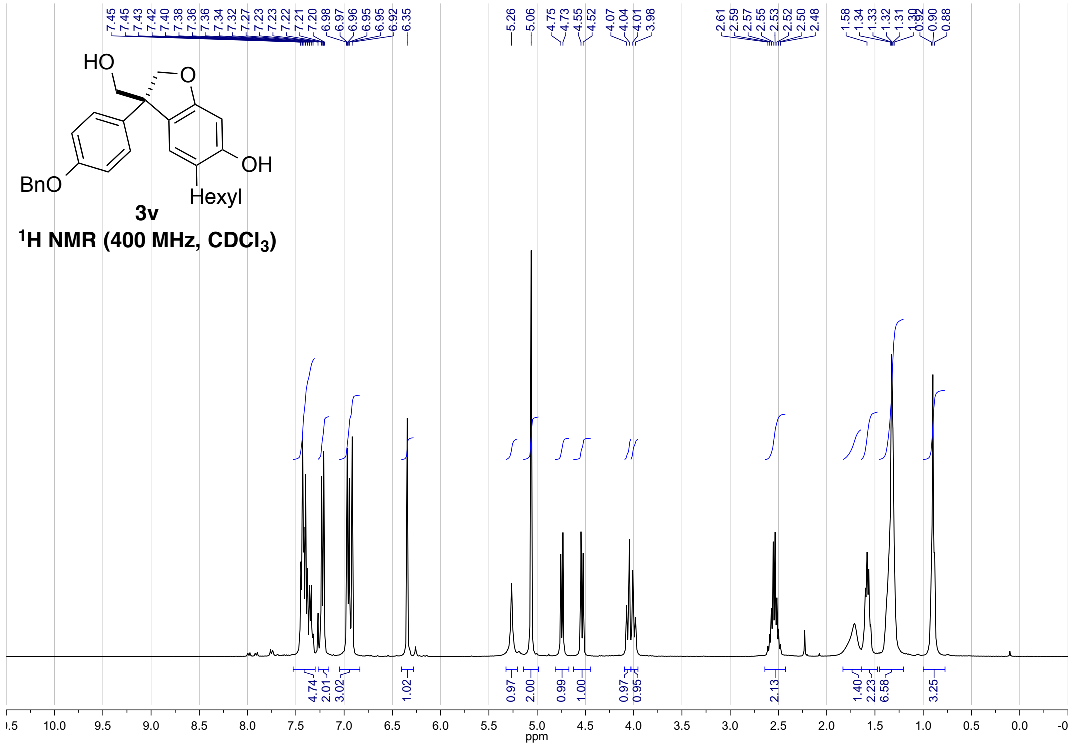
HO
O
OH
BnO
Hexyl
3v
^{1}H NMR (400 MHz, CDCl$_3$)
ppm

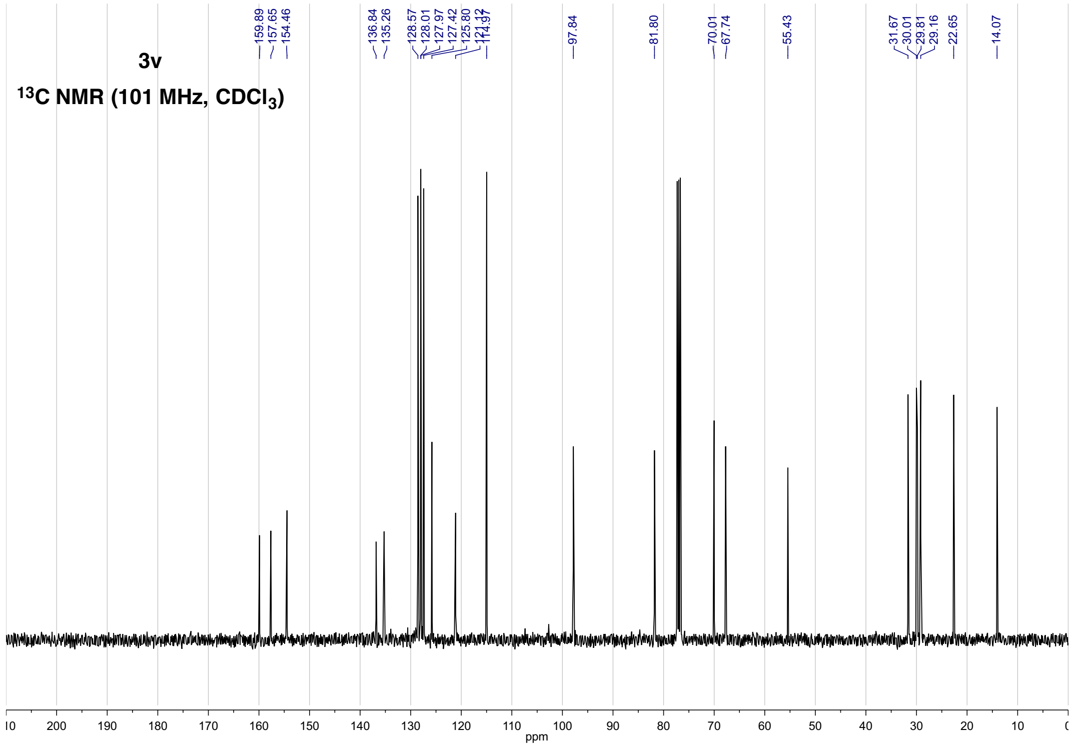
3v
^{13}C NMR (101 MHz, CDCl$_3$)
ppm

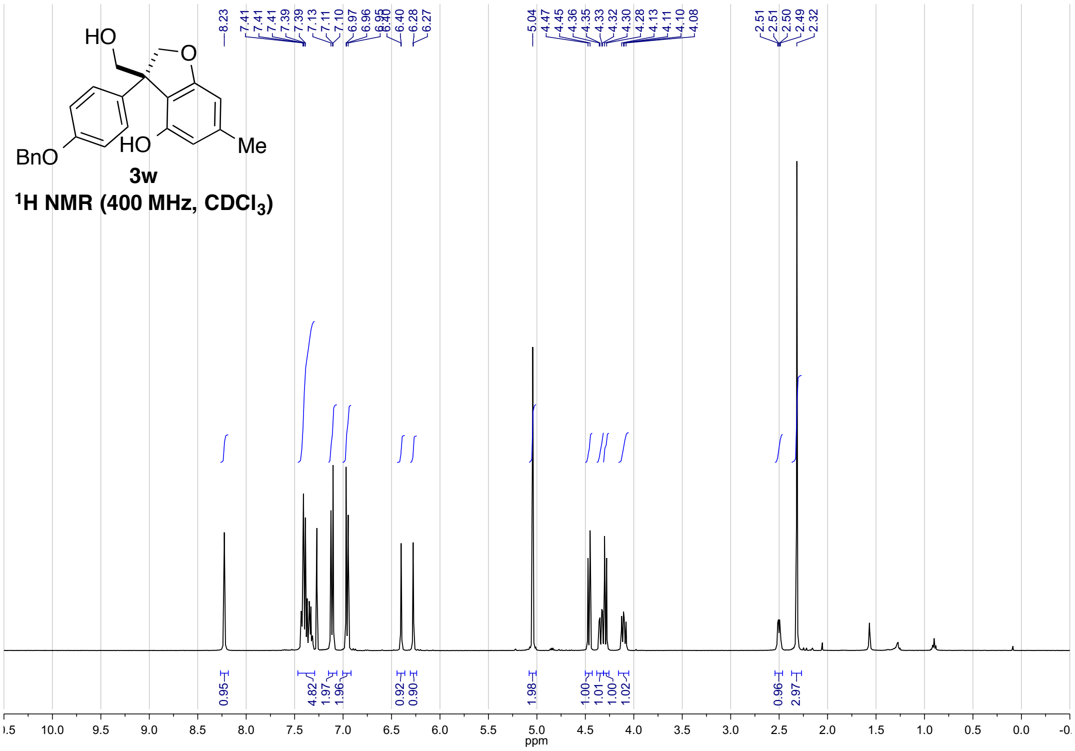
HO
O
BnO
HO
Me
3w
^{1}H NMR (400 MHz, $CDCl_3$)
8.23
7.41 7.41 7.41 7.39 7.39 7.13 7.11 7.10 6.97 6.96 6.95 6.40 6.40 6.28 6.27
5.04 4.47 4.45 4.36 4.35 4.33 4.32 4.30 4.28 4.13 4.11 4.10 4.08
2.51 2.51 2.50 2.49 2.32
0.95 4.82 1.97 1.96 0.92 0.90 1.98 1.00 1.01 1.00 1.02 0.96 2.97
ppm

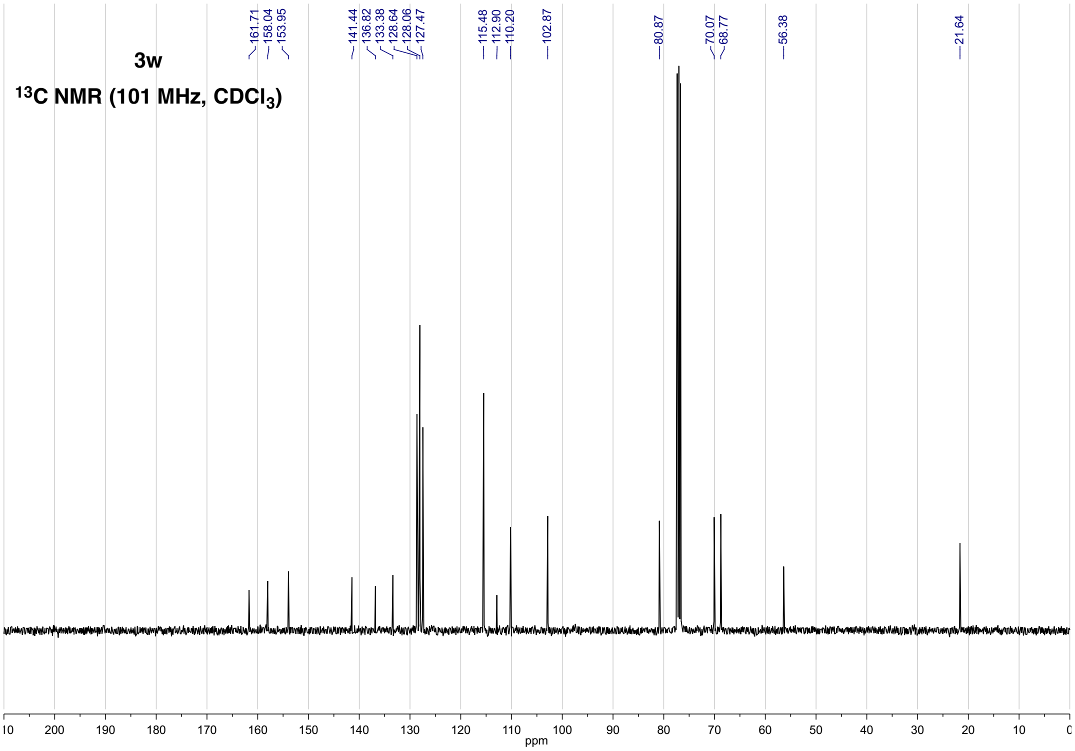
3w
^{13}C NMR (101 MHz, $CDCl_3$)
161.71 158.04 153.95
141.44 136.82 133.38 128.64 128.06 127.47
115.48 112.90 110.20
102.87
80.87
70.07 68.77
56.38
21.64
ppm

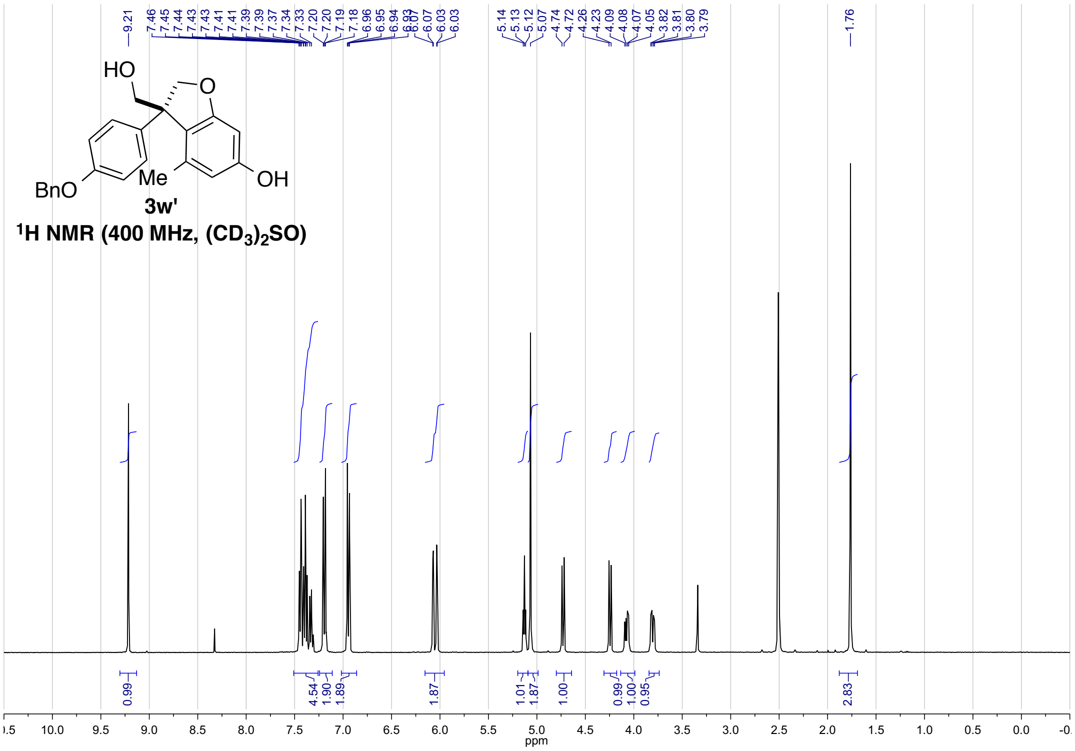
HO
O
Me
OH
BnO
3w'
¹H NMR (400 MHz, (CD3)2SO)
ppm

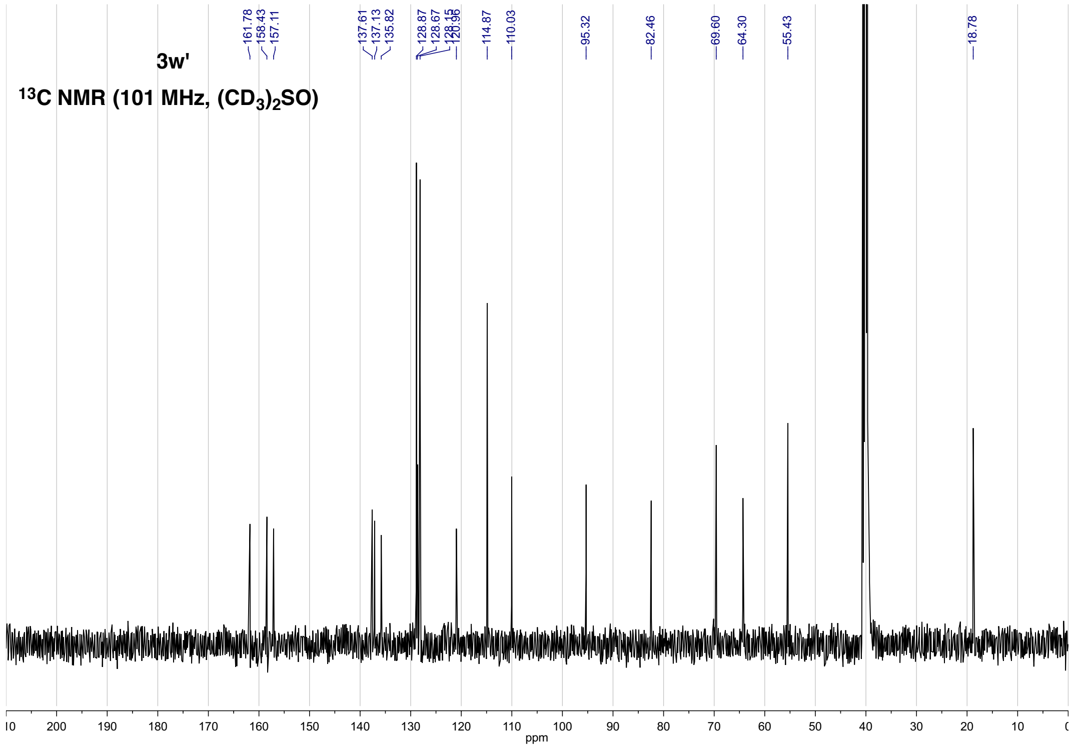
3w'
¹³C NMR (101 MHz, (CD3)2SO)
ppm

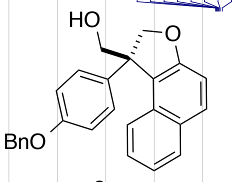

3x

^{1}H NMR (400 MHz, CDCl$_3$)

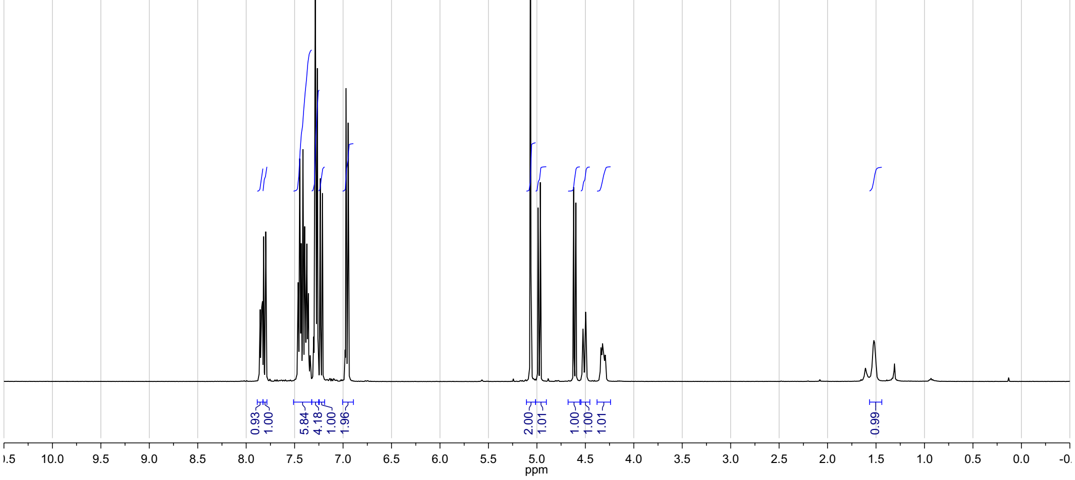

3x

^{13}C NMR (101 MHz, CDCl$_3$)

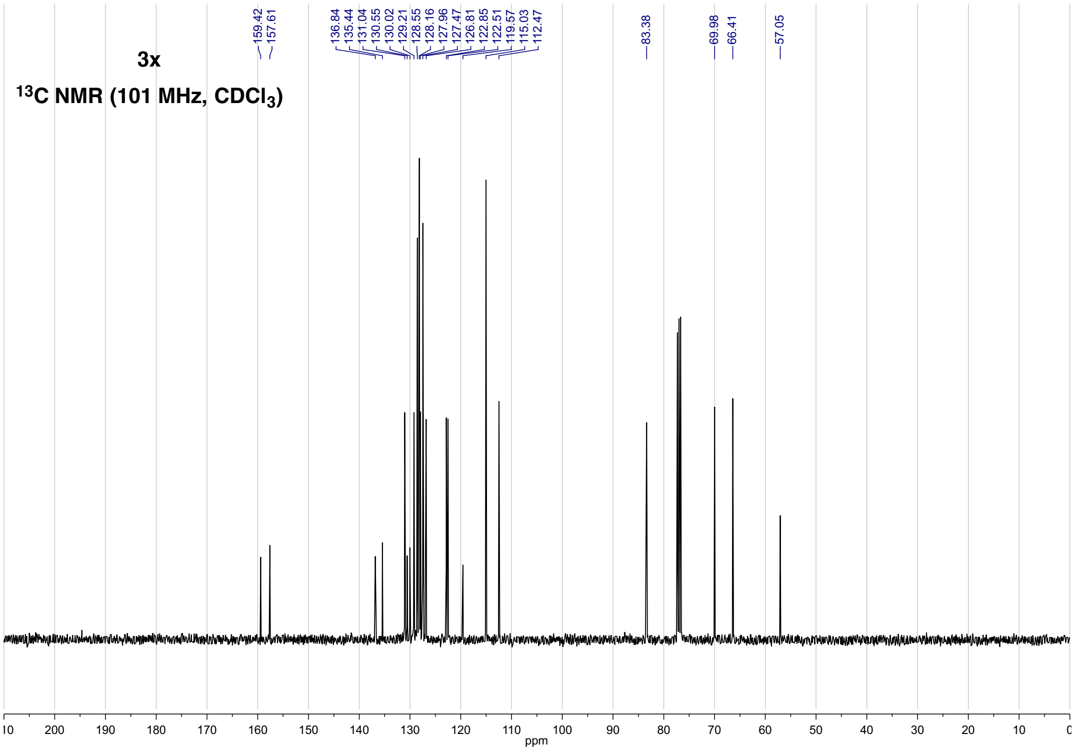

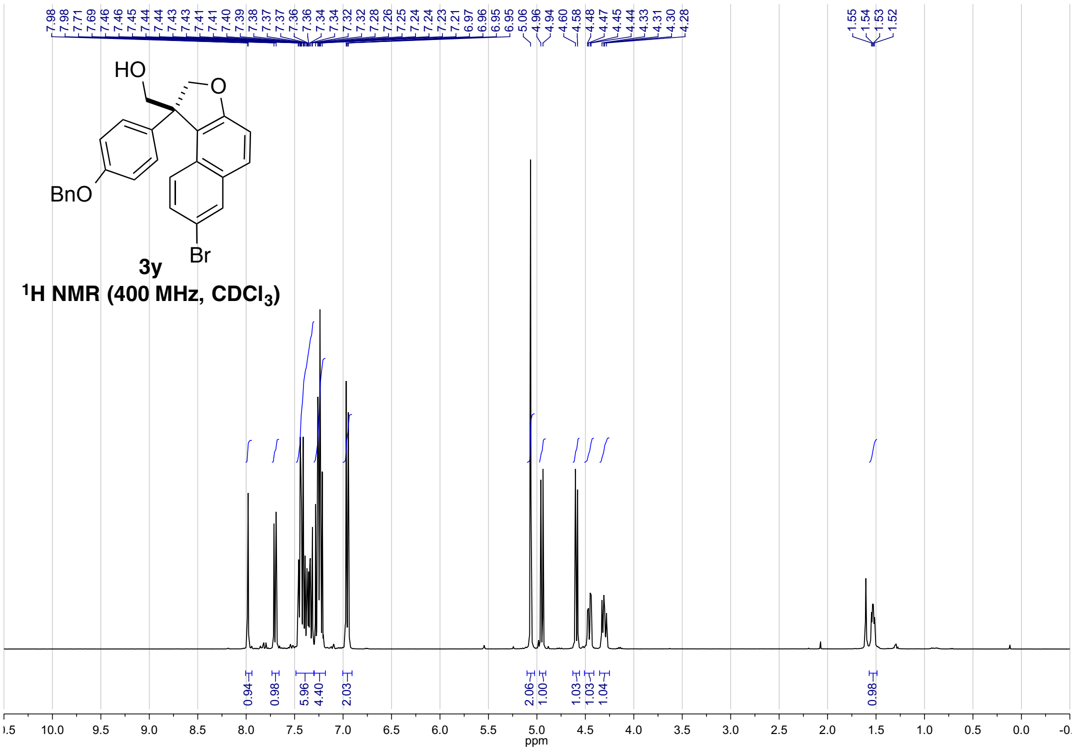
7.98 7.98 7.71 7.69 7.46 7.46 7.45 7.44 7.44 7.43 7.43 7.41 7.41 7.40 7.39 7.38 7.37 7.37 7.36 7.36 7.34 7.34 7.32 7.32 7.28 7.26 7.25 7.24 7.24 7.23 7.21 6.97 6.96 6.95 6.95 5.06 4.96 4.94 4.60 4.58 4.48 4.47 4.45 4.44 4.33 4.31 4.30 4.28
1.55 1.54 1.53 1.52
HO
O
BnO
Br
3y
1H NMR (400 MHz, CDCl3)
0.94 0.98 5.96 4.40 2.03 2.06 1.00 1.03 1.03 1.04 0.98
ppm

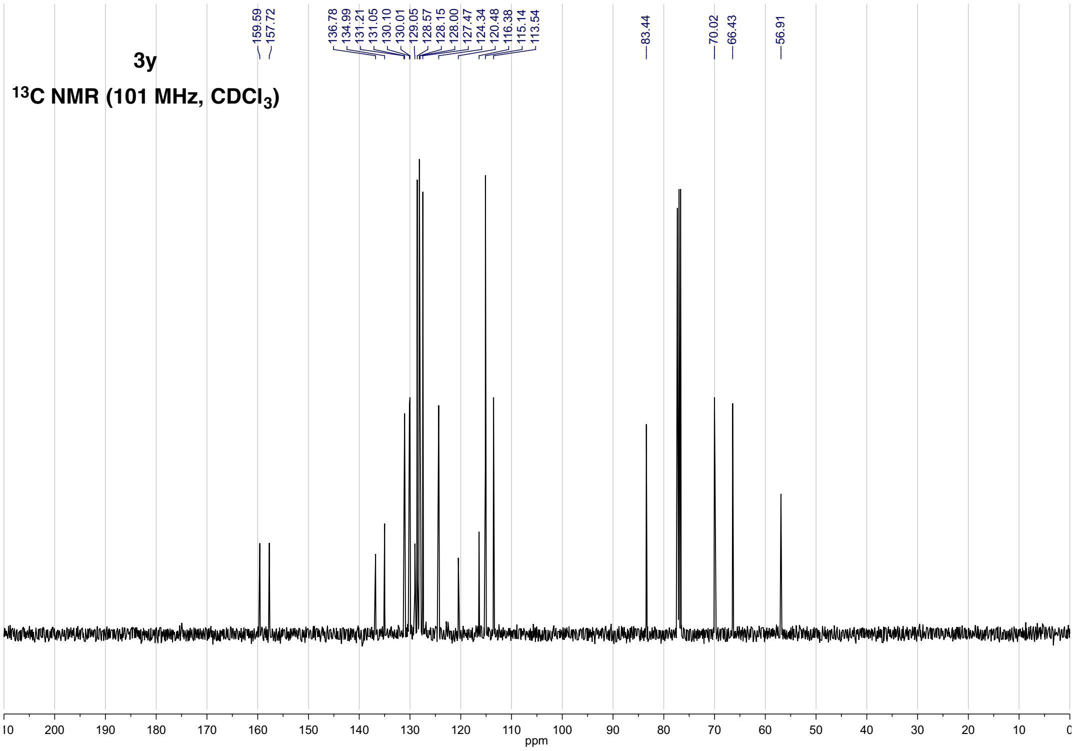
159.59 157.72
136.78 134.99 131.21 131.05 130.10 130.01 129.05 128.57 128.15 128.00 127.47 124.34 120.48 116.38 115.14 113.54
83.44
70.02 66.43
56.91
3y
13C NMR (101 MHz, CDCl3)
ppm

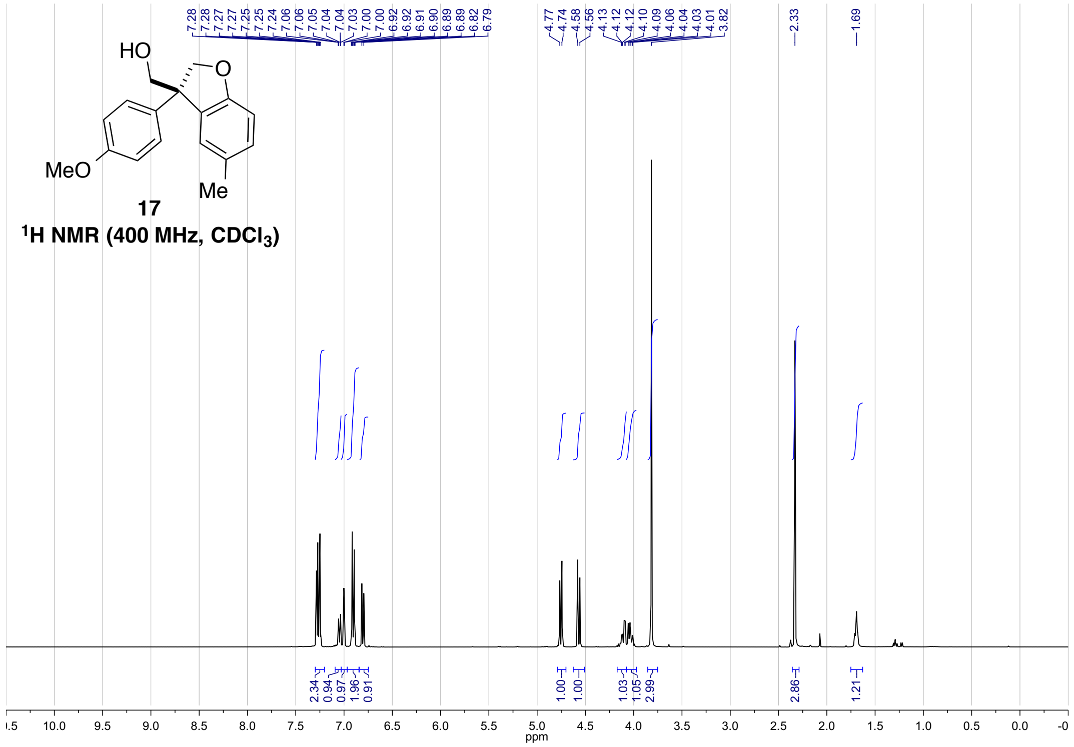

17

^{1}H NMR (400 MHz, $CDCl_3$)

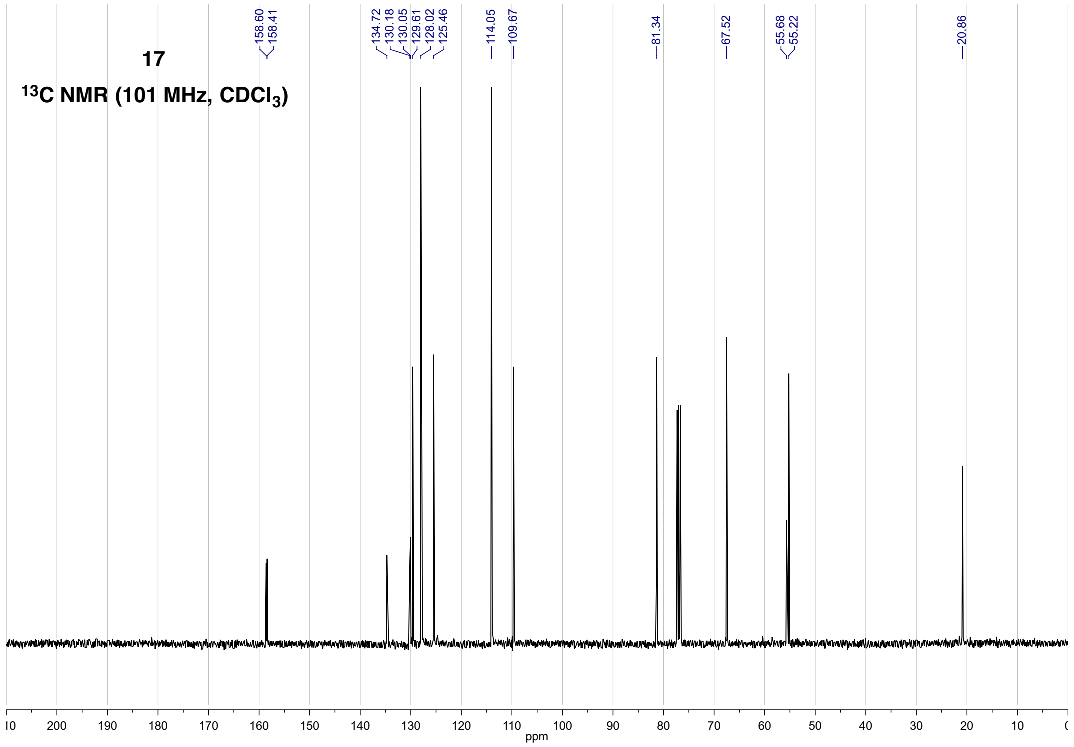

17

^{13}C NMR (101 MHz, $CDCl_3$)

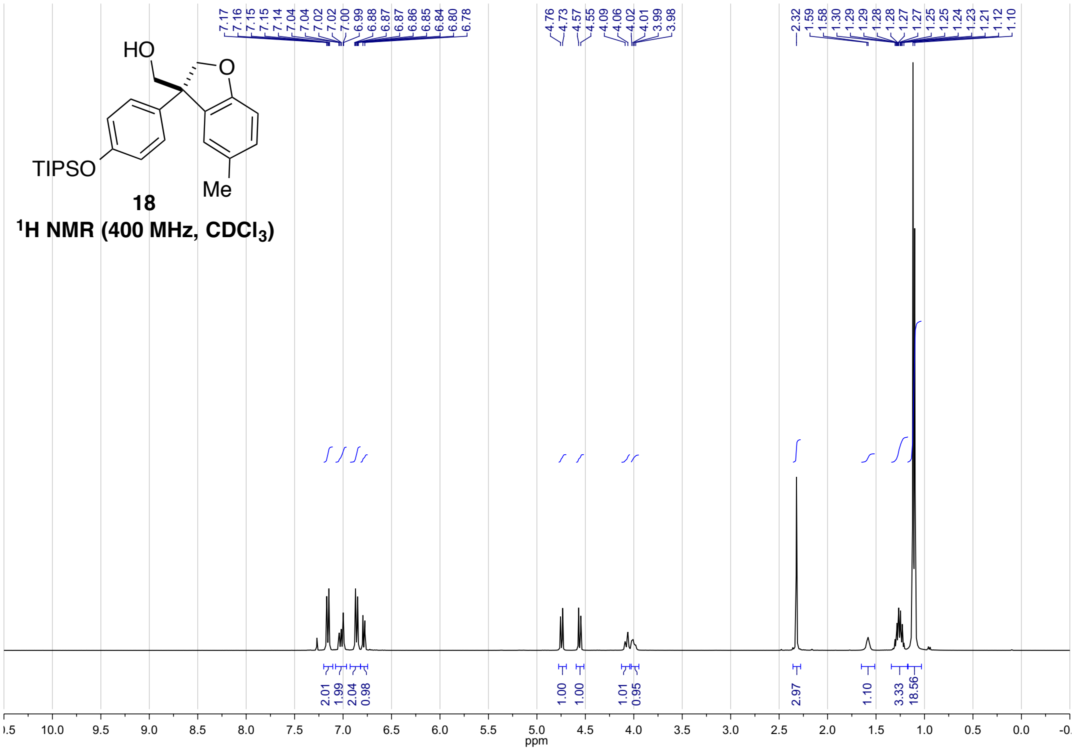
HO
O
TIPSO
Me
18
^{1}H NMR (400 MHz, $CDCl_3$)
ppm

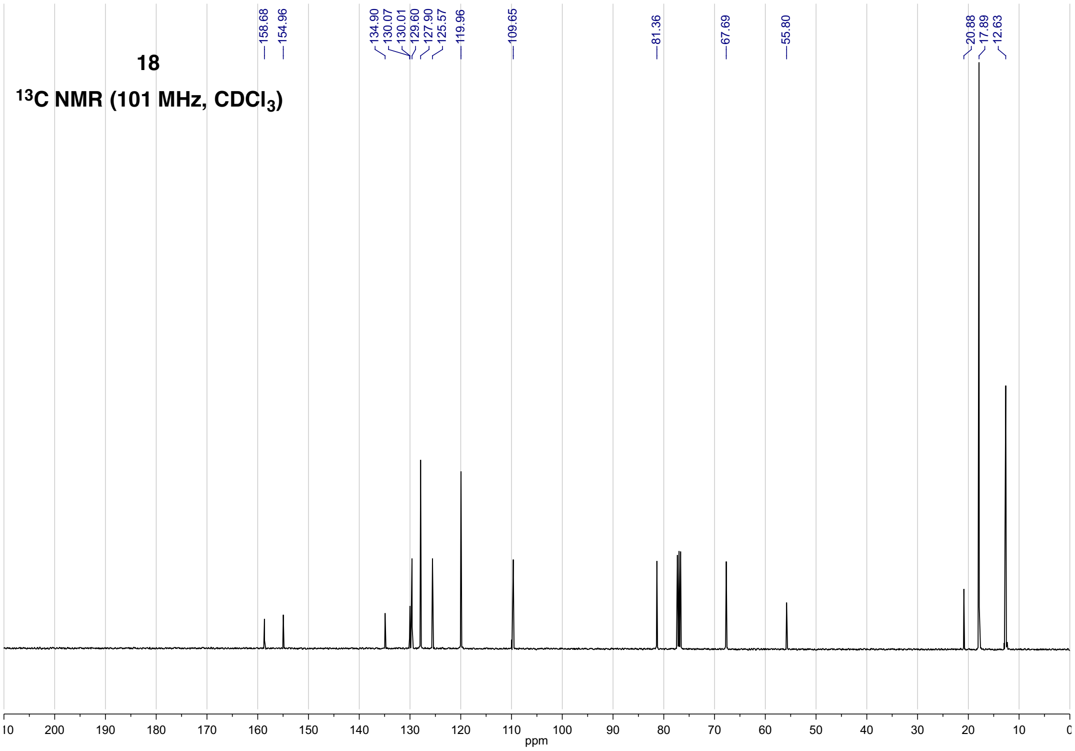
18
^{13}C NMR (101 MHz, $CDCl_3$)
ppm

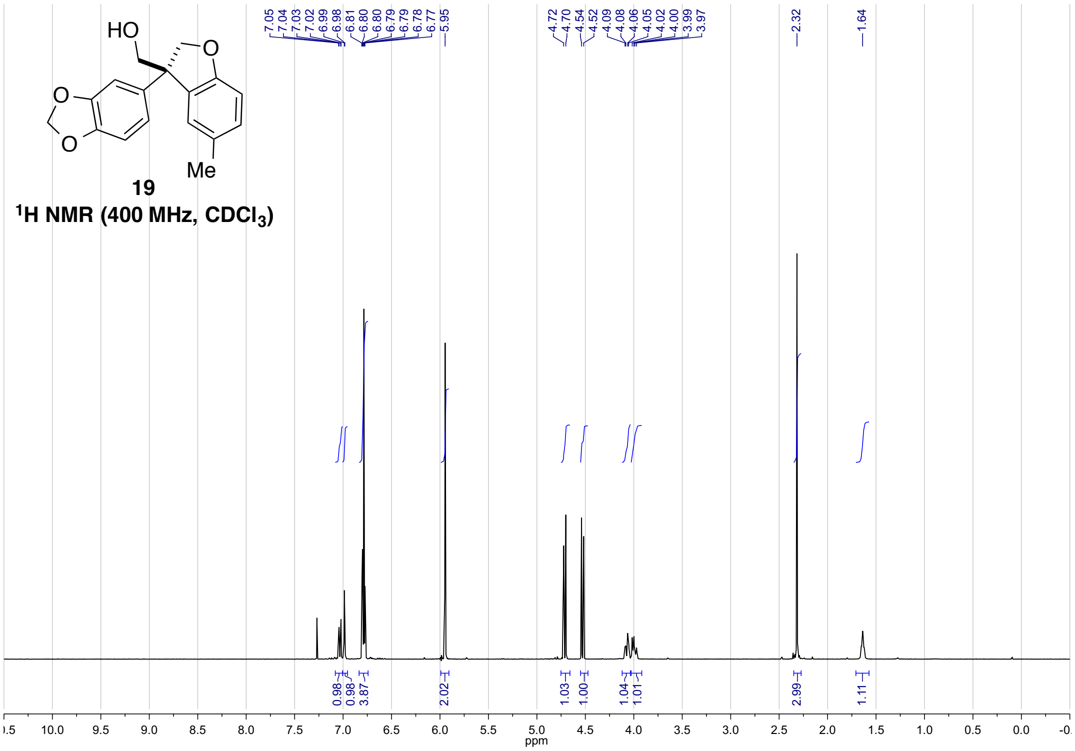
HO
O
O
O
Me
19
¹H NMR (400 MHz, CDCl₃)
7.05 7.04 7.03 7.02 6.99 6.98 6.81 6.80 6.80 6.79 6.79 6.78 6.77 5.95
4.72 4.70 4.54 4.52 4.09 4.08 4.06 4.05 4.02 4.00 3.99 3.97 2.32 1.64
0.98 0.98 3.87 2.02 1.03 1.00 1.04 1.01 2.99 1.11
ppm

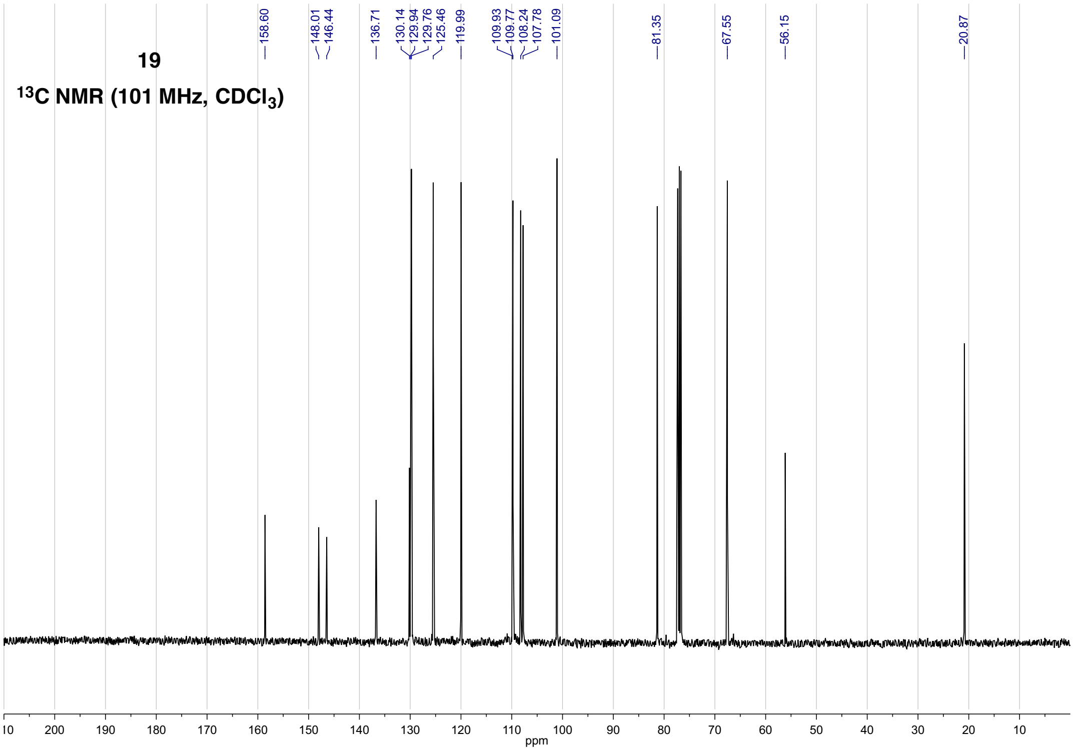
19
¹³C NMR (101 MHz, CDCl₃)
158.60 148.01 146.44 136.71 130.14 129.94 129.76 125.46 119.99
109.93 109.77 108.24 107.78 101.09 81.35 67.55 56.15 20.87
ppm

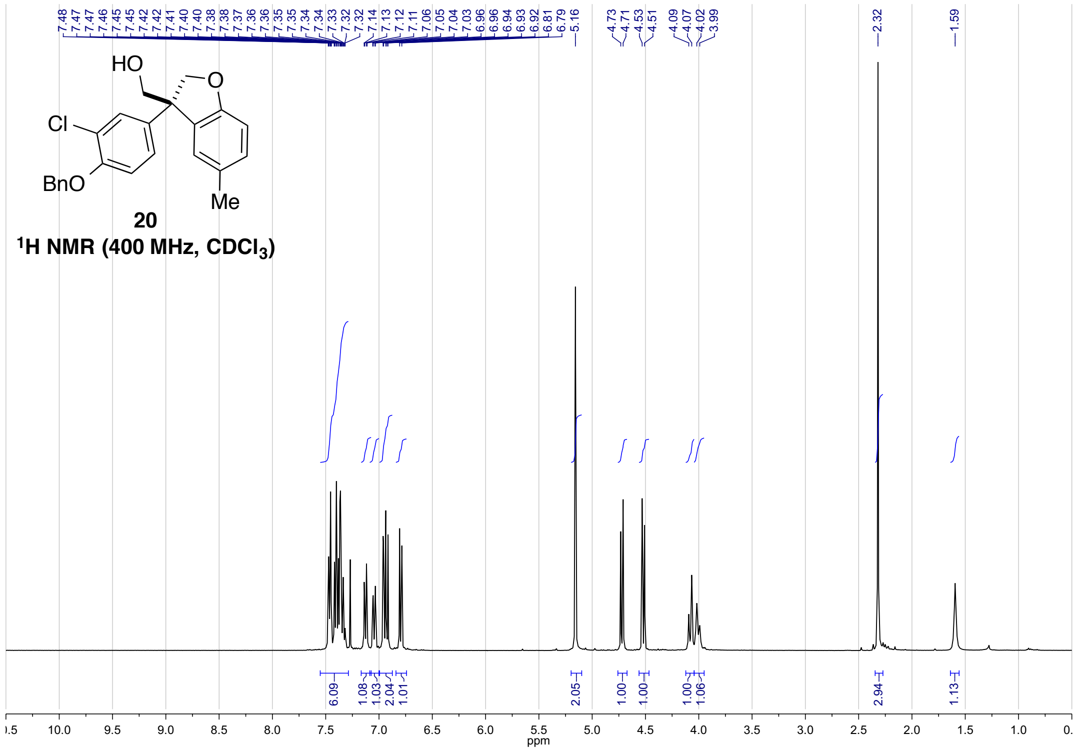
HO
O
Cl
BnO
Me
20
1H NMR (400 MHz, CDCl3)
ppm

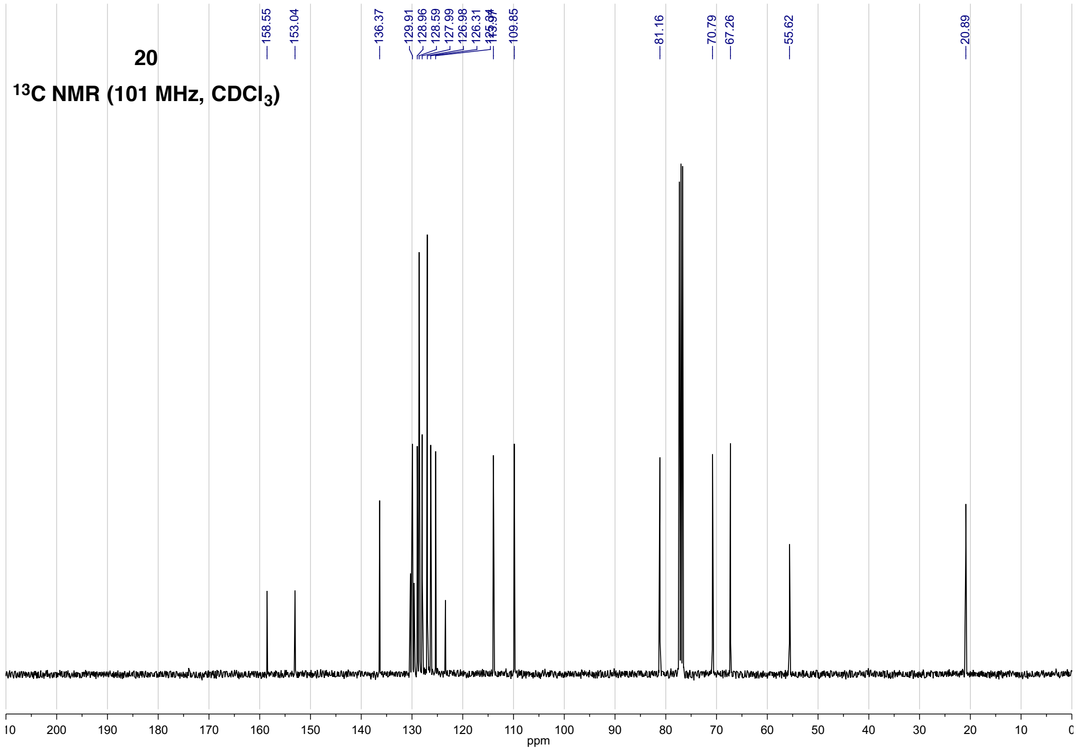
20
13C NMR (101 MHz, CDCl3)
ppm

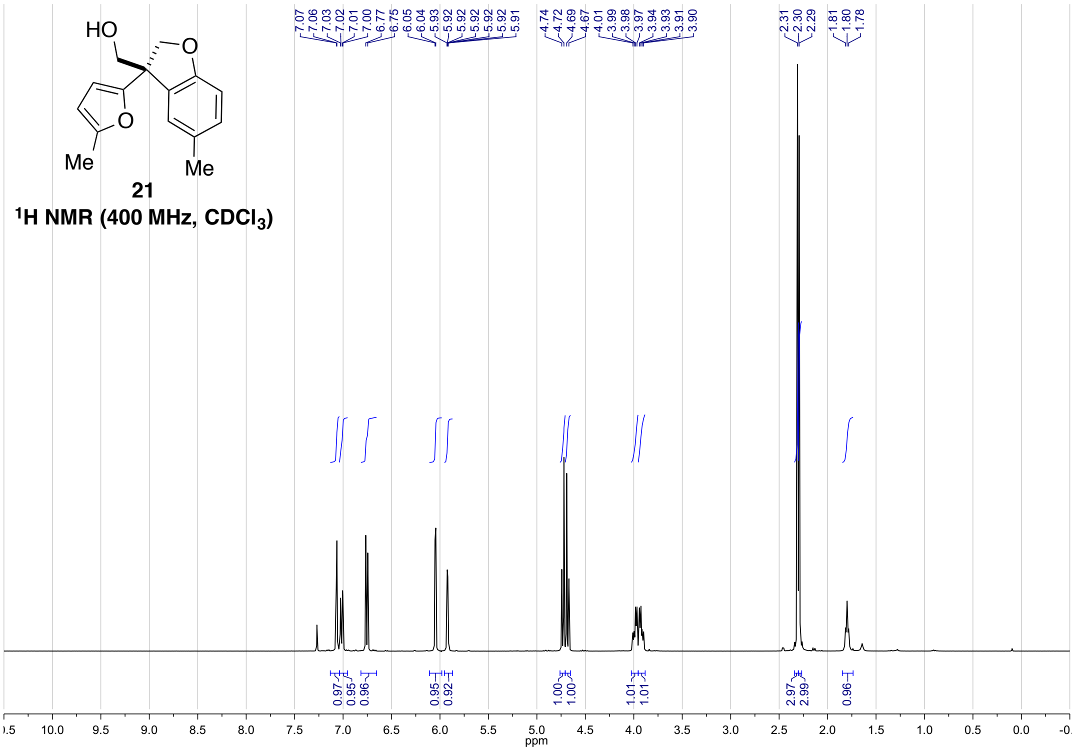

21

^{1}H NMR (400 MHz, $CDCl_3$)

21

^{13}C NMR (101 MHz, $CDCl_3$)

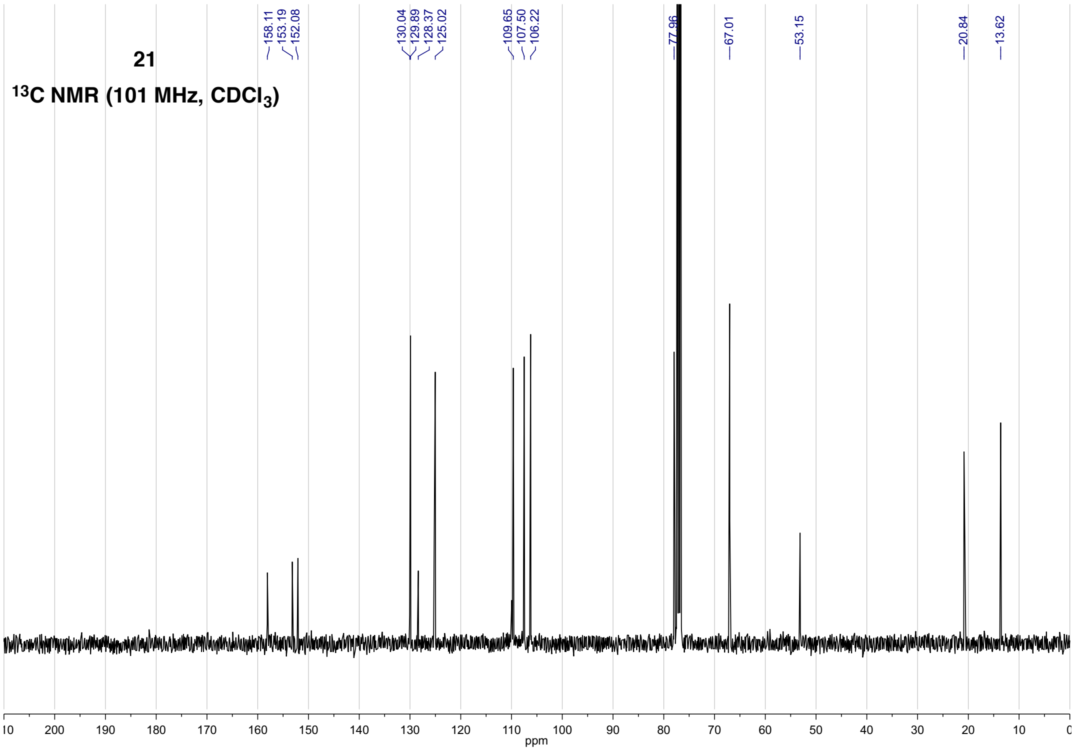

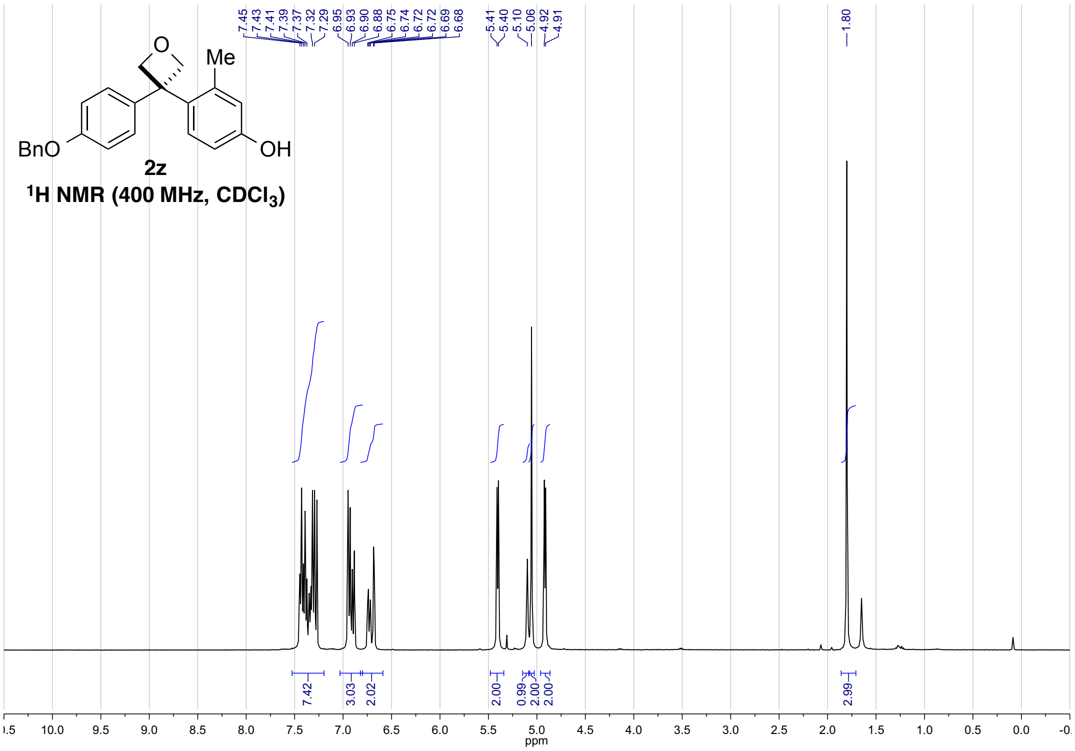
Me
BnO
OH
O
2z
¹H NMR (400 MHz, CDCl3)
ppm

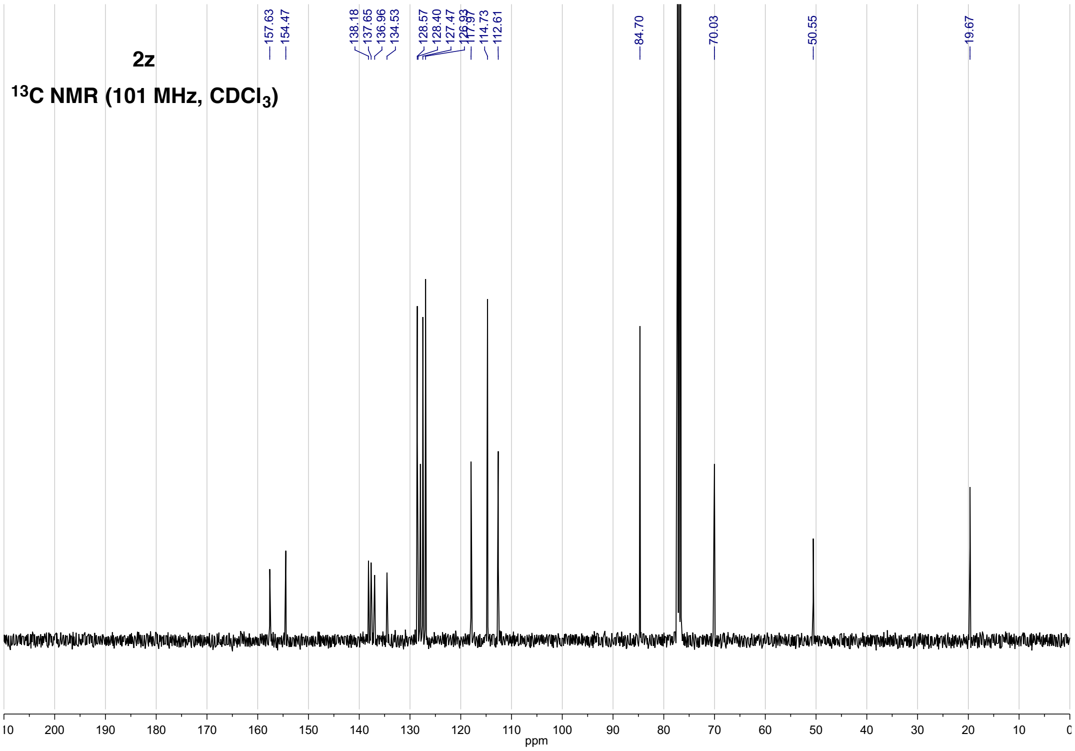
2z
¹³C NMR (101 MHz, CDCl3)
ppm

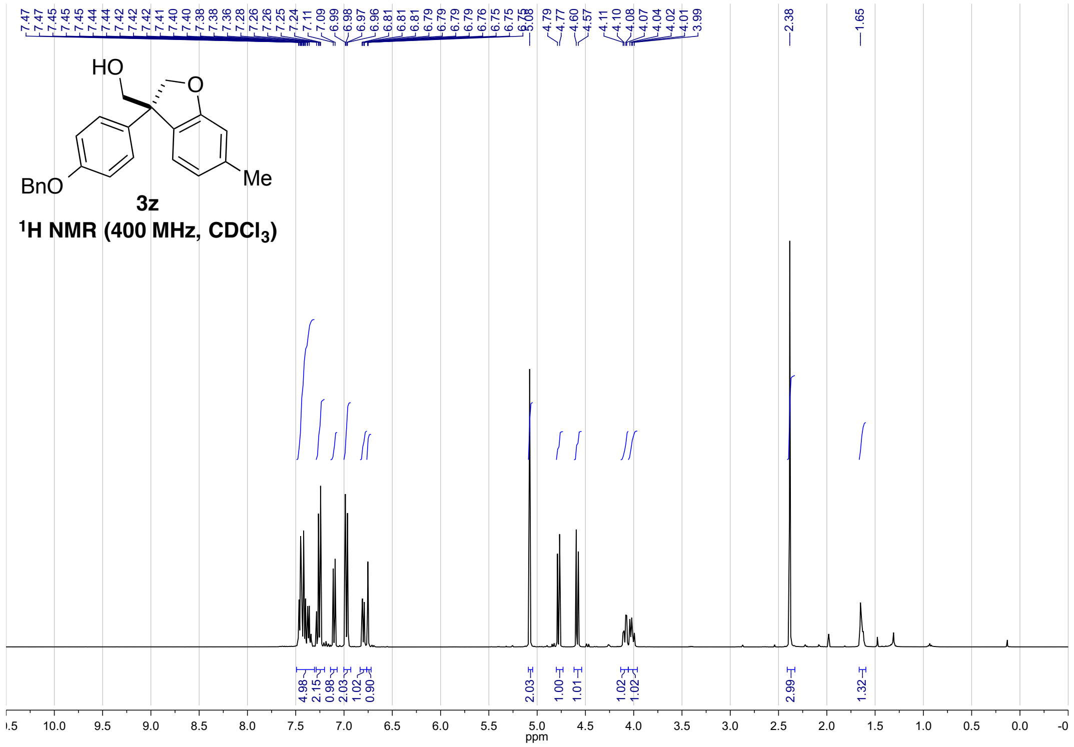
HO
O
Me
BnO
3z
1H NMR (400 MHz, CDCl3)
ppm

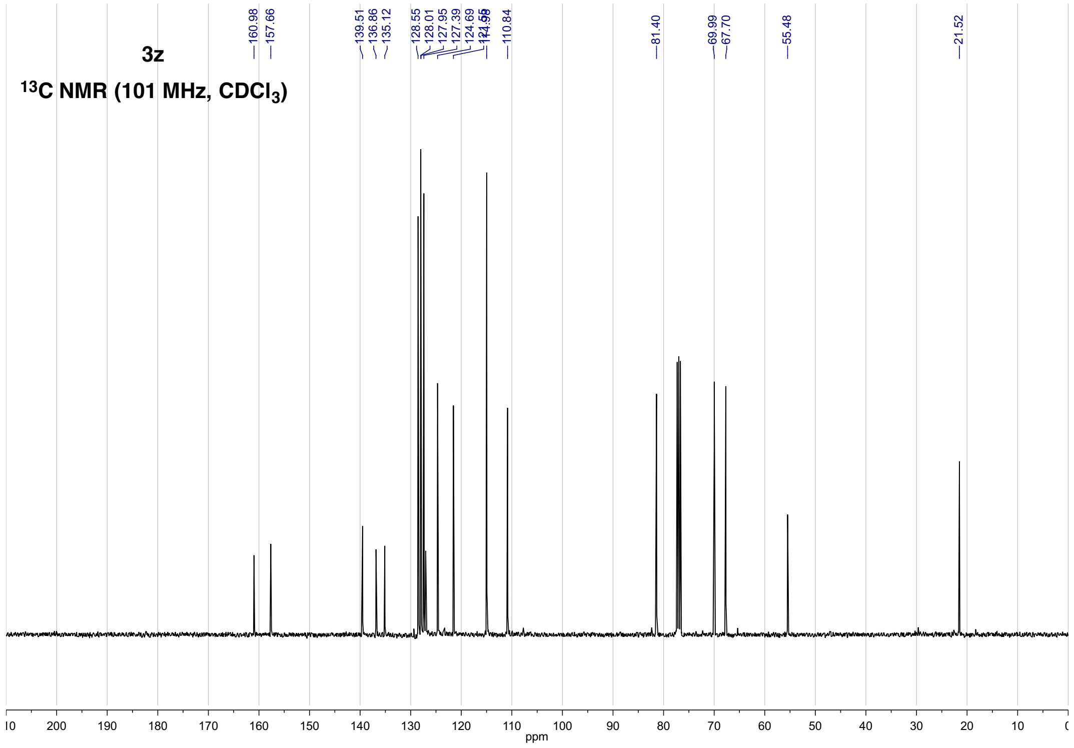
3z
13C NMR (101 MHz, CDCl3)
ppm

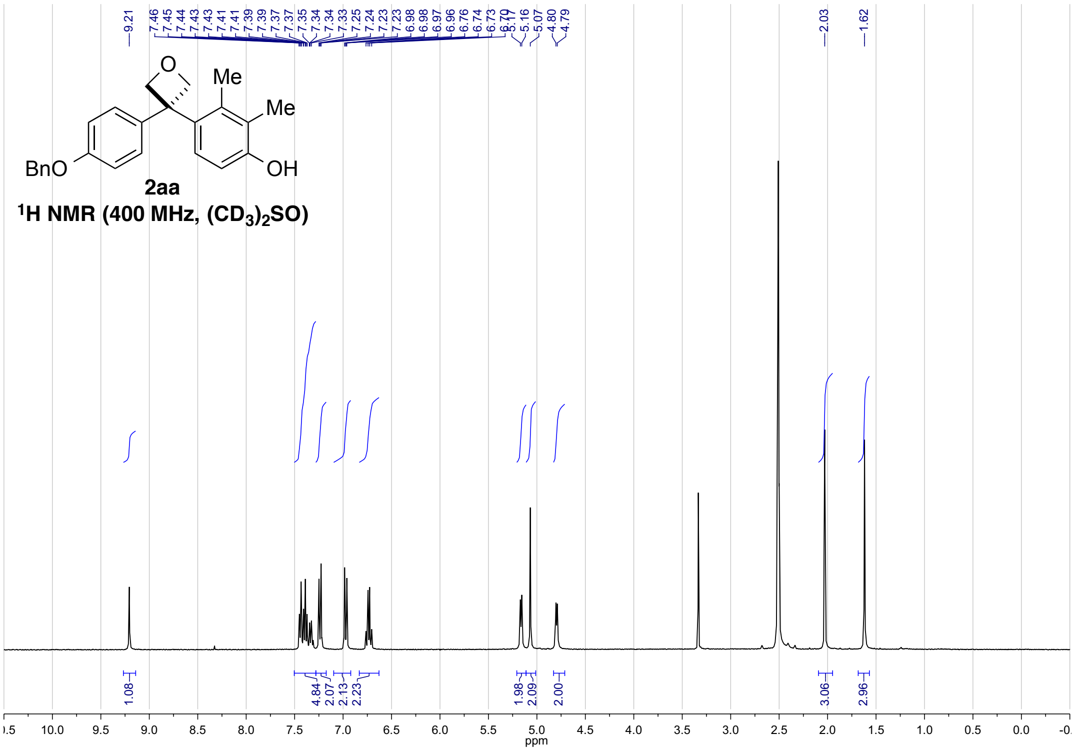
Me
Me
BnO
OH
2aa
1H NMR (400 MHz, (CD3)2SO)
9.21
7.46
7.45
7.44
7.43
7.41
7.39
7.37
7.35
7.34
7.34
7.33
7.25
7.24
7.23
7.23
6.98
6.98
6.97
6.96
6.76
6.74
6.73
6.70
5.17
5.16
5.07
4.80
4.79
2.03
1.62
1.08
4.84
2.07
2.13
2.23
1.98
2.09
2.00
3.06
2.96
ppm

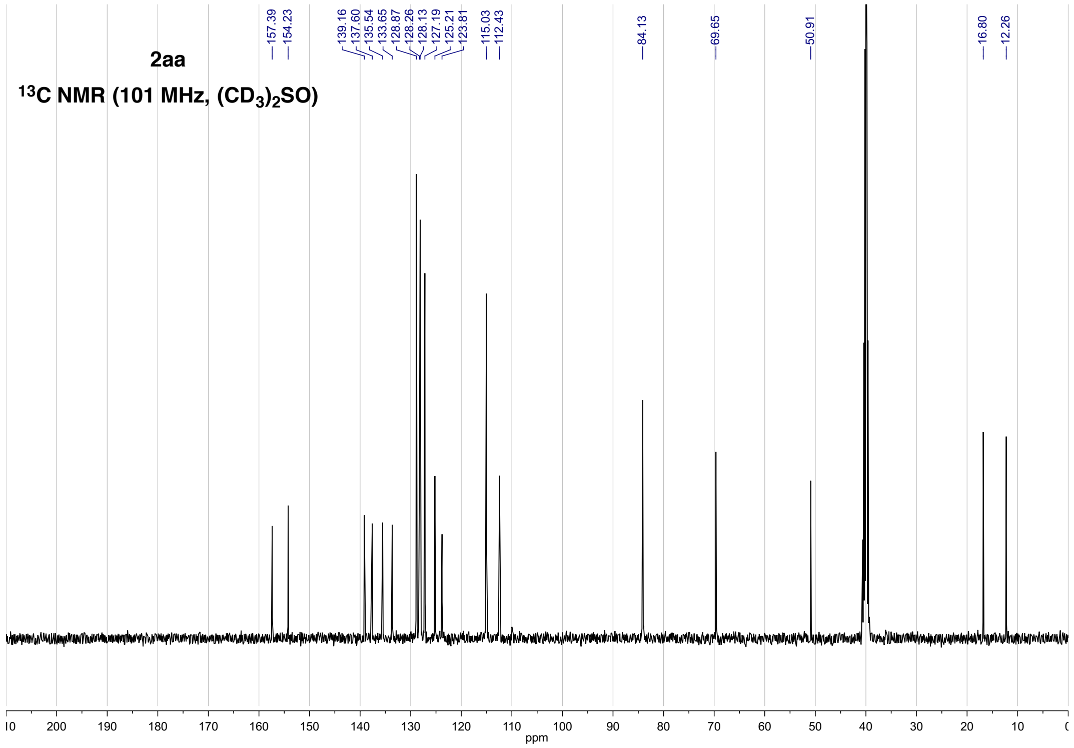
2aa
13C NMR (101 MHz, (CD3)2SO)
157.39
154.23
139.16
137.60
135.54
133.65
128.87
128.26
128.13
127.19
125.21
123.81
115.03
112.43
84.13
69.65
50.91
16.80
12.26
ppm

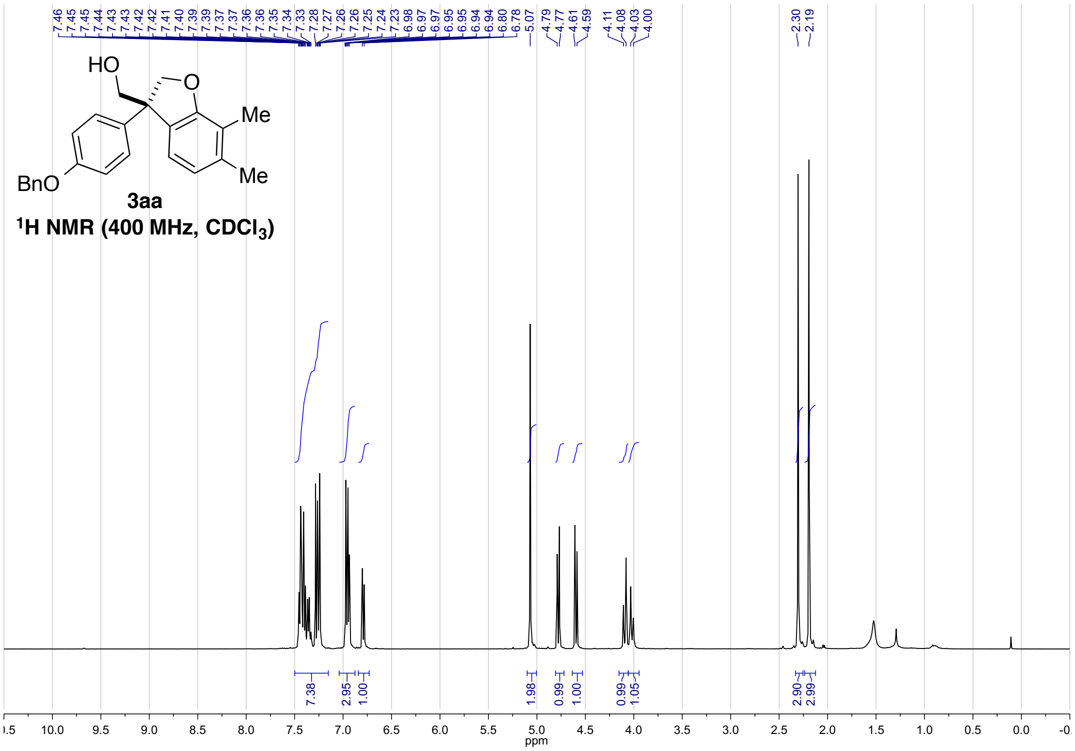
HO
O
Me
Me
BnO
3aa
1H NMR (400 MHz, CDCl3)
ppm

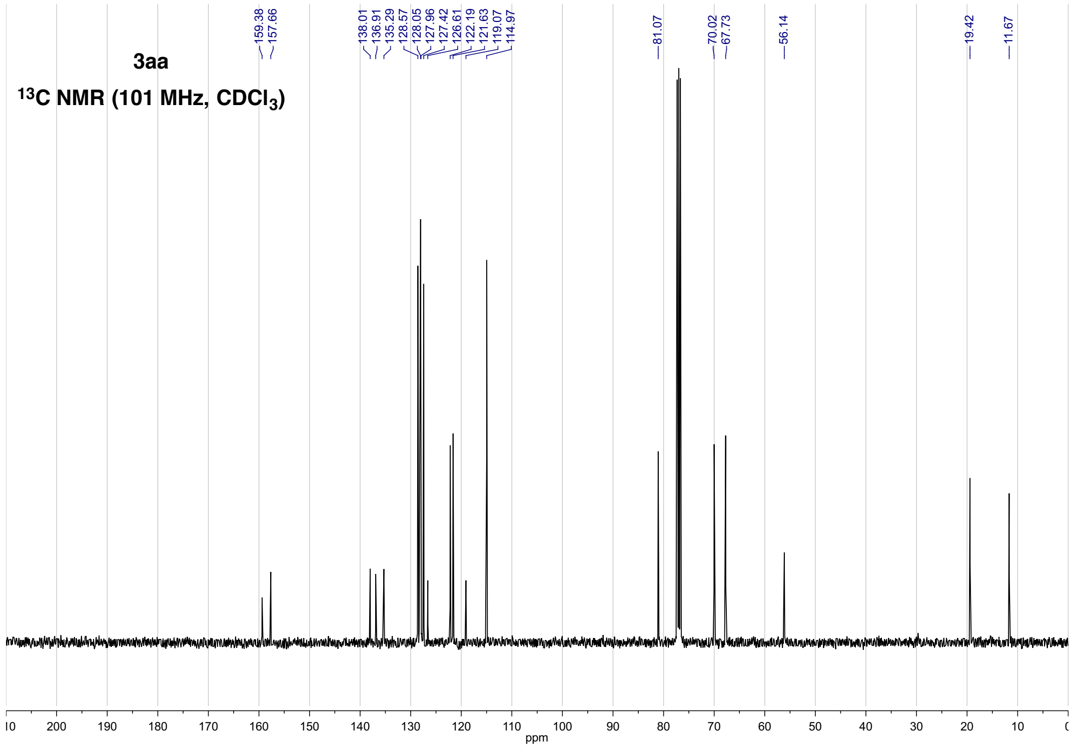
3aa
13C NMR (101 MHz, CDCl3)
ppm

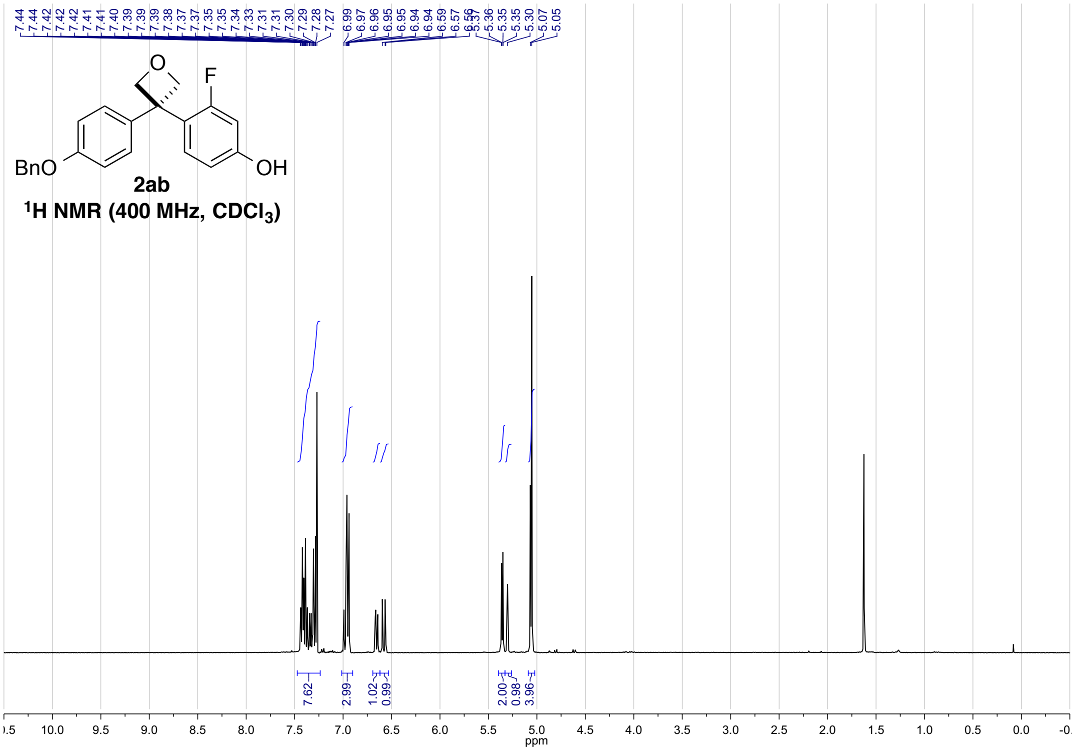

2ab

^{1}H NMR (400 MHz, $CDCl_3$)

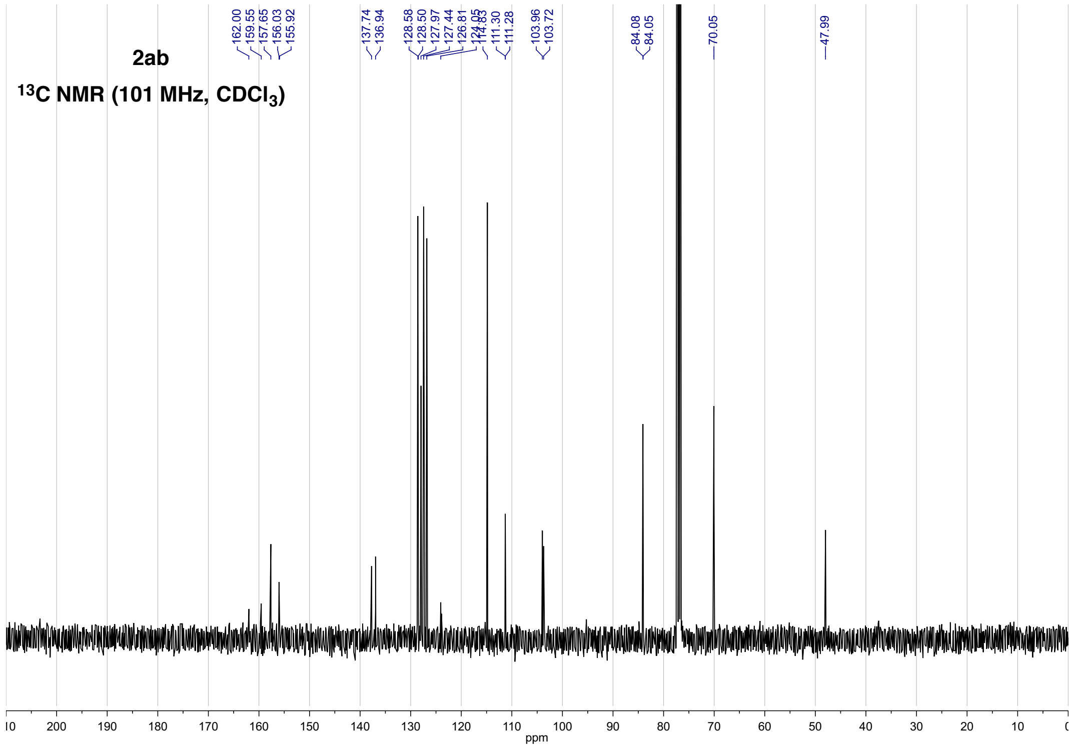

2ab

^{13}C NMR (101 MHz, $CDCl_3$)

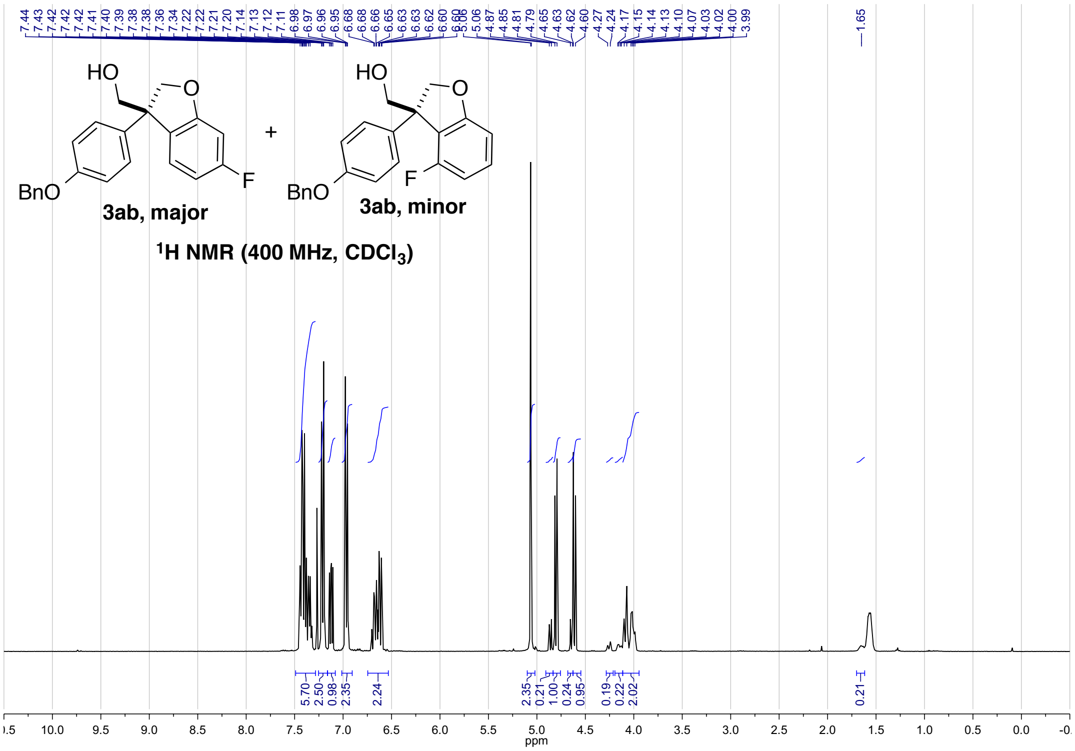
3ab, major
3ab, minor
1H NMR (400 MHz, CDCl3)
ppm

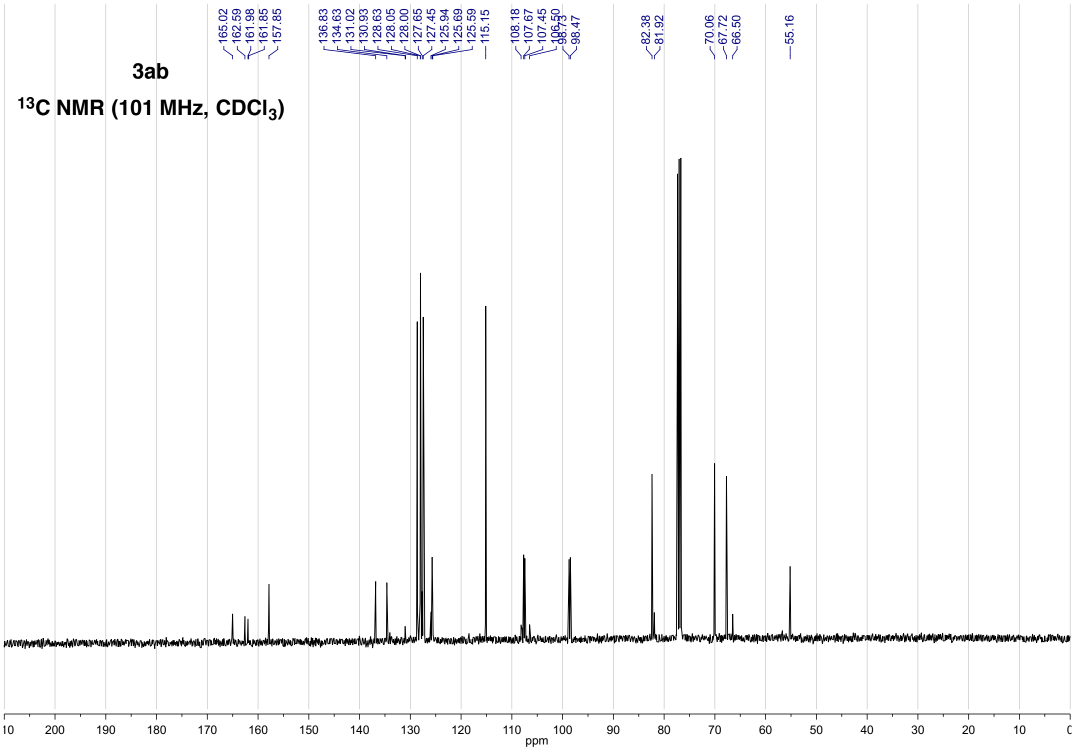
3ab
13C NMR (101 MHz, CDCl3)
ppm

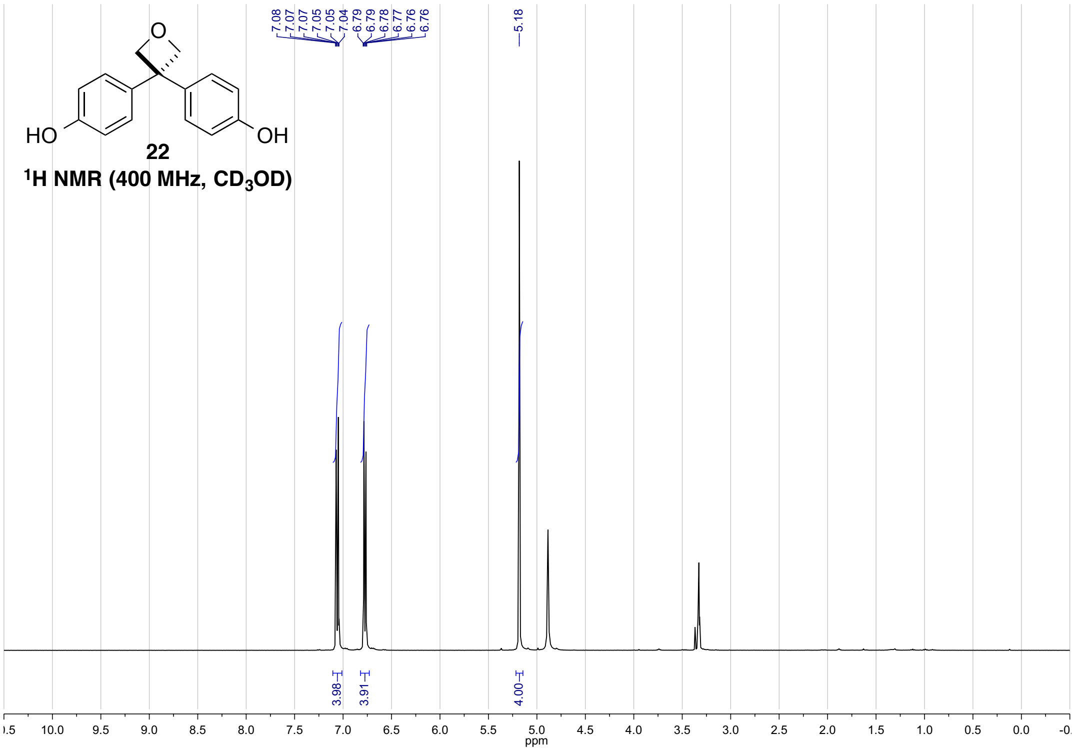
HO
OH
22
1H NMR (400 MHz, CD3OD)
7.08 7.07 7.07 7.05 7.05 7.04 6.79 6.79 6.78 6.77 6.76 6.76
5.18
3.98
3.91
4.00
ppm

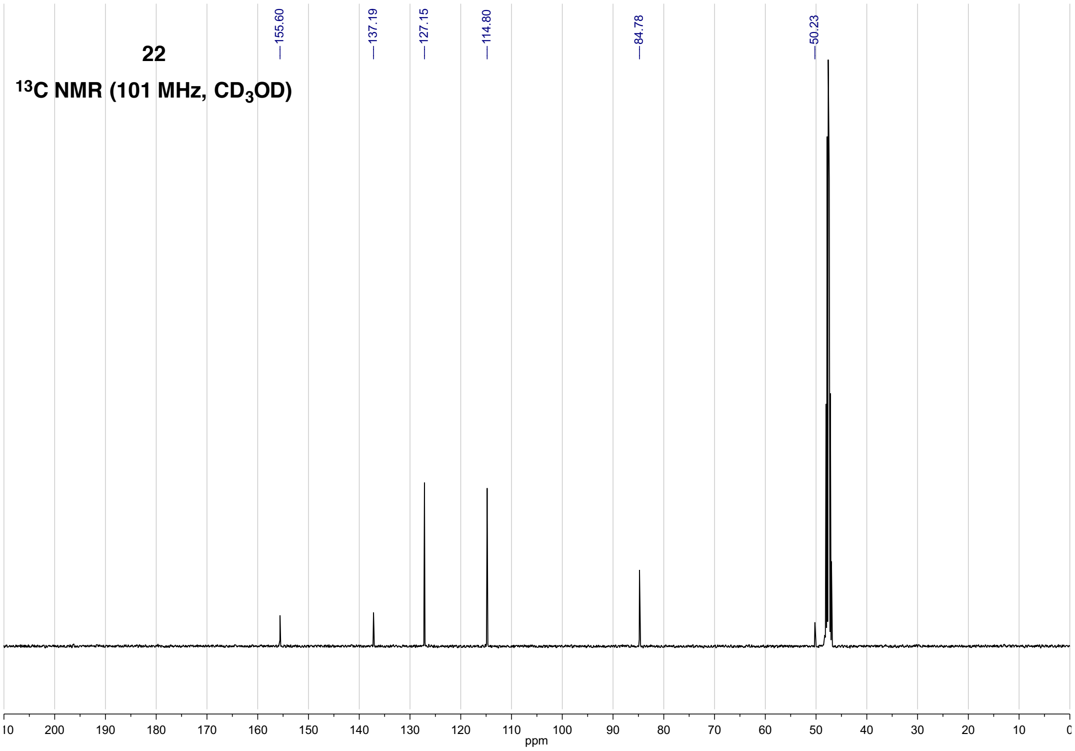
22
13C NMR (101 MHz, CD3OD)
155.60
137.19
127.15
114.80
84.78
50.23
ppm

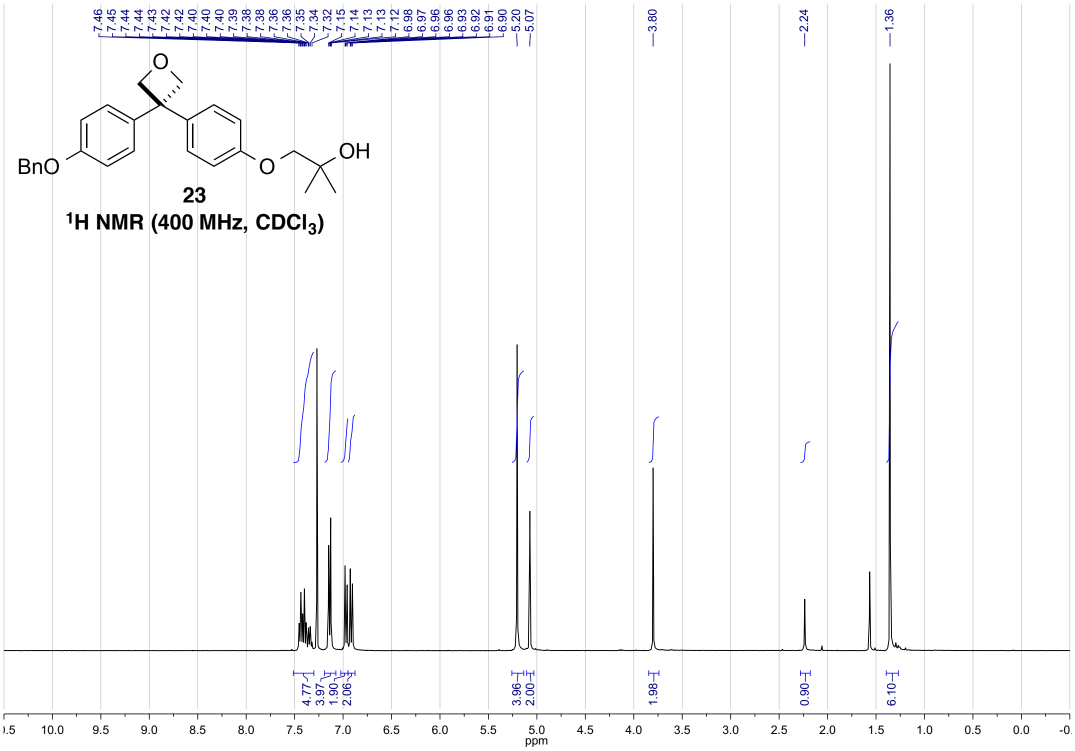
BnO
O
OH
23
^{1}H NMR (400 MHz, $CDCl_3$)
ppm

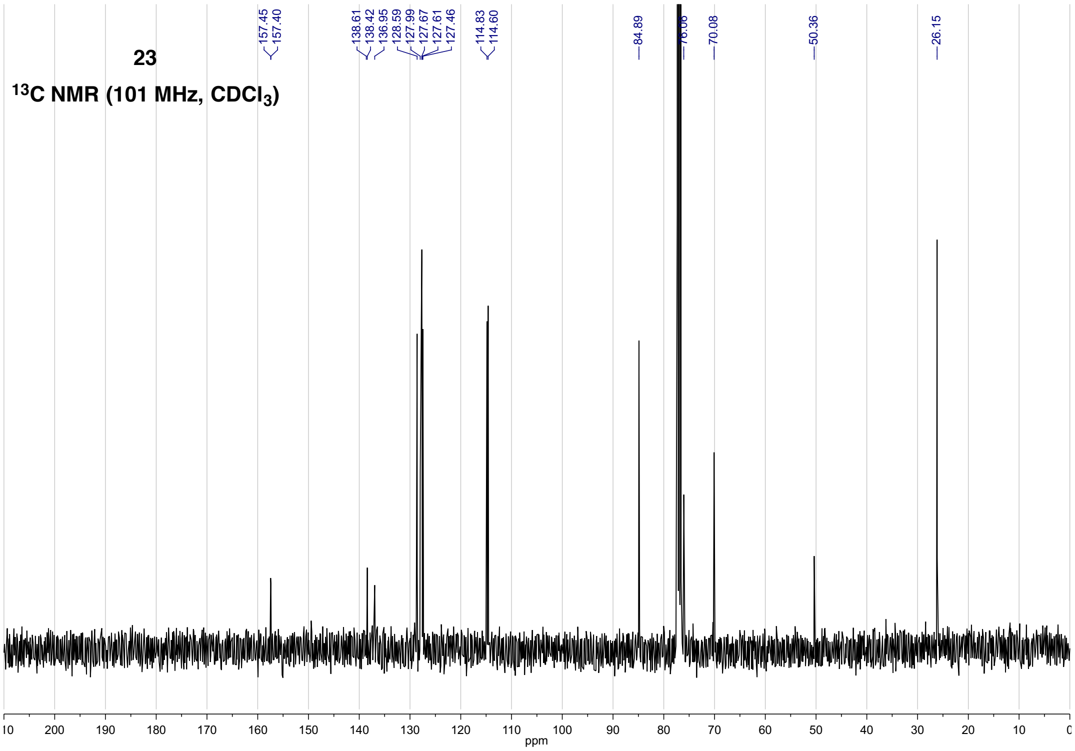
23
^{13}C NMR (101 MHz, $CDCl_3$)
ppm

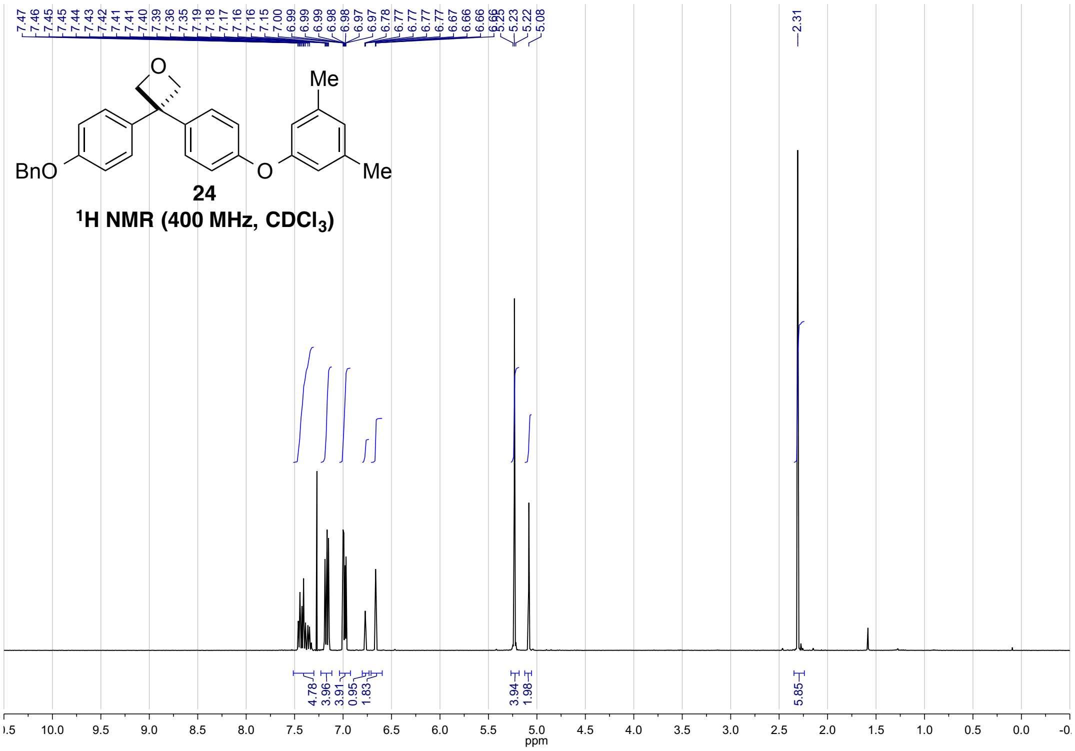
BnO
O
Me
Me
24
^{1}H NMR (400 MHz, CDCl$_3$)
ppm

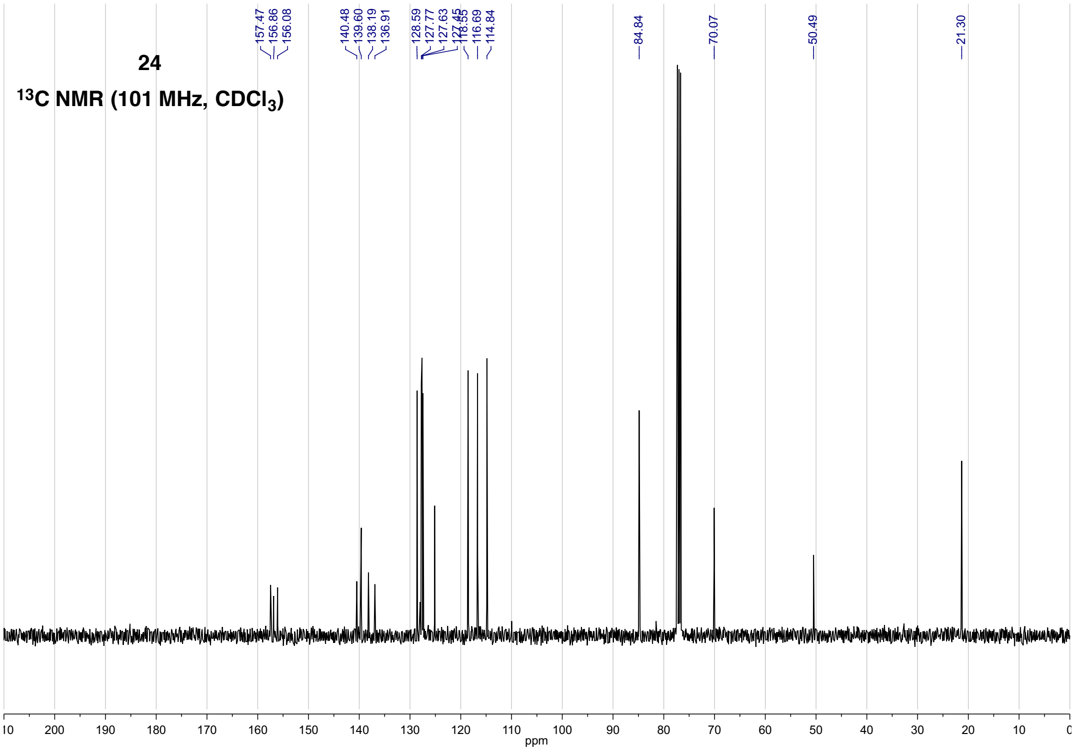
24
^{13}C NMR (101 MHz, CDCl$_3$)
ppm

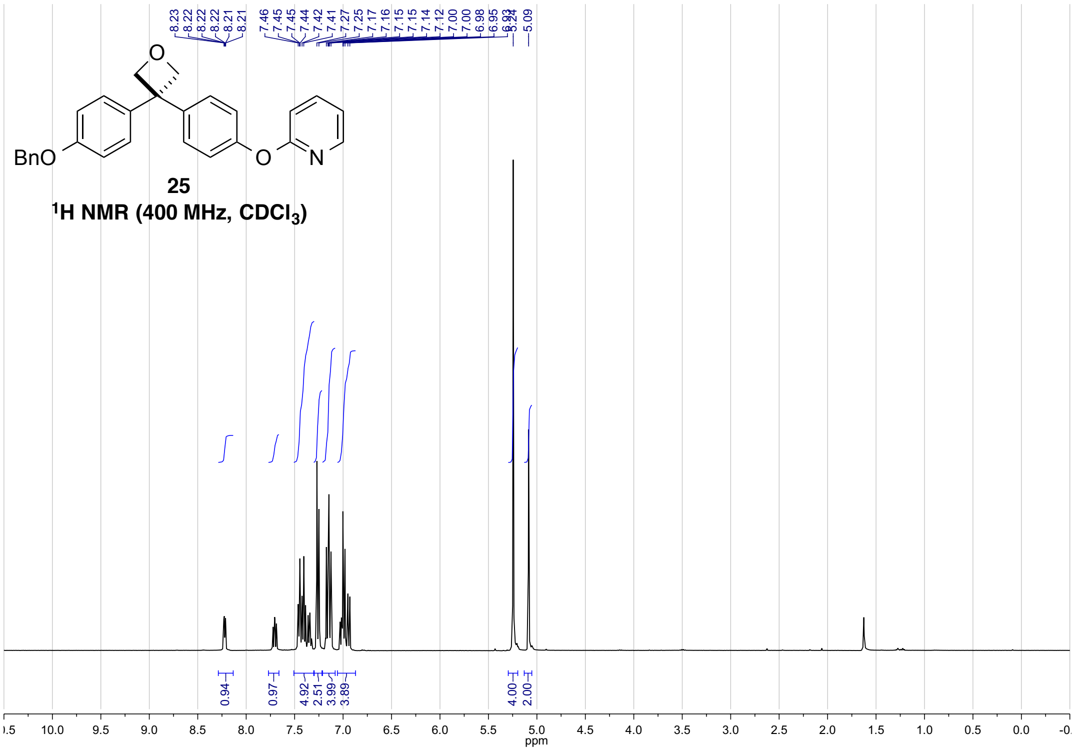
BnO
O
O
N
25
^{1}H NMR (400 MHz, CDCl$_3$)
0.94
0.97
4.92
2.51
3.99
3.89
4.00
2.00
5.24
5.09
ppm

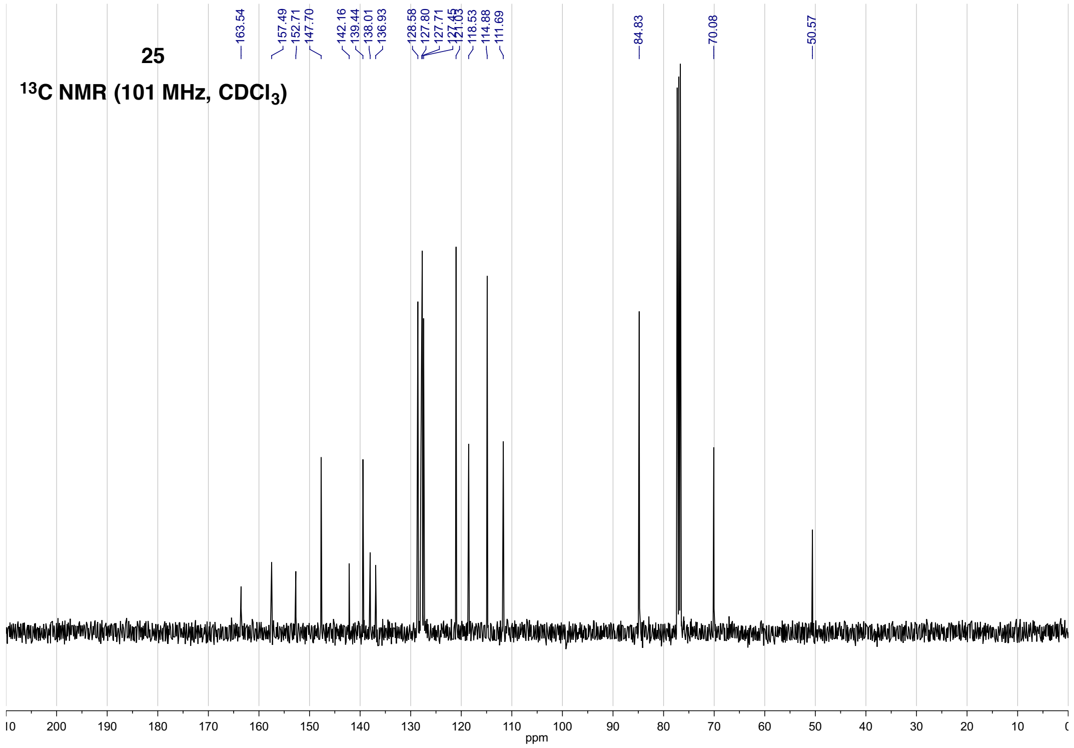
25
^{13}C NMR (101 MHz, CDCl$_3$)
163.54
157.49
152.71
147.70
142.16
139.44
138.01
136.93
128.58
127.80
127.71
118.53
114.88
111.69
84.83
70.08
50.57
ppm

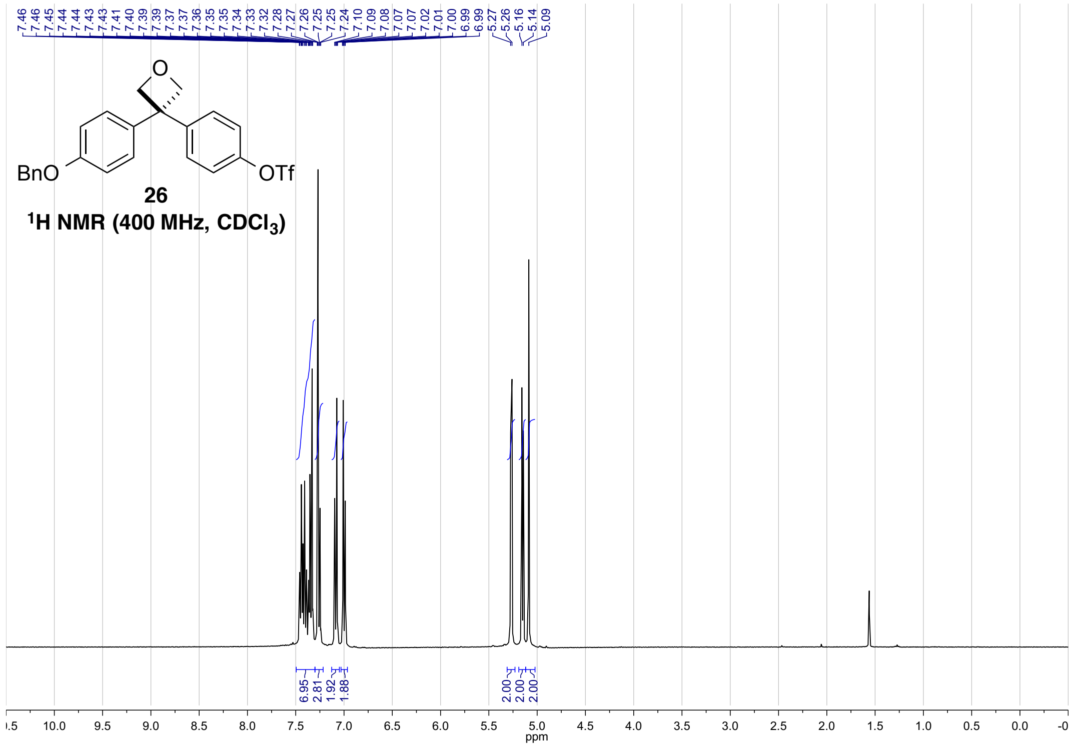
BnO
OTf
26
^{1}H NMR (400 MHz, CDCl$_3$)
ppm

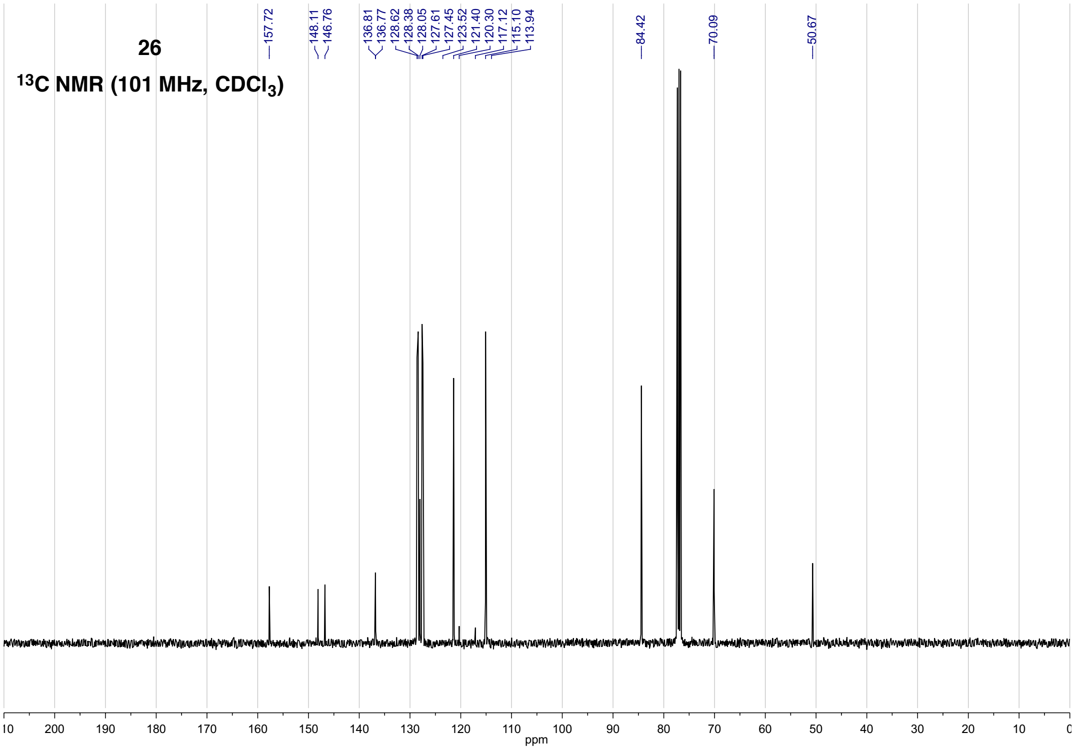
26
^{13}C NMR (101 MHz, CDCl$_3$)
ppm

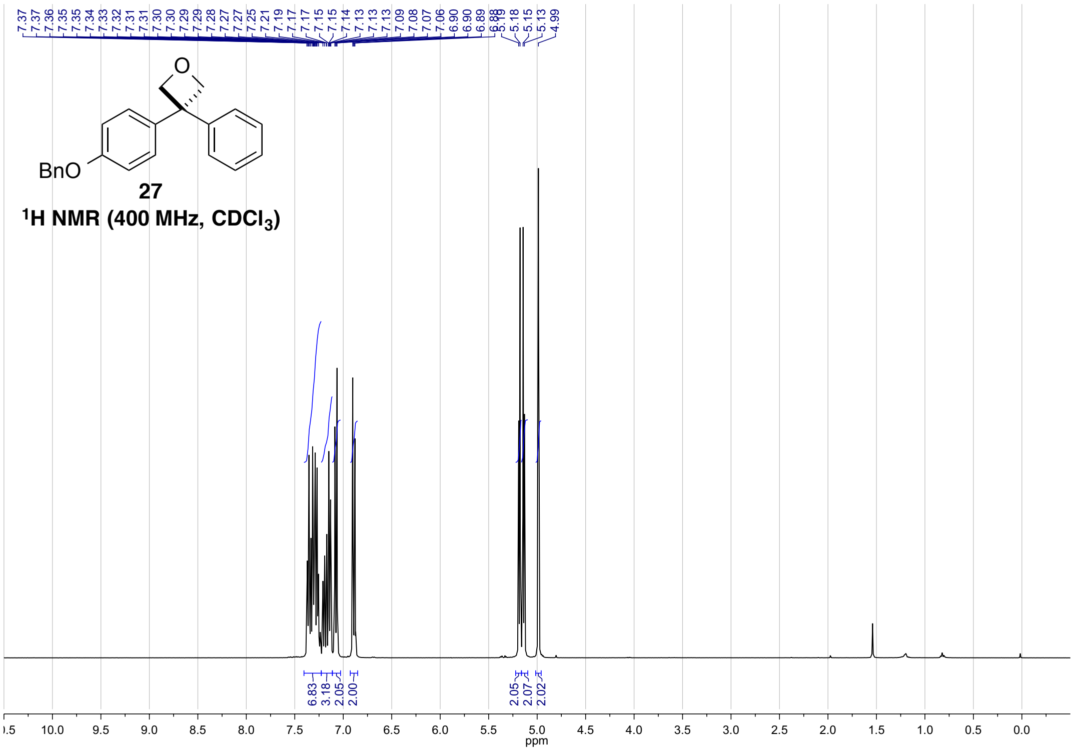
BnO
O
27
^{1}H NMR (400 MHz, $CDCl_3$)
ppm

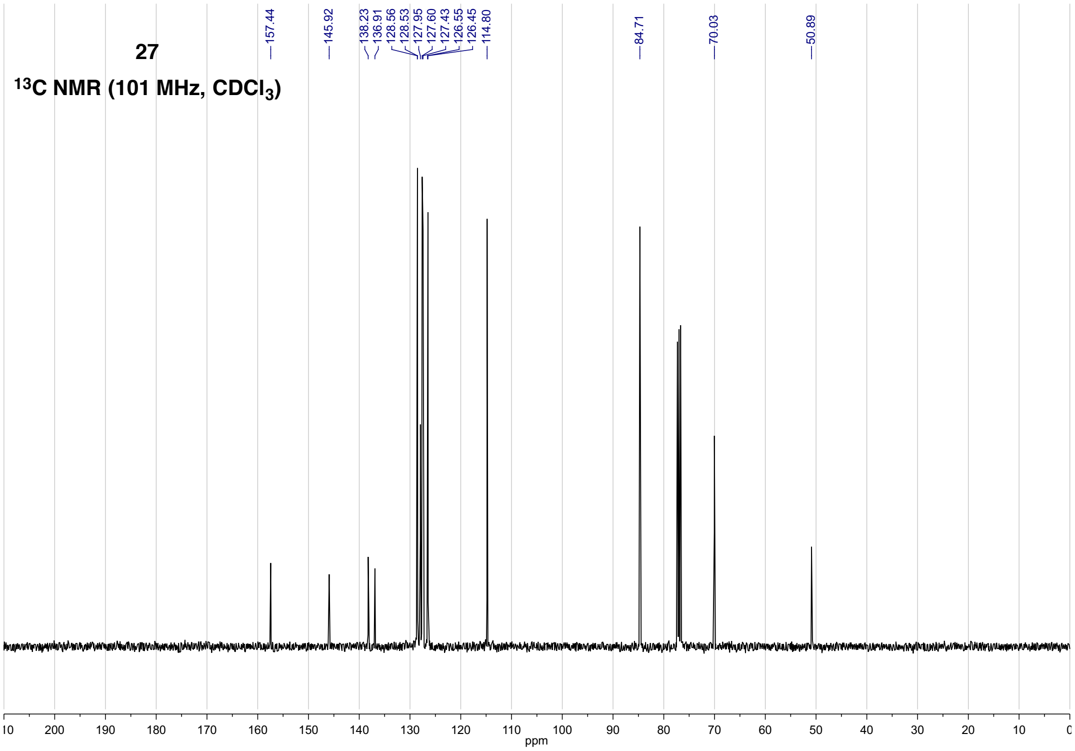
27
^{13}C NMR (101 MHz, $CDCl_3$)
ppm

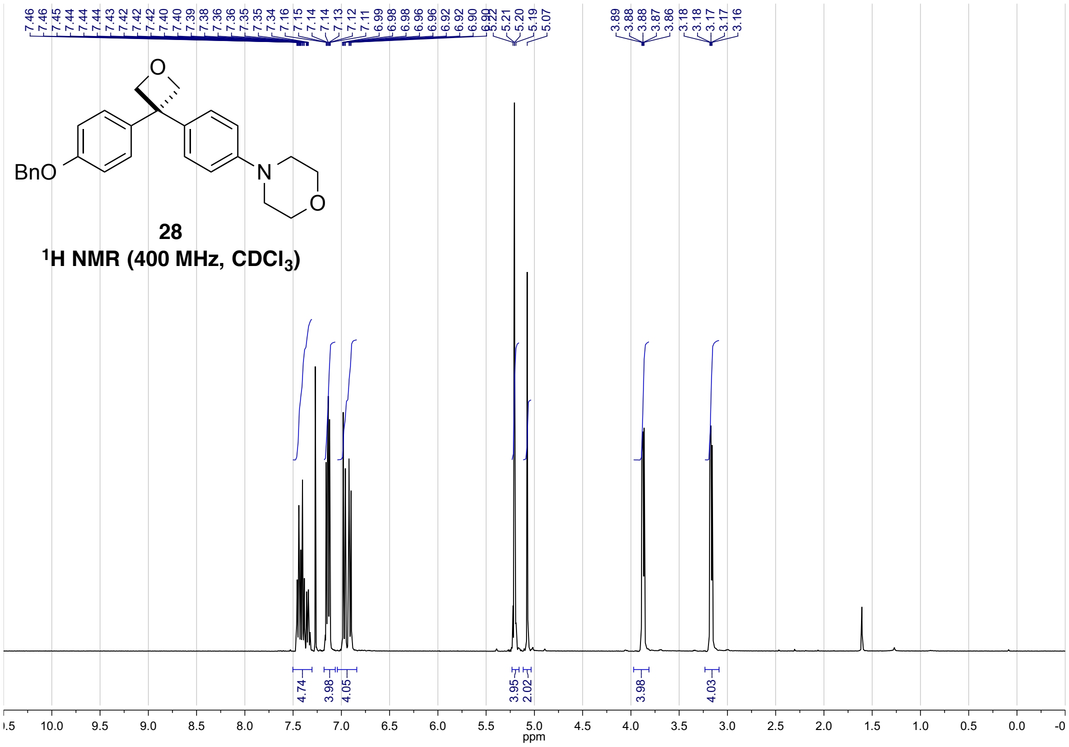

28

^{1}H NMR (400 MHz, $CDCl_3$)

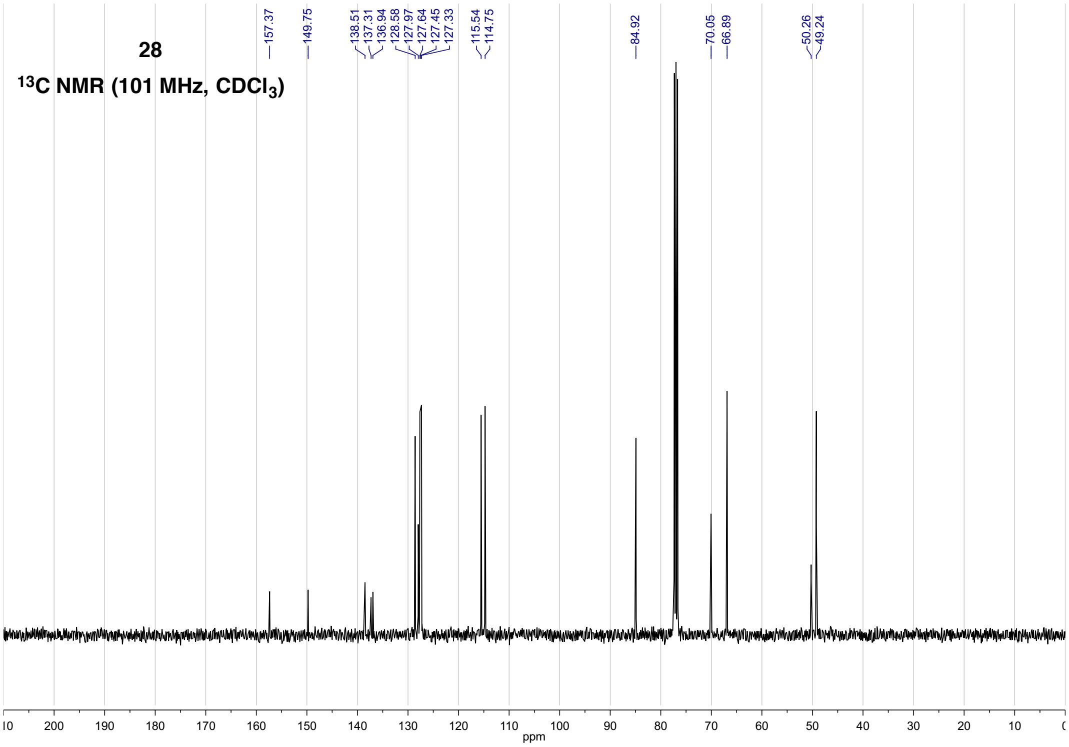

28

^{13}C NMR (101 MHz, $CDCl_3$)

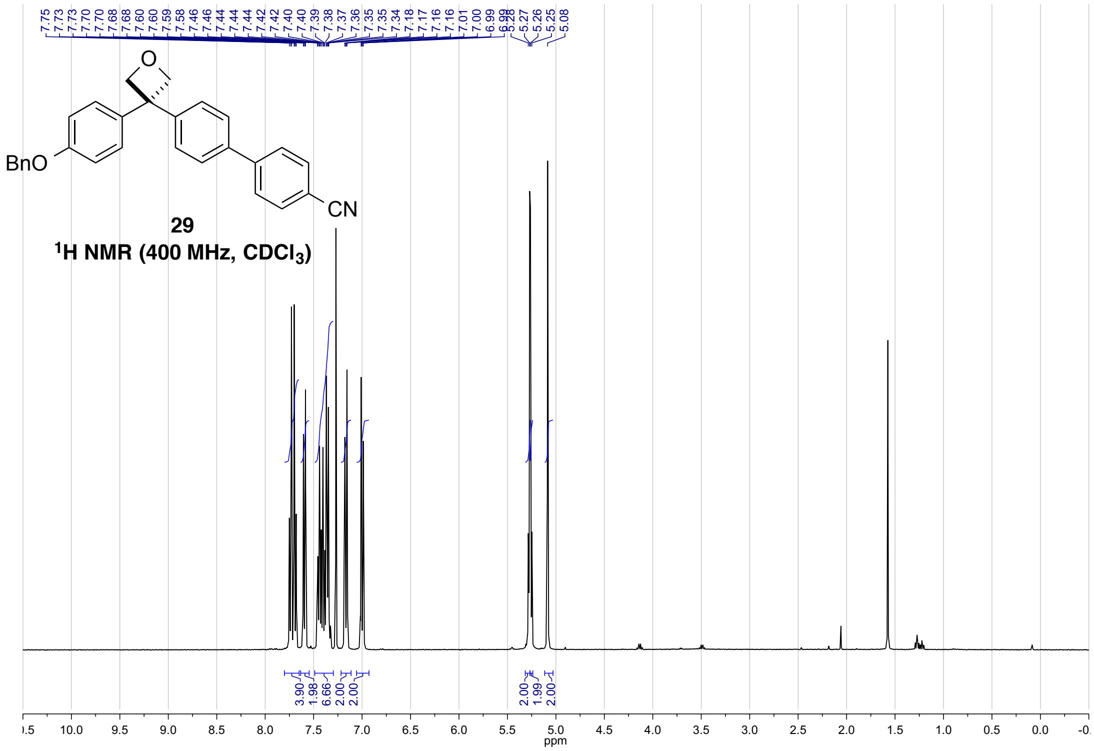
O
BnO
CN
29
1H NMR (400 MHz, CDCl3)
3.90
1.98
6.66
2.00
2.00
2.00
1.99
2.00
ppm

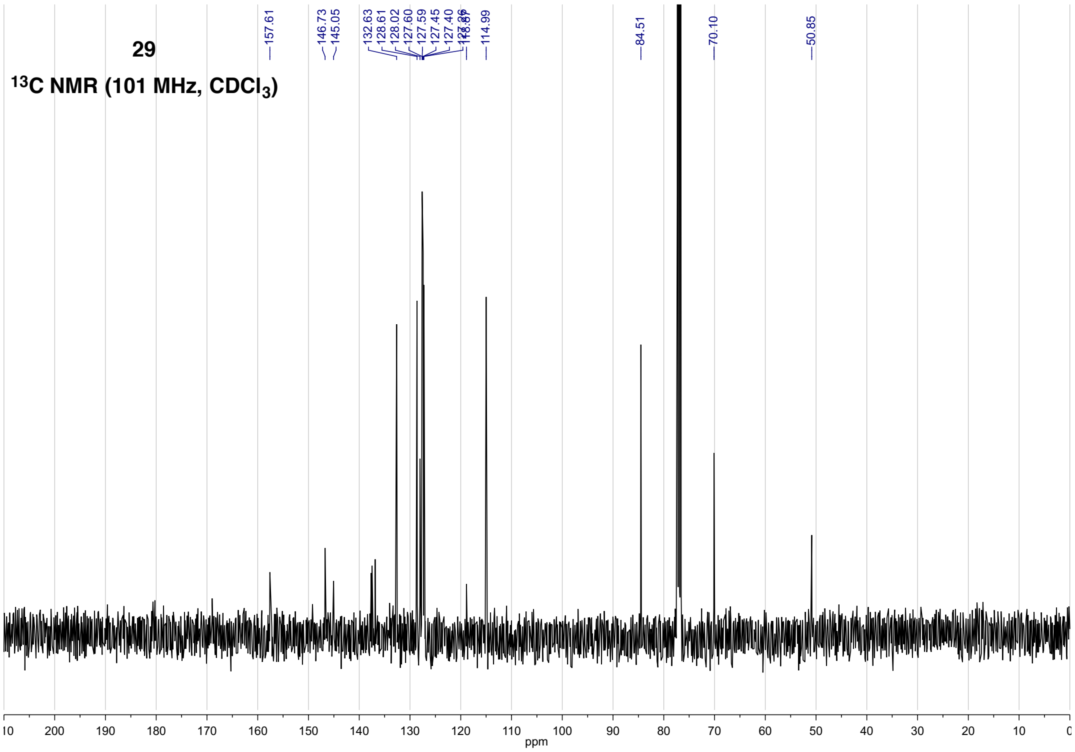
29
13C NMR (101 MHz, CDCl3)
157.61
146.73
145.05
132.63
128.61
128.02
127.60
127.59
127.45
127.40
114.99
84.51
70.10
50.85
ppm
